# Fluorescence of 4-Cyanophenylhydrazones: From Molecular Design to Electrospun Polymer Fibers

**DOI:** 10.3390/molecules30173638

**Published:** 2025-09-06

**Authors:** Paulina Sobczak-Tyluś, Tomasz Sierański, Marcin Świątkowski, Agata Trzęsowska-Kruszyńska, Oskar Bogucki

**Affiliations:** 1 Institute of General and Ecological Chemistry, Lodz University of Technology, Zeromskiego 116, 90-924 Lodz, Poland; tomasz.sieranski@p.lodz.pl (T.S.); marcin.swiatkowski@p.lodz.pl (M.Ś.); agata.trzesowska-kruszynska@p.lodz.pl (A.T.-K.); 2 Łukasiewicz—Lodz Institute of Technology, M. Sklodowskiej-Curie 19/27, 90-570 Lodz, Poland; 3 Łukasiewicz Research Network—Institute of Microelectronics and Photonics, Lotnikow 32/46, 02-668 Warsaw, Poland; oskar.bogucki@imif.lukasiewicz.gov.pl; 4 Institute of Microelectronics and Optoelectronics, Warsaw University of Technology, Koszykowa 75, 00-662 Warsaw, Poland

**Keywords:** phenylhydrazones, fluorescence, substituent effect, polymer matrix, electrospinning

## Abstract

The rational design of advanced functional materials with tailored fluorescence hinges on a profound understanding of the complex interplay between a molecule’s intrinsic structure and its local solid-state environment. This work systematically investigates these factors by employing a dual approach that combines targeted molecular synthesis with the subsequent modulation of the fluorophore’s properties within polymer matrices. First, a series of phenylhydrazone derivatives was synthesized, providing compounds with intense, solid-state fluorescence in the blue spectrum (421–494 nm). It was demonstrated that their photophysical properties were intricately linked to the substituent’s nature, which simultaneously modulated their intramolecular electron density and conformational rigidity while also governing their specific intermolecular packing in the solid state. Subsequently, we investigated the role of the supramolecular environment by embedding two fluorophores with distinct electronic profiles into electrospun poly (N-vinylpyrrolidone) (PVP) and polystyrene (PS) matrices. Our results reveal that the polymer matrix is not a passive host but an active component; it governs dye aggregation, induces significant blue shifts, and most critically, can impart exceptional thermal stability. Specifically, the PVP matrix shielded the embedded dyes from thermal quenching, maintaining robust fluorescence up to 100 °C. By combining molecular-level synthesis with matrix-level engineering, this work demonstrates a powerful strategy for the rational design of emissive materials, where properties like color and operational stability can be deliberately tuned for demanding applications in optoelectronics and sensing.

## 1. Introduction

Fluorescent organic compounds, particularly phenylhydrazones, have garnered substantial interest in recent years due to their distinctive photophysical properties and versatile applications, extending from materials science [1] to biomedical imaging [2]. As an intriguing class of molecules, phenylhydrazones exhibit fluorescence characteristics that can be finely tuned through targeted structural modifications. The intricate relationship between molecular structure and fluorescence behavior in these compounds involves a multifaceted interplay of electronic, steric, and environmental factors, providing a rich arena for both fundamental research and innovative practical implementations [3,4,5]. The photophysical properties of phenylhydrazones are modulated by several critical factors, including the nature and positioning of substituents on the phenyl ring, intramolecular charge transfer (ICT) processes, and diverse intermolecular interactions [6,7]. Theoretical investigations have demonstrated that the excited-state dynamics of these compounds rely on a delicate equilibrium between radiative and non-radiative decay pathways, which can be profoundly altered by structural adjustments. Gaining a comprehensive understanding of these structure–property relationships is essential to the rational design of phenylhydrazones with bespoke fluorescence profiles, thereby enabling their integration into advanced applications such as fluorescent probes [8], organic light-emitting diodes (OLEDs) [9], and photovoltaic devices [10,11,12,13].

Spectroscopic analyses have underscored that the influence of substituents represents one of the paramount determinants of photophysical properties [14,15]. Electron-donating groups (EDGs), such as methoxy (–OCH_3_), dimethylamino (–N(CH_3_)_2_), and diethylamino (–N(CH_2_CH_3_)_2_), enhance fluorescence intensity (Int) and quantum yield (Φ_f_) by increasing the electron density on the phenyl ring, thereby promoting a more pronounced ICT character in the excited state. This ICT enhancement leads to a larger dipole moment in the excited state, resulting in red-shifted absorption and emission spectra, augmented Stokes shifts, and markedly improved fluorescence quantum yields. By contrast, strongly electron-withdrawing groups (EWGs)—notably nitro (–NO_2_) and heavy halogens (e.g., –Br)—often diminish fluorescence in phenylhydrazones by attenuating ICT and promoting non-radiative decay pathways such as intersystem crossing (ISC) and internal conversion (IC) [16,17,18,19,20]. The cyano group (–CN), however, requires a more nuanced treatment. While it withdraws electron density in the ground state, it frequently stabilizes ICT excited states in D–π–A frameworks, producing bathochromic shifts and enlarged Stokes shifts without an inherent penalty in Φ_f_. In rigid or well-dispersed environments that suppress rotational/vibrational dissipation and mitigate aggregation, –CN-substituted phenylhydrazones can therefore remain strongly emissive, as evidenced herein [21]. Substituent position critically modulates electronic communication with the hydrazone π-backbone and the accessible excited-state pathway. Para donor–acceptor arrangements typically maximize resonance and strengthen ICT—often yielding red-shifted spectra and larger Stokes shifts relative to meta-isomers in canonical push–pull systems—whereas ortho substitution can either reduce conjugation through steric twisting or, in the presence of an *o*-OH group, open ESIPT channels that produce intense, large-Stokes-shift emission in rigid media [22,23,24,25,26]. This positional dependency allows precise modulation of the photophysical attributes of phenylhydrazones, facilitating the creation of compounds with optimized spectroscopic features [27,28,29].

The positioning of substituents on the phenyl ring further critically dictates the fluorescent properties of phenylhydrazones. In general, para-substituted variants demonstrate elevated fluorescence intensity and quantum yield relative to ortho- and meta-substituted analogs [30,31]. This superiority stems from the more efficient ICT in para-derivatives, where the substituents are in optimal conjugation with the hydrazone moiety. A systematic examination of the dimethylamino group’s positional effects in a series of phenylhydrazone derivatives revealed that the para-substituted compound exhibited the highest fluorescence quantum yield and the most red-shifted emission maximum, with ortho- and meta-isomers following in performance [32].

In addition to substituent effects, subtle intermolecular interactions—such as hydrogen bonding and π-π stacking—play a pivotal role in shaping the photophysical properties of these compounds [33,34,35,36,37]. These interactions can profoundly influence excited-state dynamics and energy transfer processes [38]. Intramolecular and intermolecular hydrogen bonding can stabilize planar molecular conformations, thereby enhancing electronic conjugation and boosting fluorescence intensity while also facilitating the assembly of supramolecular structures with unique emission signatures. Meanwhile, π-π interactions may promote ordered architectures in the solid state or concentrated solutions, inducing alterations in emission profiles, including bathochromic shifts or variations in quantum yield [39,40]. The synergistic effects of these interactions can yield sophisticated supramolecular ensembles with exceptional photophysical traits, unlocking novel opportunities in the development of advanced fluorescent materials [41,42].

Given that phenylhydrazones typically exist as solid-state compounds, their fluorescence in aggregated forms is particularly relevant where intermolecular forces dominate. While numerous techniques (such as solvent casting) exist for incorporating fluorophores into polymer matrices, melt blending or in-situ polymerization-electrospinning stands out as a particularly advantageous method due to its simplicity, cost-effectiveness, and versatility in producing nanofibers with exceptionally high surface area-to-volume ratios, enabling uniform dispersion of the active molecules and enhanced control over morphology [43,44,45]. This approach not only facilitates the fabrication of fluorescent thin films and materials suitable for applications in sensors, biomedical devices, and optoelectronics but also minimizes common drawbacks like uneven distribution or phase separation [46,47,48]. In this context, electrospinning yields hydrazone-doped polymers, wherein phenylhydrazones are embedded within supportive polymeric carriers such as polystyrene or polyvinylpyrrolidone, combining the intrinsic emissive qualities of the fluorophores with the structural integrity, flexibility, and processability of the polymers. Such systems not only alleviate issues like aggregation-caused quenching but also introduce responsiveness to external stimuli including temperature, which can modulate molecular mobility, intermolecular interactions, and phase transitions within the matrix, thereby influencing fluorescence efficiency and spectral characteristics.

This study encompasses a thorough exploration of a series of phenylhydrazones synthesized by reacting 4-cyanophenylhydrazine hydrochloride with 14 diverse aldehydes and a ketone. A suite of advanced analytical techniques was employed for in-depth characterization, including single-crystal X-ray diffraction, infrared (IR) spectroscopy, UV-Vis spectroscopy, proton nuclear magnetic resonance (^1^H NMR) spectroscopy, and differential scanning calorimetry (DSC). Complementing these experimental methods, quantum mechanical calculations were conducted to provide a more holistic insight into the underlying phenomena. The primary aim of this research was to elucidate the impacts of diverse structural elements on the fluorescent behavior of the investigated compounds, with special emphasis on the roles of electron-withdrawing (EWG) and electron-donating (EDG) substituents. A key focus was also placed on the effects of intermolecular interactions on observed fluorescence. Extending beyond isolated molecules, the work investigated the temperature-dependent fluorescence of electrospun hydrazone-doped polymer matrices, revealing how thermal variations affect emissive performance in these integrated systems. This integrated methodology fosters a profound understanding of structure–property relationships in phenylhydrazones, potentially guiding the innovation of novel materials with tailored fluorescent attributes.

## 2. Results and Discussion

### 2.1. Crystal Structure Analysis

**Figure 1:**
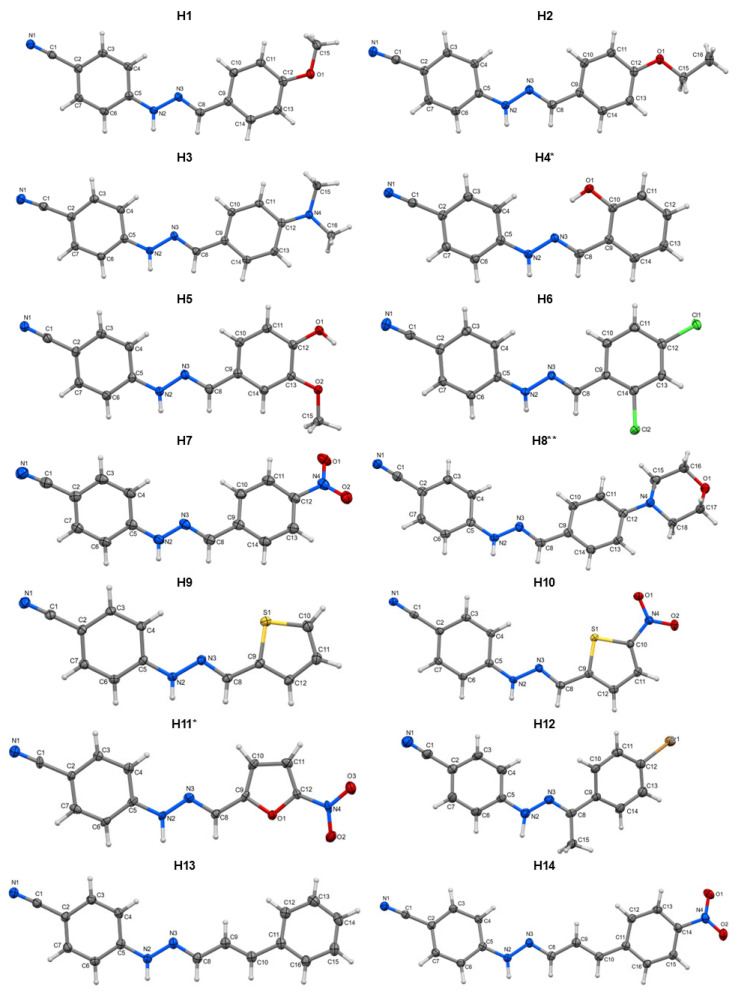
Molecular structures of phenylhydrazones **H1**–**H14**. For the asterisked compounds, which feature multiple molecules in the asymmetric unit, Z′ = 2 (*) and Z′ = 4 (**), a single molecule is depicted for simplicity.

The crystal structures of all fourteen synthesized hydrazone derivatives were successfully determined using SC-XRD analysis (Table S1). It confirmed that all structures were solvent-free (Figure 1). The structure of **H4** has been published as a crystallographic communication (without detailed structural analysis or any additional investigations), and it was determined from data collected at 150 K [49]. Therefore, to provide a uniform basis for structural comparison across the entire series, the crystal structure of **H4** was redetermined at 100 K for the present study.

The bond lengths and angles within the hydrazone moiety showed remarkable consistency across the series (Tables S2 and S3) and aligned with typical values reported for hydrazones in the literature [50]. The bond angles around the amine nitrogen atom N2 are consistent with sp^2^ hybridization, confirming that the lone electron pair on this atom participates in the extended conjugated π-system with the N3-C8 imine bond, which is a characteristic structural feature of hydrazone compounds. The molecules generally adopt relatively planar conformations, as evidenced by the interplanar angles between the aromatic rings and the torsion angles within the hydrazone backbone (Table S4). The torsion angles along the C5-N2-N3-C8-C9 chain demonstrate a *trans*-*trans* conformation in all compounds (*trans-trans-trans-trans* in compounds **H13** and **H14**), with deviations from the ideal 180° value not exceeding 10°. Larger deviations from overall planarity arise from rotations of the aromatic rings. However, the dihedral angles between the mean planes of these rings generally remain below 15°, with only one exception observed in **H12**, where this angle exceeds 20°. This structural anomaly can be attributed to the presence of a methyl group attached to the C8 atom of the hydrazone moiety. The steric hindrance introduced by this methyl substituent forces the adjacent benzene ring to adopt a more pronounced twist relative to the molecular plane. The N1-C1 bond lengths are consistent with the characteristic values for cyano groups functioning as substituents on benzene rings (1.138 Å) [51].

The crystal structures of the investigated hydrazones are stabilized by a variety of intermolecular interactions, which are comprehensively summarized in Table 1. The N2 amine group functions as a donor for classical hydrogen bonds (H-bonds) throughout the series, with additional H-bond donation provided by hydroxyl groups in compounds **H4** and **H5** (Table S5). The most frequently observed H-bond pattern in this series is the C(8) chain motif formed between the N2 amine group and the N1 nitrogen of the cyano moiety (present in 12 out of 14 studied compounds, Figures S1–S3). In the structures of **H7** and **H11**, the N2 amine group participates in a bifurcated H-bond. Besides forming the C(8) chain with the cyano group, it also engages in a second H-bond, where an oxygen atom of the nitro group acts as the acceptor. This interaction results in the formation of the C(10) chain motif in **H7** and the R_2_^2^(9) ring motif in **H11** (Figures S2 and S3). Compound **H5** exhibits distinct H-bond synthons, where the hydroxyl group serves as a hydrogen bond donor to the cyano group, forming the C(15) motif, while the N2 amine group interacts with the N3 imine nitrogen, creating the C(3) motif (Figure S4). In addition to these intermolecular interactions, the hydroxyl groups in both **H4** and **H5** also form an intramolecular hydrogen bond with the imine nitrogen N3 and the methoxy oxygen O2, respectively. Interestingly, compound **H3** lacks classical H-bonds. Instead, the N2 amine group engages in NH···π interactions with the π-system of benzene ring (Figure S5).

**Scheme 1:**
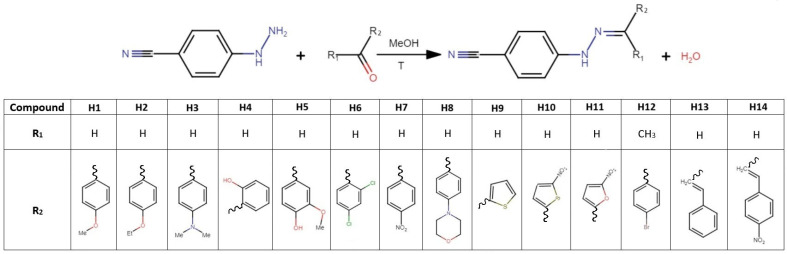
Synthesis of the studied compounds.

Typical π···π stacking interactions between aromatic rings are prevalent in the majority of the structures (Table S6). They present different types of head-to-tail (HT1 and HT2, where 1 and 2 indicate the number of aromatic rings of one molecule, which are involved in the stacking interaction with another molecule) and head-to-head (HH1 and HH2) arrangements organizing the molecules into dimers or extended supramolecular columnar systems (Figures S6–S12). A notable feature observed in structures lacking these typical ring-stacking motifs is the prevalence of alternative π-system engagements. Specifically, interactions were observed between the π-electrons of an aromatic ring and the π-systems of multiple bonds. Both the hydrazone moiety, via its C=N–N core, and the cyano substituent, via its C≡N triple bond, engage in this type of π···π interaction, characterized by a parallel orientation of the multiple bond relative to the aromatic ring plane (Figures S13–S15). This analogous engagement of different π-systems appears to serve as a key stabilizing force when typical π···π stacking is sterically hindered or otherwise unfavorable. Finally, the crystal structures are further stabilized by numerous weak CH···X interactions (where X = N, O, Cl, S) and hydrophobic H···H contacts observed throughout all compounds.

### 2.2. Analysis of Absorption Properties of Phenylhydrazones

The diffuse-reflectance UV-Vis spectra of phenylhydrazones **H1**–**H14** show up to four broad absorption bands in the range of 200–400 nm (Figures S16–S29, Table S7). These bands originate from π → π* electronic transitions delocalized across the entire conjugated molecular scaffold (Figures S30–S43). The experimental spectra are well-reproduced by our TD-DFT calculations, with the positions of the dominant bands typically matching within a 20–40 nm range. This good agreement is a direct result of the computational strategy adopted from our previous work, where multi-molecule models (3–4 molecules) were used instead of isolated single-molecule calculations [52]. This approach better reflects the solid-state environment by accounting for significant intermolecular interactions. As a result, it allows for the identification of key electronic features, such as ligand-to-ligand charge-transfer (LLCT) transitions, which are crucial to accurately interpreting the solid-state UV-Vis spectra but are often missed in single-molecule models. Despite the overall success of this method, some limitations were apparent, particularly for compounds **H8** and **H9**. For **H8**, no transitions were calculated above 450 nm, failing to account for the experimental shoulders at 478 nm and 580 nm. Similarly, for **H9**, the single calculated transition in this region has an oscillator strength near the detection limit (ƒ ≈ 0.001). These discrepancies may suggest the presence of electronic transitions with oscillator strengths below the numerical detection threshold or other complex solid-state effects not fully captured by the model. For the remainder of the series, each experimental band contains at least one calculated transition with a sufficient oscillator strength (ƒ ≈ 0.07–3.6) to account for the observed intensity. However, the theoretical manifold consistently shows several closely spaced states for each band, underscoring the composite nature of the measured absorption maxima.

A detailed analysis of the spectra reveals a clear dependence on the substituents. Para-alkoxy substituents (**H1**, **H2**) cause a 20–30 nm bathochromic (red) shift of the lowest-energy band compared to the ortho-hydroxy (**H4**) and meta-OMe/para-OH (**H5**) derivatives, which are blue-shifted due to a shorter effective conjugation path. Replacing the donor with the much stronger para-NMe2 group (**H3**) produces the largest red-shift and gives the lowest-energy states a distinct LLCT character. A similar deep-UV LLCT component is also predicted for the morpholinyl derivative H8. In contrast, electron-withdrawing or π-extending substituents shift the spectra even further to the red. A para-NO_2_ group (**H7**) introduces an additional charge-transfer band in the blue-green region. Nitro-substituted thiophene and furan rings (**H10**, **H11**) have a similar effect, and in these cases, TD-DFT calculations localize the acceptor orbital density on the nitro-bearing heterocycle. The thiophene analogue without a nitro group (**H9**) shows the longest experimental absorption wavelength in the set, although only its shorter-wavelength LLCT band is captured effectively by theory. In the para-bromo derivative **H12**, the LLCT contribution is shifted to higher energy. Finally, incorporating a cinnamyl fragment (**H13**) or its para-nitro variant (**H14**) broadens the low-energy band, which extends to ca. 500 nm in both experiment and theory.

In summary, the data demonstrate that stronger electron donors systematically red-shift the main π → π* absorption band, while donor–acceptor or π-extended systems introduce new, lower-energy LLCT bands. The generally good agreement between experiment and our multi-molecule TD-DFT model validates the theoretical description of the excited states. It also highlights specific cases, such as the long-wavelength regions of **H8** and **H9**, where very weak transitions or other solid-state phenomena may play a role and warrant further investigation.

### 2.3. Analysis of Fluorescent Properties of Phenylhydrazones

**Figure 2:**
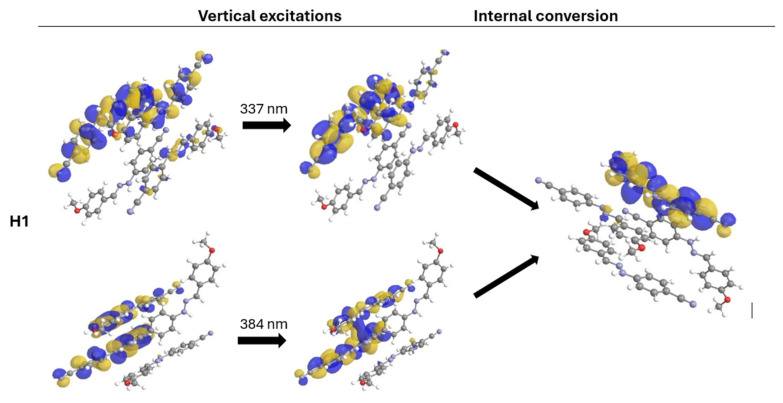

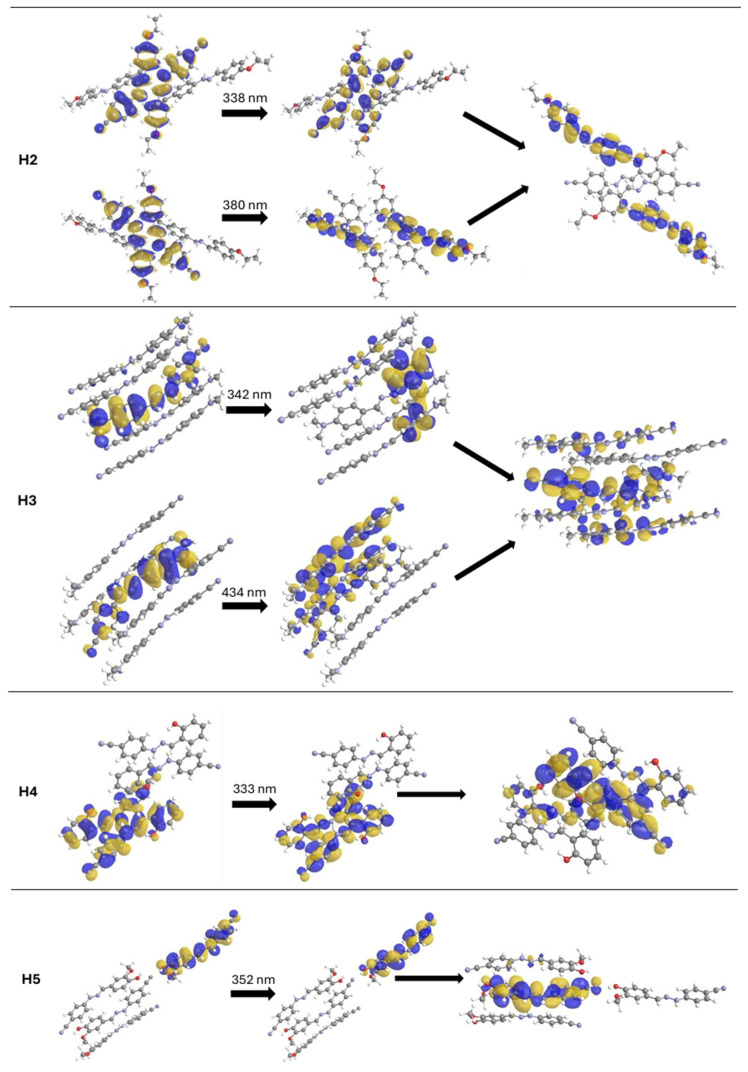

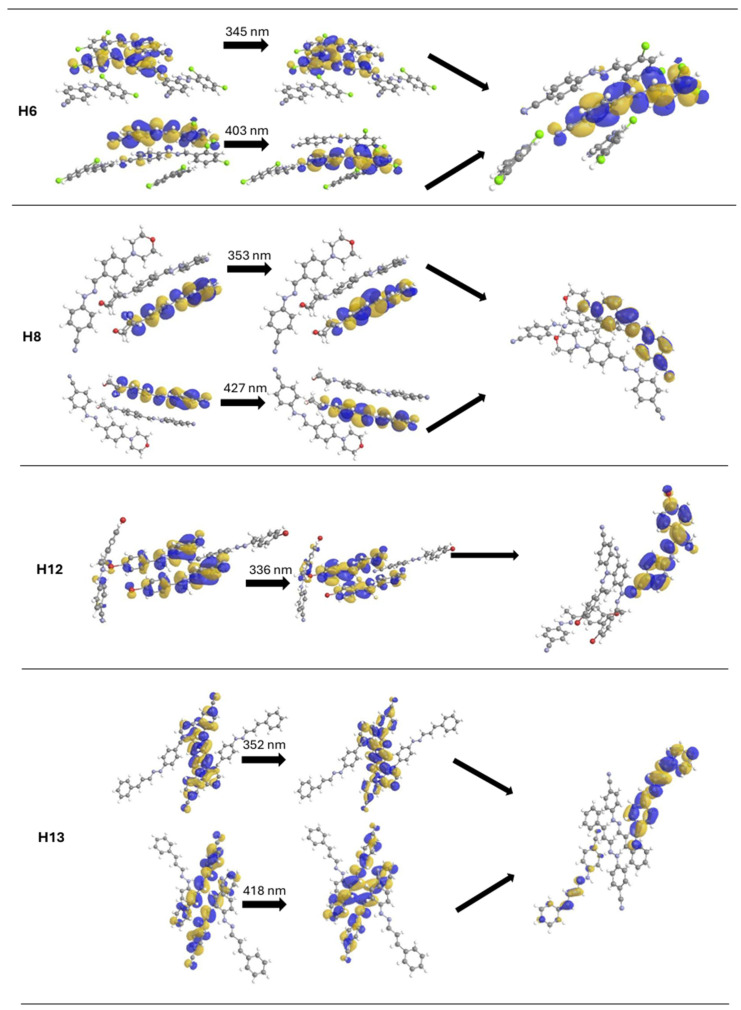
Vertical excitations assigned to the observed fluorescence maxima, followed by internal conversion (IC) to the lowest-energy excited singlet state (S1). Orbital-density maps represent the electronic distributions responsible for these fluorescence-related excitations. The S1 orbital-density state is shown qualitatively.

Nine of the fourteen synthesized phenylhydrazones exhibit solid-state fluorescence (Figure S44). Their emission spectra show either one or two global maxima, depending on the excitation wavelength (Table 2). The first set of maxima, observed upon excitation in the 333–353 nm range, originates mainly from π → π* transitions (Figure 2). The second set of bands, appearing with excitation between 380–434 nm, is attributed mostly to π → π* transitions of both an intraligand and an interligand (LLCT) nature. For all fluorescent compounds, the emission occurs in the range of 421–494 nm. Notably, for compounds displaying two emission maxima, the emission wavelength is independent of the excitation wavelength. This observation is consistent with Kasha’s rule, which states that emission occurs from the lowest vibrational level of the first excited singlet state (S1) following rapid internal conversion (Figure 2). In these instances, excitation at the longer-wavelength LLCT band produces higher fluorescence intensity but lower quantum yield compared to the shorter-wavelength π → π* band. This suggests that the LLCT state benefits from stronger absorption while being more susceptible to non-radiative decay pathways that diminish overall emission efficiency.

The observed fluorescence properties are a result of a complex interplay between molecular structure, including molecular geometry and the electronic nature of substituents, and the supramolecular architecture defined by intermolecular interactions. All synthesized compounds adopt a relatively planar molecular geometry, with a trans-trans conformation of the hydrazone backbone. This inherent planarity provides a rigid, conjugated scaffold that restricts non-radiative decay pathways, such as vibrational relaxation and internal rotation, which generally promotes fluorescence. This makes the core molecular skeleton a good starting point for emissive properties. The significant diversity in fluorescence behavior across the series can therefore be attributed to the specific effects of the substituents and the different types of intermolecular packing.

The highest fluorescence intensities and quantum yields (Φ_f_) are observed for compounds **H1**, **H2**, **H3**, **H8**, and **H13**. These derivatives all contain electron-donating substituents, which are known to enhance emission by increasing the electron density of the π-system. Crucially, their supramolecular structures are devoid of classical π···π stacking interactions between the aromatic rings. Instead, the molecules are assembled by intermolecular hydrogen bonds, and in some cases they form discrete dimers organized by π···π interactions between hydrazone moieties and aromatic rings, which together help to suppress aggregation-caused quenching (ACQ) and favors the radiative decay channel. A notable exception is the morpholinyl derivative **H8**, which combines high steady-state intensity with a lower quantum yield. The morpholine ring provides a large absorption cross-section, but its conformational flexibility supplies low-frequency vibrational modes that open efficient non-radiative channels. In addition, the ether oxygen introduces a low-lying n → π* manifold whose energetic proximity to the emissive π → π* state enhances vibronic coupling and accelerates internal conversion (and modest intersystem crossing).

Compounds **H6** and **H12** exhibit moderate fluorescence intensity, attributable to their halogen substituents (chloro and bromo). These introduce competing effects: acting as electron-donating groups via resonance while deactivating the aromatic ring inductively. Additionally, the heavy-atom effect from these substituents accelerates intersystem crossing (ISC) to the non-emissive triplet state, thereby diminishing fluorescence. Moreover, π···π stacking interactions in their crystal lattices further contribute to emission quenching.

The lowest, yet still observable, fluorescence is found in the hydroxy-substituted compounds **H4** and **H5**. The hydroxyl groups form intramolecular hydrogen bonds, which can open up an efficient non-radiative decay channel via Excited-State Intramolecular Proton Transfer (ESIPT). This effect, combined with the presence of π···π stacking columns in their crystal packing, leads to very low fluorescence intensities and quantum yields.

**Figure 3:**
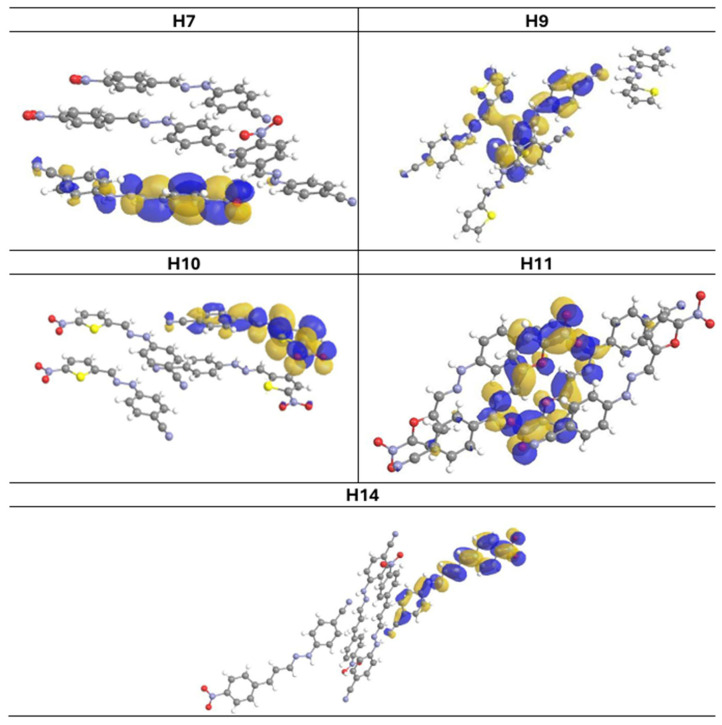
The S1 orbital-density states of non-emissive phenylhydrazones.

Finally, compounds **H7**, **H9**, **H10**, **H11**, and **H14** are completely non-emissive (dark). This total fluorescence quenching is caused by the presence of strongly electron-withdrawing groups (Figure 3), such as the nitro group, or π-deficient heterocycles like thiophene. Such substituents are well-documented fluorescence quenchers [53,54,55,56,57,58]. They function by creating low-lying, non-emissive charge-transfer states or by promoting very fast, efficient intersystem crossing, which effectively funnels the excited-state population away from the radiative fluorescence pathway.

Fluorescence lifetime measurements were performed for all emissive compounds in the solid state. The determined lifetimes (τ) are short, ranging from 0.19 to 12.5 ns, which is characteristic for organic fluorophores that do not contain metal cations. A comparison of the fluorescence lifetimes with the quantum yields allows a qualitative estimate of the radiative (k_r_) and non-radiative (k_nr_) rate constants (Table S8) for each emissive derivative [59,60]. Compounds **H1** and **H2**, whose sub-nanosecond lifetimes are paired with Φ_f_ ≈ 0.25, exhibit exceptionally efficient radiative decay, a result that can be traced to a highly allowed π → π* transition and a crystal environment that effectively dampens low-frequency vibrational modes. **H3**, by contrast, combines a similar yield with a markedly longer lifetime (~10 ns), indicating that both k_r_ and k_nr_ are intrinsically slow—a behavior expected for a donor-π-acceptor system in which the LLCT contribution lowers the oscillator strength while the lattice almost eliminates non-radiative loss. The cinnamyl derivative **H13**, with the highest Φ_f_ (~0.40) and a mid-range τ (~5 ns), falls between these extremes: its extended conjugation boosts k_r_, yet internal motion is still sufficiently restrained to keep k_nr_ moderate.

For the morpholinyl compound **H8** the opposite pattern emerges: a Φ_f_ below 0.05 coupled with a sub-nanosecond lifetime implies that k_nr_ overwhelms k_r_. Here, conformational flexibility of the morpholine together with the plausible presence of low-lying n → π* states associated with the ether oxygen provide efficient non-radiative funnels. Halogenated **H6** and **H12** share modest quantum yields with τ ≈ 1 ns; the heavy-atom effect accelerates intersystem crossing, enhancing k_nr_ despite reasonably allowed radiative transitions. The hydroxy derivatives **H4** and **H5** combine very short lifetimes with very low Φ_f_, indicating that ultrafast non-radiative channels prevail. For both compounds, features consistent with ESIPT together with π···π stacking provide efficient quenching pathways, which accounts for their weak steady-state fluorescence.

### 2.4. Modulation of Hydrazone Properties in Electrospun Polymer Matrices

For a detailed investigation of solid-state environmental effects on phenylhydrazone fluorescence, compounds **H3** and **H13** were selected from the synthesized series based on their superior photophysical performance and contrasting structural profiles. This pairing provides a robust framework for comparative analysis, as both compounds exhibit among the highest fluorescence quantum yields and emission intensities in the series, ensuring strong and readily measurable optical signals for characterization.

These compounds represent two distinct electronic architectures that enable a comprehensive structure–property analysis. Compound **H3** exemplifies a classic donor-π-acceptor (D-π-A) system, incorporating a potent electron-donating dimethylamino group and an electron-withdrawing cyano group. This configuration promotes a strong intramolecular charge transfer (ICT) character, making it an ideal probe for studying how environments of varying polarity modulate a highly polarized, solvatochromic fluorophore. In contrast, H13 introduces a different structural modification to the core scaffold. Derived from cinnamaldehyde, it uniquely incorporates a vinylene bridge (–CH=CH–) that extends the π-conjugated system between the phenyl ring and the hydrazone core. Its selection therefore allows for an investigation into how the polymer matrix interacts with a fluorophore whose optical properties are governed by the physical length and planarity of its π-system (**H13**) rather than by strong substituent-induced charge transfer effects (**H3**).

To evaluate the influence of the matrix, the selected hydrazones were incorporated into two polymer systems chosen for their distinct physicochemical properties: poly (N-vinylpyrrolidone) (PVP), a water-soluble and non-toxic polymer, and polystyrene (PS), a water-insoluble and durable material. This strategic choice was aimed at directly investigating the influence of matrix characteristics such as polarity and hydrophilicity on the resulting fiber morphology and the optical properties of the embedded dyes. Both the materials and pure reference mats were fabricated via electrospinning, and the successful incorporation of the dyes into the fibers was confirmed by Attenuated Total Reflectance Fourier-Transform Infrared (ATR-FTIR) spectroscopy (Figures S45–S48). For the PVP-based matrices, the spectra showed clear evidence of the hydrazone’s presence through the appearance of a new, distinct band at approximately 1602 cm^−1^, characteristic of the C=N imine bond stretching vibration. The intensity of this band became more prominent at higher dye loading, confirming a concentration-dependent incorporation. Furthermore, the spectrum of the PVP matrix doped with **H13** exhibited additional new bands at 1565 and 1533 cm^−1^, attributable to the C-C ring stretching vibrations of the dye’s phenyl group. In contrast, analysis of the PS-based materials was more complex due to the inherent aromatic nature of the polystyrene backbone, whose own vibrational bands overlap with the key diagnostic region. Consequently, only subtle increases in the intensity of the band around 1602 cm^−1^ were observed upon dye incorporation.

#### 2.4.1. Morphological Analysis and Structural Stability

SEM analysis revealed distinct morphological characteristics influenced by polymer type and dye incorporation, highlighting the critical role of matrix properties in fiber formation. PVP-based materials, including both reference and dye-doped samples, consistently produced smooth, defect-free fibers with diameters ranging from several hundred nanometers to approximately 1.2 µm, forming a heterogeneous mix of nano- and microfibers (Figures S49–S58). Notably, dye incorporation did not significantly alter fiber morphology compared to pure PVP matrices.

PS-based materials exhibited markedly different behavior, with pronounced morphological changes upon dye incorporation. While reference PS fibers maintained smooth surfaces, **H3** incorporation induced characteristic wrinkled surface features, and **H13** integration resulted in densely distributed fine porous morphology (Figures S59–S61). PS fibers were consistently thicker (8–13 µm, Figures S62–S64) due to higher solution viscosity during processing. These morphological modifications indicate dye-mediated effects on solution rheology and solvent evaporation dynamics during electrospinning.

Water stability assessments revealed complementary but distinct stabilization mechanisms. PS matrices demonstrated exceptional stability, remaining intact after 24 h in aqueous environments with no detectable dye leaching and preserved fluorescence properties (Figure S65). While pure PVP dissolved rapidly in water, the **H13**-doped PVP matrix (1% loading) exhibited significantly enhanced stability, maintaining structural integrity (Figure S66). This enhanced stability is attributed to a pseudo-crosslinking effect involving direct dye–polymer interactions that alter the local polymer environment. ATR-FTIR analysis (Figures S45–S48) provides spectroscopic evidence through a systematic, concentration-dependent blue shift of the PVP carbonyl band upon **H13** incorporation. This spectral change indicates that the **H13** molecules disrupt the native intermolecular associations of the PVP chains, establishing a new supramolecular network. This network effectively restricts polymer chain mobility and modifies the local hydration environment, leading to the observed water resistance. In contrast, **H3**-doped PVP matrices dissolved similarly to pure PVP, indicating dye-specific interaction mechanisms.

These observations reveal contrasting stabilization mechanisms, with PVP exhibiting bulk property modifications, such as enhanced solubility resistance, without morphological alterations—an effect attributed to molecular-scale network formation. In contrast, PS shows morphological modifications without significant changes in bulk properties, which result from process-induced effects. This mechanistic duality highlights the importance of selecting the appropriate matrix to achieve designed material properties.

#### 2.4.2. Photophysical Properties and Dye–Polymer Interactions

**Figure 4:**
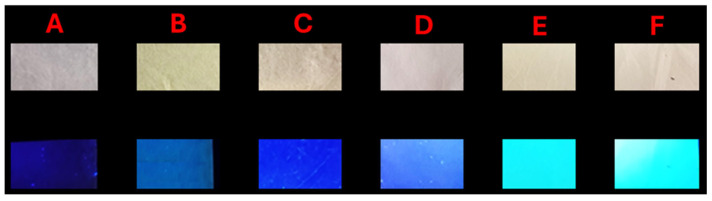
Electrospun materials under daylight (**top row**) and UV light (365 nm, **bottom row**). Where: (**A**)-PS_R, (**B**)-1% **H3**/PS, (**C**)-1% **H13**/PS, (**D**)-PVP_R, (**E**)-1% **H3**/PVP, (**F**)-1% **H13**/PVP.

**Figure 5:**
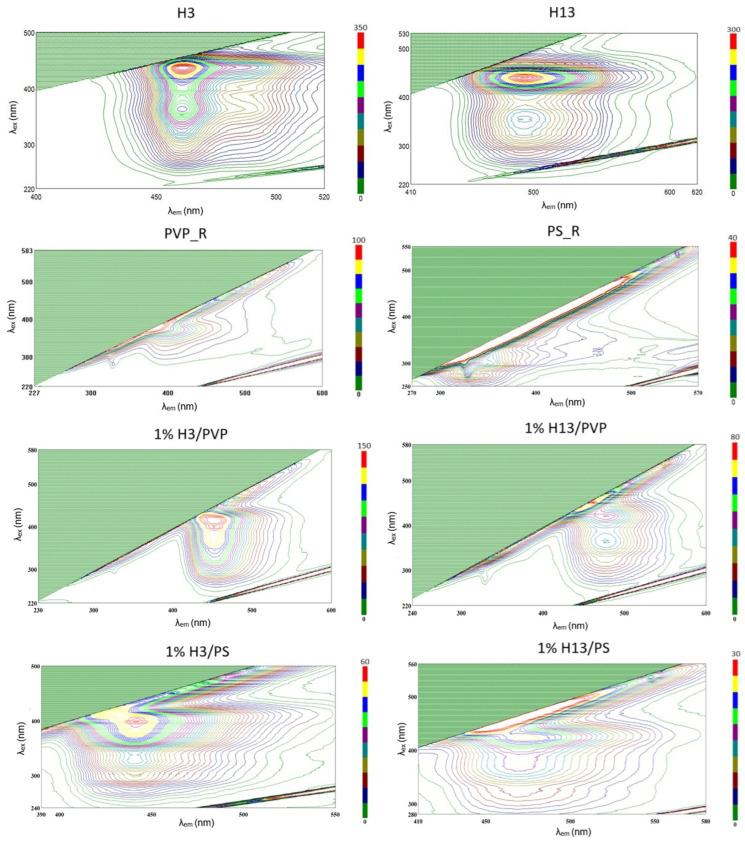
3D-EEM fluorescence spectra of the phenylhydrazones (**H3** and **H13**), the pure polymers (PVP_R and PS_R), and dye-doped mats.

As a visual overview, photographs of the reference mats and dye–polymer materials under ambient light and 365 nm UV are provided in Figure 4, illustrating the matrix-dependent appearance. The fluorescence properties of **H3** and **H13** were systematically investigated both in their pure powder forms and within dye–matrix systems (1% **H3**/PVP, 1% **H13**/PVP, 1% **H3**/PS, 1% **H13**/PS) using 3D excitation-emission matrix spectroscopy (3D-EEM) (Figure 5). Analysis of the pure polymer matrices revealed important differences in their baseline emission profiles. The pure PVP mat was confirmed to be effectively non-fluorescent, exhibiting only scattering artifacts. In contrast, the pure PS mat displayed a weak fluorescence, attributed to its aromatic backbone, with both its excitation and resulting broad emission being confined to the deep-UV region. While the **H3** and **H13** dyes also possess strong excitation bands in the UV region, their subsequent fluorescence occurs entirely in the visible spectral window. The crucial point is the clear spectral separation between the deep-UV emission of the PS matrix and the visible-range emission of the hydrazones. This lack of spectral overlap ensures that even when the systems are excited with UV light, the intrinsic emission of the PS does not interfere with the measurement of the dye’s fluorescence. Therefore, the pronounced emission observed in the visible range for the dye-doped systems originates exclusively from the embedded fluorophores. Interactions between the dyes and the polymer matrices modulate the energetic states of the fluorophores, leading to significant changes in their spectroscopic properties compared to dye powders. The key parameters defining these excitation and emission maxima are summarized in Table S9.

Dye–matrix systems display distinct fluorescence characteristics relative to pure dyes, with marked blue shifts observed in both the excitation and emission bands (Figures S67–S74). These features reflect strong dye–polymer interactions and suggest considerable disaggregation of the dye molecules within the polymer environments.

The observed blue shifts result from molecular-level processes, where dispersion within the polymer matrix promotes disaggregation of the dye, thereby favoring emission at shorter wavelengths compared to aggregated powders. Such spectral shifts can be attributed to the influence of matrix polarity on the electronic states of the fluorophores. These shifts are somewhat more pronounced in hydrophobic PS matrices than in hydrophilic PVP, a consequence of reduced stabilization of the fluorophore’s excited state in the nonpolar PS environment, which increases the energy gap and enhances the blue shift (Figures S67–S74, Table S9) [61]. These shifts are somewhat more pronounced in hydrophobic PS matrices than in hydrophilic PVP, a consequence of reduced stabilization of the fluorophore’s excited state in the nonpolar PS environment, which increases the energy gap and enhances the blue shift (Table S9) [61].

The rigid nature of the polymer matrices can also limit the internal motions of the dye molecules, known as the RIM effect, which affects the energies of electronic transitions and supports the blue shift. The differences in dye–matrix interactions are also important. In PVP, hydrogen bonds can form between the carbonyl groups of the polymer and the N–H groups of the dye, while in PS, interactions such as π···π stacking between aromatic rings may play a role. The smaller shift differences noted for **H13** in both matrices suggest that this compound is less sensitive to polarity changes or that specific interactions have a comparable influence in both environments (Figures S67–S74, Table S9) [62].

Steric effects imparted by the polymer chains and fiber morphology additionally shape dye conformation, local concentration, and interchromophoric interactions. In PVP, smooth nanofiber formation facilitates uniform dye dispersion, whereas thicker, structurally modified PS fibers (wrinkled for **H3**, porous for **H13**) create a complex environment that can influence dye distribution and light scattering.

The nature of dye–polymer interactions indicates the presence of two concurrent mechanisms. In PVP, the presence of **H13** induces changes in bulk material properties, such as enhanced water resistance via pseudo-crosslinking, without altering fiber morphology. In contrast, in PS, morphological modifications such as wrinkling or porosity are observed without significant changes in the bulk properties, which is likely a result of the interplay between solution rheology and evaporation kinetics during electrospinning.

Fluorescence intensities are found to be lower in PS than in PVP, a phenomenon ascribed to inhomogeneous dye dispersion leading to aggregation and quenching, π···π interactions providing non-radiative relaxation pathways, and differences in refractive index affecting light extraction.

#### 2.4.3. Influence of Dye Concentration on Quantum Yield in the PVP Matrix

**Figure 6:**
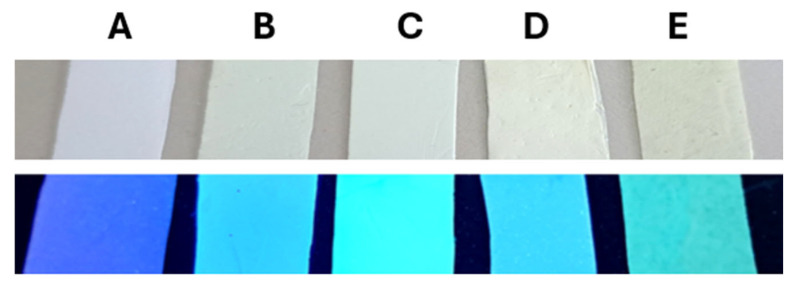
Electrospun PVP materials with different dye concentrations under daylight (**top row**) and UV light (365 nm, **bottom row**). Where: (**A**)-PVP_R, (**B**)-1% **H3**/PVP, (**C**)-1% **H13**/PVP, (**D**)-5% **H3**/PVP, (**E**)-5% **H13**/PVP.

To investigate concentration-dependent photophysical properties, poly (N-vinylpyrrolidone) (PVP) samples containing 1 wt% and 5 wt% of **H3** and **H13** hydrazones were prepared (Figure 6).

Excitation-emission spectral analysis showed that blue shifts relative to the powder forms were more pronounced at lower dye concentrations of 1 wt%, which indicates enhanced dye–matrix interactions and more efficient molecular disaggregation. At higher concentrations of 5 wt%, dye–dye interactions limit the extent of disaggregation, resulting in smaller spectral shifts while the shapes of the emission bands remain preserved.

Quantum yield measurements (Table S10) revealed an additional level of complexity in the concentration-dependent behavior of dyes in PVP matrices. Contrary to typical expectations, both **H3** and **H13** exhibited higher quantum yields at 5 wt% than at 1 wt%. This unexpected trend suggests that the restriction of intramolecular motion (RIM effect) becomes the dominant factor at higher dye concentrations, effectively reducing non-radiative deactivation pathways despite the presence of dye–dye interactions. The balance between spectral shifts and quantum efficiency indicates that optimal dye dispersion, which favors blue shifts, occurs at lower concentrations, whereas optimal fluorescence brightness is achieved at higher concentrations, where molecular motion within the polymer matrix is more restricted.

#### 2.4.4. Thermal Stability of Fluorescence Properties

The thermal stability of the fluorescent materials was evaluated to assess their performance under elevated temperatures, revealing significant differences between the two polymer matrices. The PS-based materials exhibited limited thermal resistance, melting at elevated temperatures (Figure S75). This behavior restricted detailed temperature measurements to room temperature only (Figure S76), confirming the PS matrix’s unsuitability for high-temperature applications. In contrast, the pure PVP reference mat and the dye-doped systems containing 1 wt% of **H3** or **H13** were all successfully analyzed across the full temperature range.

Analysis of the control sample PVP_R confirmed excellent thermal resistance and structural integrity up to 100 °C (Figure S77). Spectral analysis revealed that the polymer matrix itself contributes negligibly to the emission signal, with background fluorescence remaining at minimal levels regardless of temperature. Most importantly, the dye-doped materials exhibited remarkable thermal stability of fluorescence properties. For both dye–matrix systems, temperature elevation from 25 °C to 100 °C resulted in no significant decrease in emission intensity, while emission band shapes remained almost unchanged (Figures S78 and S79).

The exceptional thermal stability of fluorescence in PVP-dye systems can be attributed to several factors. The rigid PVP matrix structure restricts molecular motion and vibrational processes that typically lead to non-radiative deactivation of excited states at elevated temperatures. Additionally, specific dye–matrix interactions, such as hydrogen bonding between hydrazone functional groups and PVP carbonyl groups, may further stabilize the fluorophore conformation and protect against thermal quenching. This thermal robustness represents a significant advantage for applications requiring stable fluorescence performance under varying temperature conditions.

## 3. Materials and Methods

### 3.1. Synthesis of Phenylhydrazones

Phenylhydrazones were prepared by reacting equimolar amounts (1 mmol) of 4-cyanophenylhydrazine hydrochloride with various aldehydes and a ketone (Scheme 1). The aldehydes used included 4-methoxybenzaldehyde, 4-ethoxybenzaldehyde, 4-dimethylaminobenzaldehyde, salicylaldehyde, 4-hydroxy-3-methoxybenzaldehyde, 2,4-dichlorobenzaldehyde, 4-nitrobenzaldehyde, 4-(4-morpholinyl)benzaldehyde, 2-thiophenecarboxaldehyde, 5-nitro-2-thiophenecarboxaldehyde, 5-nitro-2-furaldehyde, 4-bromoacetophenone, *trans*-cinnamaldehyde, and *trans*-4-nitrocinnamaldehyde. The reactants were combined in 80 mL of methanol and heated at 65 °C for 1 h under constant stirring. The resulting crystalline products were isolated by allowing the solvent to gradually evaporate at ambient temperature.

### 3.2. Preparation of Electrospinning Solutions

A 10% (*w*/*v*) solution of PVP was prepared by dissolving the polymer in ethanol, chosen for its excellent solubility and optimal rheological properties at this concentration. For PS, a 30% (*w*/*v*) solution was prepared in a 9:1 (*v*/*v*) mixture of chloroform and dimethyl sulfoxide (DMSO). The higher concentration and DMSO addition were crucial to achieve sufficient viscosity for stable electrospinning while moderating the high vapor pressure of chloroform to ensure process stability.

To create hydrazone-doped polymer matrices, dyes **H3** and **H13** were added to the polymer solutions to achieve final concentrations of 1% and 5% (*w*/*w*) relative to the polymer mass. The mixtures were magnetically stirred until fully homogeneous. Control samples of pure PVP and PS were prepared under identical conditions without dyes. Notably, the 5% dye concentration was successfully achieved only for PVP matrices; for PS, the solution became excessively viscous, resulting in irregular fiber formation (Figure S80).

### 3.3. Electrospinning Process

**Figure 7:**
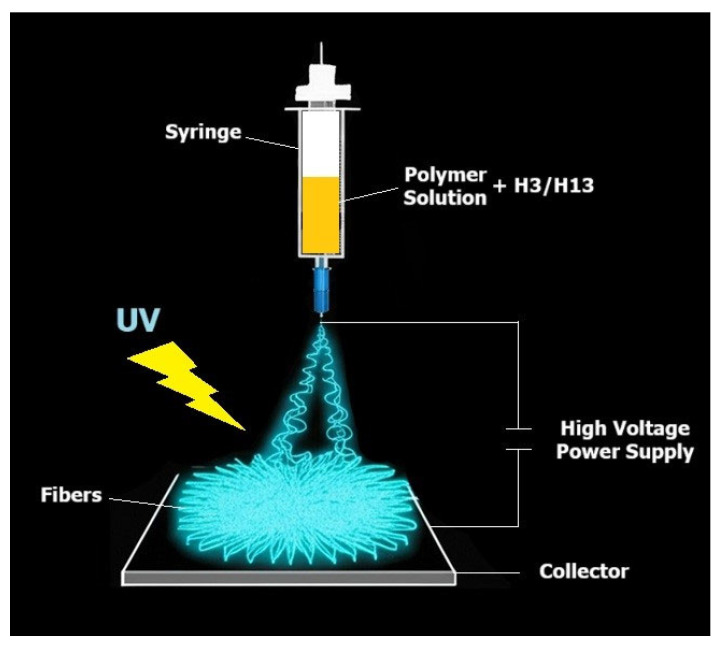
Electrospinning setup.

Electrospinning was performed at ambient temperature using a vertical setup (Figure 7). The polymer solution was loaded into a 3 mL syringe equipped with a metal needle (1.2 mm inner diameter), with solution delivery driven by gravity. For PVP-based matrices, a voltage of 25 kV was applied; for PS-based matrices, 15 kV was sufficient. These voltages were optimized to account for differences in electrical conductivity and rheology: higher voltage was required for the less conductive ethanol-based PVP solutions, while lower voltage sufficed for the more conductive chloroform/DMSO-based PS solutions. The needle was positioned 20 cm from a grounded flat collector covered with aluminum foil. The resulting fibrous mats were dried under ambient conditions for at least 24 h prior to characterization.

### 3.4. Instrumental Studies

#### 3.4.1. Single-Crystal X-Ray Diffraction (SC-XRD) Studies

The well-quality crystals of phenylhydrazones (**H1**–**H14**) were chosen and the SC-XRD measurements were carried out on an XtaLAB Synergy Dualflex Pilatus 300K diffractometer (Rigaku Corporation, Tokyo, Japan) at 100.0(1) K. The structures were solved and refined using SHELXT [45] and SHELXL [46], respectively. All non-hydrogen atoms were refined anisotropically. The N- and O-bound hydrogen atoms were located on the difference Fourier map and refined freely. The remaining hydrogen atoms were positioned geometrically and refined using the riding model. Details regarding crystal data and structural refinement can be found in Table S1. Supplementary crystallographic information for this study is available under CCDC 2476850-2476863.

#### 3.4.2. FTIR and ATR Spectroscopy

FTIR spectra for the **H1**–**H14** powders were recorded on a JASCO FT/IR-6200 spectrophotometer (Jasco, Easton, MD, USA) within the 4000–400 cm^−1^ range (Figures S81–S94). The samples were prepared as KBr pellets, in which the mass ratio of the analyzed substance to KBr was 1:100.

ATR spectra for the reference polymer materials and the dye-doped matrices were performed using a Nicolet iS50 spectrometer (Thermo Fisher Scientific, Madison, WI, USA) (Figures S45–S48). The analysis was conducted in the 4000–400 cm^−1^ measurement range with a resolution of 4.0 cm^−1^. The number of scans for both the baseline and spectrum collection was 32 and a DTGS ATR detector (Thermo Fisher Scientific, Madison, WI, USA) equipped with a diamond crystal was used. The accuracy of the wavenumber readout for the characteristic bands was ±1 cm^−1^.

#### 3.4.3. NMR

NMR spectra (Figures S95–S108) were acquired on a Bruker AVANCE DPX instrument (Bruker BioSpin GmbH, Rheinstetten, Germany), running at 250 MHz. Chemical shifts (δ) are reported in ppm relative to residual proton signals of (CD_3_)_2_S=O).

#### 3.4.4. DSC

DSC thermograms were recorded to determine the melting or disintegration temperature using a NETZSCH DSC 200 F3 Maia calorimeter (Netzsch-Geratebau GmbH, Selb, Germany). Samples were placed in closed aluminum crucibles. The measurements were conducted in an N2 atmosphere over a temperature range of 20–300 °C, with a heating/cooling rate of 20 °C/min.

- Results of FTIR, NMR and DSC measurements:

**(*E*)-4-(2-(4-methoxybenzylidene)hydrazineyl)benzonitrile** (**H1**): Yield 73%. IR (KBr, cm^−1^) 3265 (νN-H); 3065, 2970, 2931, 2836 (νCring-H); 2210 (νC≡N), 1604 (νC=N); 1535, 1510 (νC-Cring); 1463, 1428 (νC-Cring), (δC-H); 1327 (νC-N), (δC-H); 1298 (νasymC-O-C), (νCring –O); 1278 (νC-H3); 1255, 1164 (δC-H); 1135 (νN-N), 1032 (νC(CH3)-O); 911, 833, 820 (νsymC-O-C); 775, 714, 650, 639 (δCring-H); 601, 574, 524 (δO-C=O), (δC-C-C). 1H NMR (DMSO-d6 δH) ppm: 10.75 (s, 1H, N-N-H), 7.88 (s, 1H, N=C-H), 7.62–7.54 (m, 4H, Ar-H), 7.08 (d, J = 5.25 Hz, 2H, Ar-H), 6.96–6.92 (m, 2H, Ar-H), 3.75 (s, 3H, CH3). DSC mp. 154 °C.

**(*E*)-4-(2-(4-ethoxybenzylidene)hydrazineyl)benzonitrile** (**H2**): Yield 89%. IR (KBr, cm^−1^) 3258 (νN-H); 3075, 3041 2987, 2969, 2927, 2882 (νCring-H); 2216 (νC≡N), 1607 (νC=N); 1537, 1508 (νC-Cring); 1477, 1423 (νC-Cring), (νC-H3); 1334 (νC-N), (δC-H); 1298 (νasymC-O-C), (νCring –O); 1273 (νC-H3), 1244, 1175 (δC-H); 1132 (νN-N), 1045 (νC(CH3)-O); 911, 837 (νsymC-O-C); 799, 714, 661, 616 (δCring-H); 549, 525 (δO-C=O), (δC-C-C). 1H NMR (DMSO-d6 δH) ppm: 10.75 (s, 1H, N-N-H), 7.88 (s, 1H, N=C-H), 7.59–7.54 (m, 4H, Ar-H), 7.07 (d, J = 5.25 Hz, 2H, Ar-H), 6.92 (d, J = 5.5 Hz, 2H, Ar-H), 4.05–3.99 (m, 2H, CH2), 1.31–1.28 (m, 3H, CH3). DSC mp. 186 °C.

**(*E*)-4-(2-(4-(dimethylamino)benzylidene)hydrazineyl)benzonitrile** (**H3**): Yield 96%. IR (KBr, cm^−1^) 3298 (νN-H); 2995, 2899, 2837, 2804 (νCring-H); 2212 (νC≡N); 1611 (νC=N); 1510 (νC-Cring); 1440 (νC-Cring), (δC-H); 1367, 1336, 1270 (νC-N), (δC-H); 1171 (δC-H); 1123 (νN-N); 835, 813 (δCring-H), 545, 527 (δC-C-C), (δC-N-C). 1H NMR (DMSO-d6 δH) ppm: 10.59 (s, 1H, N-N-H), 7.82 (s, 1H, N=C-H), 7.53 (d, J = 5.75 Hz, 2H, Ar-H), 7.47 (d, J = 5.5 Hz, 2H, Ar-H), 7.03 (d, J = 5.5 Hz, 2H, Ar-H), 6.69 (d, J = 5.5 Hz, 2H, Ar-H), 2.91 (s, 6H, N(CH3)2). DSC mp. 219 °C.

**(*E*)-4-(2-(2-hydroxybenzylidene)hydrazineyl)benzonitrile** (**H4**): Yield 95%. IR (KBr, cm^−1^) 3420 (νO-H) 3262 (νN-H); 3154, 3047 (νCring-H); 2211 (νC≡N), 1608 (νC=N); 1569, 1536, 1503 (νC-Cring); 1489, 1470 (νC-Cring), (δC-H); 1413 (νO-H); 1364, 1330, 1309 (νC-N), (δC-H); 1274 (νC-O) 1216, 1200 (δC-H); 1170 (νN-N); 822, 815 (δCring-H), 543 (δC-C-C). 1H NMR (DMSO-d6 δH) ppm: 10.91 (s, 1H, OH), 10.12 (s, 1H, N-N-H), 8.23 (s, 1H, N=C-H), 7.65–7.63 (m, 1H, Ar-H), 7.59 (d, 2H, J = 5.5 Hz, Ar-H), 7.16–7.14 (m, 1H, Ar-H), 7.04 (d, 2H, J = 5.25 Hz, Ar-H), 6.86–6.83 (m, 2H, Ar-H). DSC mp. 228 °C.

**(*E*)-4-(2-(4-hydroxy-3-methoxybenzylidene)hydrazineyl)benzonitrile** (**H5**): Yield 88%. IR (KBr, cm^−1^) 3318 (νO-H), (νN-H); 2994, 2947, 2850 (νCring-H); 2225 (νC≡N), 1602 (νC=N); 1512 (νC-Cring); 1462 (νC-Cring), (δC-H); 1424 (νO-H); 1353, 1313 (νC-N), (δC-H); 1282 (νC-O) 1242, 1207 (δC-H); 1165 (νN-N); 1033 (νC(CH3)-O); 929, 852 (νsymC-O-C), (δCring-H); 837, 813, 777 (δCring-H), 668 (δO-C=O), 548 (δC-C-C). 1H NMR (DMSO-d6 δH) ppm: 10.70 (s, 1H, OH), 9.31 (s, 1H, N-N-H), 7.83 (s, 1H, N=C-H), 7.55 (d, 2H, J = 5.5 Hz, Ar-H), 7.26–7.25 (m, 1H, Ar-H), 7.07 (d, 2H, J = 5.5 Hz, Ar-H), 7.03–7.01 (m, 1H, Ar-H), 6.76 (d, 1H, J = 5.25 Hz, Ar-H), 3. 80 (s, 3H, CH3). DSC mp. 242 °C.

(***E*)-4-(2-(2,4-dichlorobenzylidene)hydrazineyl)benzonitrile** (**H6**): Yield 78%. IR (KBr, cm^−1^) 3262 (νN-H); 3177, 3116, 3093, 3056 (νCring-H); 2220 (νC≡N), 1608 (νC=N); 1589, 1558, 1545, 1504 (νC-Cring); 1469, 1424 (νC-Cring), (δC-H); 1385, 1361 (δC-H); 1321 (νC-N), (δC-H); 1279, 1255, (δC-H); 1167 (νN-N); 1095, 1045, 905, 858 (δC-H), 825 (νC-Cl); 543 (δC-C-C). 1H NMR (DMSO-d6 δH) ppm: 11.27 (s, 1H, N-N-H), 8.21 (s, 1H, N=C-H), 8.01 (d, J = 5.25 Hz, 1H, Ar-H), 7.62 (m, 3H, Ar-H), 7.42 (m, 1H, Ar-H), 7.14 (d, J = 5.5 Hz, 2H, Ar-H). DSC mp. 236 °C.

**(*E*)-4-(2-((4-nitrobenzylidene)hydrazineyl)benzonitrile** (**H7**): Yield 95%. IR (KBr, cm^−1^) 3294 (νN-H); 3111 (νCring-H); 2210 (νC≡N); 1609 (νC=N); 1565 (νC-Cring); 1534 (νNO2); 1498 (νC-Cring); 1423, 1423 (νC-Cring), (δC-H); 1336 (νC-N), (δC-H), (νNO2); 1293, 1272 (δC-H); 1150 (νN-N); 1110, 917 (δC-H); 856, 827, 750, 695 (δCring-H); 587, 543 (δC-C-C). 1H NMR (DMSO-d6 δH) ppm: 11.32 (s, 1H, N-N-H), 8.21 (m, 2H, Ar-H), 8.01 (s, 1H, N=C-H), 7.91 (m, 2H, Ar-H), 7.64 (d, J = 5.5 Hz, 2H, Ar-H), 7.20 (d, J = 5.5 Hz, 2H, Ar-H). DSC mp. 221 °C.

**(*E*)-4-(2-(4-morpholinobenzylidene)hydrazineyl)benzonitrile** (**H8**): Yield 75%. IR (KBr, cm^−1^) 3258 (νN-H); 2960, 2858 (νCring-H); 2215 (νC≡N), 1607 (νC=N); 1537, 1513 (νC-Cring); 1447, 1424 (νC-Cring), (δC-H); 1381, 1342 (νC-N), (δC-H); 1275, 1265, 1228 (δC-H); 1168 (νN-N); 927 (δC-O-C); 826, 815 (δCring-H), 544 (δC-C-C). 1H NMR (DMSO-d6 δH) ppm: 10.69 (s, 1H, N-N-H), 7.84 (s, 1H, N=C-H), 7.55–7.51 (m, 4H, Ar-H), 7.06 (d, 2H, J = 5.5Hz, Ar-H), 6.93 (d, 2H, J = 5.5Hz, Ar-H), 3.71–3.69 (m, 4H, O-morpholinyl-H), 3.15–3.12 (m, 4H, N-morpholinyl-H). DSC mp. 248 °C.

**(*E*)-4-(2-(thiophen-2-ylmethylene)hydrazineyl)benzonitrile** (**H9**): Yield 75%. IR (KBr, cm^−1^) 3263 (νN-H); 3159, 3120, 3068 (νCring-H); 2212 (νC≡N), 1603 (νC=N); 1581, 1538, 1515 (νC-Cring); 1499, 1436 (νC-Cring), (δC-H); 1363, 1339, 1307 (νC-N), (δC-H); 1277, 1236, 1216 (δC-H); 1165 (νN-N); 1039, 909 (δC-H); 834, 704 (δCring-H), (νC-S); 546 (δC-C-C). 1H NMR (DMSO-d6 δH) ppm: 10.88 (s, 1H, N-N-H), 8.13 (s, 1H, N=C-H), 7.58 (m, 2H, Ar-H), 7.51 (m, 1H, thiofene-H), 7.29 (m, 2H, Ar-H), 7.04 (m, 2H, thiofene-H). DSC mp. 164 °C.

**(*E*)-4-(2-((5-nitrothiophen-2yl)methylene)hydrazineyl)benzonitrile** (**H10**): Yield 63%. IR (KBr, cm^−1^) 3259 (νN-H); 3177, 3094, 2947 (νCring-H); 2210 (νC≡N); 1608 (νC=N); 1560 (νC-Cring); 1523 (νNO2); 1505 (νC-Cring); 1489, 1438, 1420 (νC-Cring), (δC-H); 1363 (νC-N), (δC-H); 1327 (νNO2); 1306 (νC-N), (δC-H); 1294, 1278, 1226 (δC-H); 1171 (νN-N); 1034, 884 (δC-H); 838, 813, 793 (δCring-H), (νC-S); 587, 544 (δC-C-C). 1H NMR (DMSO-d6 δH) ppm: 11.47 (s, 1H, N-N-H), 8.10 (s, 1H, N=C-H), 8.05 (d, J = 2.75 Hz, 1H, Ar-H), 7.65 (d, J = 5.5 Hz, 2H, Ar-H), 7.36 (d, J = 2.75 Hz, 1H, Ar-H), 7.14 (m, 2H, Ar-H). DSC disintegration 276 °C.

**(*E*)-4-(2-((5-nitrofuran-2-yl)methylene)hydrazineyl)benzonitrile** (**H11**): Yield 80%. IR (KBr, cm^−1^) 3312 (νN-H); 3148 (νCring-H); 2208 (νC≡N); 1607 (νC=N); 1569 (νC-Cring); 1544 (νNO2); 1505 (νC-Cring); 1468, 1421 (νC-Cring), (δC-H); 1360 (νC-N), (δC-H); 1334 (νNO2); 1313 (νC-N), (δC-H); 1275, 1250, 1198 (δC-H); 1168 (νN-N); 1019 (νC-O-C); 895, 881, 830, 813, 793 (δC-H); 618, 547 (δC-C-C). 1H NMR (DMSO-d6 δH) ppm: 11.50 (s, 1H, N-N-H), 7.86 (s, 1H, N=C-H), 7.75 (d, J = 2.5 Hz, 1H, NO2-furan -H), 7.66 (d, J = 5.5 Hz, 2H, Ar-H), 7.17 (m, 2H, ArH), 7.1 (d, J = 2.5 Hz, 1H, NO2-furan -H). DSC disintegration 248 °C.

**(*E*)-4-(2-(1-(4-bromophenyl)ethylidene)hydrazineyl)benzonitrile** (**H12**): Yield 90%. IR (KBr, cm^−1^) 3302 (νN-H); 3088, 3054 (νCring-H); 2210 (νC≡N), 1607 (νC=N); 1519 (νC-Cring); 1483 (νC-Cring), (δC-H); 1394, 1336 (νC-N), (δC-H); 1271, 1168, (δC-H); 1144 (νN-N); 835, 815 (δCring-H), 545 (δC-C-C). 1H NMR (DMSO-d6 δH) ppm: 9.91 (s, 1H, N-N-H), 7.74–7.71 (m, 2H, Ar-H), 7.60–7.53 (m, 4H, Ar-H), (d, J = 5.5 Hz, 2H, Ar-H), 2.25 (s, 3H, CH3). DSC mp. 219 °C.

**4-(2-((1*E*,2*E*)-3-phenylallylidene)hydrazineyl)benzonitrile** (**H13**): Yield 69%. IR (KBr, cm^−1^) 3256 (νN-H); 3077, 3058 3028, 3001 (νCring-H); 2211 (νC≡N), 1602 (νC=N); 1563, 1531 (νC-Cring); 1495, 1446, 1426 (νC-Cring), (δC-H); 1356 (νC-N), (δC-H); 1319, 1329, 1305, 1295, 1269 (δC-H); 1173 (νN-N); 959 (νC=C); 838, 838, 746, 688 (δCring-H), 549 (δC-C-C). 1H NMR (DMSO-d6 δH) ppm: 10.84 (s, 1H, N-N-H), 7.77 (d, J = 5.5 Hz, 1H, N=C-H), 7.55 (m, 4H, Ar-H), 7.33 (t, J = 4.75 Hz, 2H, Ar-H), 7.25 (t, J = 4.5 Hz, 1H, Ar-H), 7.03 (d, J = 5.5 Hz, 2H, Ar-H), 6.98 (m, 1H, C=C-H). 6.90 (m, 1H, C=C-H). DSC mp. 162 °C.

**4-(2-((1*E*,2*E*)-3-(4-nitrophenyl)allylidene)hydrazineyl)benzonitrile** (**H14**): Yield 81%. IR (KBr, cm^−1^) 3269 (νN-H); 3065 (νCring-H); 2211 (νC≡N); 1602 (νC=N); 1555 (νC-Cring); 1535 (νNO2); 1511 (νC-Cring); 1457, 1429 (νC-Cring), (δC-H); 1375 (νC-N), (δC-H), 1336 (νNO2); 1273, 1256, 1208 (δC-H); 1141 (νN-N); 1103 (δC-H); 965 (νC=C); 871, 841, 824, 748 (δCring-H); 564, 547 (δC-C-C). 1H NMR (DMSO-d6 δH) ppm: 11.06 (s, 1H, N-N-H), 8.16 (d, J = 5.5 Hz, 2H, Ar-H), 7.80 (m, 3H, N=C-H, Ar-H), 7.59 (d, J = 5.5 Hz, 2H, Ar-H), 7.23 (m, 1H, C=C-H), 7.06 (d, J = 5.5 Hz, 2H, Ar-H), 7.01 (m, 1H, C=C-H). DSC mp. 280 °C.

#### 3.4.5. UV-VIS Spectroscopy

The UV-Vis diffuse reflectance spectra (Figures S16–S29) were recorded on a Jasco V-660 spectrometer (Jasco, Easton, MD, USA), in the spectral range 200–800 nm, using spectralon as a standard with 100% reflectance.

#### 3.4.6. Fluorescence Spectroscopy

Initial characterization was performed using a JASCO FP-6500 spectrophotometer (Jasco, Easton, MD, USA). The solid-state three-dimensional excitation-emission matrix (3D-EEM) spectra were recorded for all samples, including the pure hydrazone powders, the reference polymer materials, and the 1% dye-doped polymer matrices. This initial screening was performed in a very broad wavelength range (excitation λ_ex_ 220–640 nm; emission λ_em_ 220–745 nm) with the primary objective of mapping the complete photoluminescent “fingerprint” of each material and identifying the optimal spectral regions for more detailed subsequent investigations.

The absolute fluorescence quantum yields were measured on an Edinburgh Instruments FLS920 spectrofluorometer (Edinburgh Instruments Ltd., Livingston, UK) using an integrating sphere with spectralon as a reference blank. Each compound was excited at its respective absorption maximum to ensure the highest possible signal-to-noise ratio, a critical factor for the accurate determination of fluorescence efficiency.

The fluorescence lifetime measurements on the solid-state powder samples were performed using the Time-Correlated Single Photon Counting (TCSPC) technique on an Edinburgh Instruments FLS920 spectrofluorometer (Figures S109–S117). The system was equipped with a pulsed picosecond diode laser as the excitation source and a high-speed photomultiplier tube (PMT) detector. For each measurement, the samples were excited at their absorption maximum, and the emission was monitored at the peak of their fluorescence band. The resulting fluorescence decay curves were analyzed by fitting them to a multi-exponential model using iterative reconvolution with the instrument response function (IRF). The quality of the fit for all reported lifetimes was validated by an χ^2^ value close to 1.0.

Detailed photophysical analysis of the dye-doped polymer matrices was conducted using an Edinburgh Instruments FS5 spectrofluorometer (Edinburgh Instruments Ltd., Livingston, UK):

1. Excitation and Emission Spectra: High-resolution excitation spectra were recorded over a focused range (250–450 nm) while monitoring the emission at the respective maxima identified in the initial screening (464 nm for **H3** and 485 nm for **H13** systems). Correspondingly, detailed emission spectra were recorded upon excitation at several distinct wavelengths (280 nm, 320 nm, 380 nm) to verify the principle of excitation-emission independence within the polymer host.
2. Quantum Yield of Doped Matrices: The absolute quantum yields of the dye-doped mats were also determined using the FS5 instrument’s integrating sphere. The excitation wavelengths were specifically selected to match the absorption maxima of the hydrazones once embedded within the polymer matrix (415 nm for **H3**-doped matrices and 405 nm for **H13**-doped matrices), which can differ slightly from the pure powder form.
3. Temperature-Dependent Studies: The thermal stability of the polymer mats was assessed using a temperature-controlled reflectance setup integrated with the FS5 spectrometer. For the analysis, the sample was positioned on a heating plate equipped with an adjacent thermocouple to monitor the surface temperature. A 460 nm LED was employed as a stable excitation source, and emission was recorded at 25 °C, 50 °C, and 100 °C. Each measurement was taken at the moment the thermocouple confirmed that the setpoint temperature had been reached.

#### 3.4.7. Scanning Electron Microscopy (SEM)

Fiber morphology was analyzed using a Thermo Scientific Phenom ProX G6 Desktop instrument (Thermo Fisher Scientific, Eindhoven, The Netherlands). Prior to imaging, all samples were sputtered with a nanometric gold layer using a Quorum Q150RS coater (Quorum Technologies Ltd., Laughton, UK) to ensure electrical conductivity and prevent charging effects. Key imaging parameters included: detection mode BSD, operating voltage 10 kV, and vacuum at 1 Pa. Magnification ranges were adapted to the sample type to account for the vast differences in fiber dimensions: broader magnifications (500× to 10,000×) were used for the thick PS fibers, while higher magnifications (1000× to 20,000×) were necessary to resolve the details of the much thinner PVP nanofibers.

### 3.5. Computational Methods

Excited states of the hydrazone series (**H1**–**H14**) were computed by time-dependent density functional theory (TD-DFT) using cluster models derived from single-crystal X-ray diffraction geometries. Initial molecular assemblies were constructed in Mercury (v2022.3.0) [63] by extracting nearest-neighbor motifs that capture the dominant supramolecular contacts (hydrogen bonding, offset π···π and CH···O/N interactions) identified by the program’s empirical contact analysis. To standardize the crystallographic models while preserving packing, hydrogen atoms were shifted along their bond vectors to match average X–H distances from neutron diffraction data; no further geometry optimization was performed. All TD-DFT calculations were carried out in Gaussian 09 (rev. E.01) [64] using the B3LYP functional [65] in combination with the 6–31G (2d,2p) basis set [66,67], and vertical excitations were evaluated at the adjusted X-ray coordinates. For each cluster, 700 singlet excitations were calculated to ensure coverage of the experimental absorption and emission ranges.

In line with the multi-molecule protocol established in our previous work [52], the number of molecules per cluster was chosen to reflect the strength and topology of local packing interactions for each compound. Three-molecule clusters were used for **H8** and **H13**; four-molecule clusters were employed for **H1**, **H2**, **H4**–**H7**, **H9**–**H12**, and **H14**; and a five-molecule cluster was used for **H3**. All models were treated as neutral singlets and evaluated in the gas phase (no continuum) so as to isolate the influence of specific supramolecular contacts present in the solid state. A correlation between theory and experiment was established by comparing excitation energies and oscillator strengths with the positions and relative intensities of the measured absorption bands and fluorescence maxima. The electronic character of the dominant transitions was assigned from molecular-orbital contribution analysis and orbital contour plots generated with Chemissian (v4.67) [68].

## 4. Conclusions

This work demonstrates a multiscale strategy for designing emissive materials, starting from the molecular level with substituent engineering and extending to supramolecular control within electrospun polymer matrices. By systematically studying a series of fourteen 4-cyanophenylhydrazones, this research establishes practical design rules for achieving solid-state fluorescence with emission maxima that can be precisely tuned across the blue spectral region (421–494 nm). Donor substitution combined with packing that avoids classical ring-stacking yields the strongest emitters, whereas hydroxy-substituted members remain emissive but weak due to fast non-radiative channels. Nitro-substituted compounds and the thiophene analogue are non-emissive, consistent with low-lying charge-transfer states and/or accelerated intersystem crossing. The trends are quantitatively supported by rate constants extracted from quantum yields and lifetimes, with efficient systems characterized by sizable k_r_ and suppressed low-frequency motions, and weak systems dominated by k_nr_.

The study further reveals that the polymer matrix is not a passive host but an active component that can be used to engineer both photophysical and material properties. Embedding the fluorophores induced significant blue shifts through a combination of disaggregation and environmental effects. A key finding is the mechanistic specificity dependent on the dye’s structure. While both **H3** and **H13** are efficient emitters, only **H13** induced a functional change in the host material, transforming the water-soluble PVP into a stable, water-resistant material. This stabilization is attributed to a pseudo-crosslinking effect, where dye–polymer interactions establish a supramolecular network that restricts polymer chain mobility and modifies the local polymer environment. This highlights a principle where the molecular design of the guest dye can directly influence the macroscopic properties of the final material.

Furthermore, a notable relationship between dye concentration and the material’s properties was identified. Contrary to typical aggregation-caused quenching, increasing the dye loading from 1 wt% to 5 wt% in PVP led to an enhancement of the fluorescence quantum yield. This indicates that at higher concentrations, the Restriction of Intramolecular Motion (RIM) becomes a more influential factor, effectively suppressing non-radiative decay pathways. This showcases that dye loading can be used as a simple handle to switch between optimizing for spectral shifts (at low concentrations) or for high fluorescence efficiency (at high concentrations).

Positioning these findings in the broader context of blue-emitting materials highlights their practical significance. A comparison with other prominent material classes reveals a landscape of distinct trade-offs. For instance, metal-free carbon dots (CDs), while sustainable, typically exhibit moderate quantum yields in the blue region (often 15–25%) and their solid-state performance can be limited by environmental sensitivity [69,70]. In the realm of metal complexes, high-performance phosphorescent emitters based on noble metals like Ir(III) can achieve exceptional efficiencies (Φ > 80%), but their reliance on rare and costly materials poses significant sustainability challenges [71]. More accessible first-row transition metal complexes, such as those based on Cu(I) or Zn(II), often present their own limitations, including either modest quantum yields (frequently below 20%) or poor operational stability, with their emission being highly susceptible to quenching when incorporated into polymer matrices [72,73]. In this context, the 4-cyanophenylhydrazones reported herein offer a compelling balance of advantages. They combine a facile, metal-free synthesis with efficient and tunable solid-state fluorescence, achieving quantum yields up to 40% in the blue-to-cyan region. Critically, their exceptional thermal stability when embedded in electrospun PVP fibers—maintaining robust emission up to 100 °C—directly addresses a key challenge for many organic and organometallic fluorophores.

Collectively, substituent pattern, packing control, host identity, and dye loading emerge as accessible levers for engineering blue emission that is tunable, efficient, and operationally robust in phenylhydrazone-based materials.

## Figures and Tables

**Table 1:** Intermolecular interactions in the crystal structures of **H1**–**H14**.

Compound	H-Bond Motifs or NH···π (Ring) Interactions	π···π Ring-Stacking Motifs (System)	π···π Stacking Between Hydrazone Moiety and Ring (System)	π···π Stacking Between Cyano group and Ring
**H1**	NH···NC, L_1_C(8)	–	hm···R_2_ (dimers)	CN···R_2_
**H2**	NH···NC, L_1_C(8)	–	–	CN···R_2_
**H3**	NH···R_2_	–	–	–
**H4**	NH···NC, L_2_C_2_^2^(16)[C(8)]	HT1 and HT2 (columnar) *	–	–
**H5**	NH···N, L_1_C(3) OH···NC, L_1_C(15)	HT2 (dimers)	–	–
**H6**	NH···NC, L_1_C(8)	HH2 (columnar)	–	–
**H7**	NH···NC, L_1_C(8) NH···ON, L_1_C(10)	–	hm···PhCN (columnar)	CN···PhCN
**H8**	NH···NC, L_4_C_4_^4^(32)[C(8)]	–	–	CN···PhCN
**H9**	NH···NC, L_1_C(8)	HH1 (columnar)	–	–
**H10**	NH···NC, L_1_C(8)	HH2 (columnar)	–	–
**H11**	NH···NC, L_1_C(8) NH···ON, L_1_R_2_^2^(9)	HT2 (columnar)	–	–
**H12**	NH···NC, L_1_C(8)	HH2 (columnar)	–	–
**H13**	NH···NC, L_1_C(8)	–	hm···R_2_ (dimers)	–
**H14**	NH···NC, L_1_C(8)	HT2 (dimers)	–	–

G_d_^a^(n)—hydrogen-bond pattern; L_1–4_—H-bond graph level, respectively; PhCN—benzene ring with cyano substituent, R_2_—second ring (see Section 3, Scheme 1); HT—head-to-tail; HH—head-to-head; hm—hydrazone moiety; *—two structurally different columnar systems exist within the crystal lattice.

**Table 2:** Fluorescence data for emissive phenylhydrazones in the solid state.

Compound	λ_ex_ (nm)	λ_em_ (nm)	Int	H → L Transition	Orbital Transition	Φ_f_	τ (ns)
**H1**	343	440	252	H-1 → L + 3 H → L + 4	π → π*	0.23	0.94
	419	440	436	H → L + 2	π → π*	0.11	
**H2**	338	425	227	H-1 → L + 2	π → π*	0.25	0.72
	407	425	407	H-1 → L	π → π* (LLCT)	0.20	
**H3**	362	462	249	H-1 → L + 6	π → π* (LLCT)	0.22	12.50
	438	462	359	H-1 → L	π → π* (LLCT)	0.10	
**H4**	372	434	17	H-2 → L + 2 H-3 → L	π → π*	0.02	0.45
**H5**	367	433	16	H → L + 4	π → π*	0.03	<0.2 ^#^
**H6**	357	463	101	H-3 → L	π → π* (LLCT)	0.07	1.18
	434	463	171	H-2 → L	π → π* (LLCT)	0.04	
**H8**	344	446	121	H → L + 2 H-2 → L	π → π*	0.05	0.58
	425	446	244	H → L	π → π* (LLCT)	0.03	
**H12**	366	421	43	H-1 → L + 2	π → π*	0.02	1.33
**H13**	351	494	135	H-1 → L + 3 H-3 → L + 1 H-2 → L	π → π*	0.40	5.14
	439	494	296	H → L + 1	π → π*	0.22	

^#^ The lifetime for this compound could not be accurately determined as the decay profile significantly overlaps with the Instrument Response Function (IRF). The actual lifetime may be shorter than the instrument’s temporal resolution.

## Data Availability

All data generated or analyzed during this study are included in this published article and its Supplementary Information files. The X-ray crystallographic data for structures reported in this study have been deposited with the Cambridge Crystallographic Data Centre (CCDC). These data can be obtained free of charge via https://www.ccdc.cam.ac.uk/structures/ (accessed on 3 September 2025). Supplementary crystallographic information for this study is available under CCDC 2476850-2476863.

## Supplementary Materials

The following supporting information can be downloaded at: https://www.mdpi.com/article/10.3390/molecules30173638/s1.
