# Supplementary material for: Fluorescence of 4-Cyanophenylhydrazones: From Molecular Design to Electrospun Polymer Fibers
Source: Molecules. 2025 Sep 6;30(17):3638. doi: 10.3390/molecules30173638 (PMC12430612; doi:10.3390/molecules30173638)
Supplement: Supplementary file 1 [file molecules-30-03638-s001.zip › molecules-3847970-supplementary materials.pdf]

Supplementary Information

Fluorescence of 4-Cyanophenylhydrazones: From Molecular Design to Electrospun Polymer Fibers

Paulina Sobczak-Tyluś [1,2,*], Tomasz Sierański [1], Marcin Świątkowski [1], Agata Trzęsowska-Kruszyńska [1] and Oskar Bogucki [3,4]

1 Institute of General and Ecological Chemistry, Lodz University of Technology, Zeromskiego 116, 90-924 Lodz, Poland; tomasz.sieranski@p.lodz.pl (T.S.); marcin.swiatkowski@p.lodz.pl (M.Ś.); agata.trzesowska-kruszynska@p.lodz.pl (A.T.-K.)

2 Łukasiewicz—Lodz Institute of Technology, M. Sklodowskiej-Curie 19/27, 90-570 Lodz, Poland

3 Łukasiewicz Research Network—Institute of Microelectronics and Photonics, Lotnikow 32/46, 02-668 Warsaw, Poland; oskar.bogucki@imif.lukasiewicz.gov.pl

4 Institute of Microelectronics and Optoelectronics, Warsaw University of Technology, Koszykowa 75, 00-662 Warsaw, Poland

* Correspondence: paulina.sobczak@dokt.p.lodz.pl

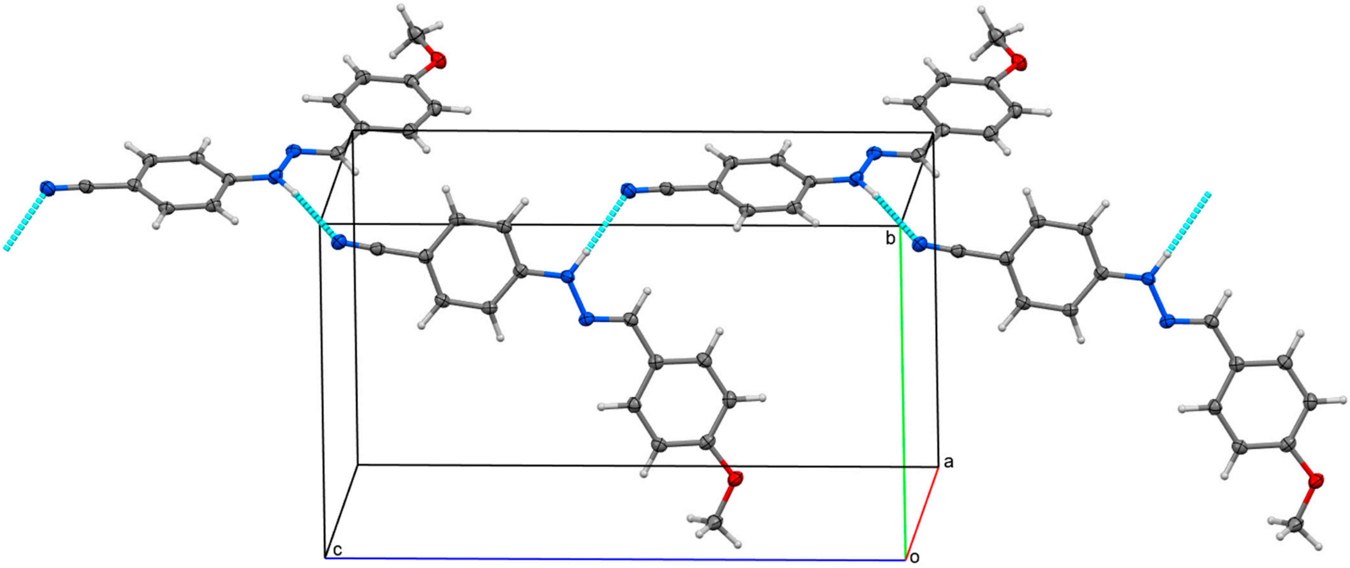

Figure S1. Hydrogen bond chain motif C(8) in the structure of **H1.**

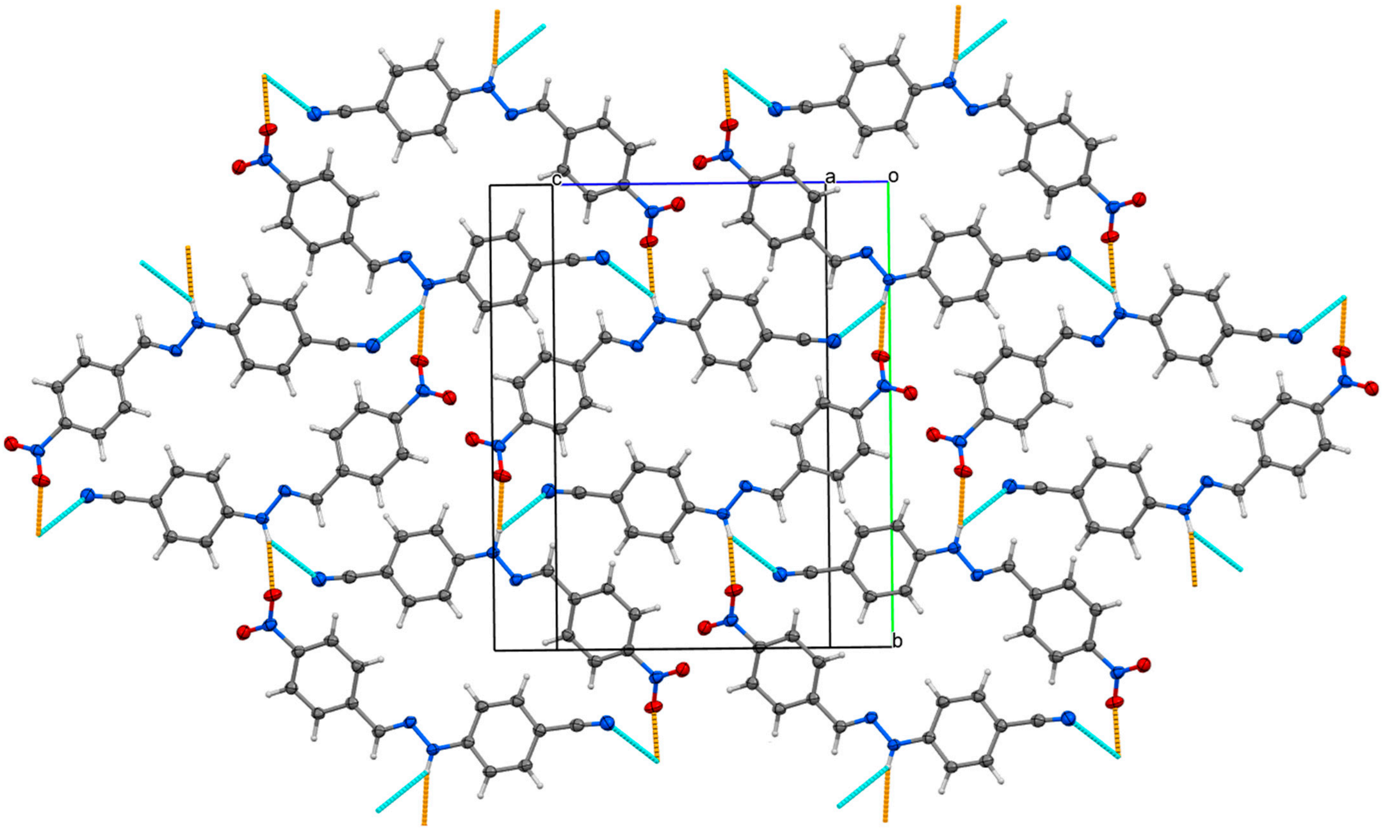

Figure S2. Hydrogen bond chain motifs, C(8) – cyan, C(10) – orange, in the structure of **H7.**

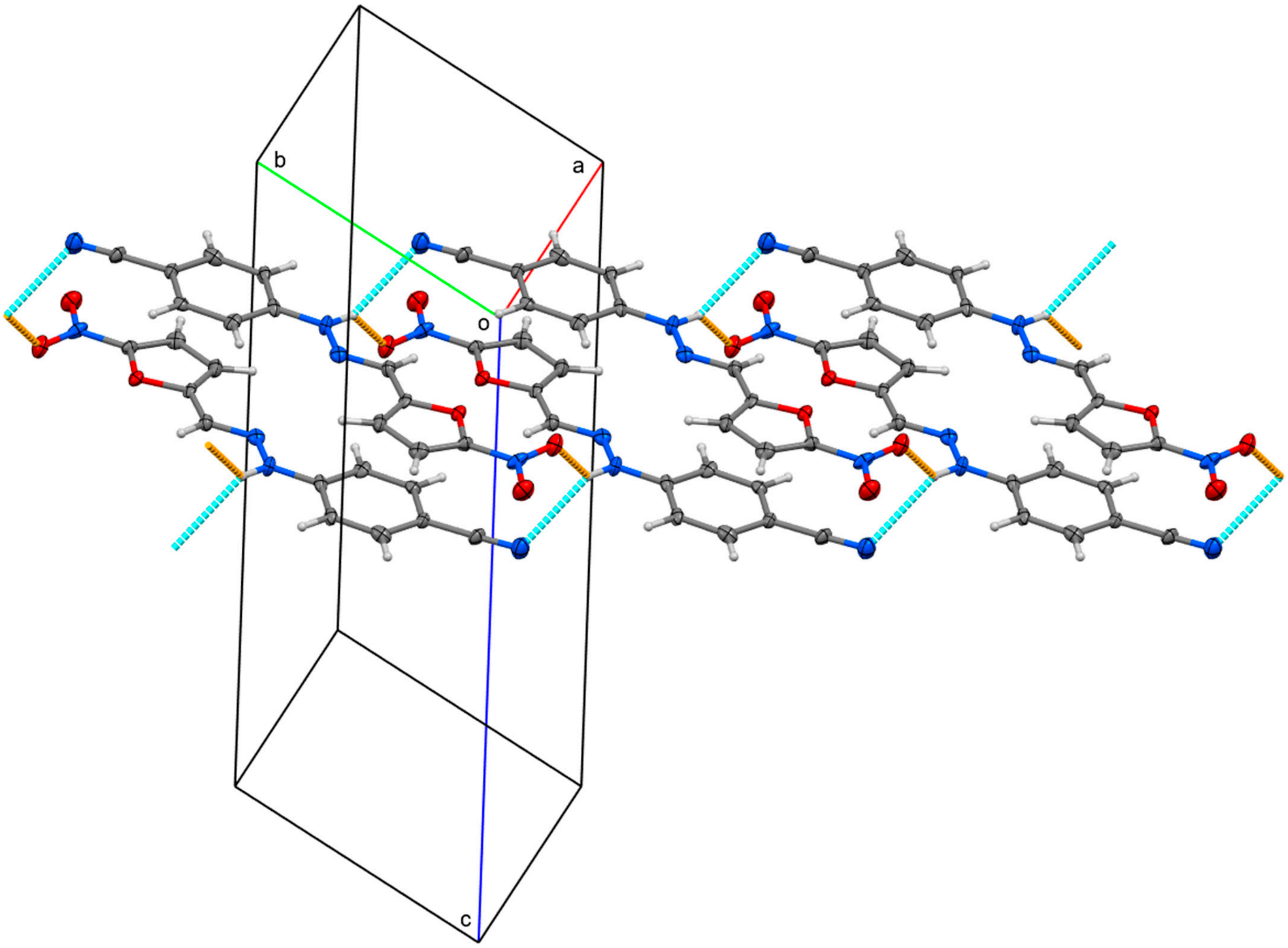

Figure S3. Hydrogen bond chain motifs, C(8) – cyan, $R_2^2(9)$ – orange, in the structure of **H11.**

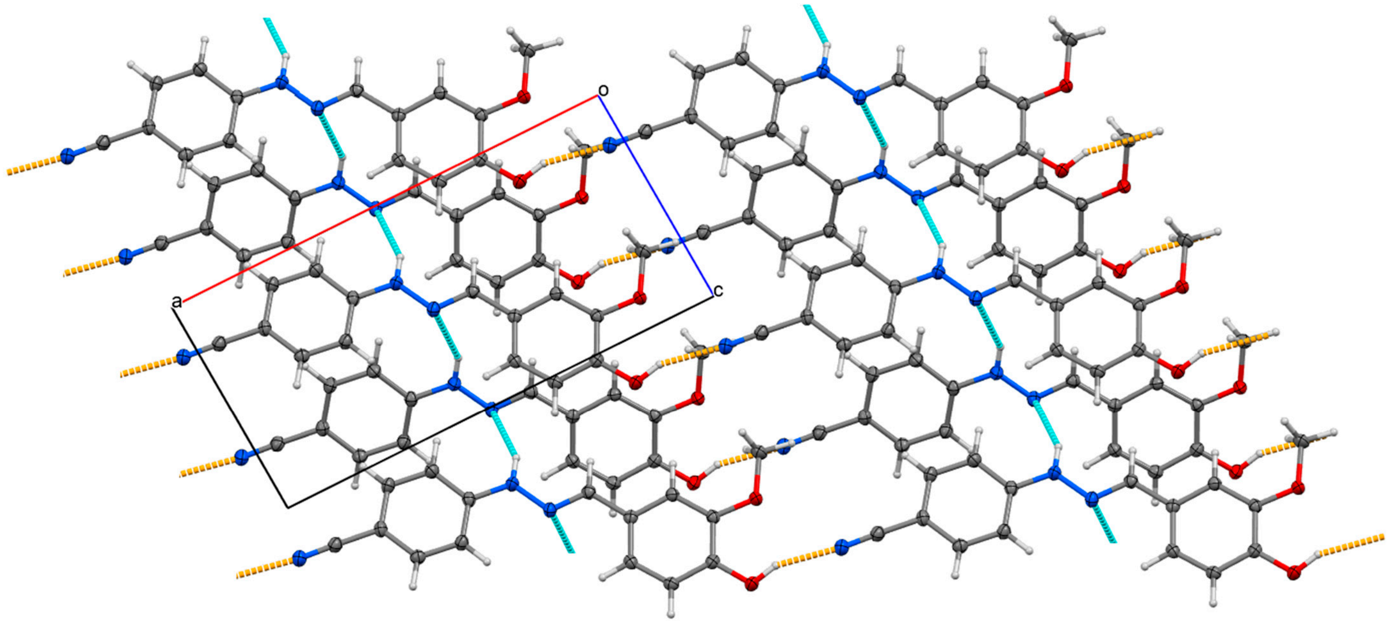

Figure S4. Hydrogen bond chain motifs, C(3) – cyan, C(15) – orange, in the structure of **H5.**

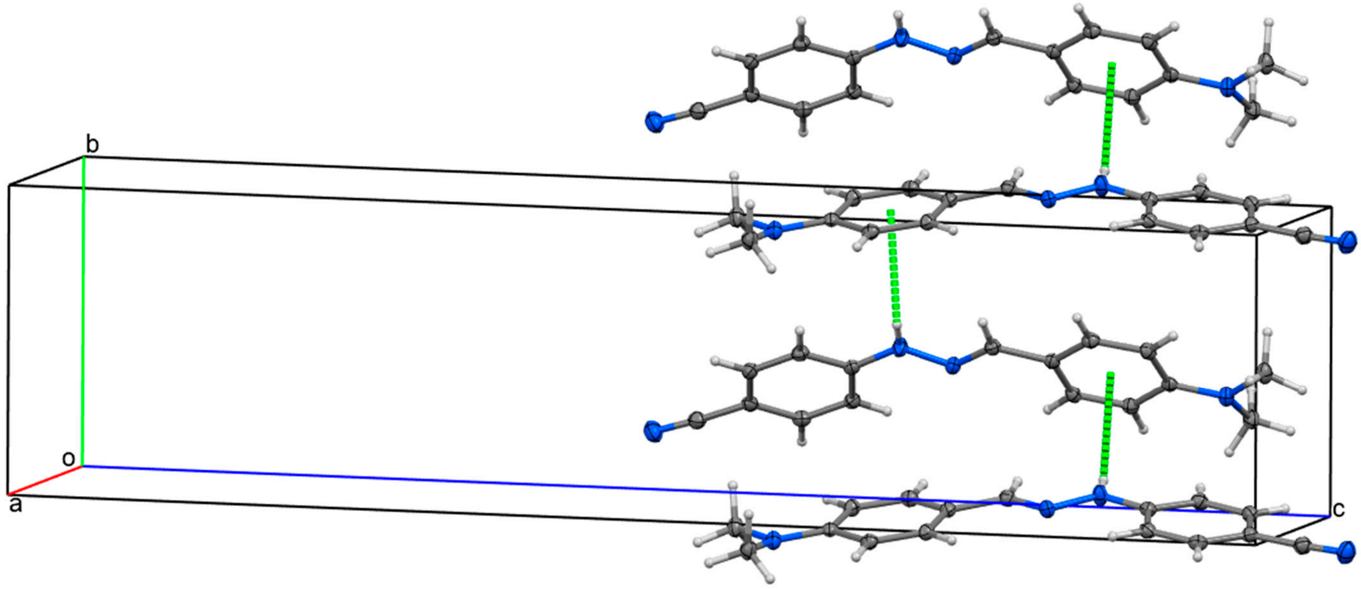

Figure S5. NH•••π interactions in the structure of **H3.**

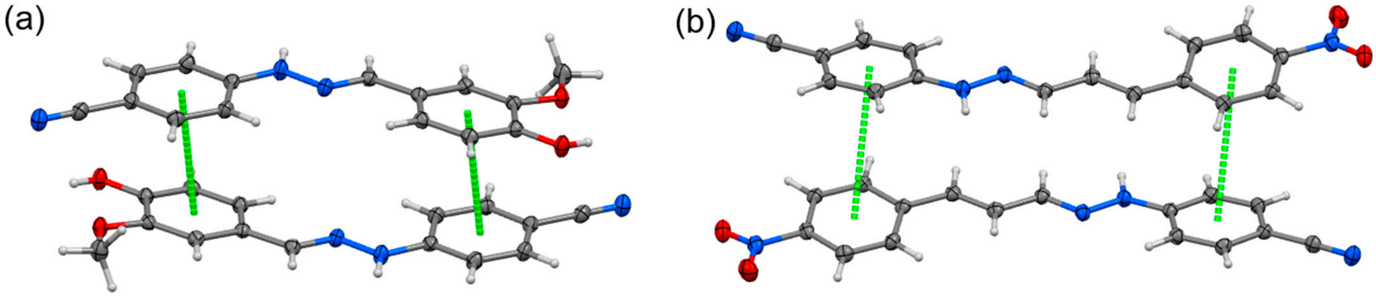

Figure S6. Supramolecular π•••π stacking dimers in the structures of **H5** (a) and **H14** (b), organized by HT2 motifs.

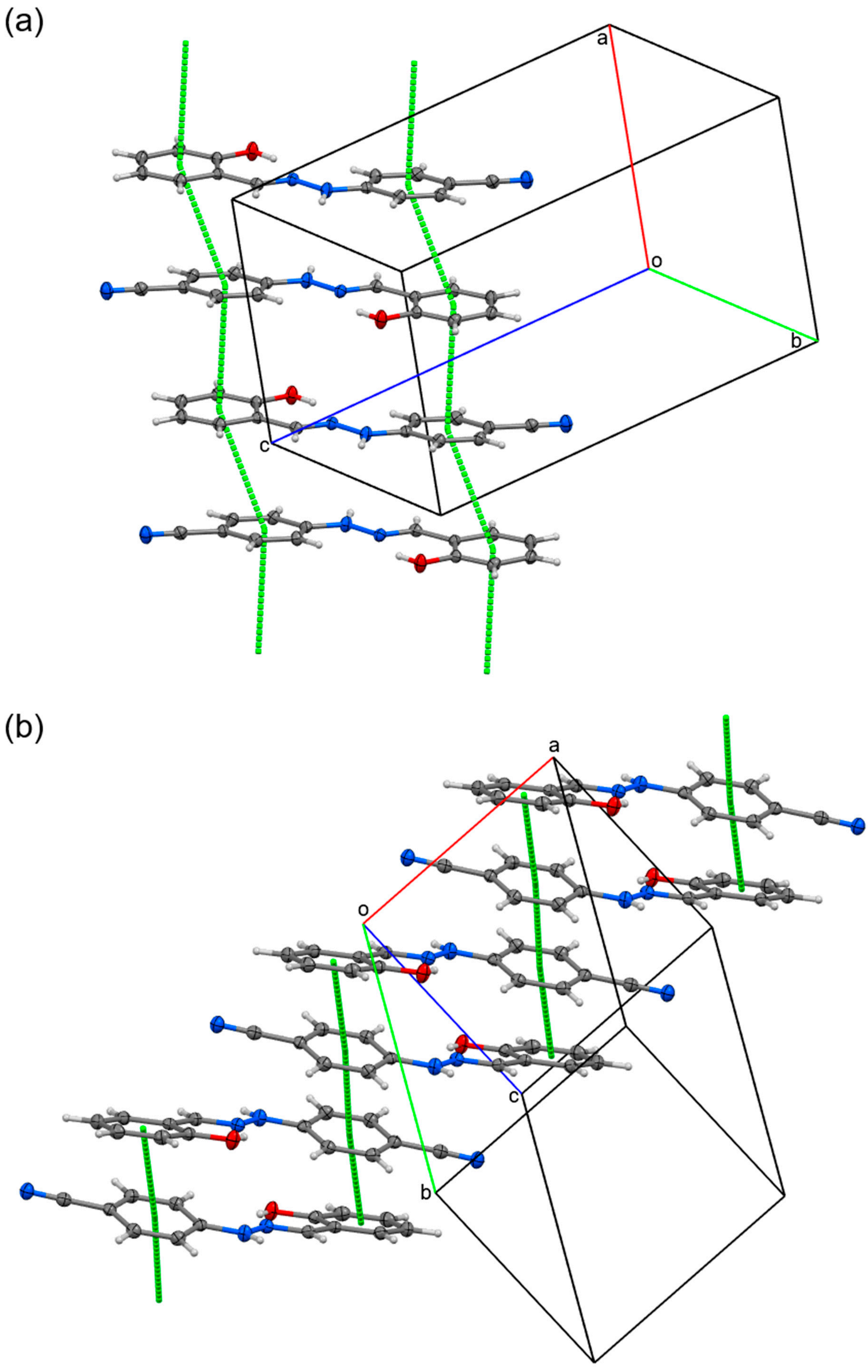

Figure S7. Suprmolecular π•••π stacking columnar systems in the structure of **H4** organized by different HT motifs. Columnar systems formed by molceules containg N1 atoms (a) and N21 atoms (b).

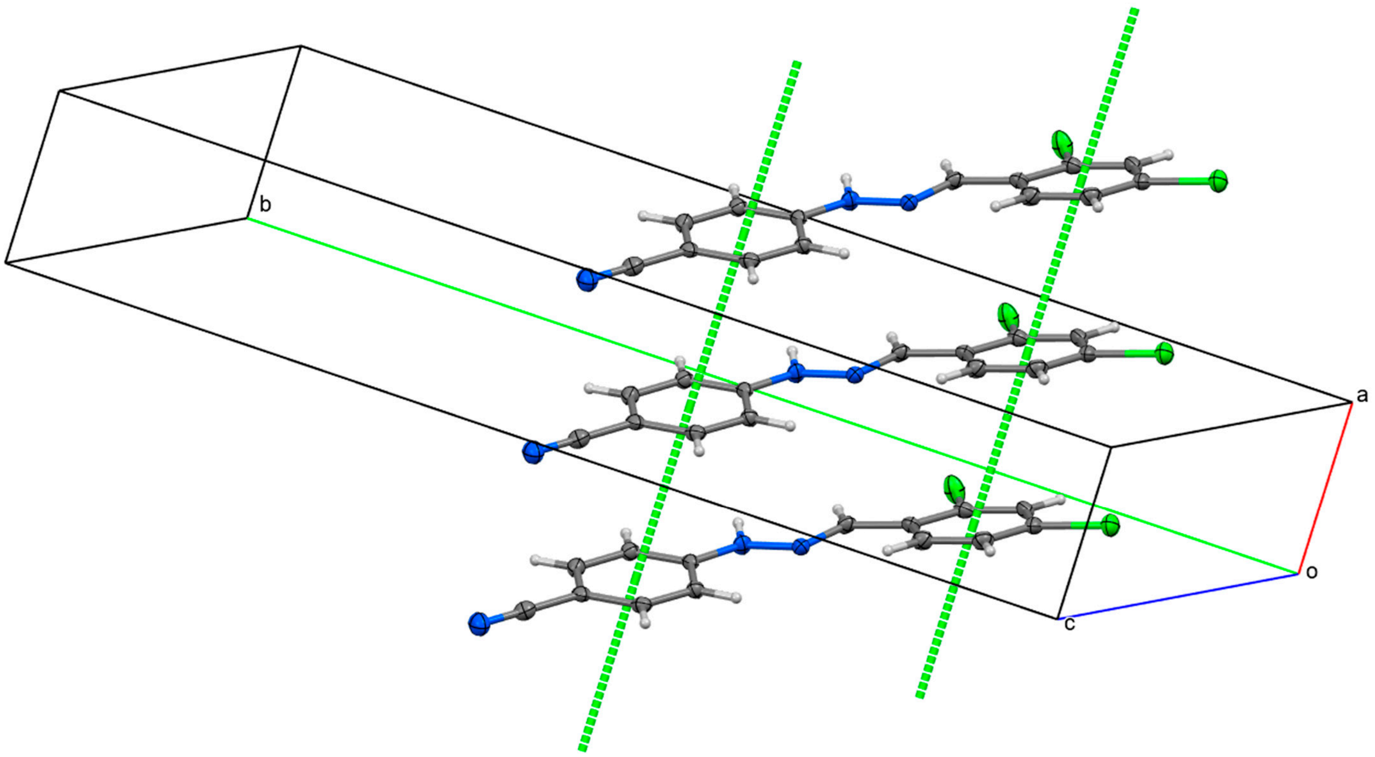

Figure S8. Suprmolecular π•••π stacking columnar system in the structure of **H6**, organized by HH2 motifs.

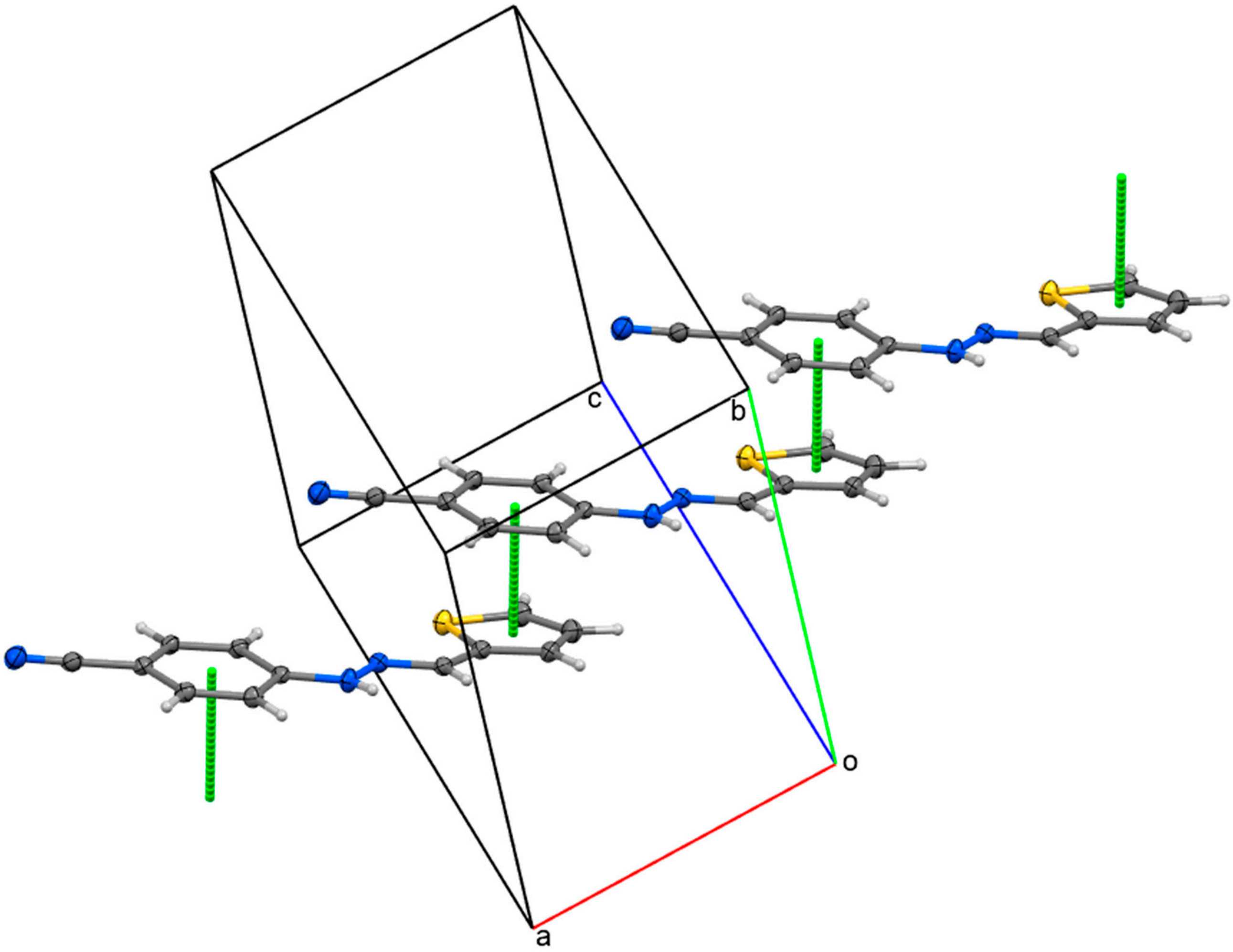

Figure S9. Suprmolecular π•••π stacking columnar system in the structure of **H9**, organized by HH1 motifs.

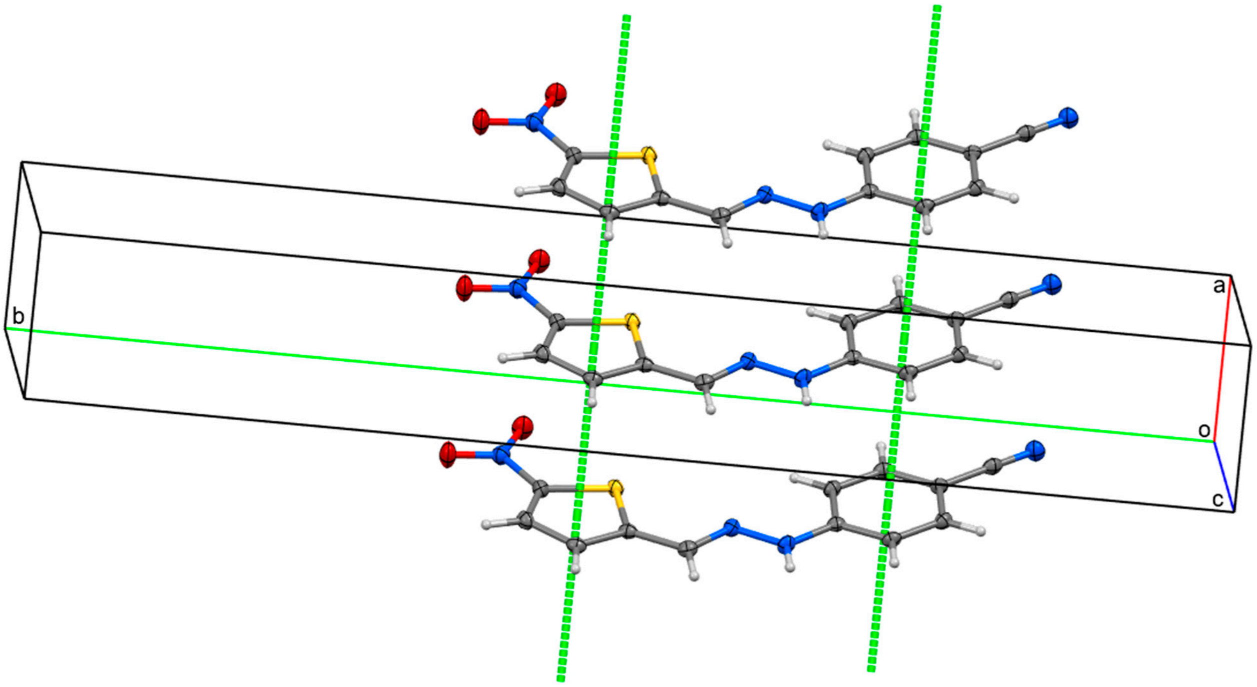

Figure S10. Suprmolecular π•••π stacking columnar system in the structure of **H10**, organized by HH2 motifs.

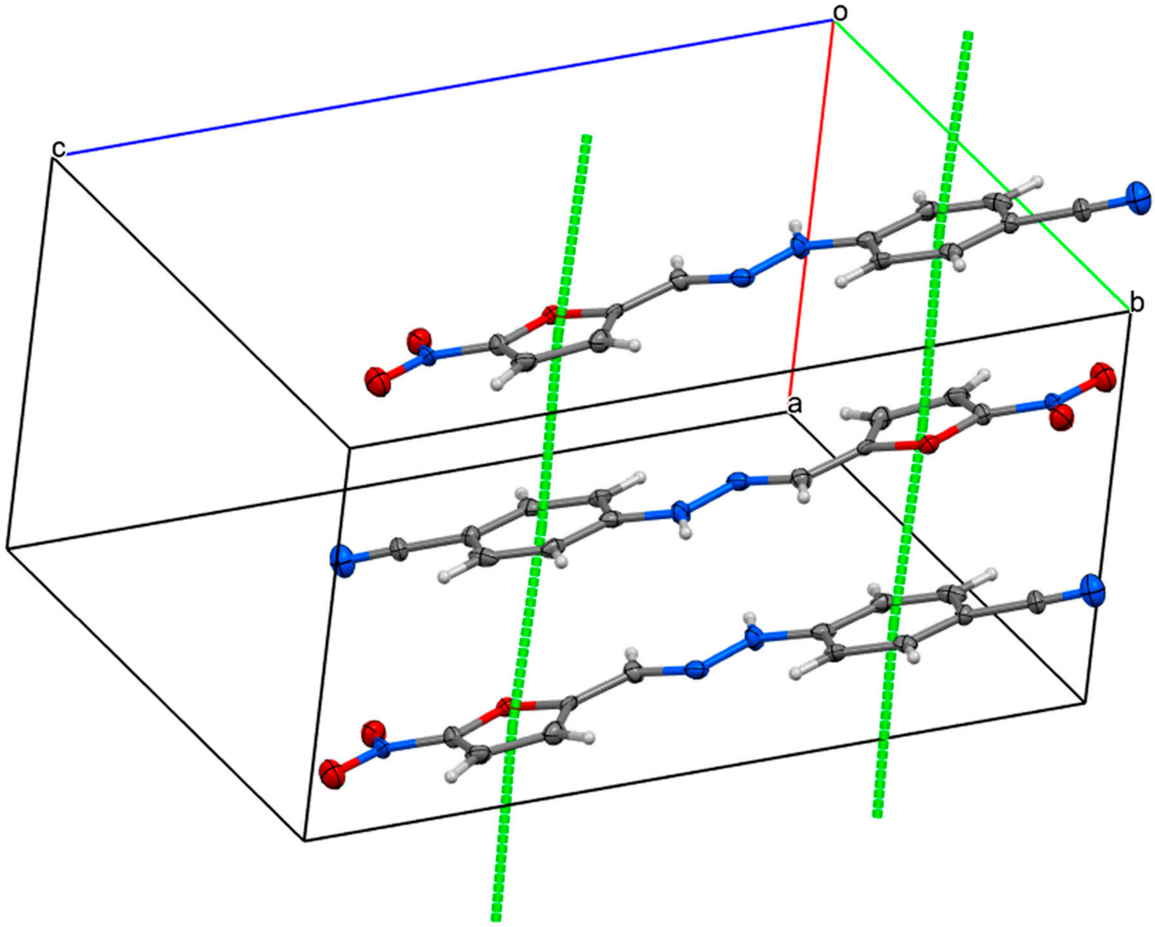

Figure S11. Suprmolecular π•••π stacking columnar system in the structure of **H11**, organized by HT2 motifs.

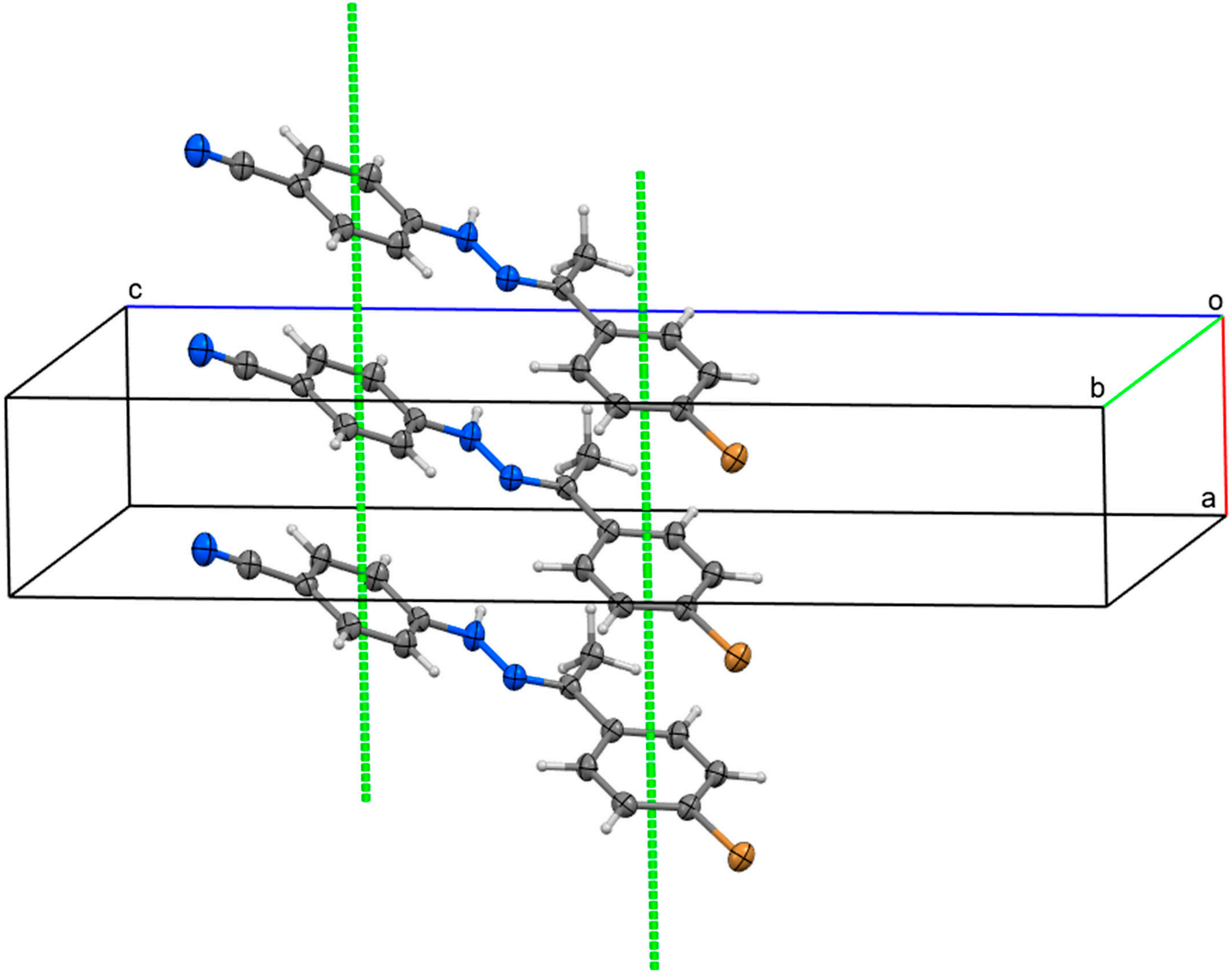

Figure S12. Suprmolecular π•••π stacking columnar system in the structure of **H12**, organized by HH2 motifs.

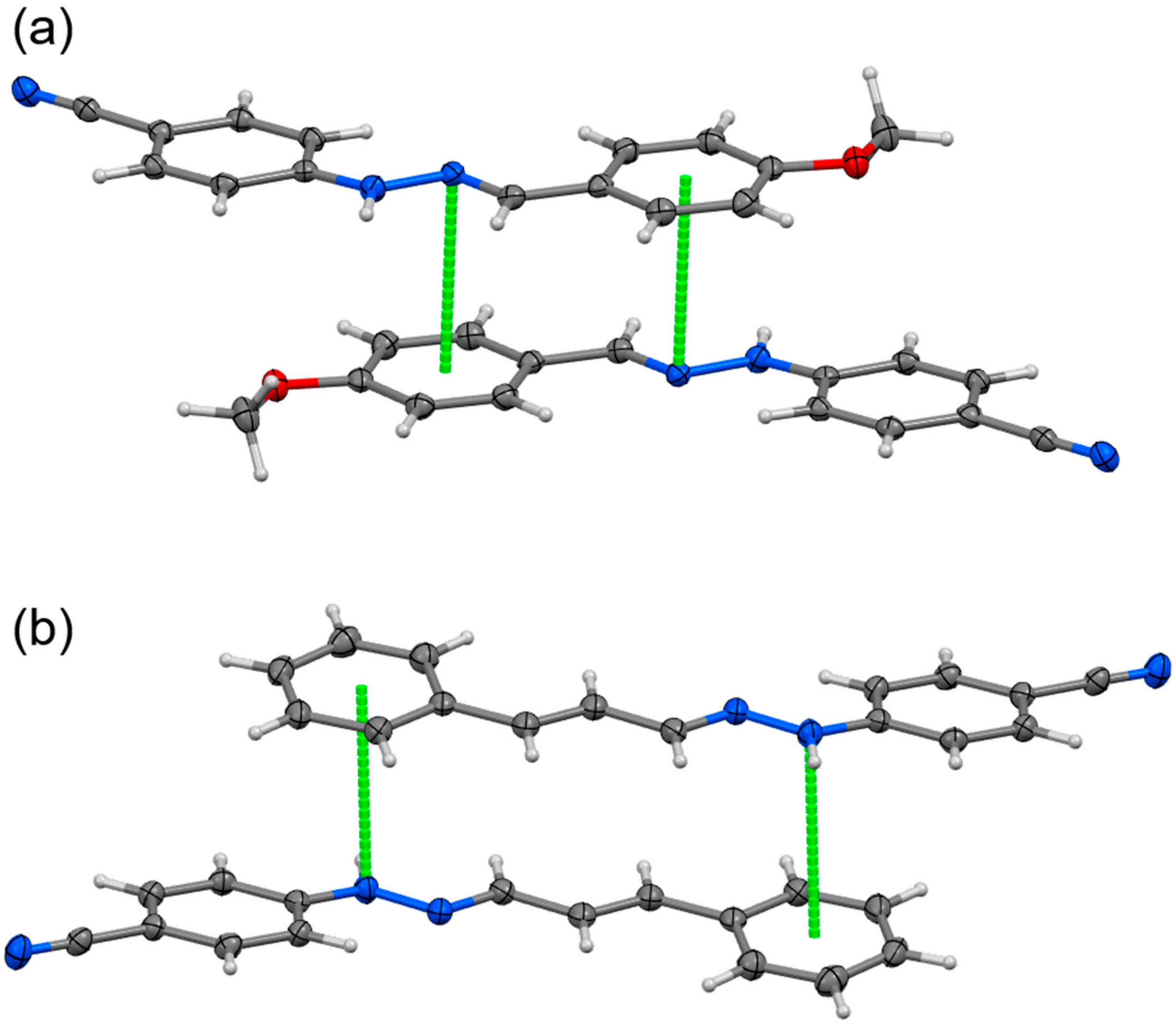

Figure S13. Supramolecular dimers in the structures of **H1** (a) and **H13** (b), organized by hm•••R_2 π•••π interactions.

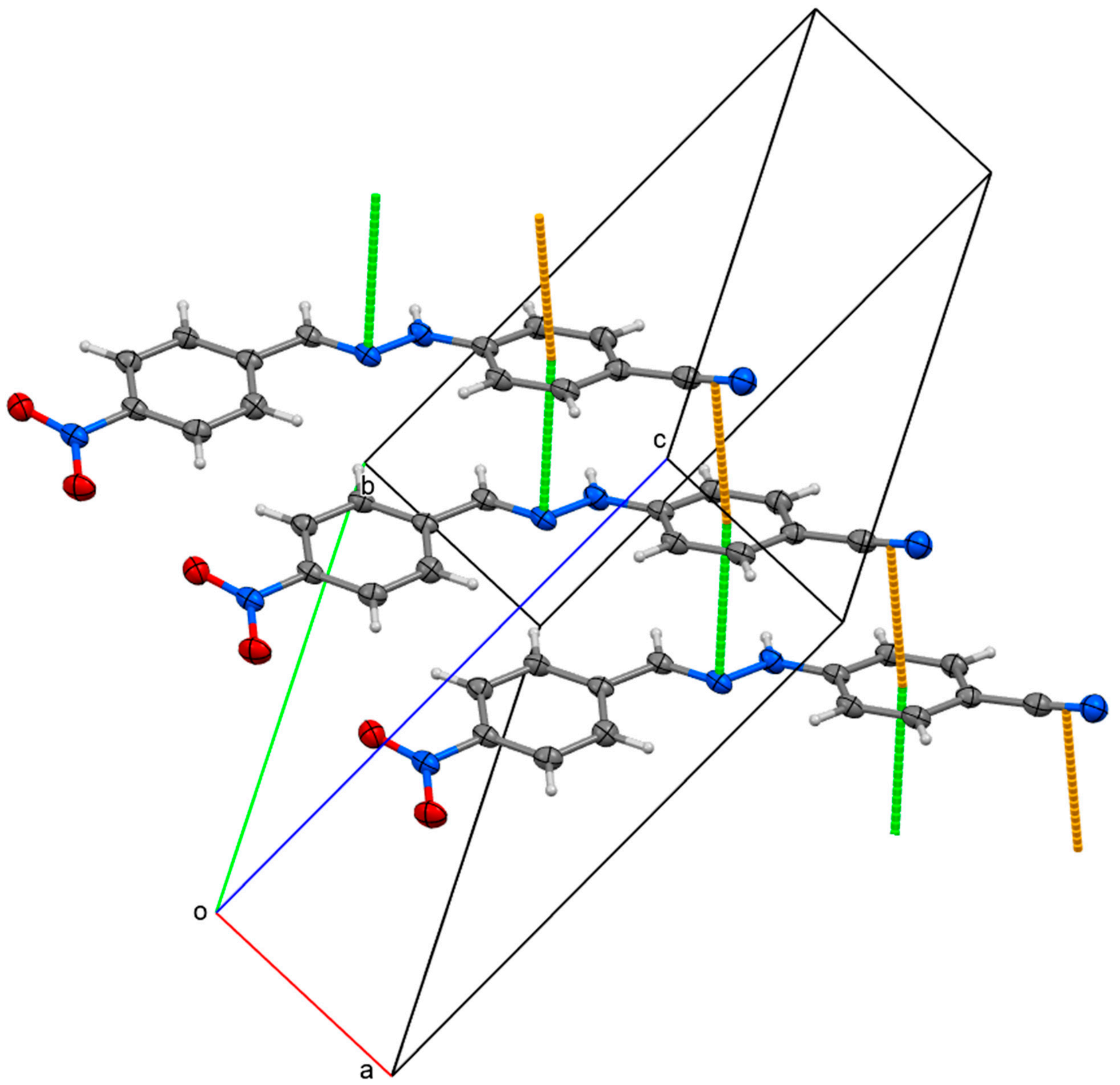

Figure S14. Suprmolecular π–π stacking columnar system in the structure of **H7** organized by hm•••PhCN (green) and CN•••PhCN (orange) π•••π interactions.

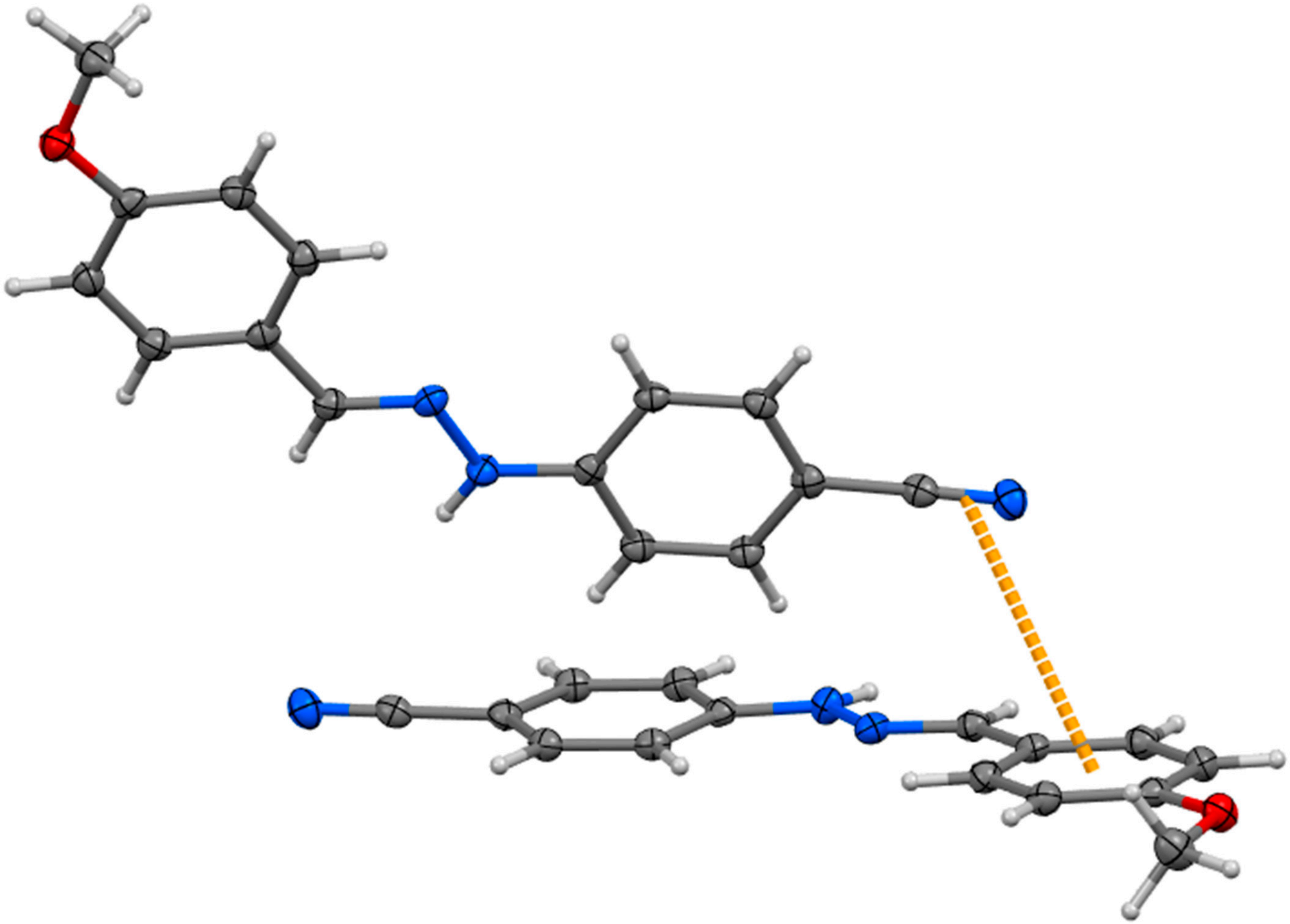

Figure S15. Suprmolecular CN•••PhCN π•••π interactions in the structure of **H1**.

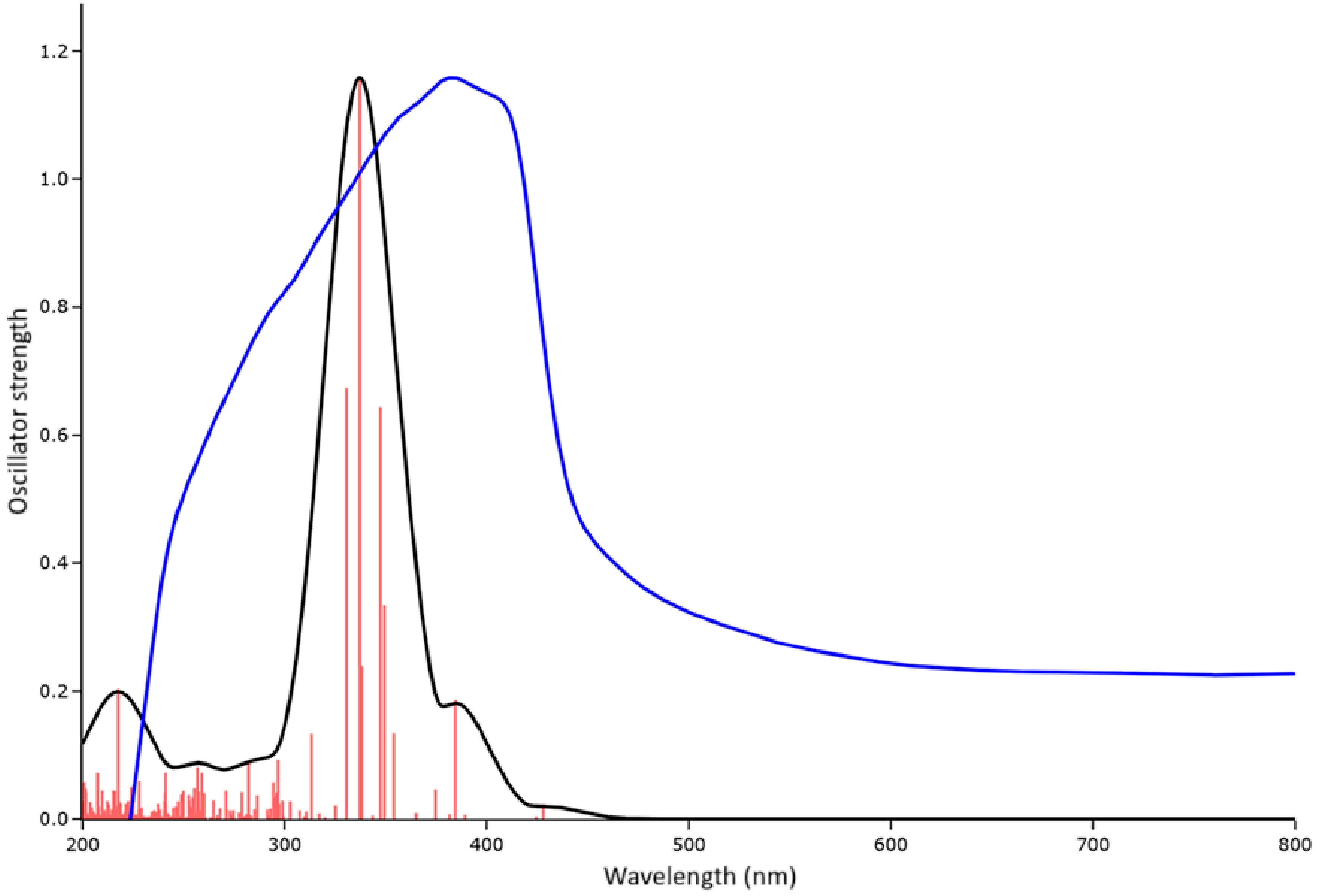

Figure S16. Experimental (blue) and calculated (black) UV-Vis spectrum of **H1**. Oscillator strength are represented as vertical navy-red line.

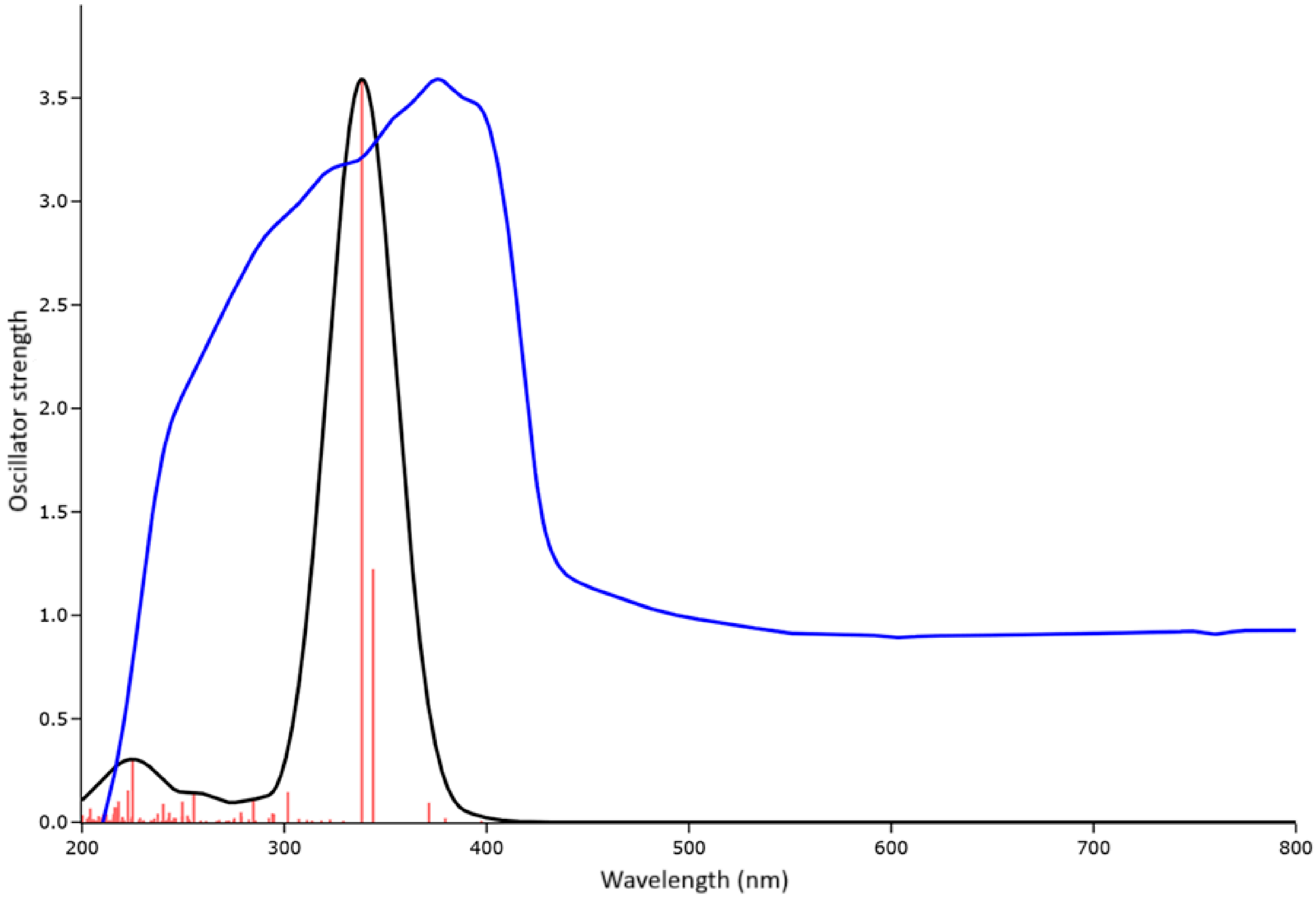

Figure S17. Experimental (blue) and calculated (black) UV-Vis spectrum of **H2**. Oscillator strength are represented as vertical navy-red line.

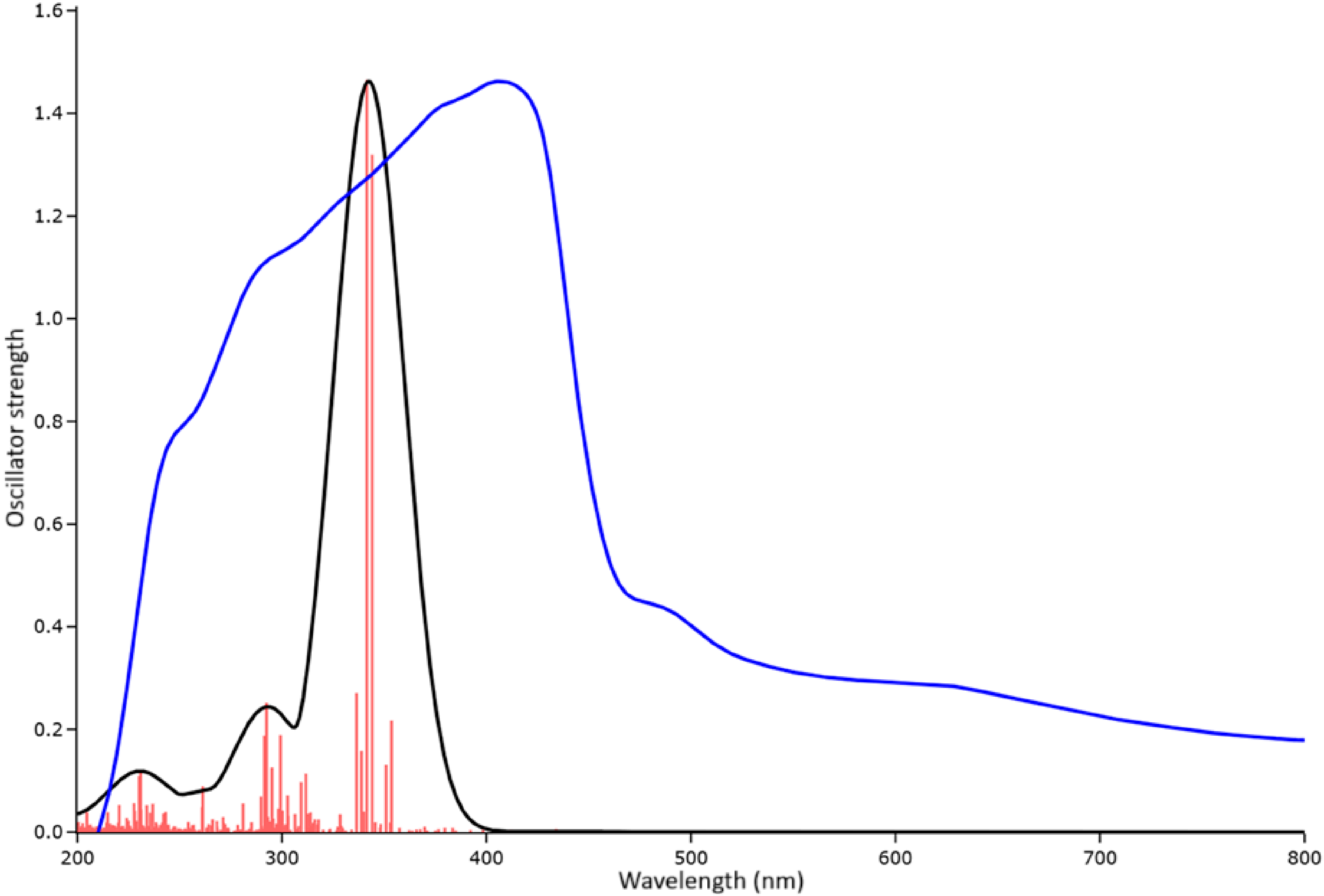

Figure S18. Experimental (blue) and calculated (black) UV-Vis spectrum of **H3**. Oscillator strength are represented as vertical navy-red line.

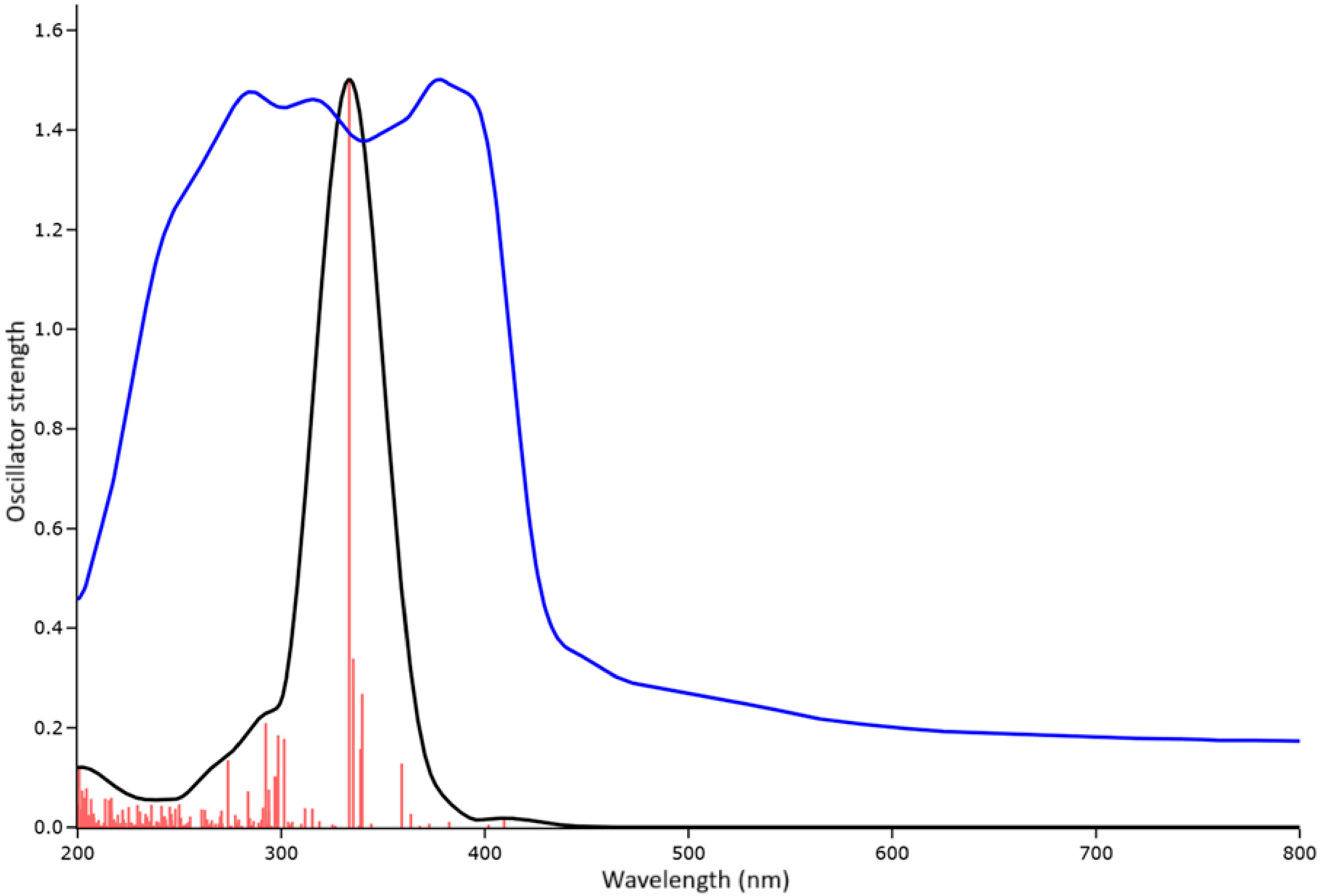

Figure S19. Experimental (blue) and calculated (black) UV-Vis spectrum of **H4**. Oscillator strength are represented as vertical navy-red line.

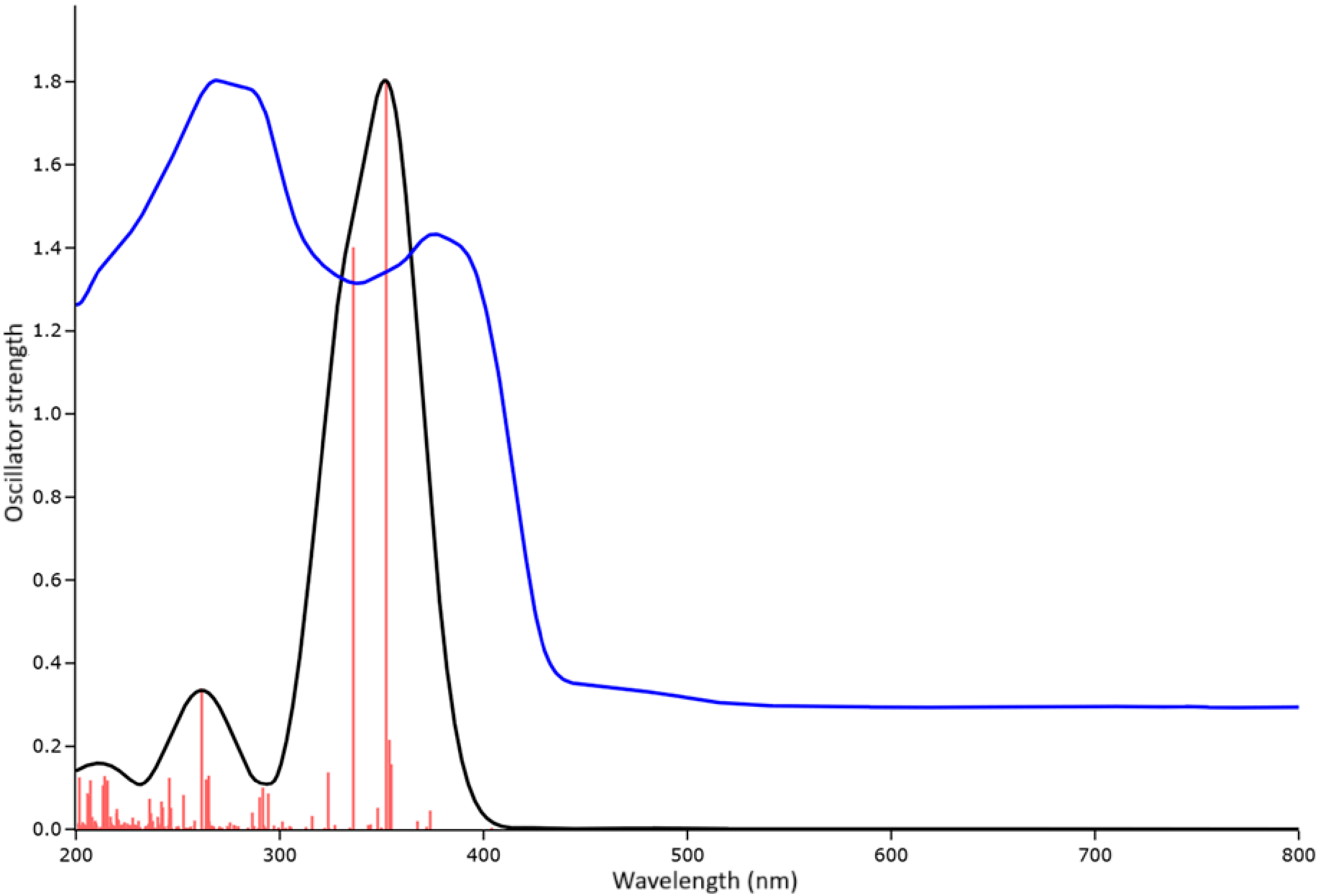

Figure S20. Experimental (blue) and calculated (black) UV-Vis spectrum of **H5**. Oscillator strength are represented as vertical navy-red line.

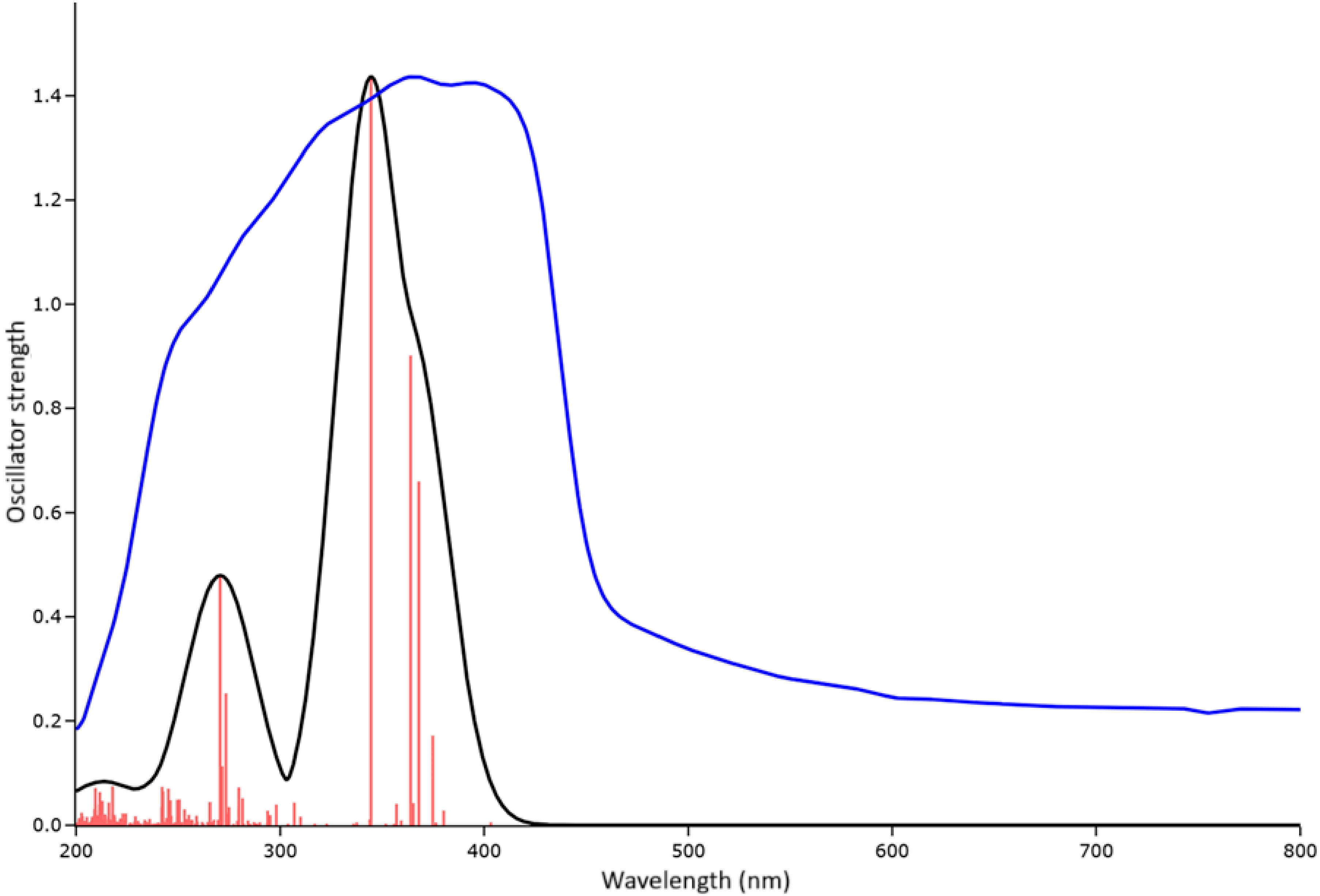

Figure S21. Experimental (blue) and calculated (black) UV-Vis spectrum of **H6**. Oscillator strength are represented as vertical navy-red line.

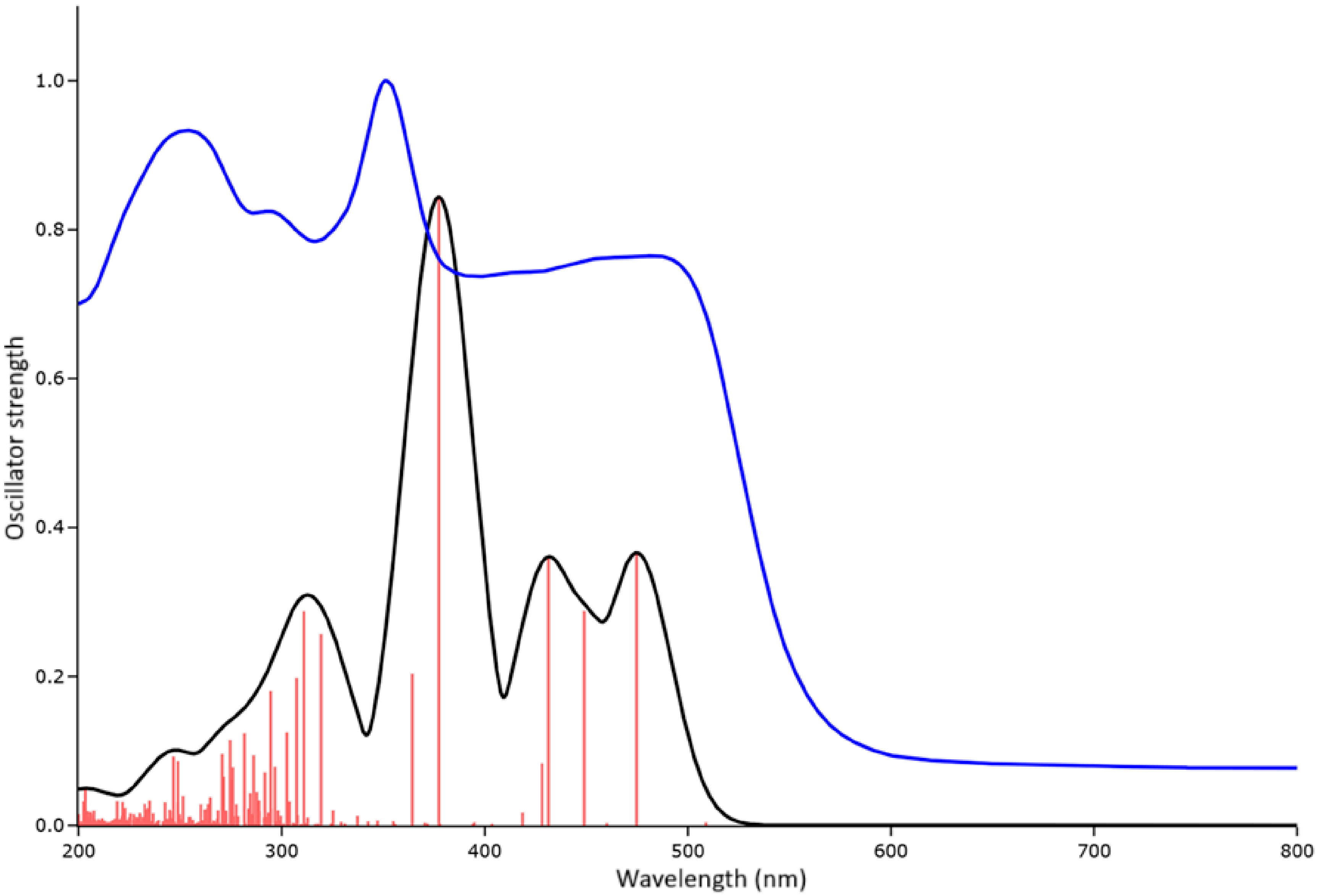

Figure S22. Experimental (blue) and calculated (black) UV-Vis spectrum of **H7**. Oscillator strength are represented as vertical navy-red line.

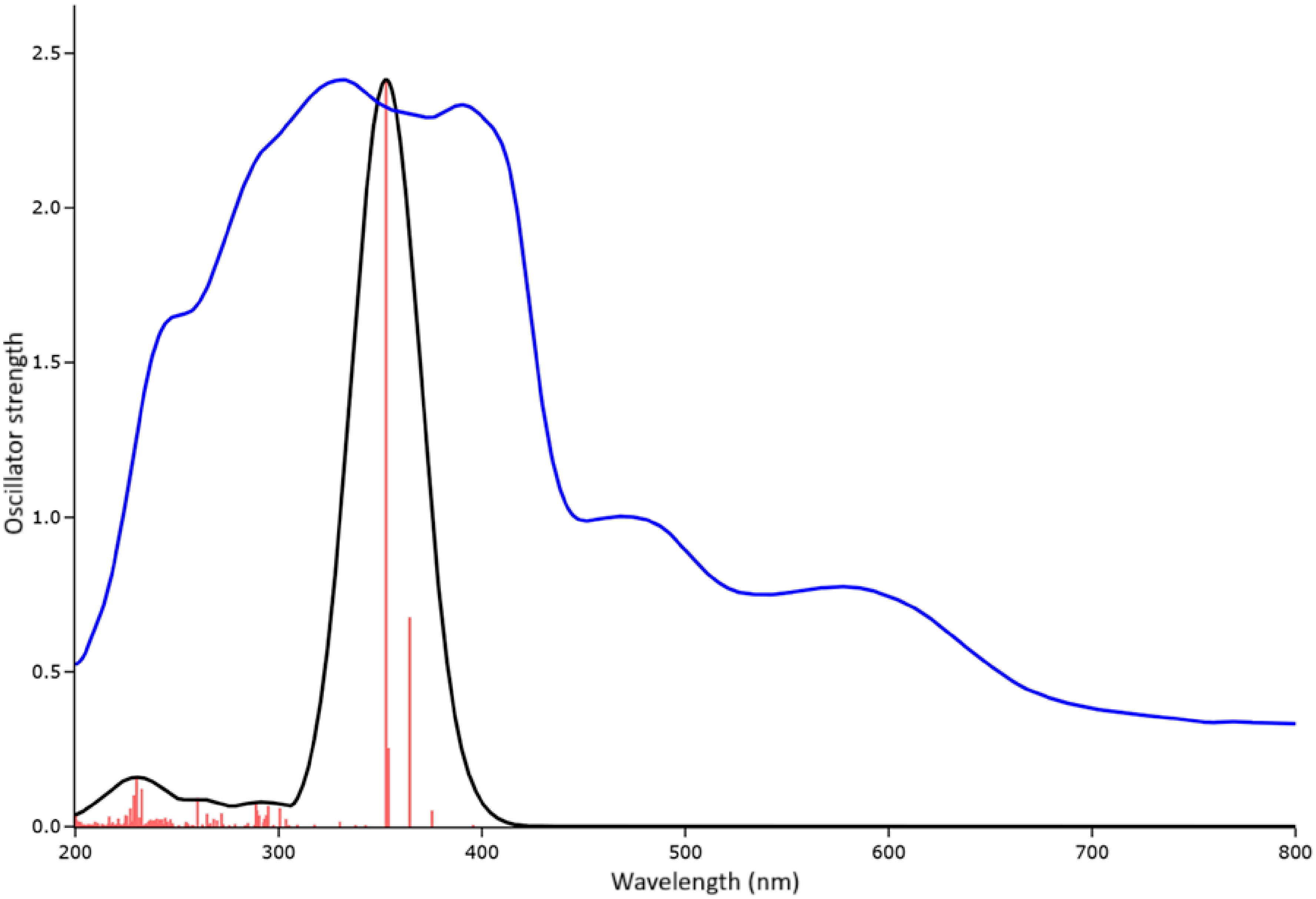

Figure S23. Experimental (blue) and calculated (black) UV-Vis spectrum of **H8**. Oscillator strength are represented as vertical navy-red line.

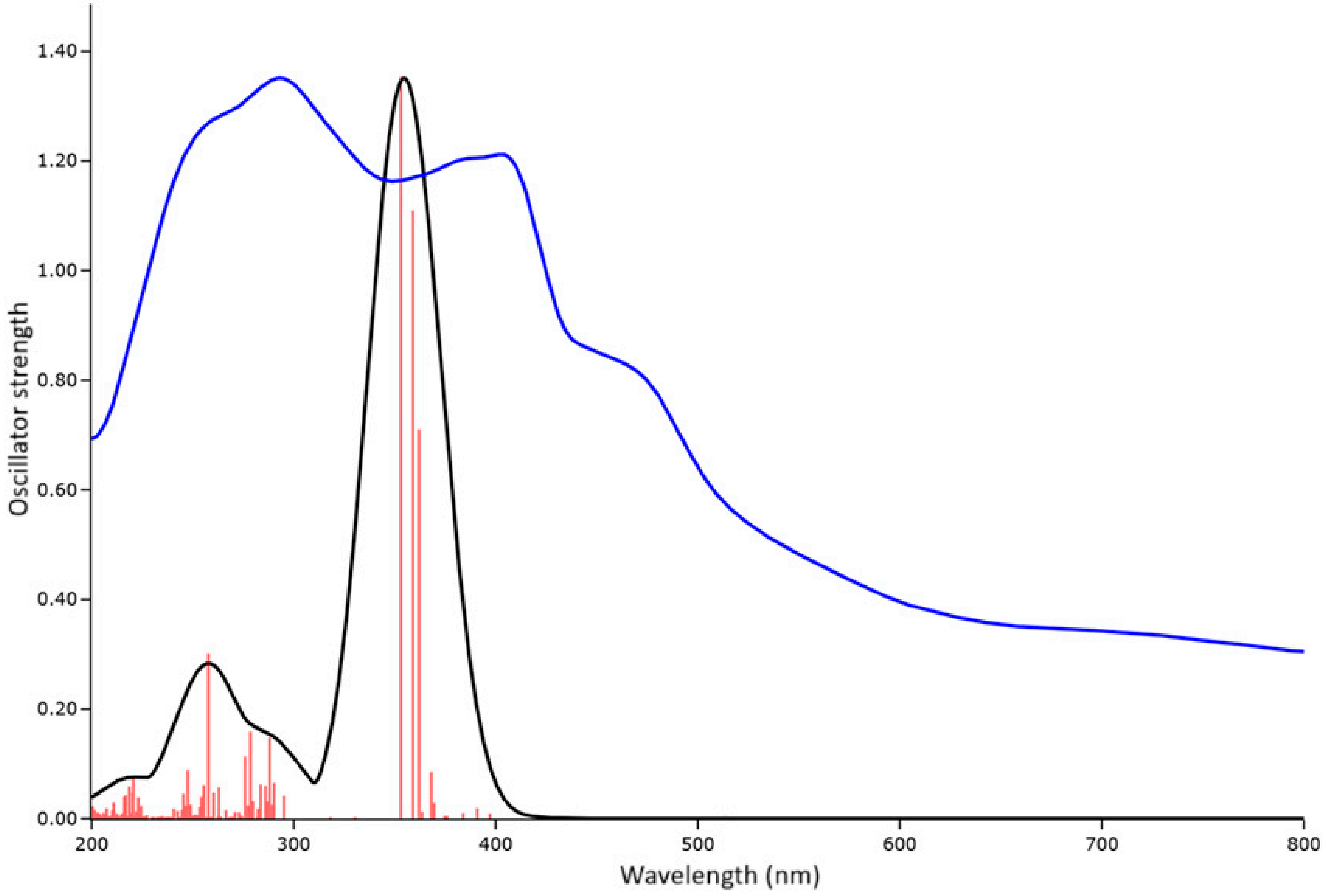

Figure S24. Experimental (blue) and calculated (black) UV-Vis spectrum of **H9**. Oscillator strength are represented as vertical navy-red line.

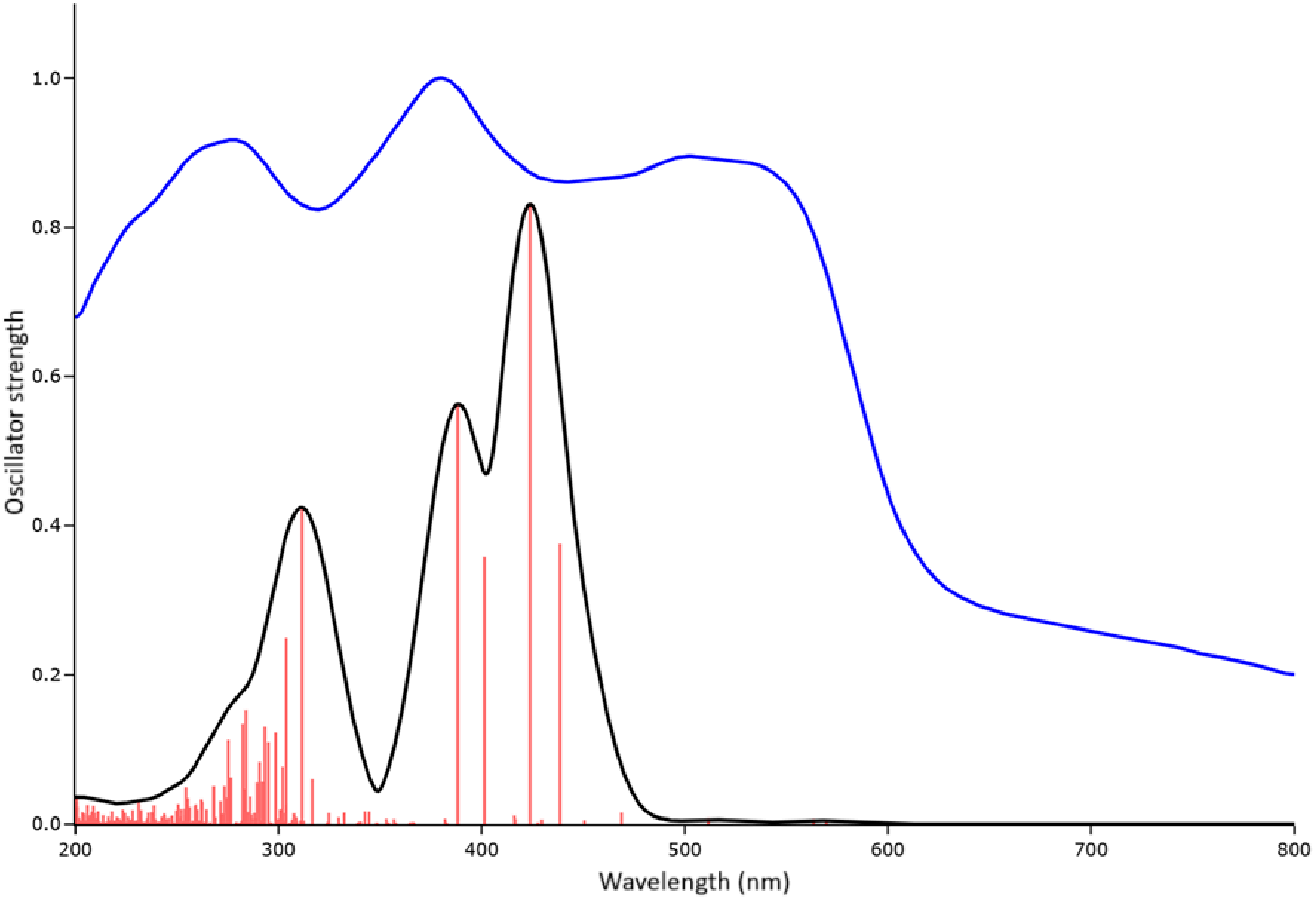

Figure S25. Experimental (blue) and calculated (black) UV-Vis spectrum of **H10**. Oscillator strength are represented as vertical navy-red line.

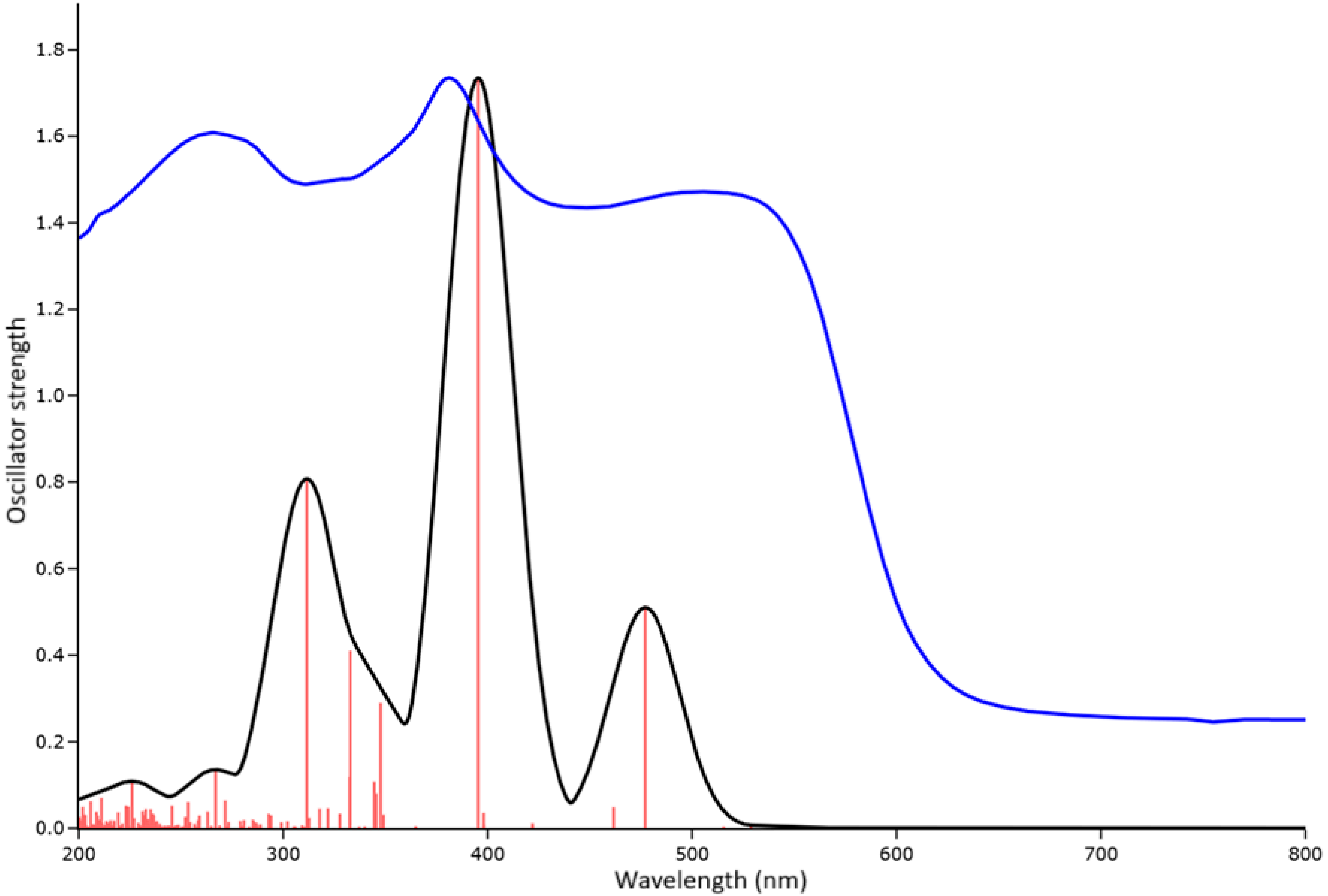

Figure S26. Experimental (blue) and calculated (black) UV-Vis spectrum of **H11**. Oscillator strength are represented as vertical navy-red line.

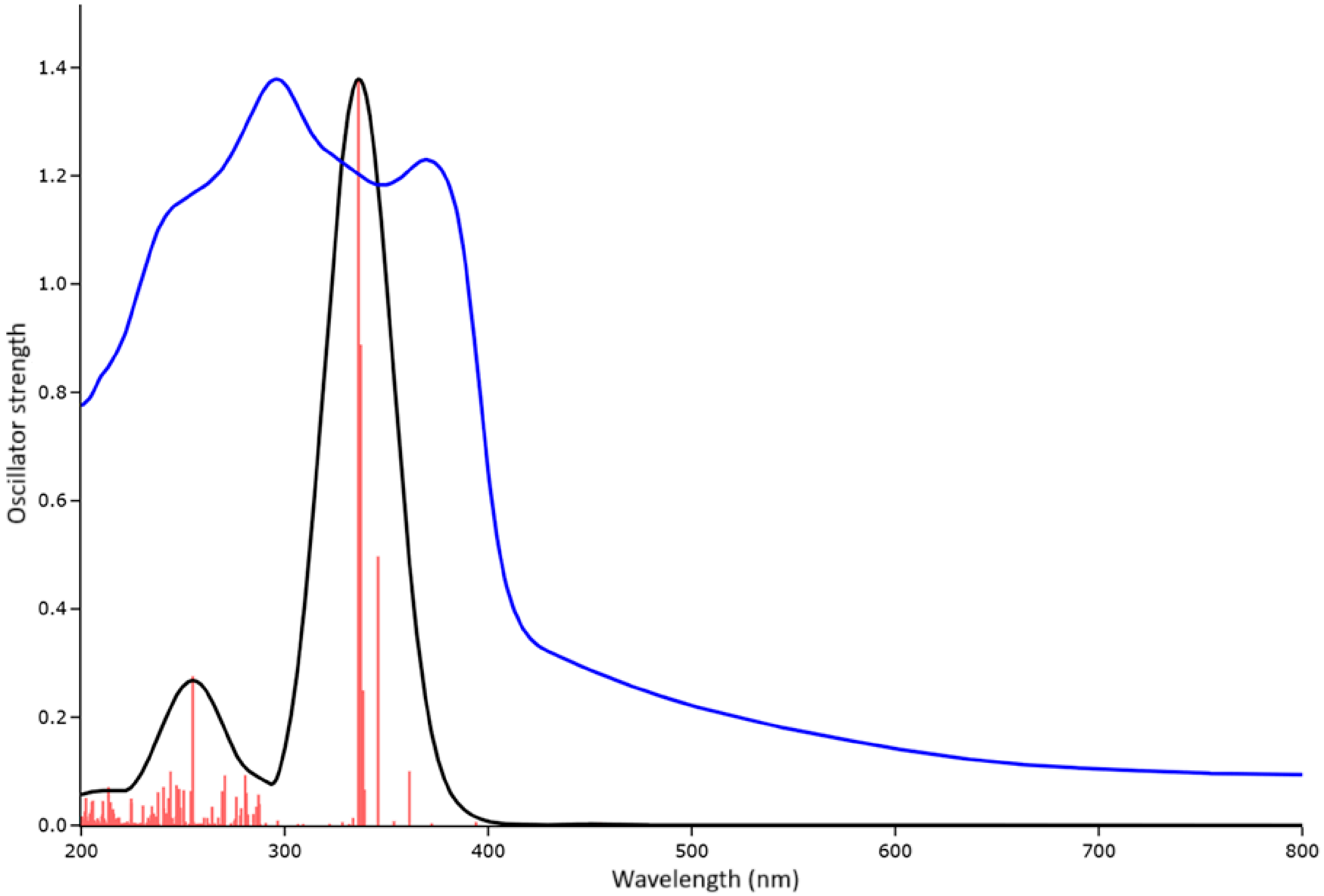

Figure S27. Experimental (blue) and calculated (black) UV-Vis spectrum of **H12**. Oscillator strength are represented as vertical navy-red line.

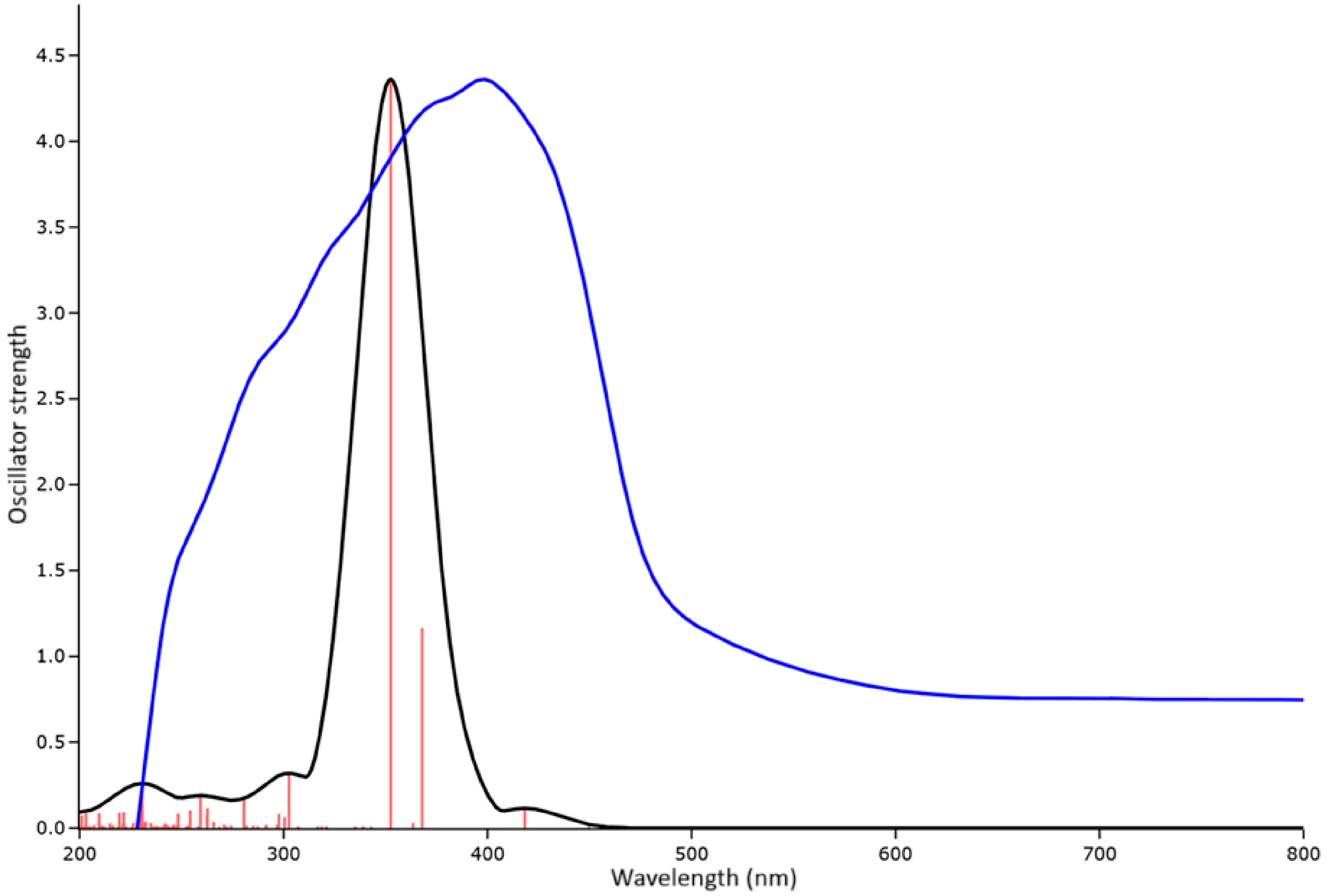

Figure S28. Experimental (blue) and calculated (black) UV-Vis spectrum of **H13.** Oscillator strength are represented as vertical navy-red line.

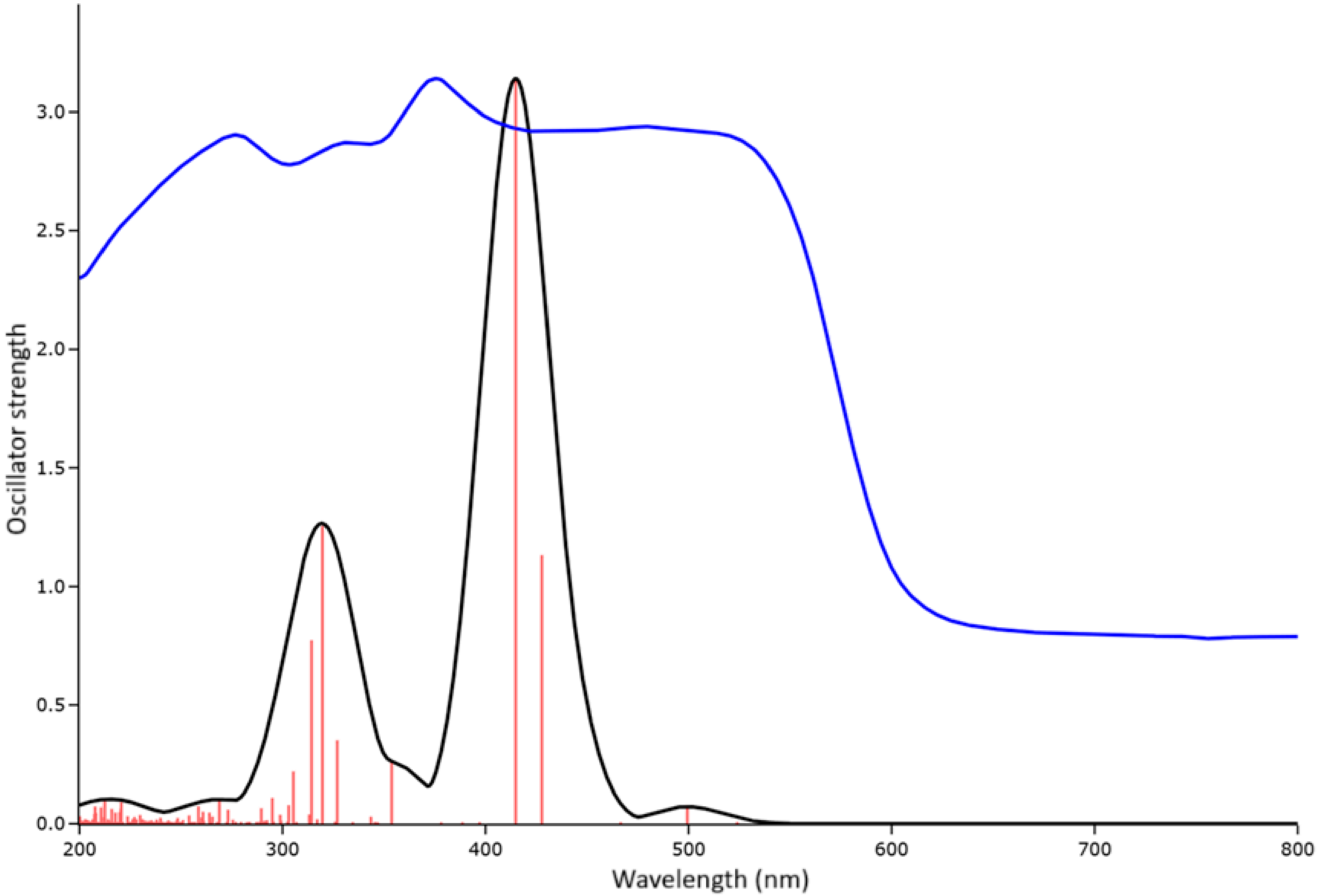

Figure S29. Experimental (blue) and calculated (black) UV-Vis spectrum of **H14**. Oscillator strength are represented as vertical navy-red line.

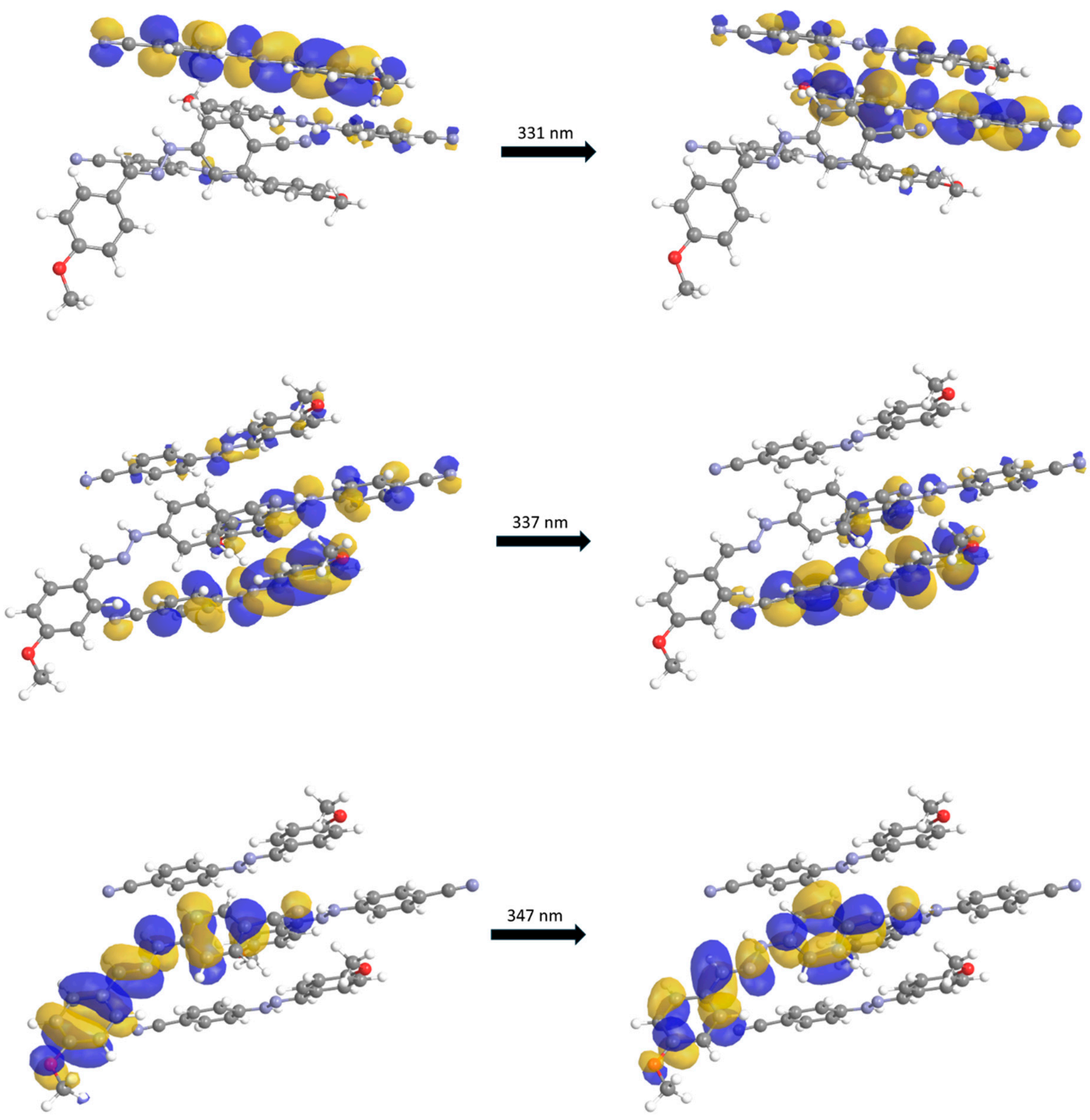

Figure S30. Calculated molecular orbital transition in the studied compound (**H1**).

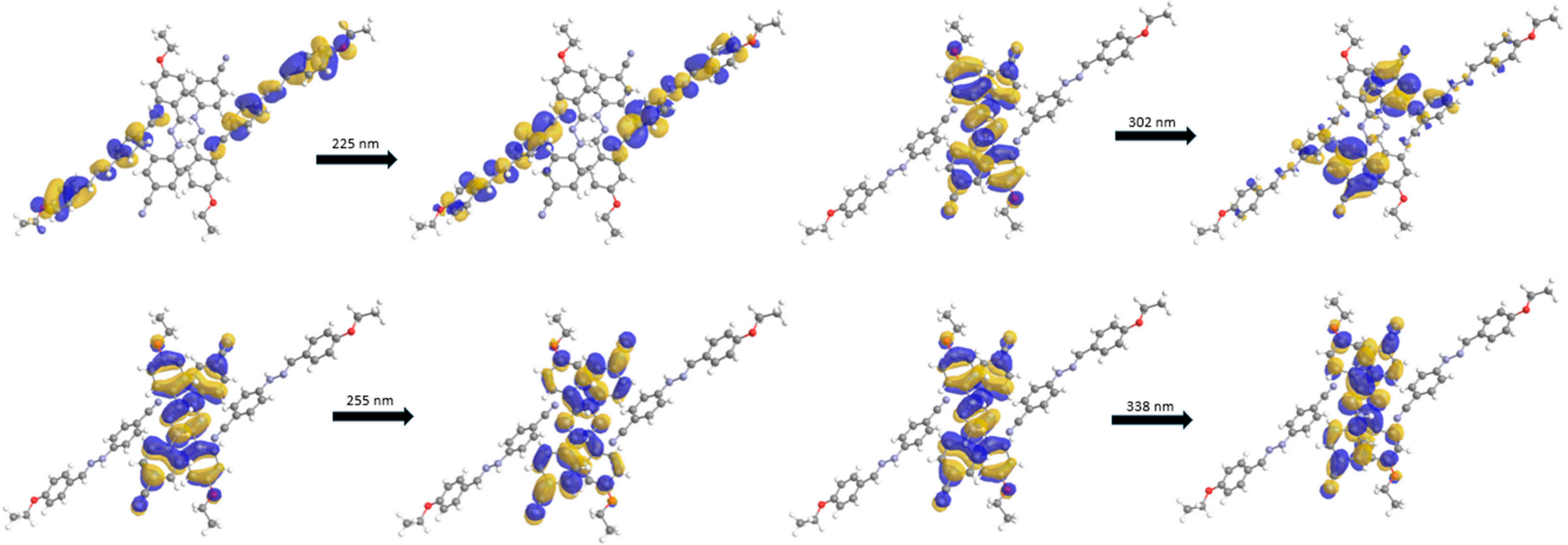

Figure S31. Calculated molecular orbital transition in the studied compound **(H2)**.

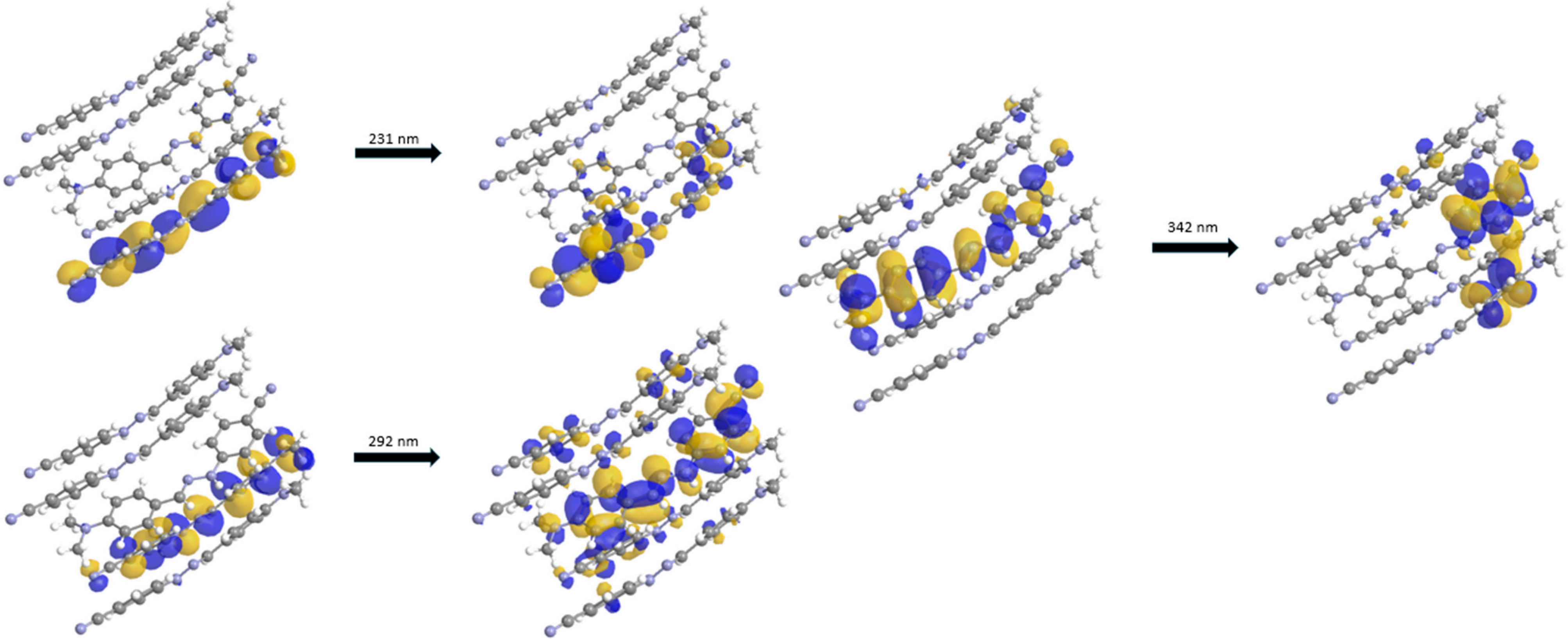

Figure S32. Calculated molecular orbital transition in the studied compound **(H3)**.

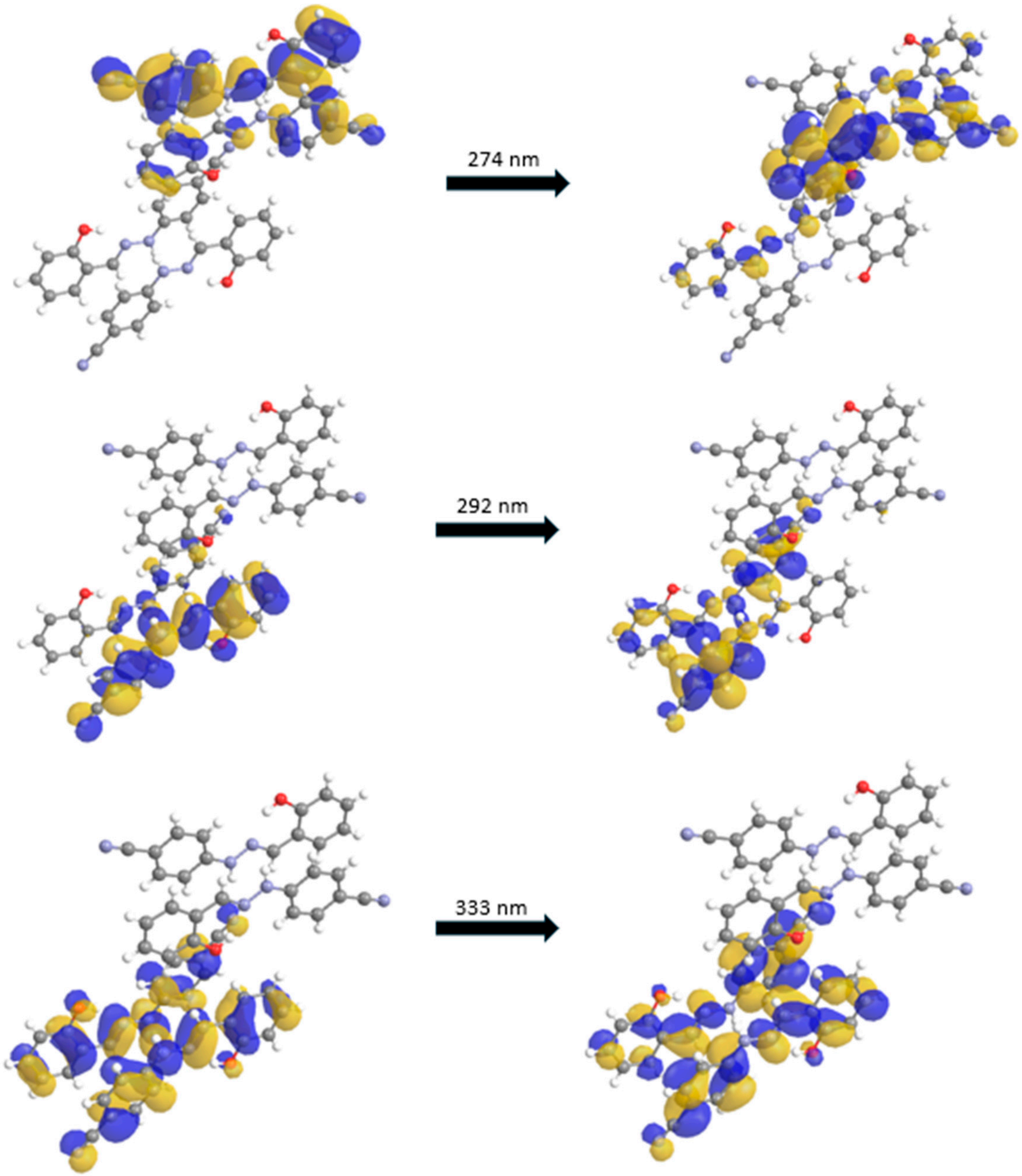

Figure S33. Calculated molecular orbital transition in the studied compound **(H4)**.

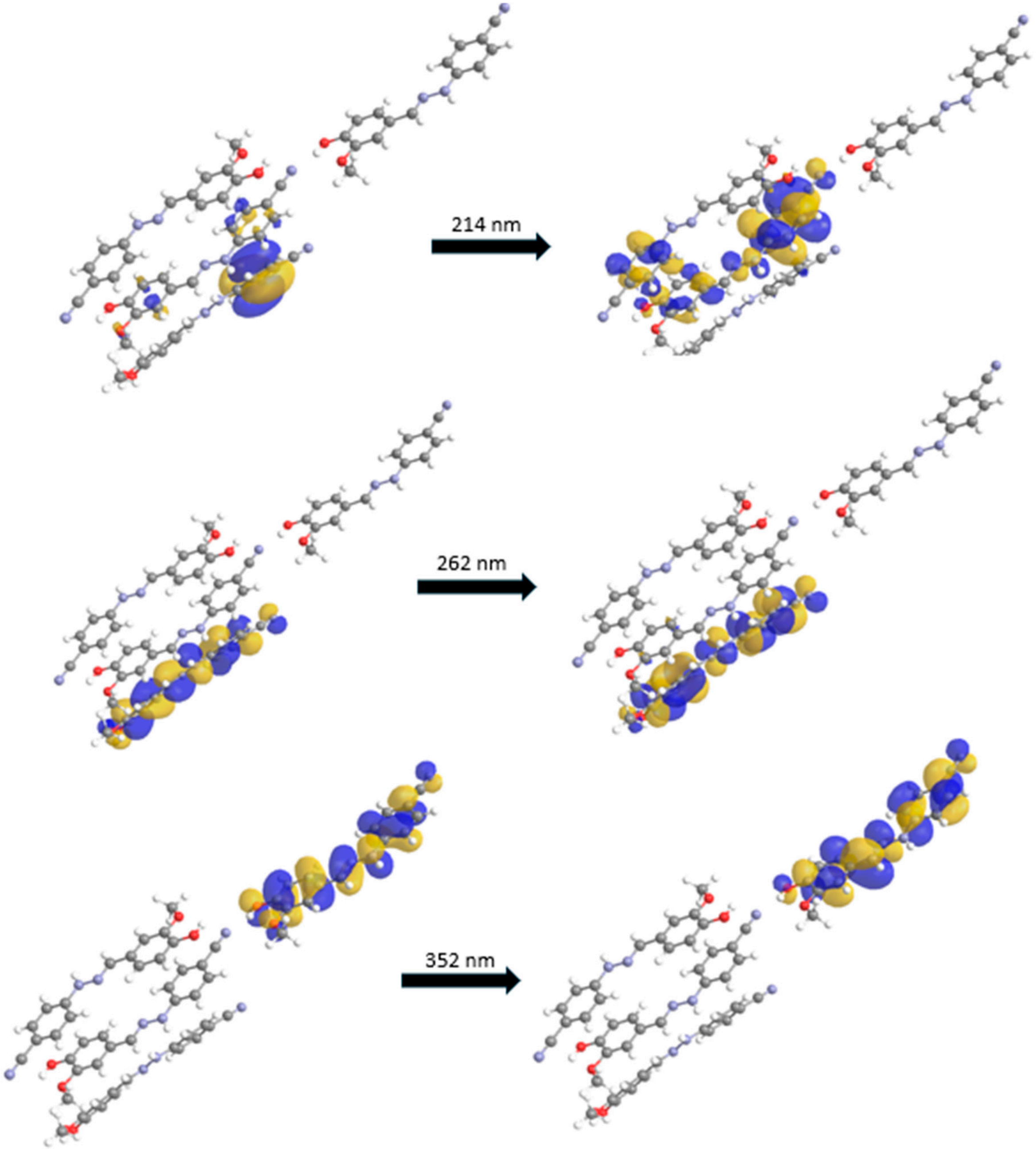

Figure S34. Calculated molecular orbital transition in the studied compound **(H5)**.

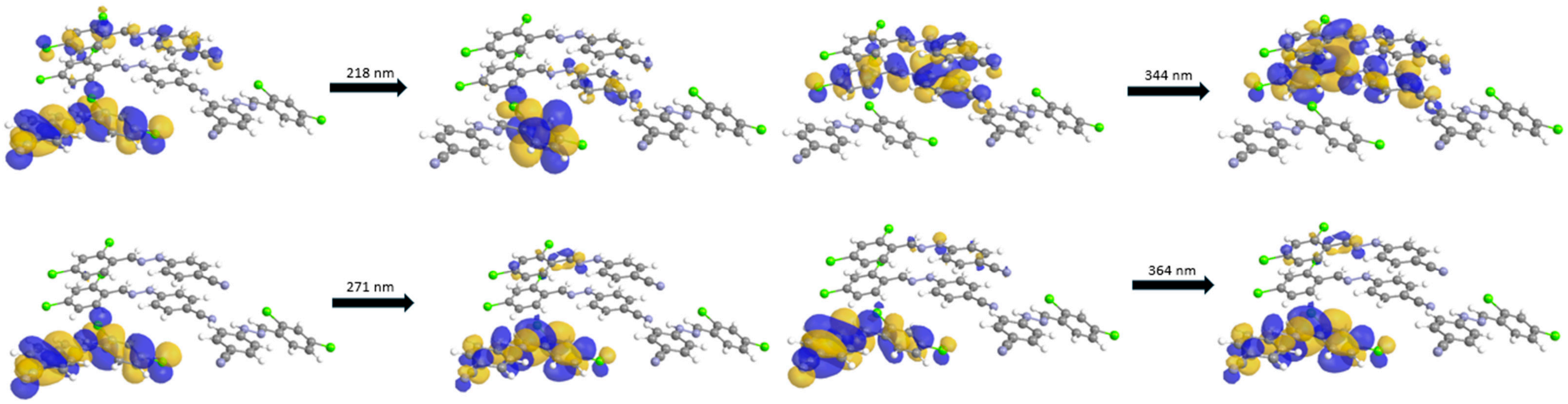

Figure S35. Calculated molecular orbital transition in the studied compound **(H6)**.

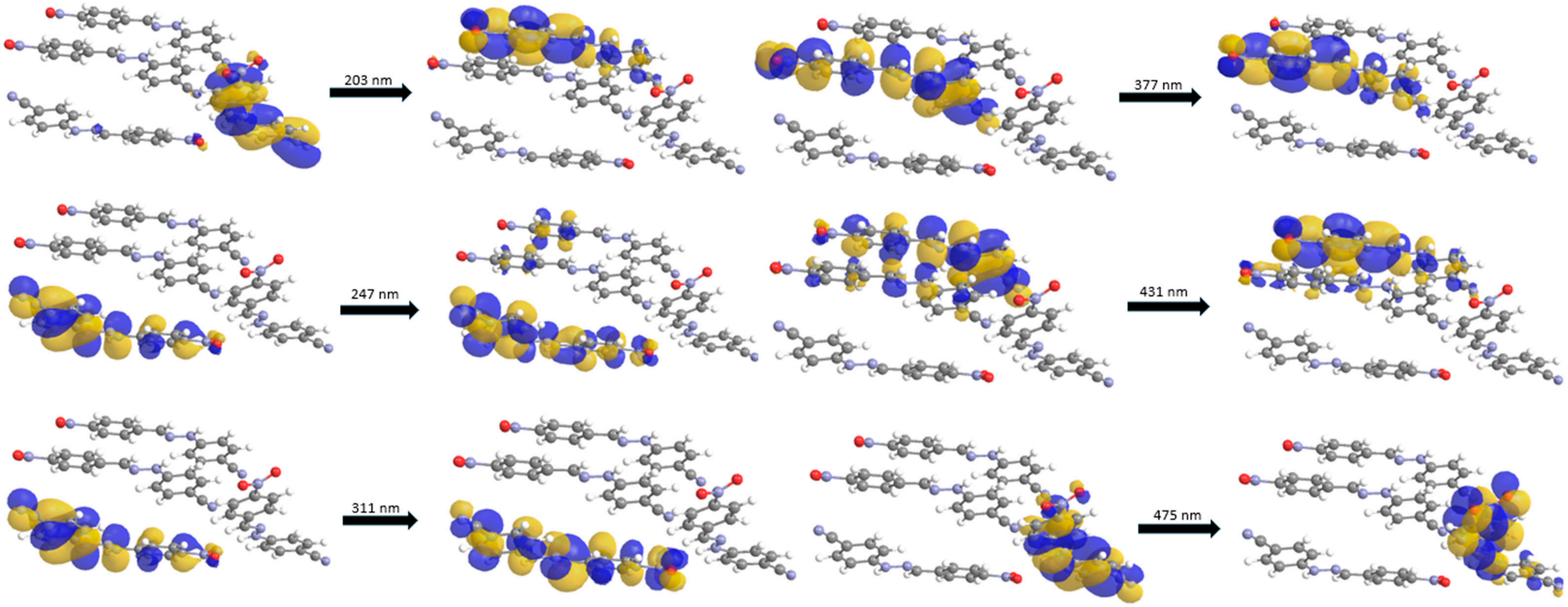

Figure S36. Calculated molecular orbital transition in the studied compound **(H7)**.

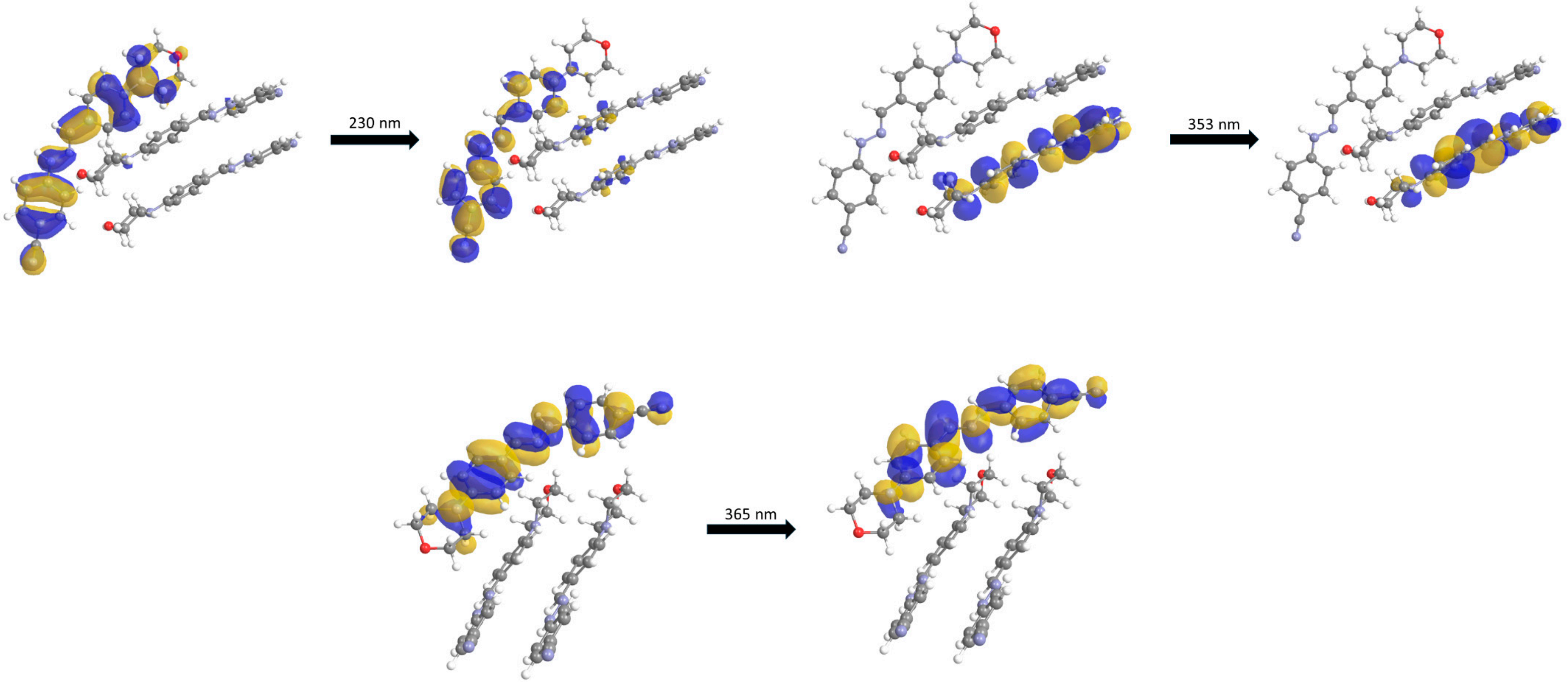

Figure S37. Calculated molecular orbital transition in the studied compound **(H8)**.

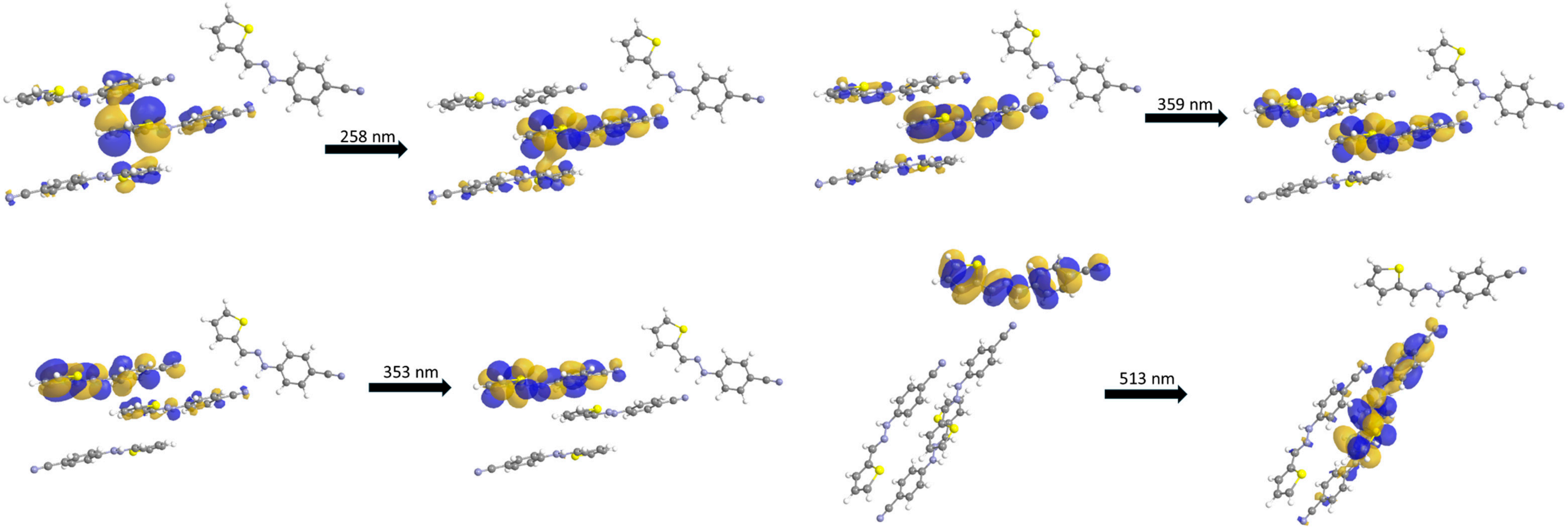

Figure S38. Calculated molecular orbital transition in the studied compound **(H9)**.

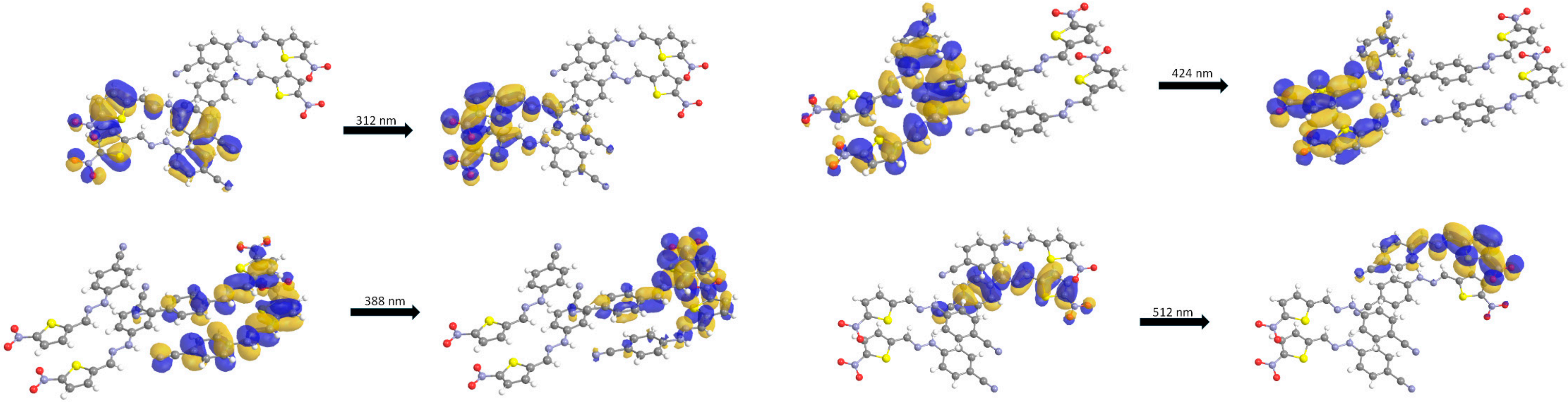

Figure S39. Calculated molecular orbital transition in the studied compound **(H10)**.

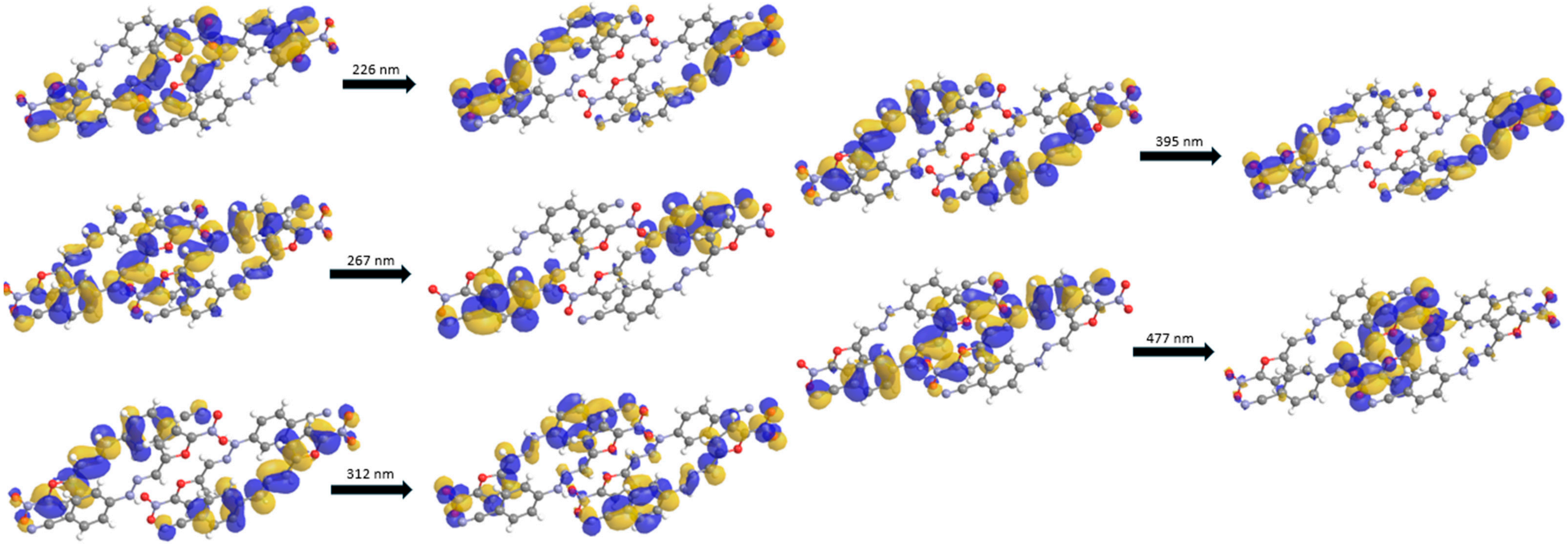

Figure S40. Calculated molecular orbital transition in the studied compound **(H11)**.

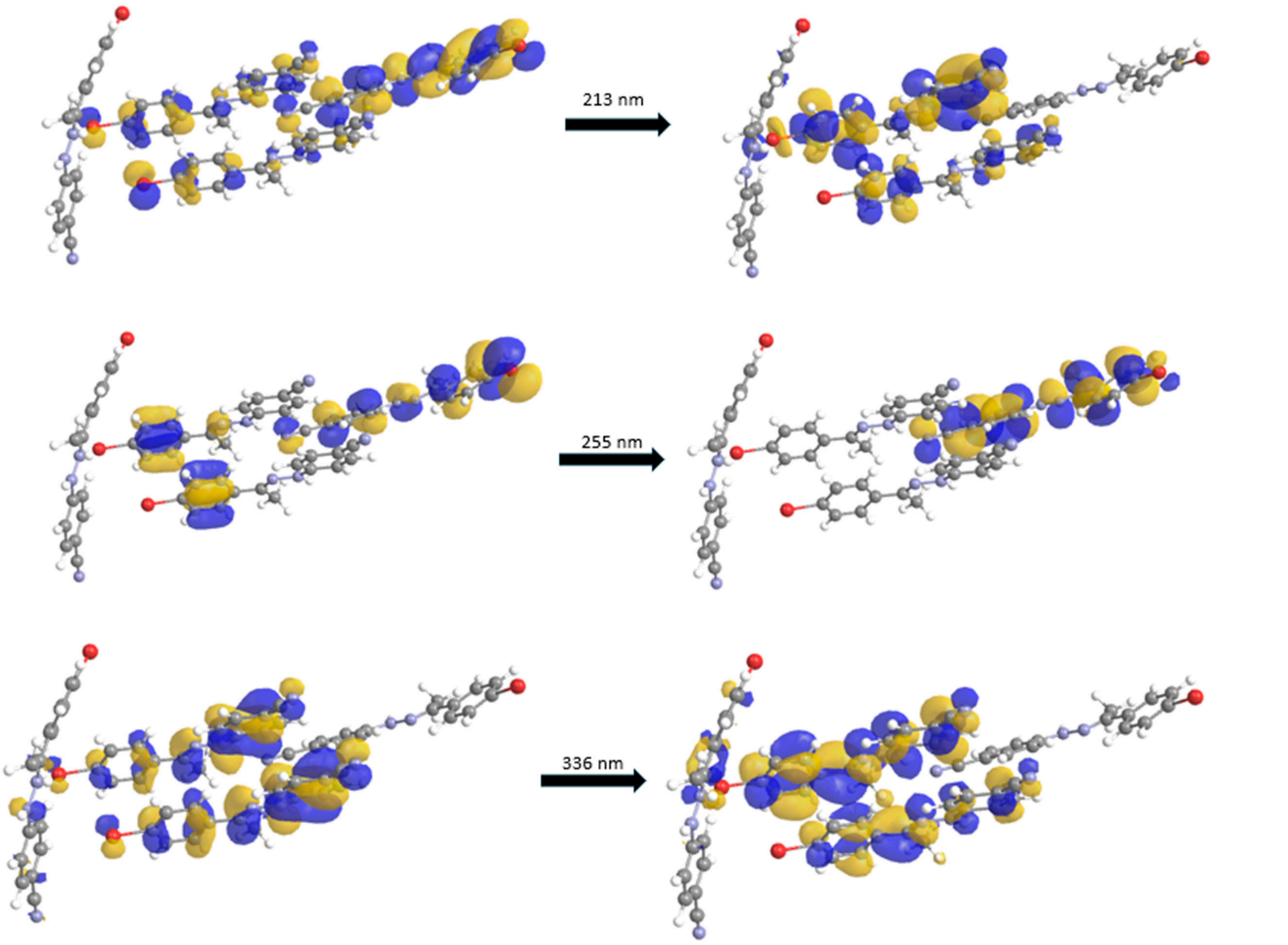

Figure S41. Calculated molecular orbital transition in the studied compound **(H12)**.

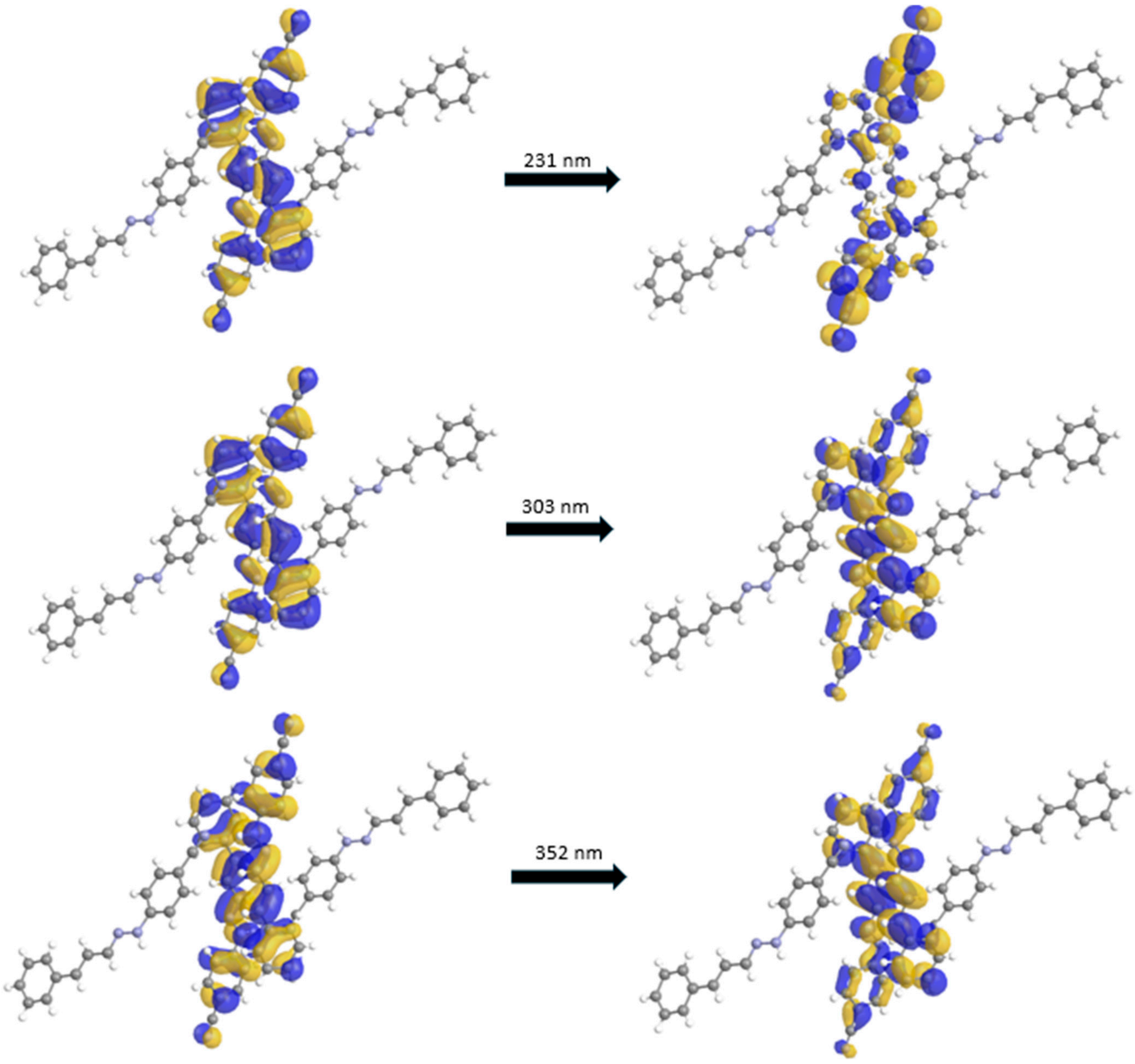

Figure S42. Calculated molecular orbital transition in the studied compound **(H13)**.

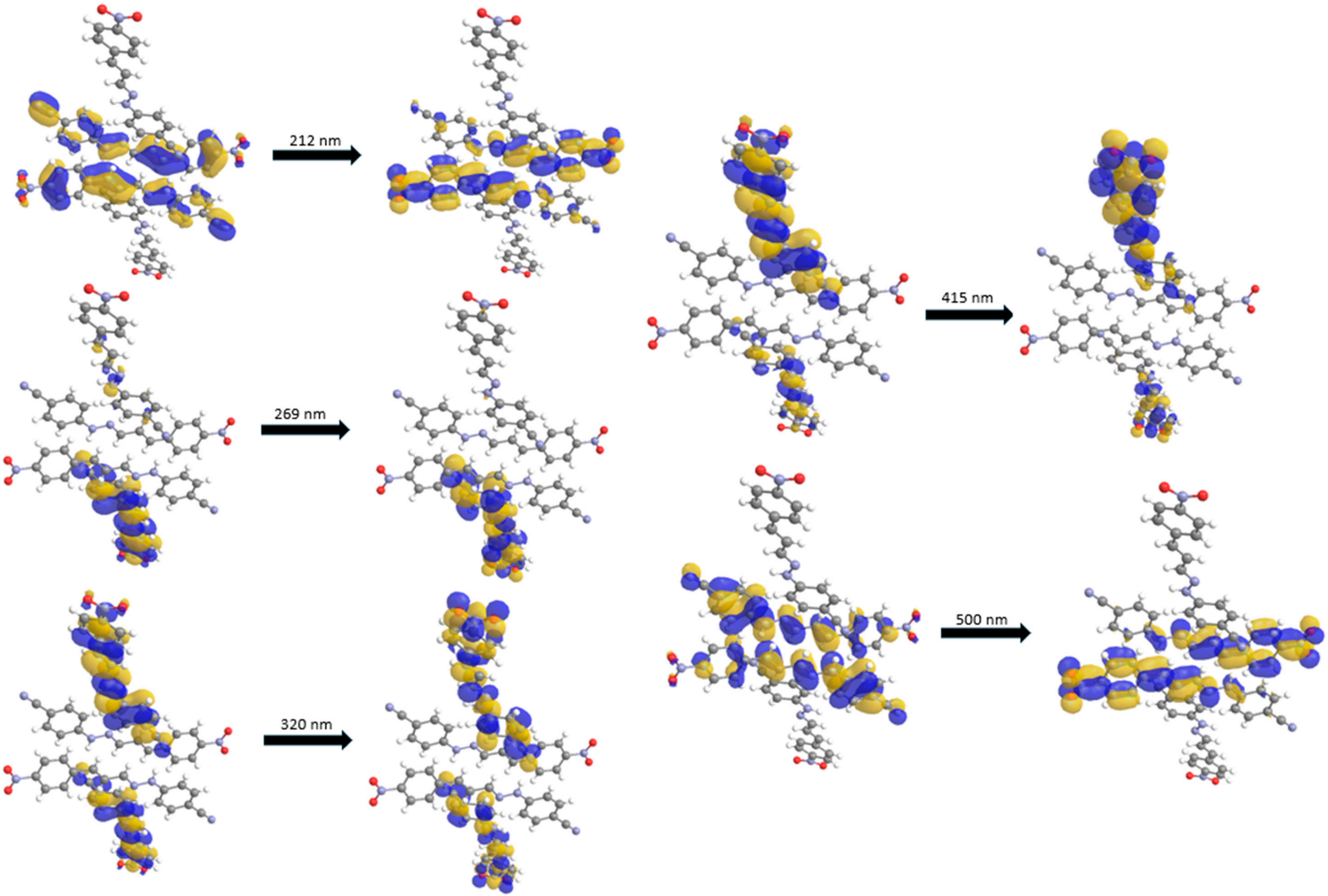

Figure S43. Calculated molecular orbital transition in the studied compound **(H14)**.

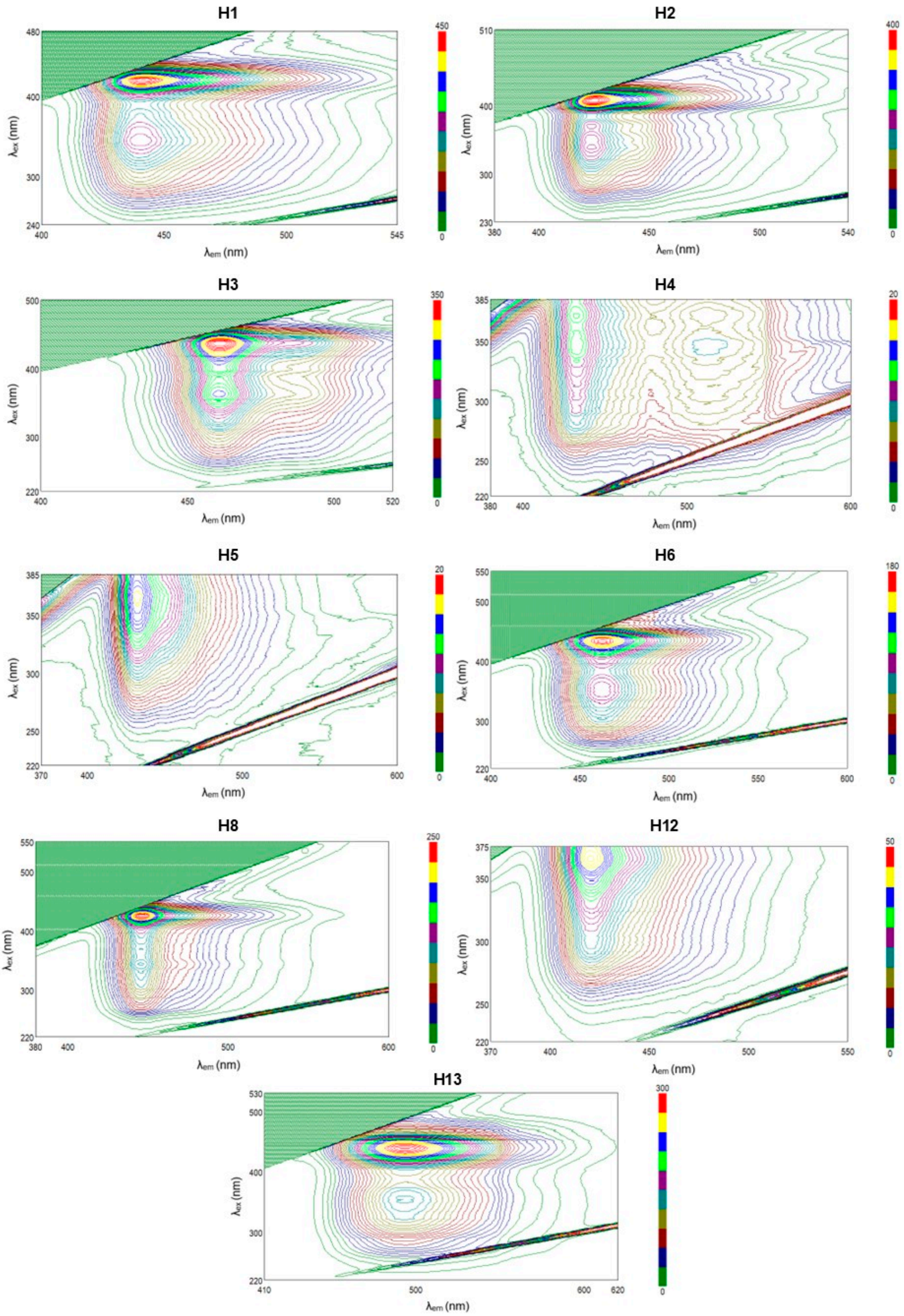

Figure S44. 3D-EEM fluorescence spectra of the phenylhydrazones. The fluorescence intensity colour code is displayed on the right side.

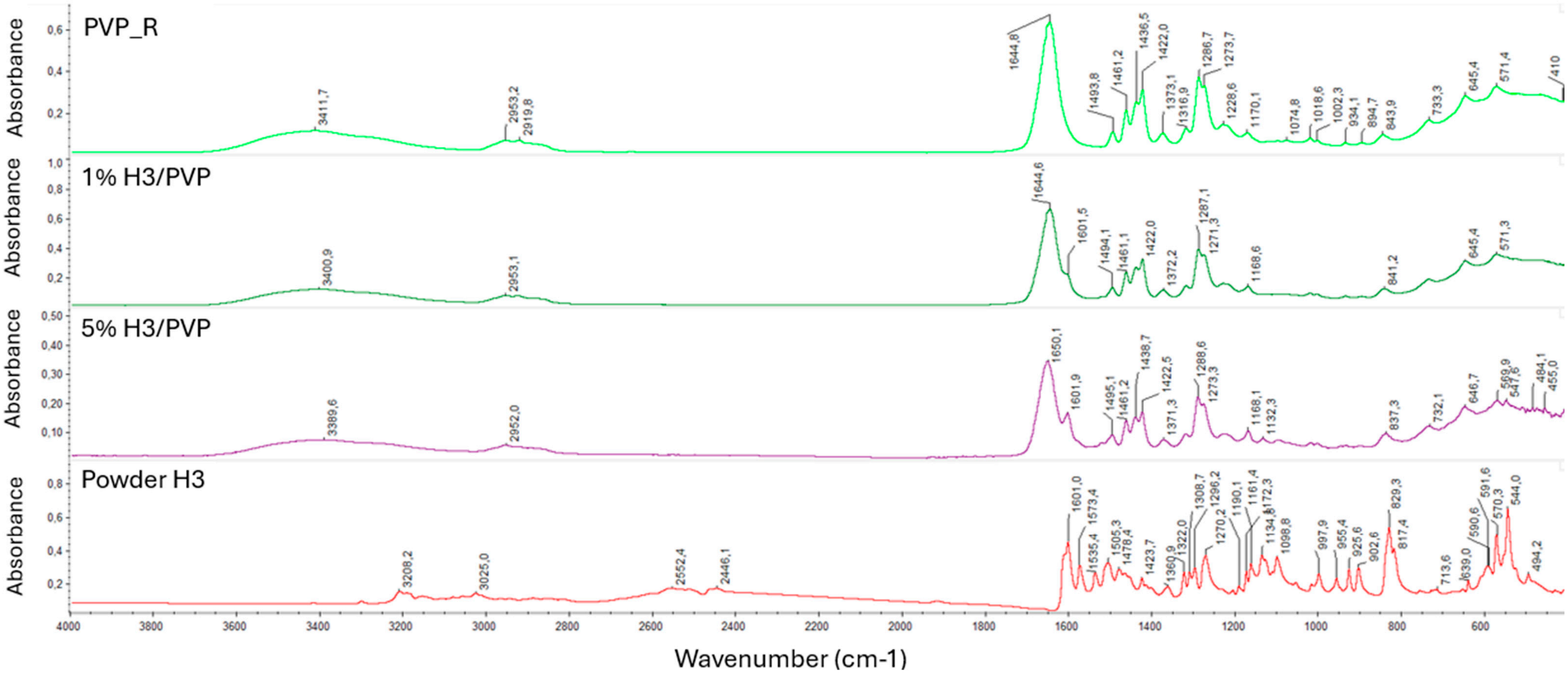

Figure S45. ATR-FTIR spectra of pure **H3** powder (red), 5% **H3**/PVP (purple), 1% **H3**/PVP (dark green), and the PVP_R (light green).

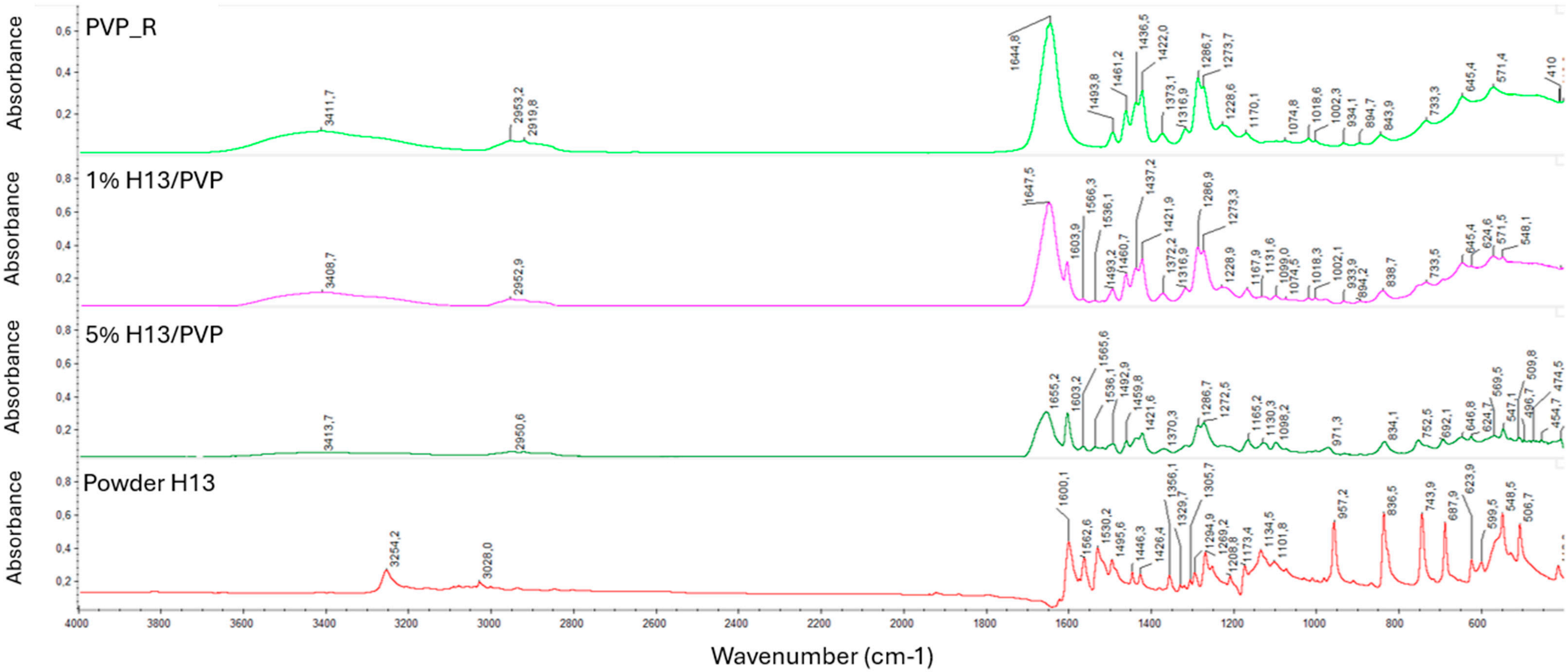

Figure S46. ATR-FTIR spectra of pure **H13** powder (red), 5% **H13**/PVP (purple), 1% **H13**/PVP (dark green), and the PVP_R (light green).

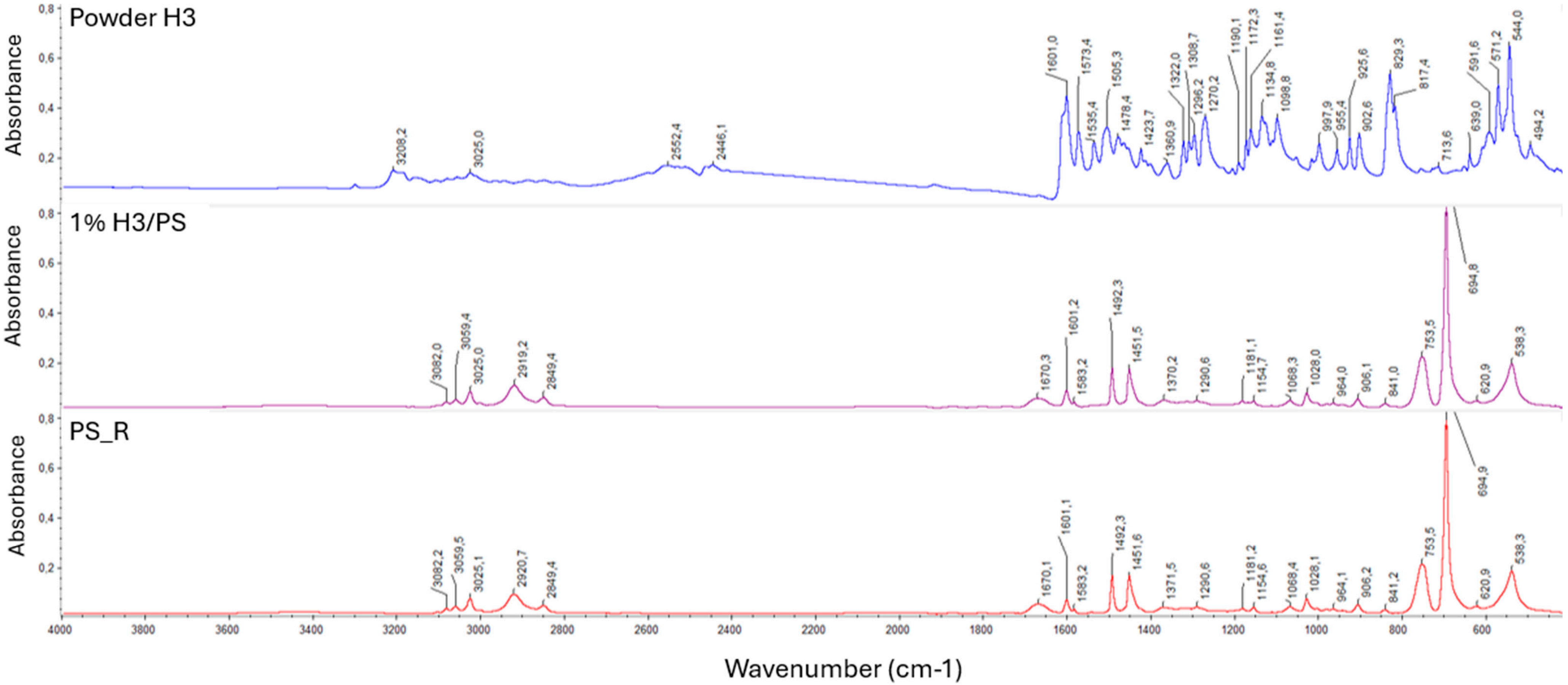

Figure S47. ATR-FTIR spectra of pure **H3** powder (blue), 1% **H3**/PS (purple), and the PS_R (red).

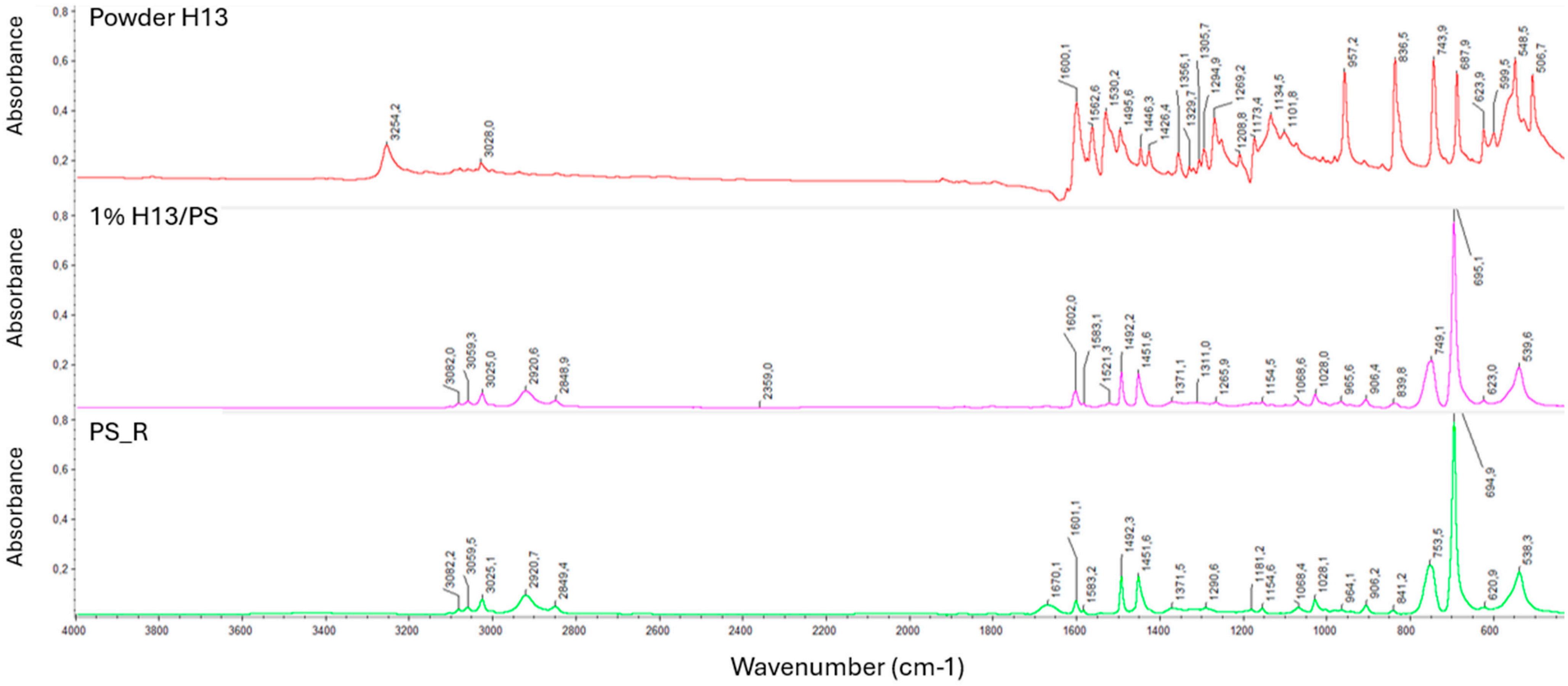

Figure S48. ATR-FTIR spectra of pure **H13** powder (blue), 1% **H13**/PS (purple), and the PS_R (red).

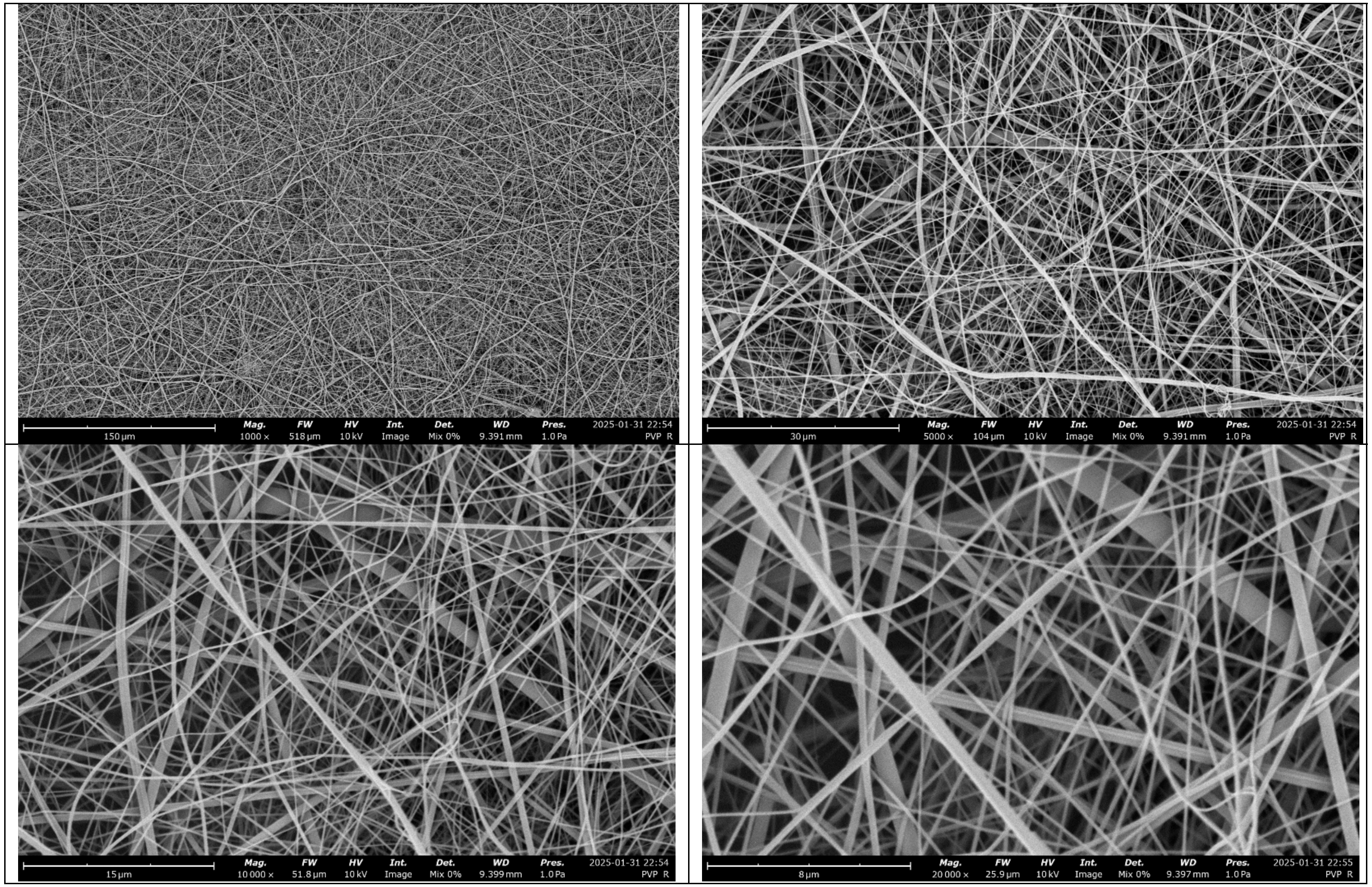

Figure S49. SEM images of the reference poly(N-vinylpyrrolidone) (PVP_R) material obtained by electrospinning, at different magnifications: 1000×, 5000×, 10,000×, 20,000×.

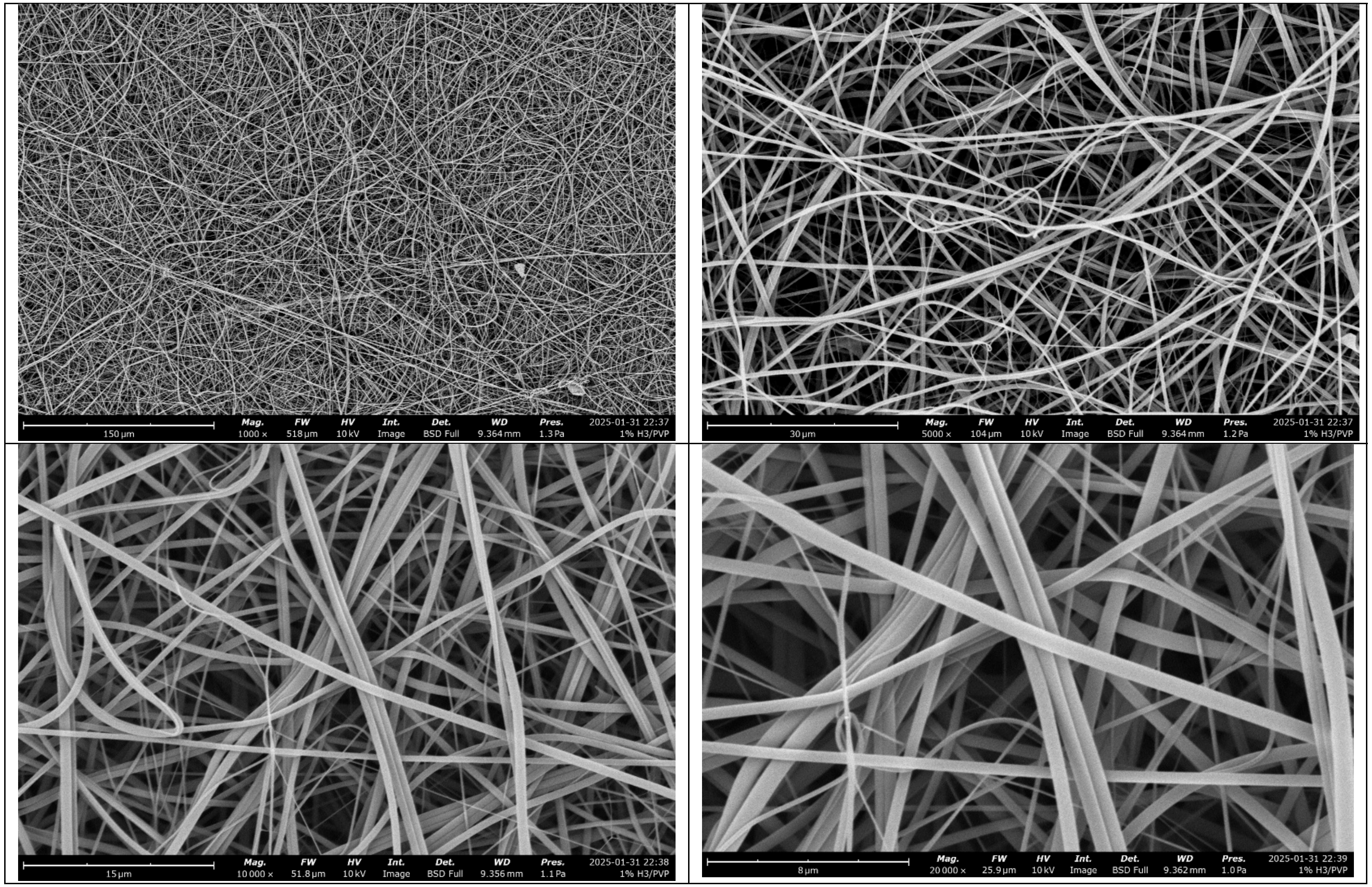

Figure S50. SEM images of the PVP10% material containing 1 wt.% of **H3** phenylhydrazone (1% **H3**/PVP) obtained by electrospinning, at different magnifications: 1000×, 5000×, 10,000×, 20,000×.

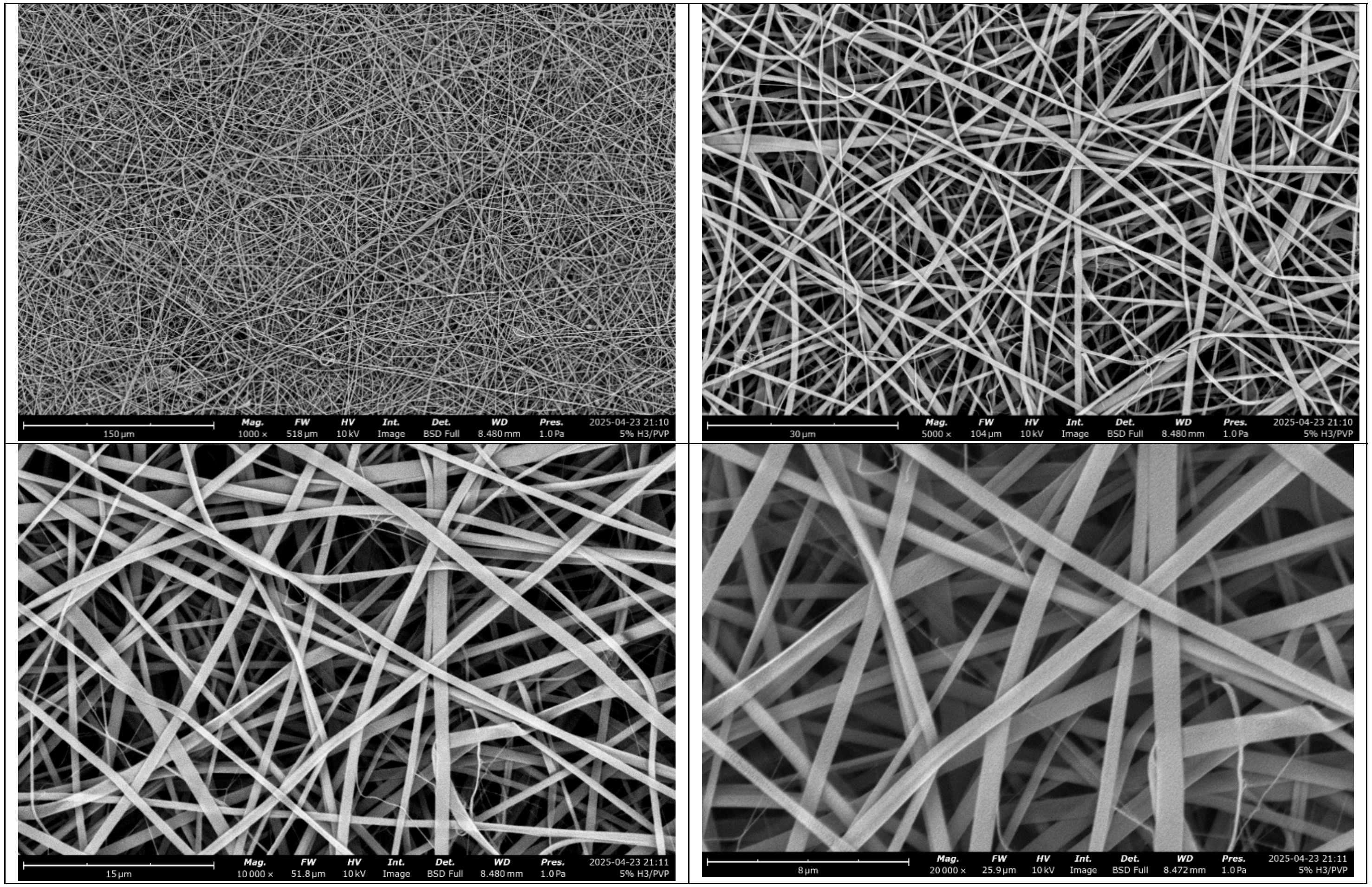

Figure S51. SEM images of the PVP10% material containing 5 wt.% of **H3** phenylhydrazone (5% **H3**/PVP) obtained by electrospinning, at different magnifications: 1000×, 5000×, 10,000×, 20,000×.

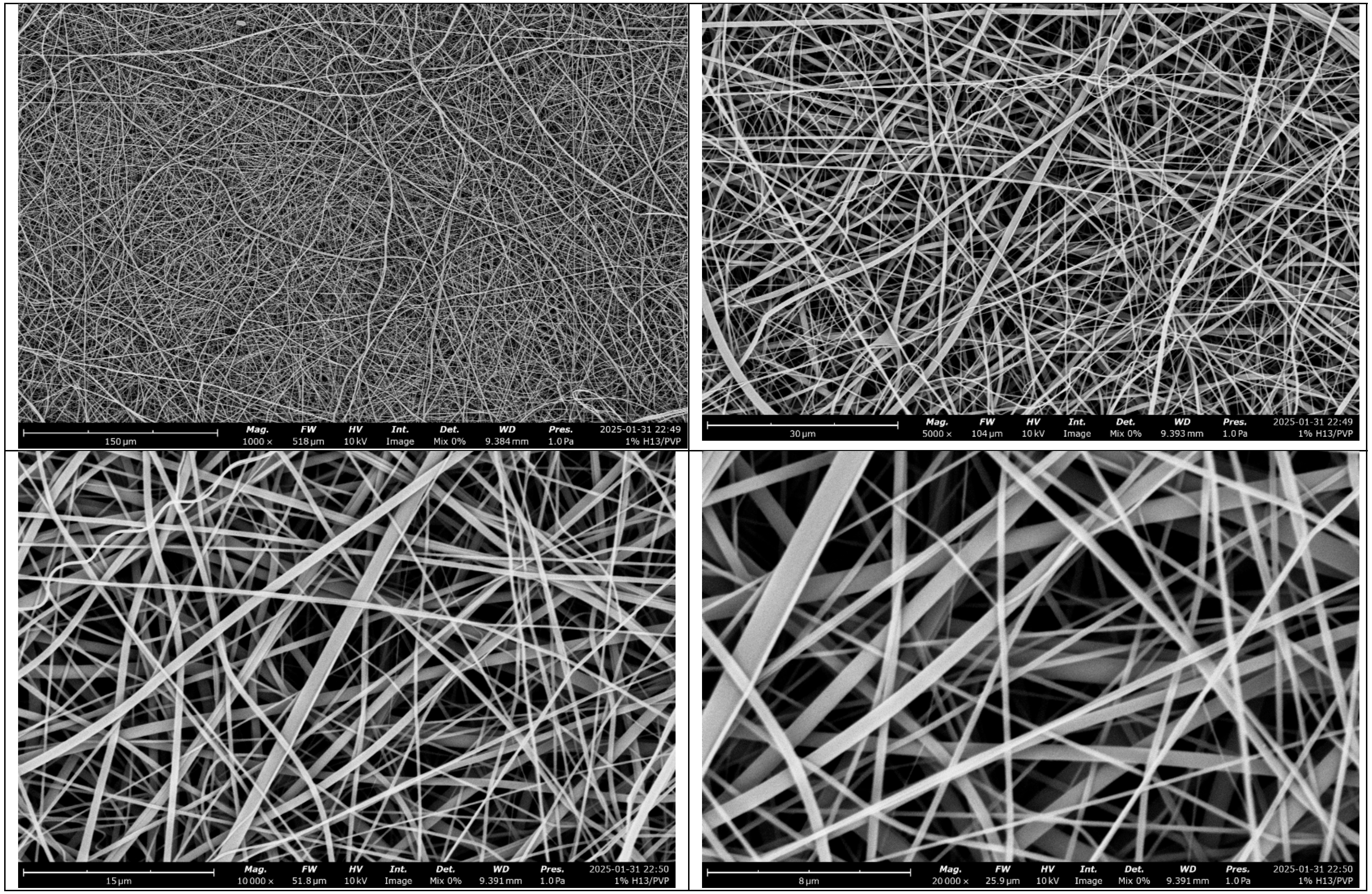

Figure S52. SEM images of the PVP10% material containing 1 wt.% of **H13** phenylhydrazone (1% **H13**/PVP) obtained by electrospinning, at different magnifications: 1000×, 5000×, 10,000×, 20,000×.

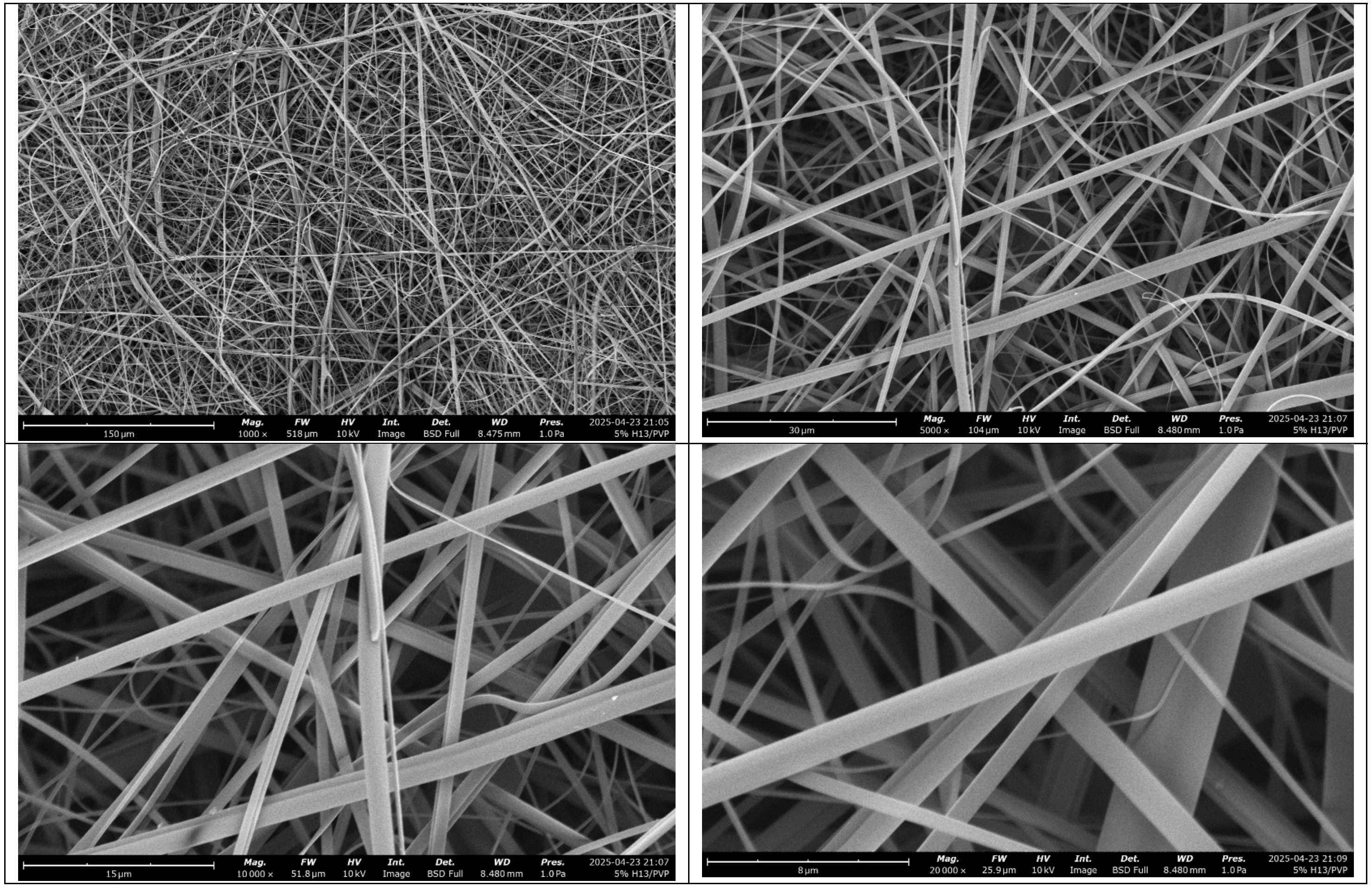

Figure S53. SEM images of the PVP10% material containing 5 wt.% of **H13** phenylhydrazone (5% **H13**/PVP) obtained by electrospinning, at different magnifications: 1000×, 5000×, 10,000×, 20,000×.

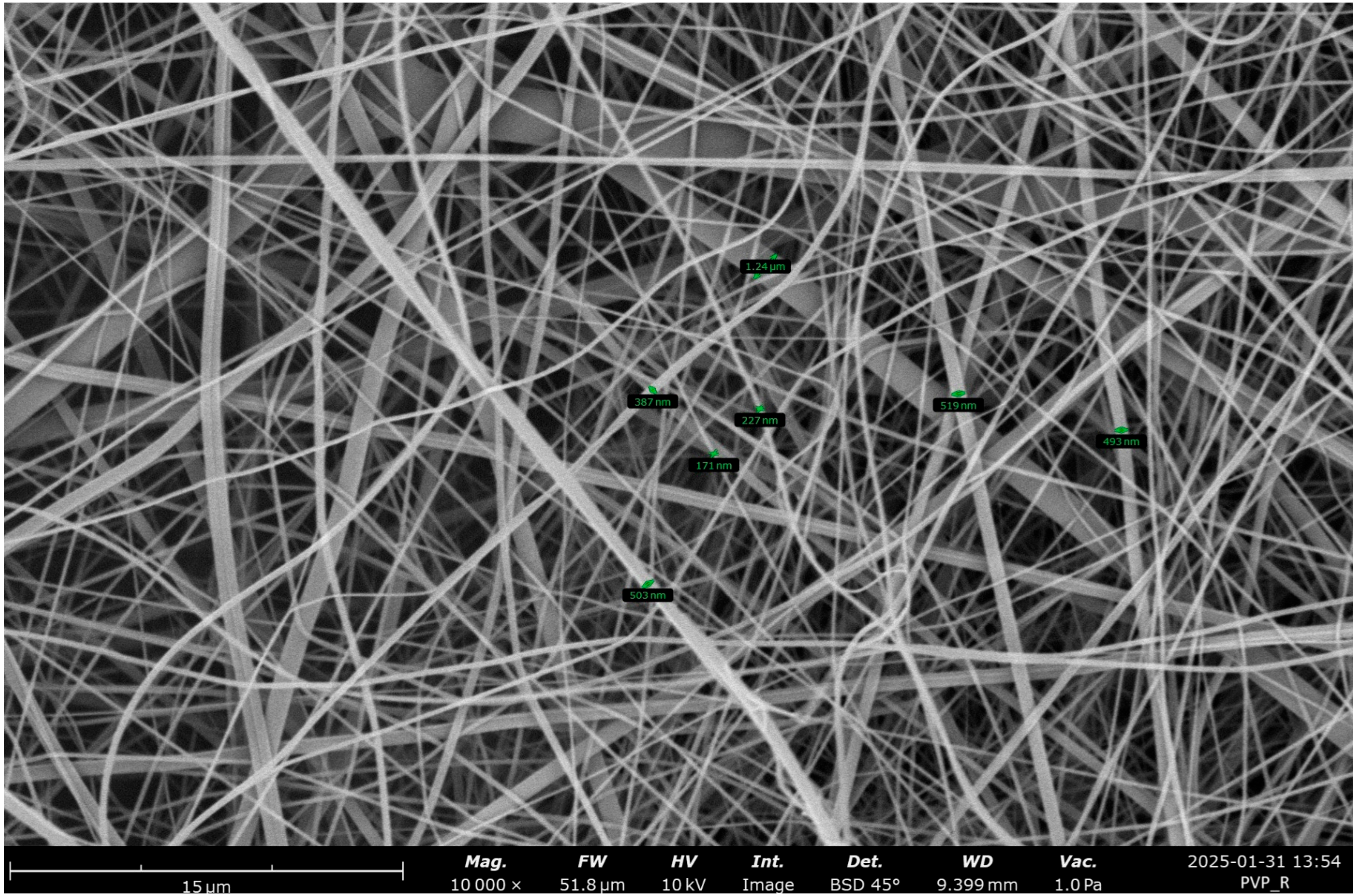

Figure S54. SEM image of the reference poly(N-vinylpyrrolidone) (PVP_R) material obtained by electrospinning, showing the measurement of fiber diameters at 10,000× magnification.

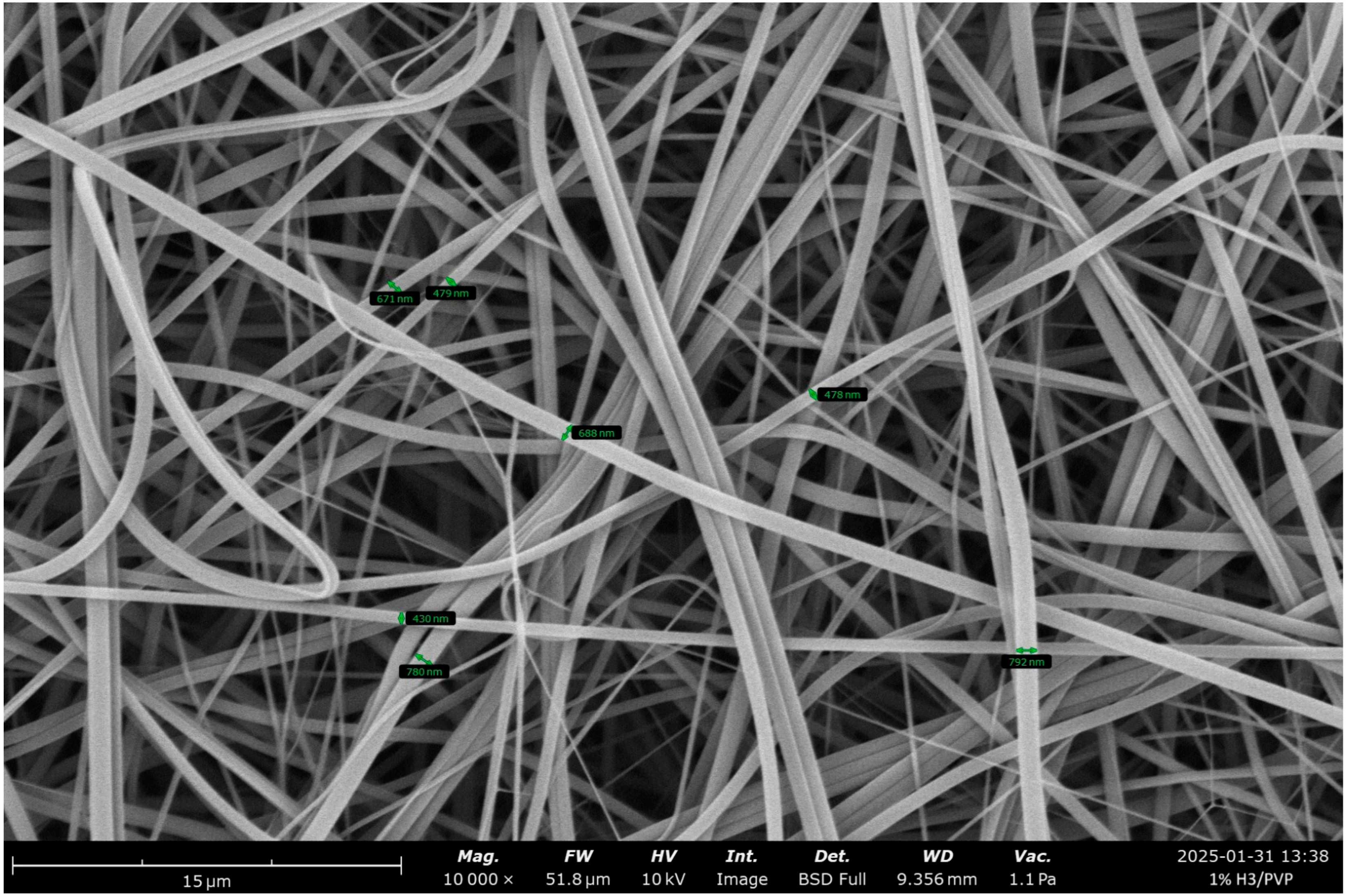

Figure S55. SEM image of the PVP material containing 1 wt.% of **H3** phenylhydrazone (1% **H3**/PVP) obtained by electrospinning, showing the measurement of fiber diameters at 10,000× magnification.

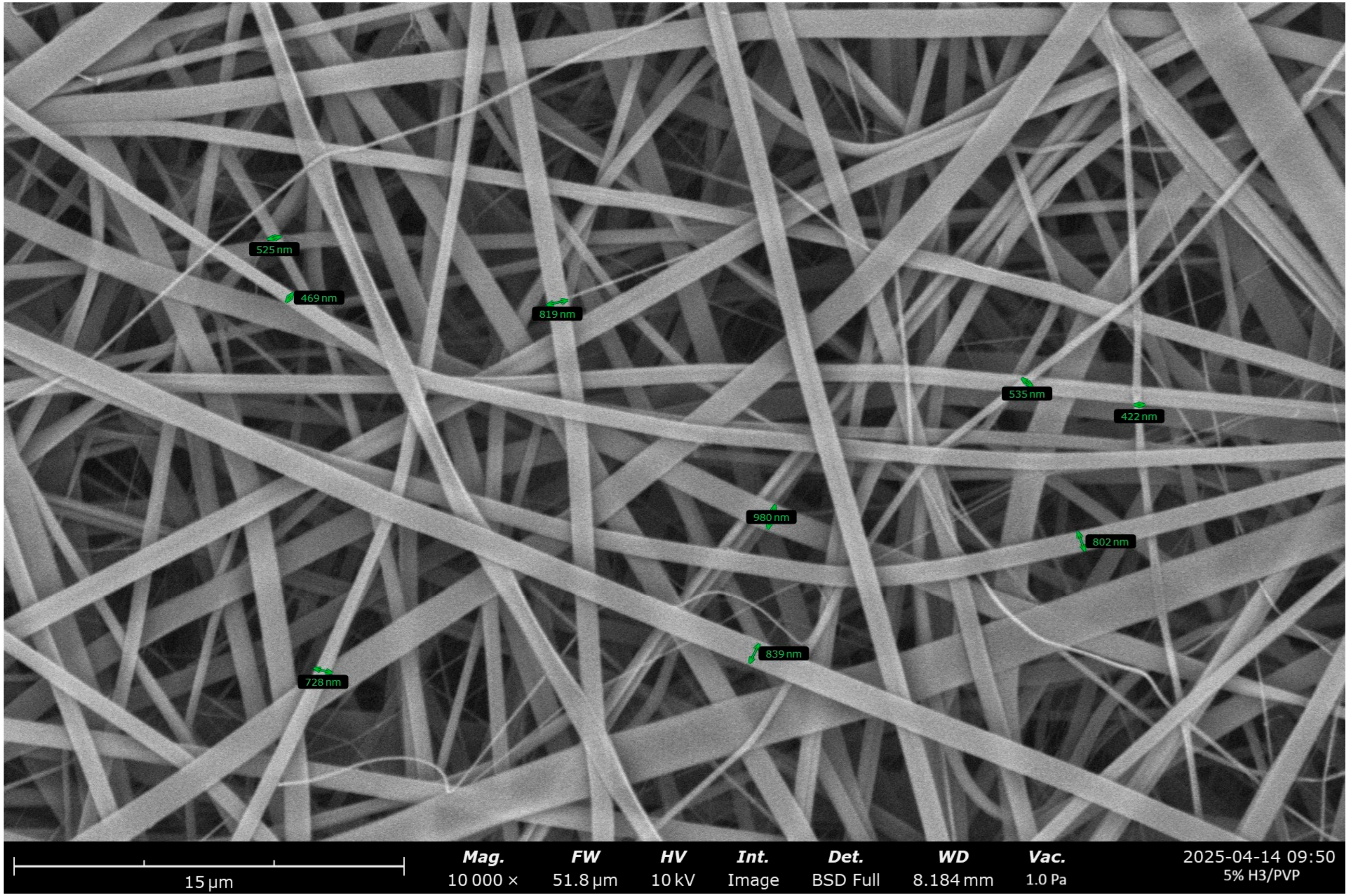

Figure S56. SEM image of the PVP material containing 5 wt.% of **H3** phenylhydrazone (5% **H3**/PVP) obtained by electrospinning, showing the measurement of fiber diameters at 10,000× magnification.

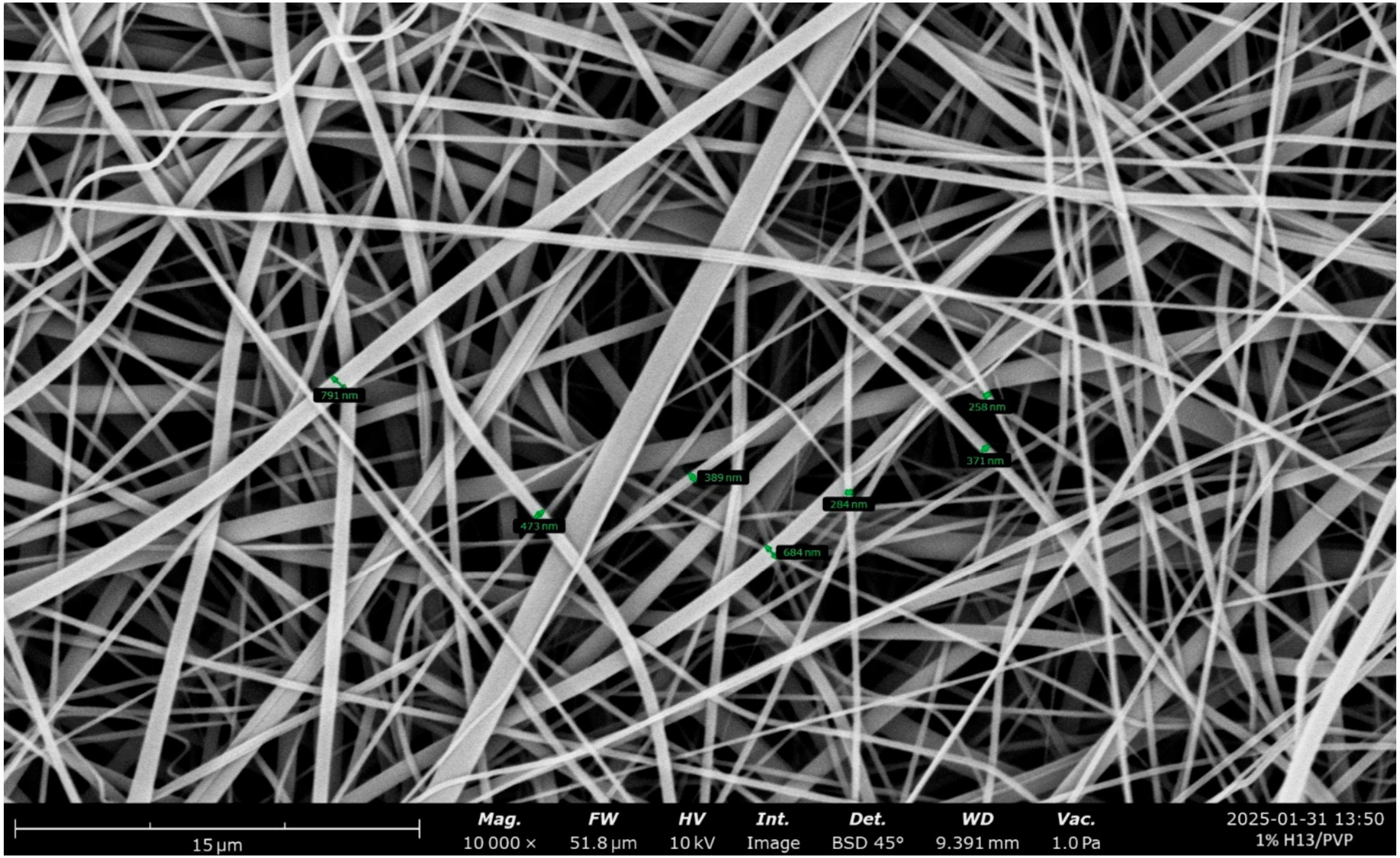

Figure S57. SEM image of the PVP material containing 1 wt.% of **H13** phenylhydrazone (1% **H13**/PVP) obtained by electrospinning, showing the measurement of fiber diameters at 10,000× magnification.

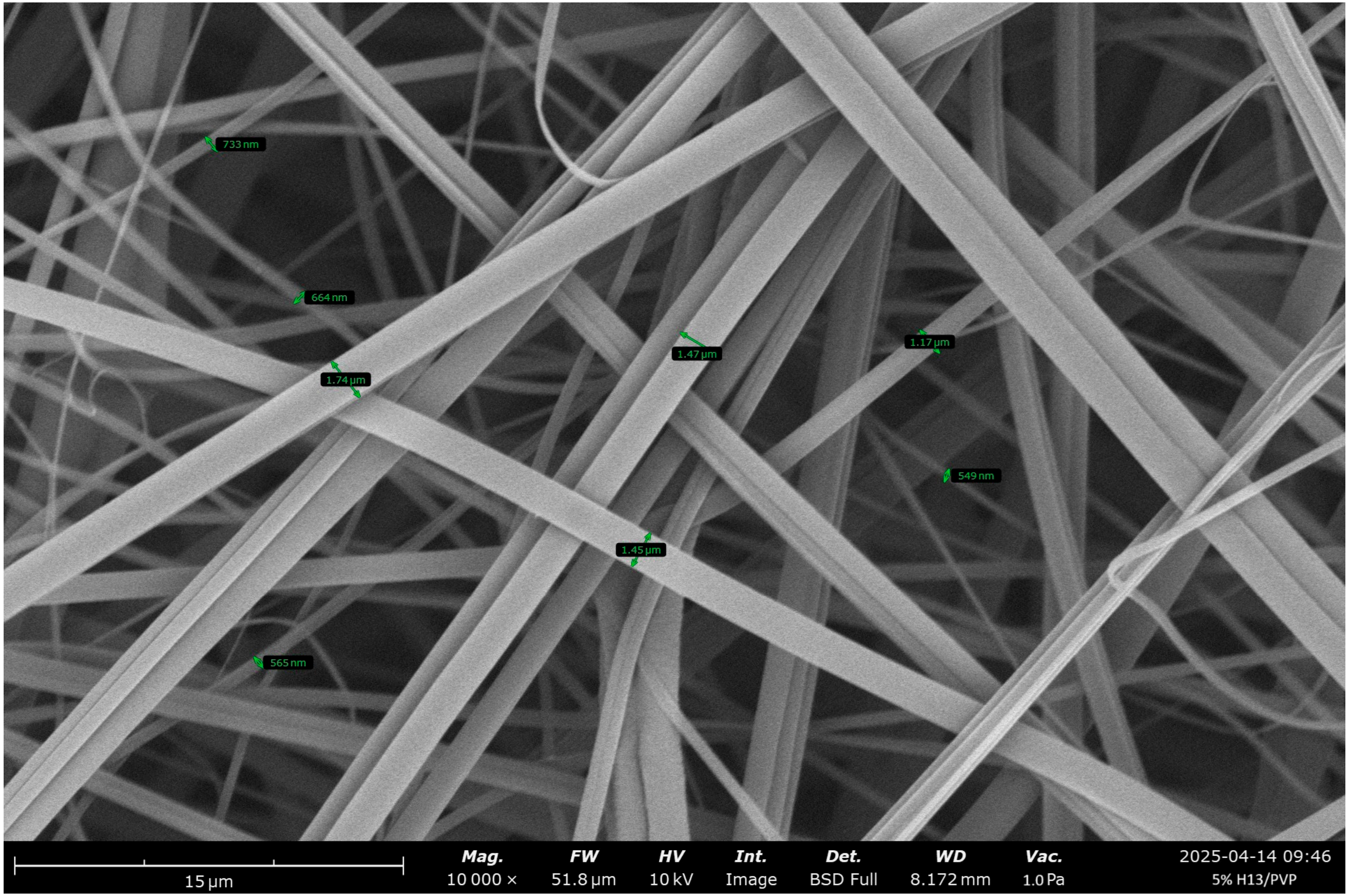

Figure S58. SEM image of the PVP material containing 5 wt.% of **H13** phenylhydrazone (5% **H13**/PVP) obtained by electrospinning, showing the measurement of fiber diameters at 10,000× magnification.

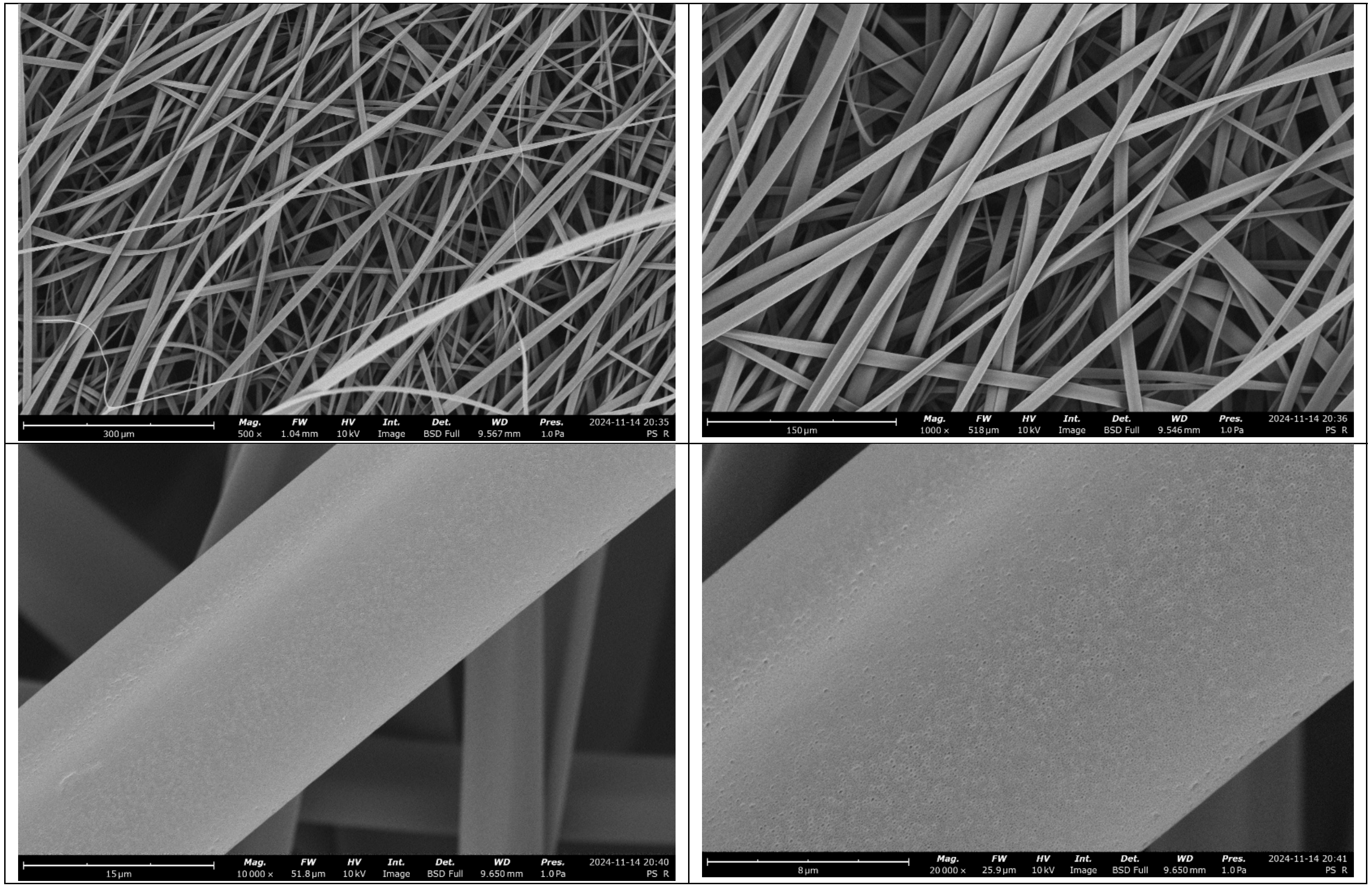

Figure S59. SEM images of the reference polystyrene (PS_R) material obtained by electrospinning, at different magnifications: 500×, 1000×, 10,000×, 20,000×.

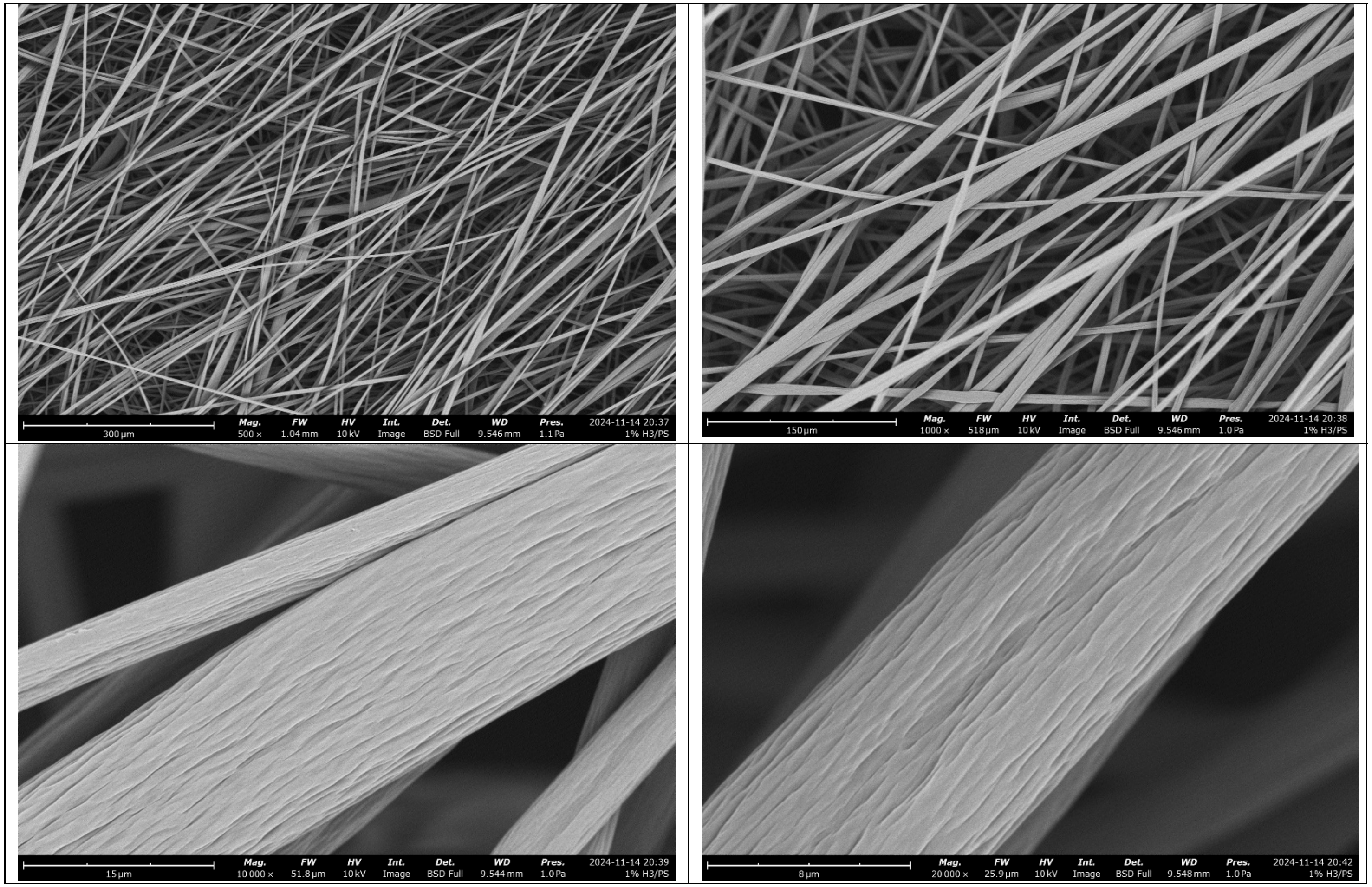

Figure S60. SEM images of the PS material containing 1 wt.% of **H3** phenylhydrazone (1% **H3**/PS) obtained by electrospinning, at different magnifications: 500×, 1000×, 10,000×, 20,000×.

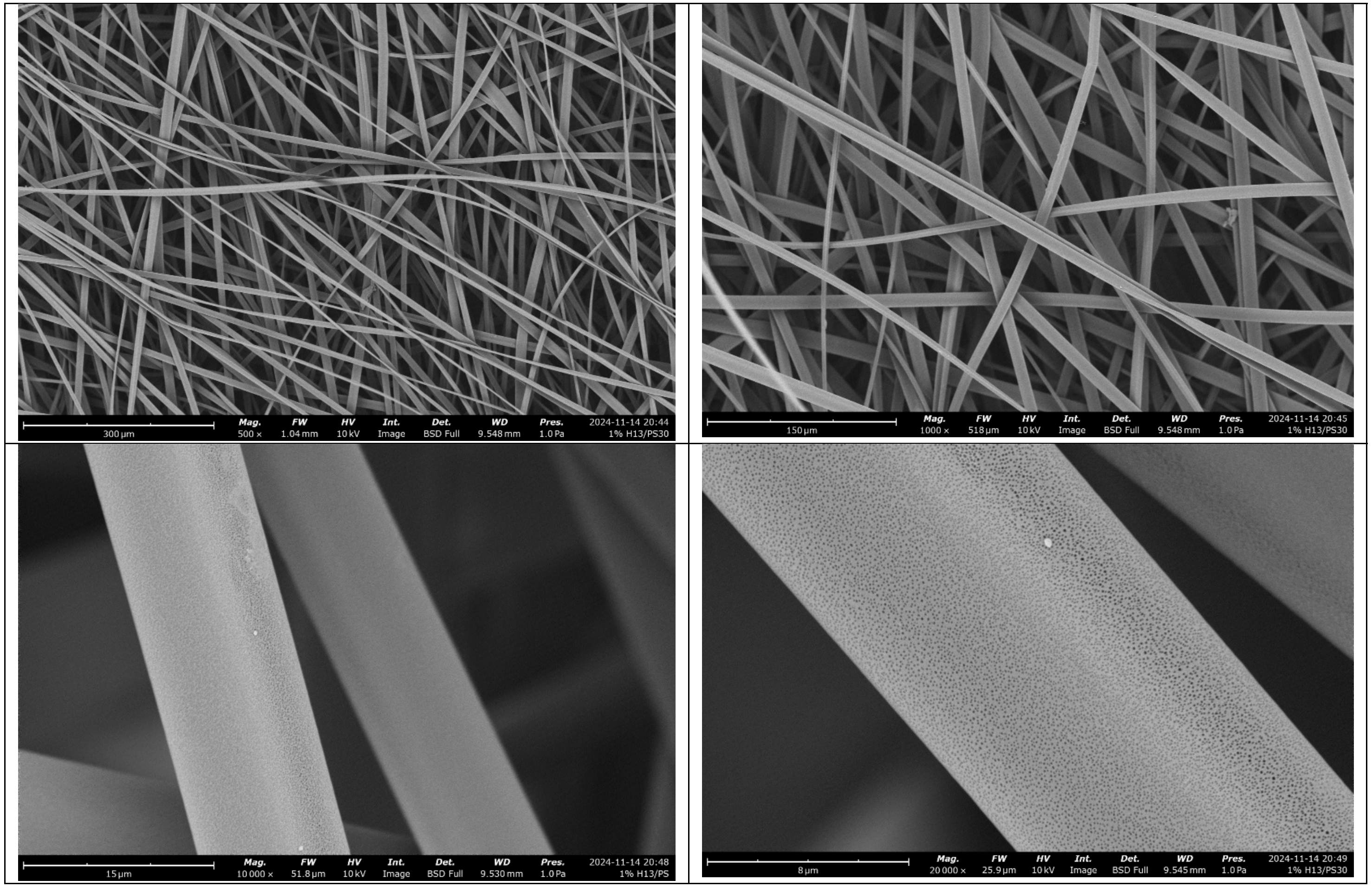

Figure S61. SEM images of the PS material containing 1 wt.% of **H13** phenylhydrazone (1% **H13**/PS) obtained by electrospinning, at different magnifications: 500×, 1000×, 10,000×, 20,000×.

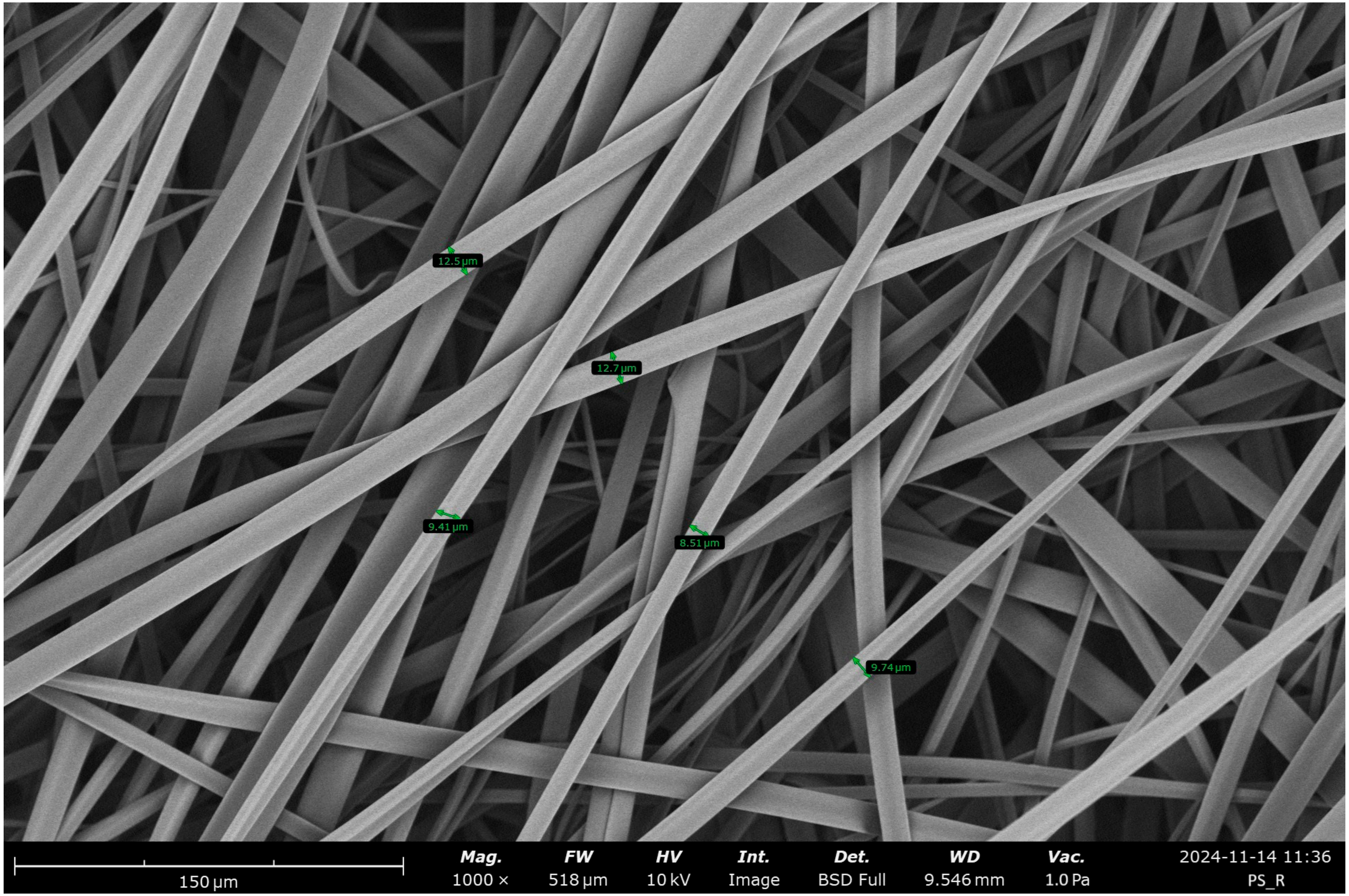

Figure S62. SEM image of the reference polystyrene (PS_R) material obtained by electrospinning, showing the measurement of fiber diameters at 1000× magnification.

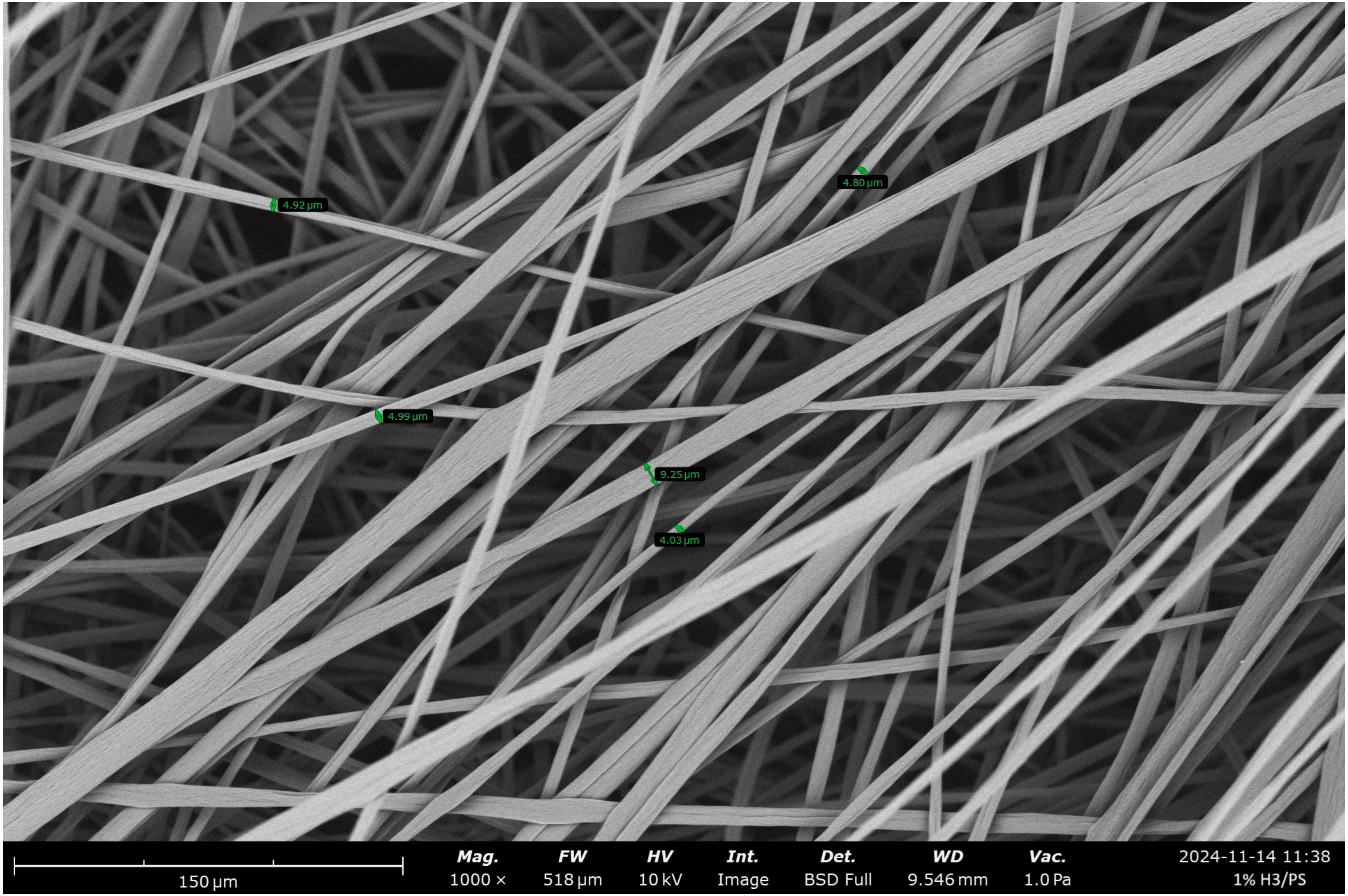

Figure S63. SEM image of the PS30% material containing 1 wt.% of **H3** phenylhydrazone (1% **H3**/PS) obtained by electrospinning, showing the measurement of fiber diameters at 1000× magnification.

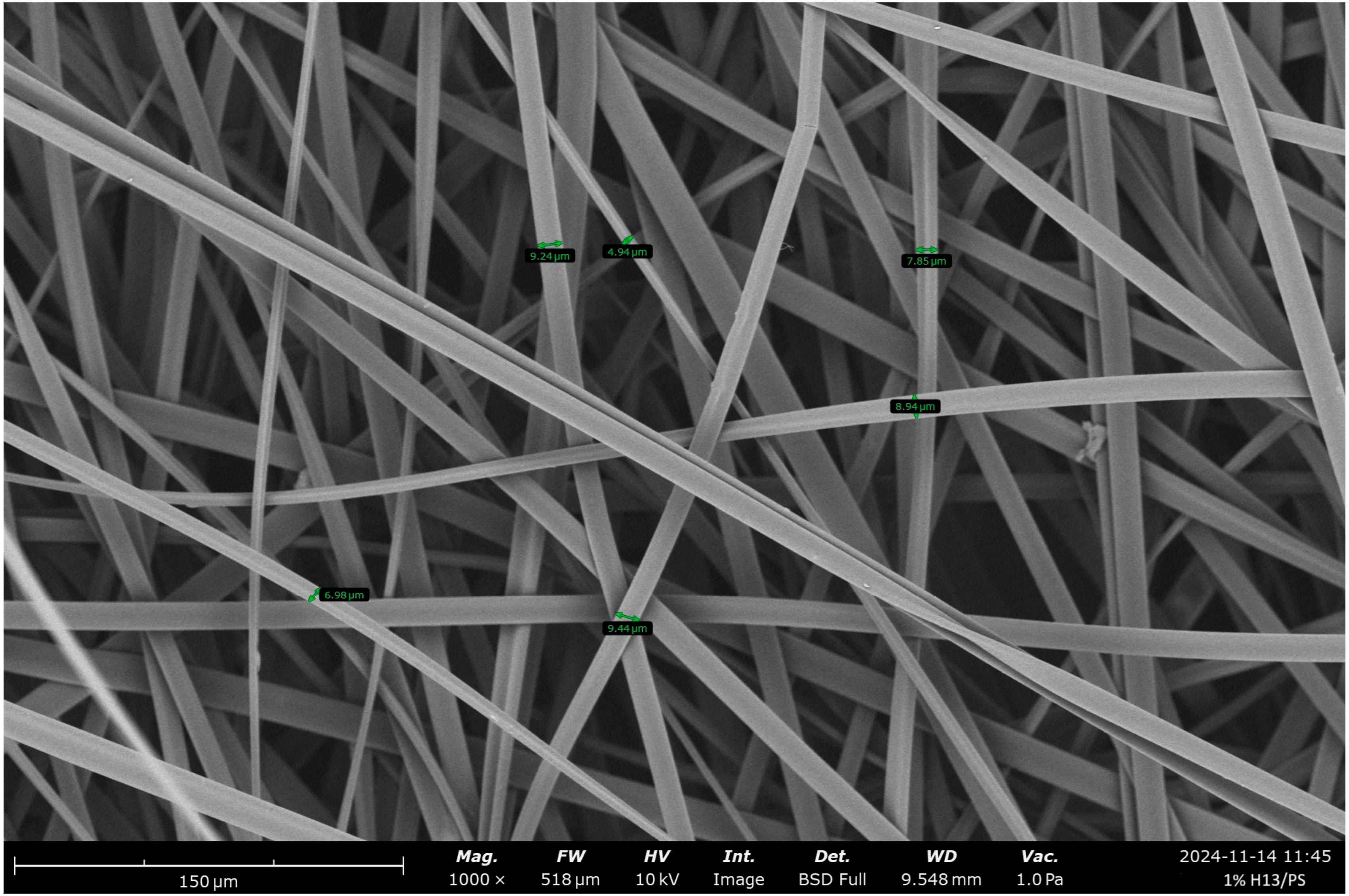

Figure S64. SEM image of the PS material containing 1 wt.% of **H13** phenylhydrazone (1% **H13**/PS) obtained by electrospinning, showing the measurement of fiber diameters at 1000× magnification.

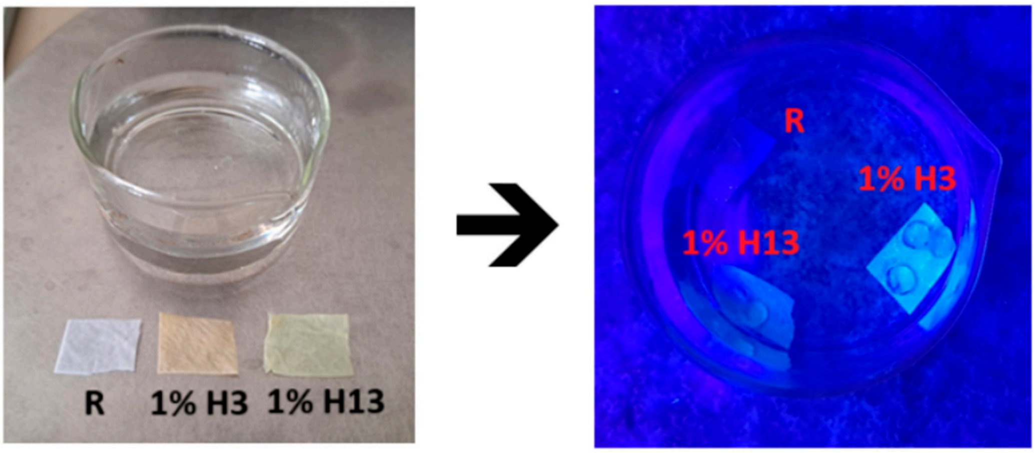

Figure S65. Water stability test of polystyrene (PS) based materials. **Left:** Appearance of the reference (R) sample and materials containing 1 wt.% of H3 and H13 phenylhydrazones before immersion in water. **Right:** The same samples after 24-hour exposure to water, photographed under UV illumination (365 nm).

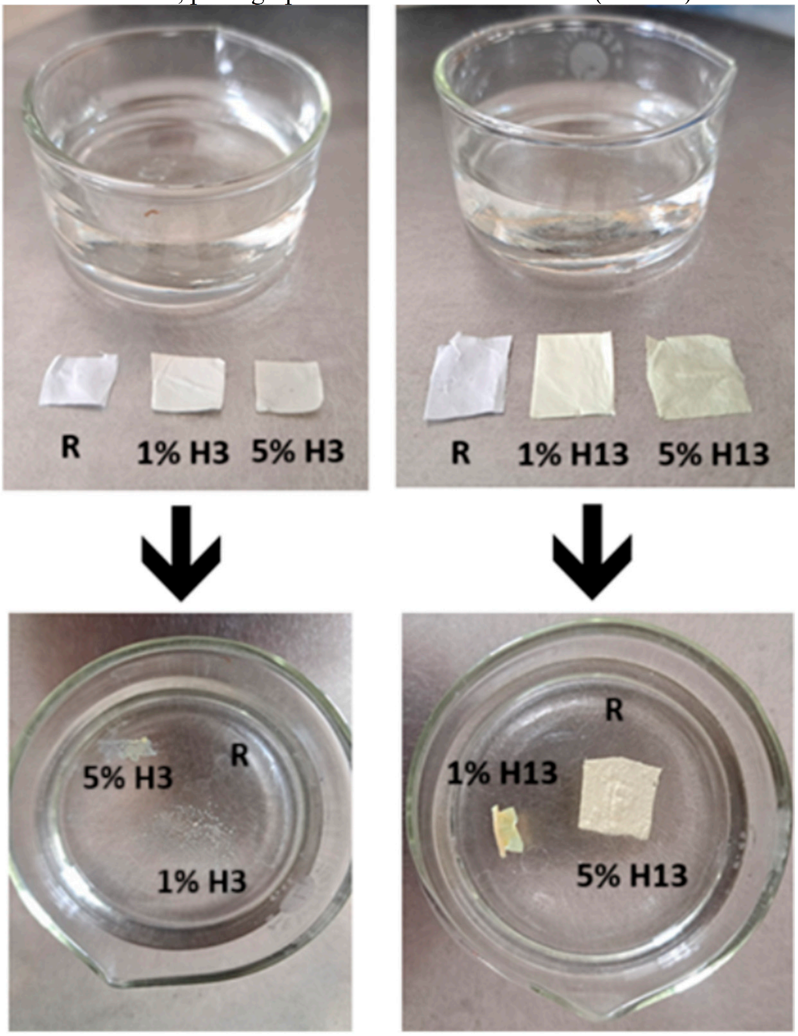

Figure S66. Water stability test of poly(N-vinylpyrrolidone) (PVP) based materials. **Top:** Appearance of the reference (R) sample and materials containing H3 (1% and 5%) and H13 (1% and 5%) phenylhydrazones before immersion in water. **Bottom:** The same samples after 24-hour exposure to water.

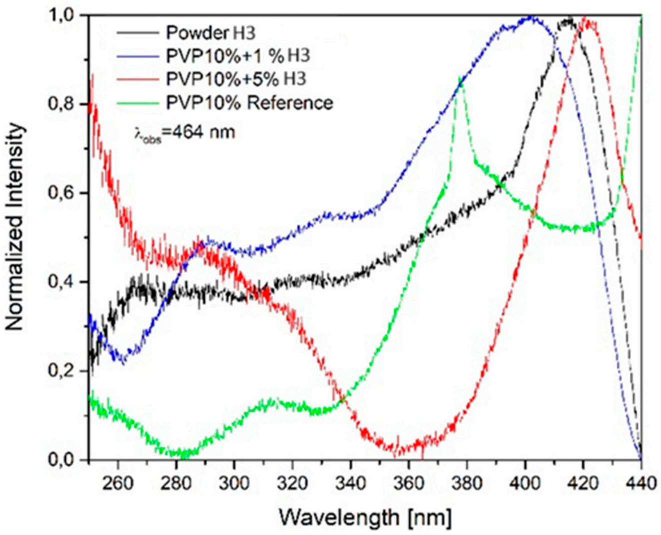

Figure S67. Excitation spectrum.

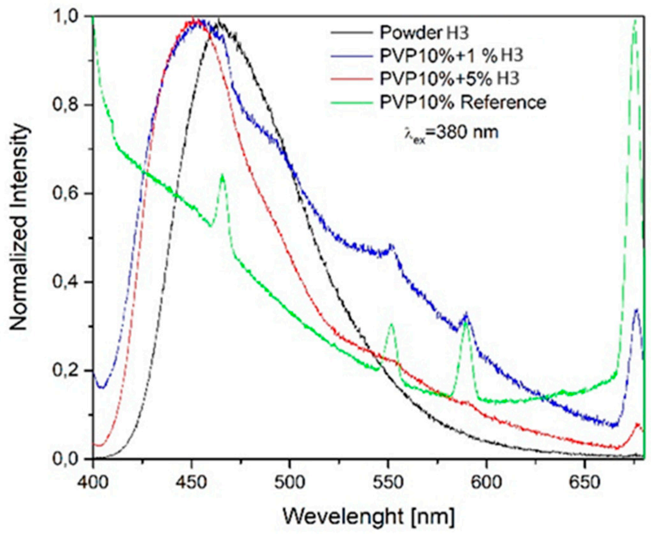

Figure S68. Emission spectrum excited λ=380nm.

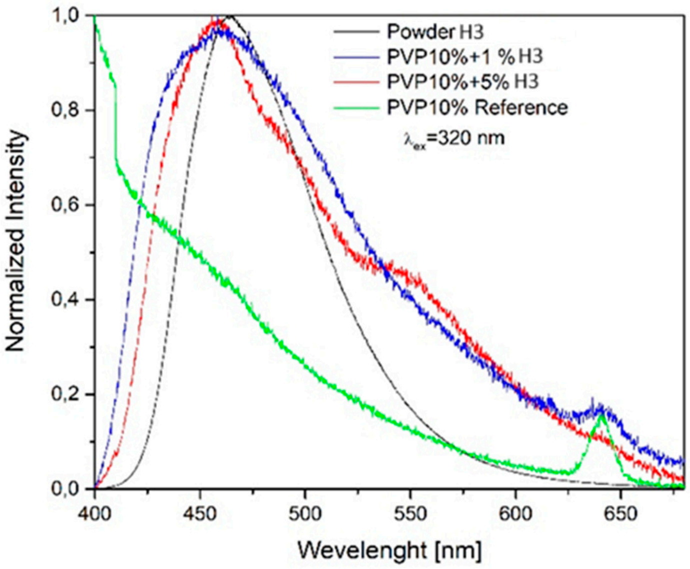

Figure S69. Emission spectrum excited λ=320nm.

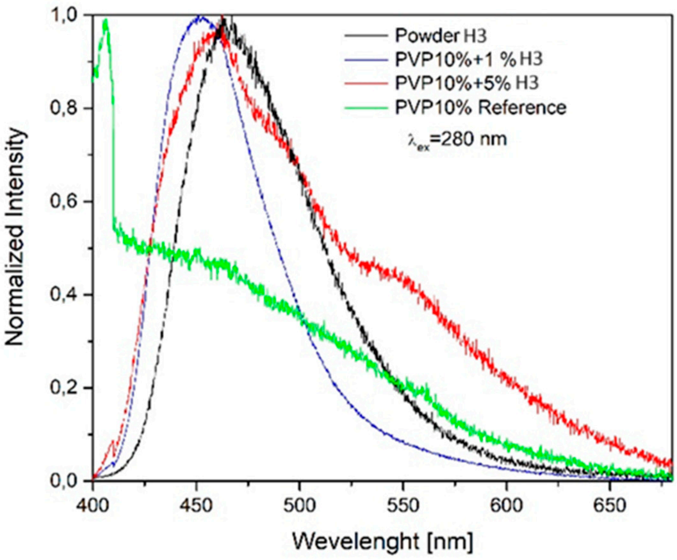

Figure S70. Emission spectrum excited λ=280nm.

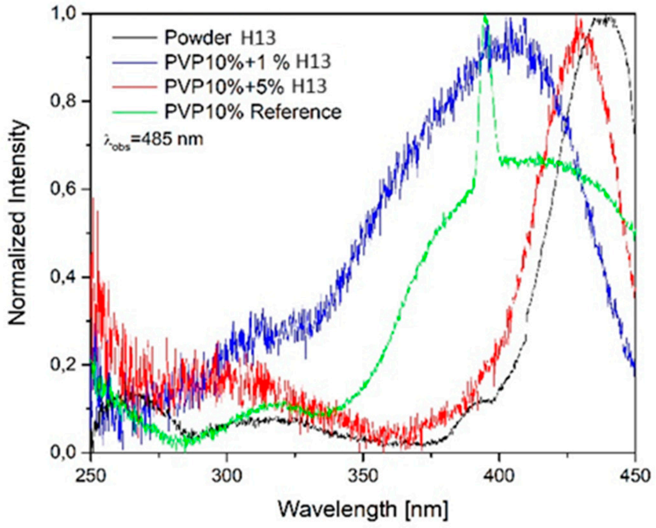

Figure S71. Excitation spectrum

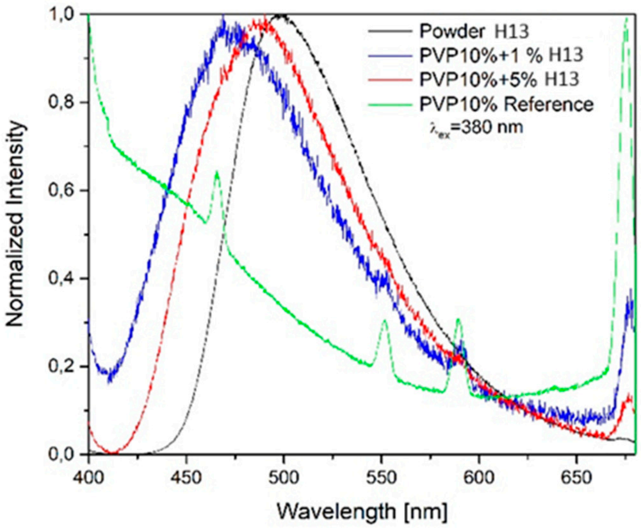

Figure S72. Emission spectrum excited λ=380nm

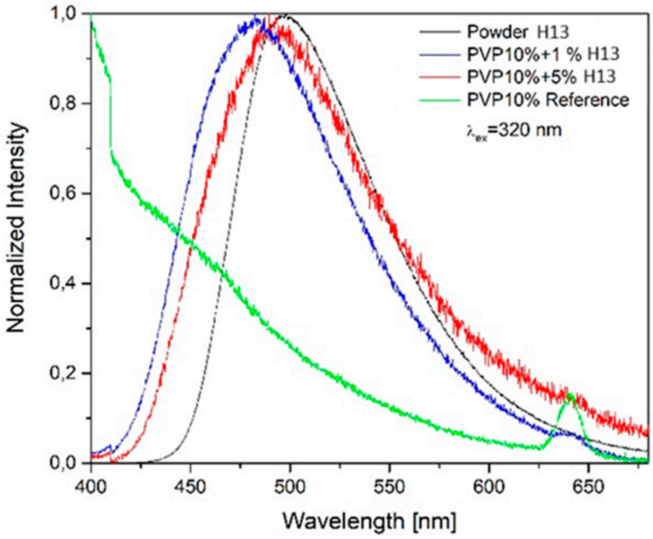

Figure S73. Emission spectrum excited λ=320nm

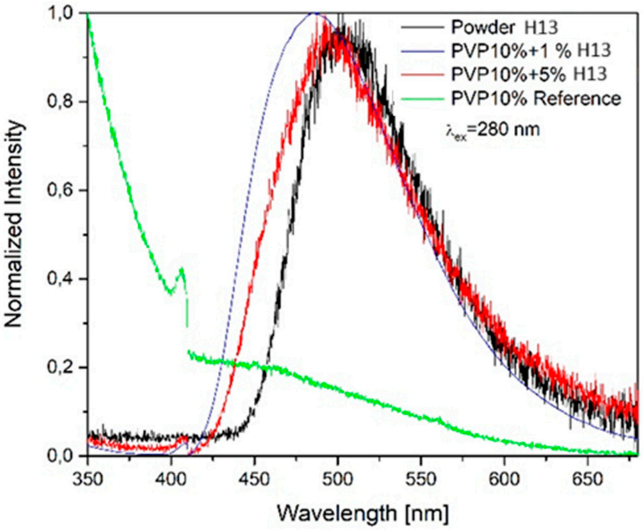

Figure S74. Emission spectrum excited λ=280nm

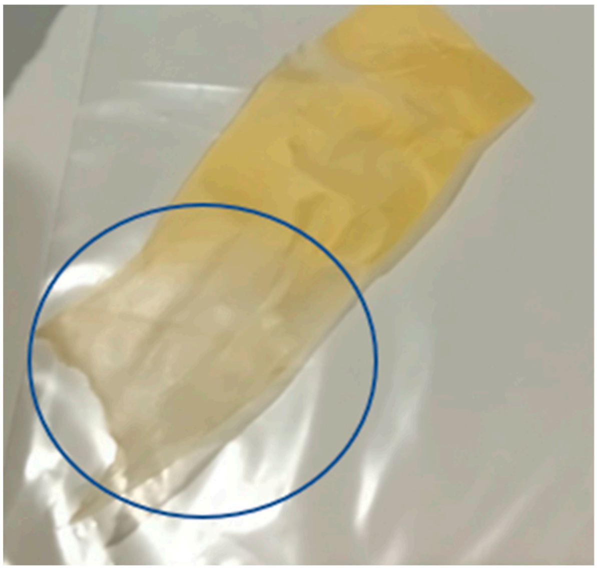

Figure S75. The PS material containing 1 wt.% **H3** phenylhydrazone, melted at 100°C.

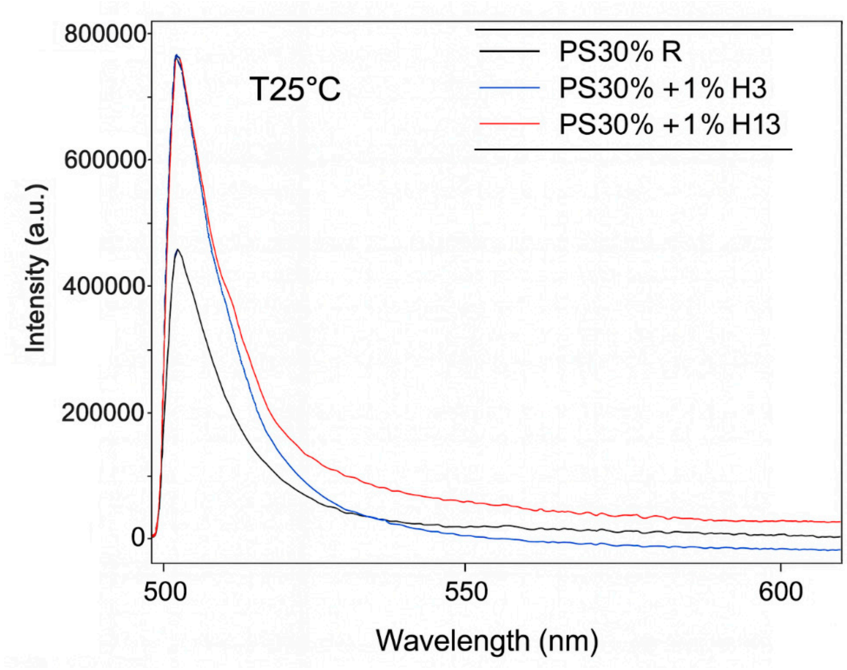

Figure S76. Polystyrene samples at room temperature, excited with a 460 nm LED.

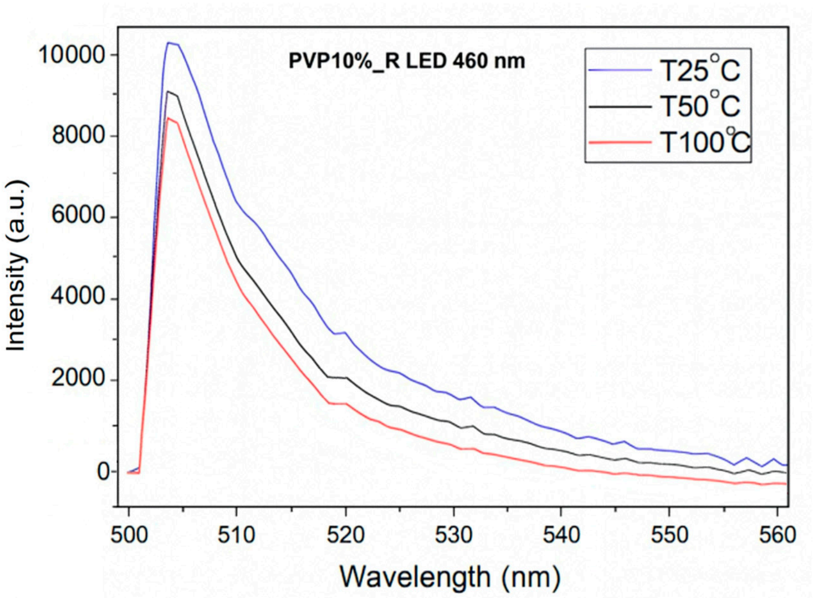

Figure S77. Temperature-dependent emission spectra of the PVP_R recorded at 25°C, 50°C, and 100°C. The excitation wavelength was 460 nm.

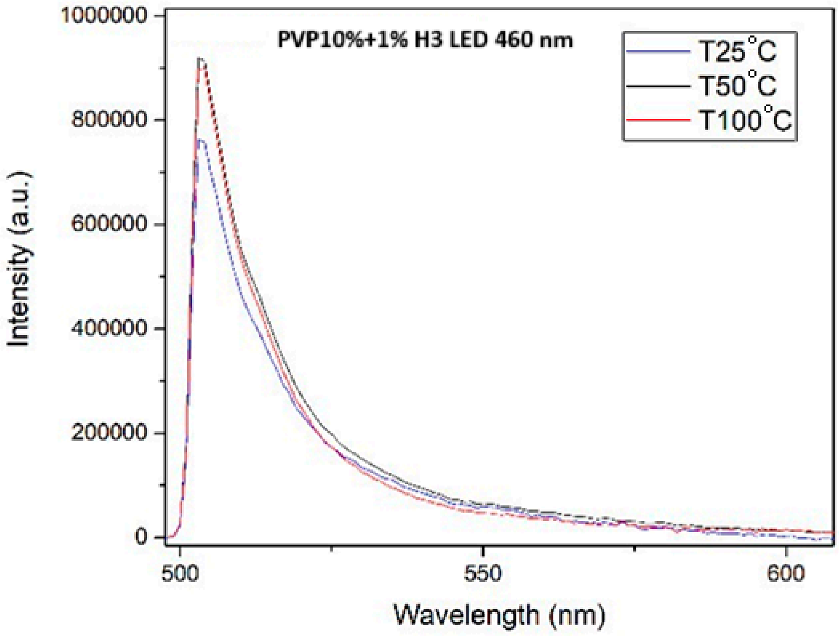

Figure S78. Temperature-dependent emission spectra of the 1% **H3**/PVP recorded at 25°C, 50°C, and 100°C. The excitation wavelength was 460 nm.

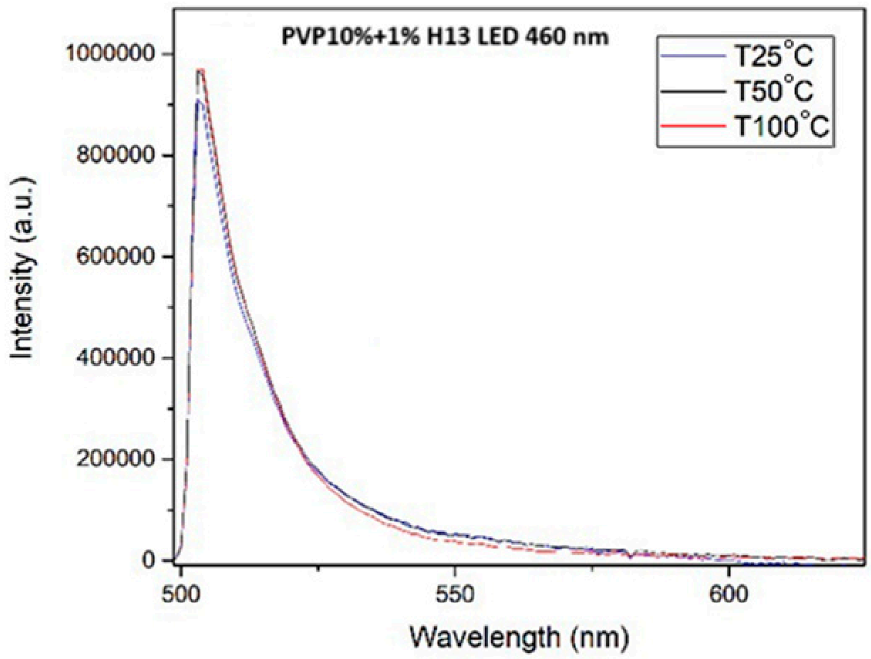

Figure S79. Temperature-dependent emission spectra of the 1% **H13**/PVP recorded at 25°C, 50°C, and 100°C. The excitation wavelength was 460 nm.

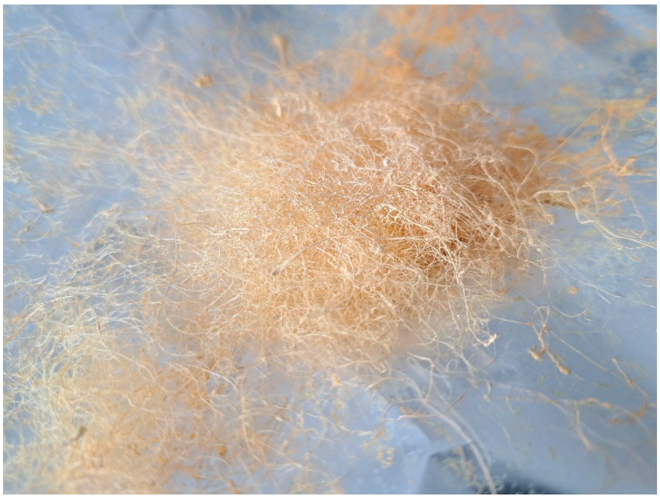

Figure S80. Material obtained from the electrospinning of a PS solution with 5 wt.% phenylhydrazone.

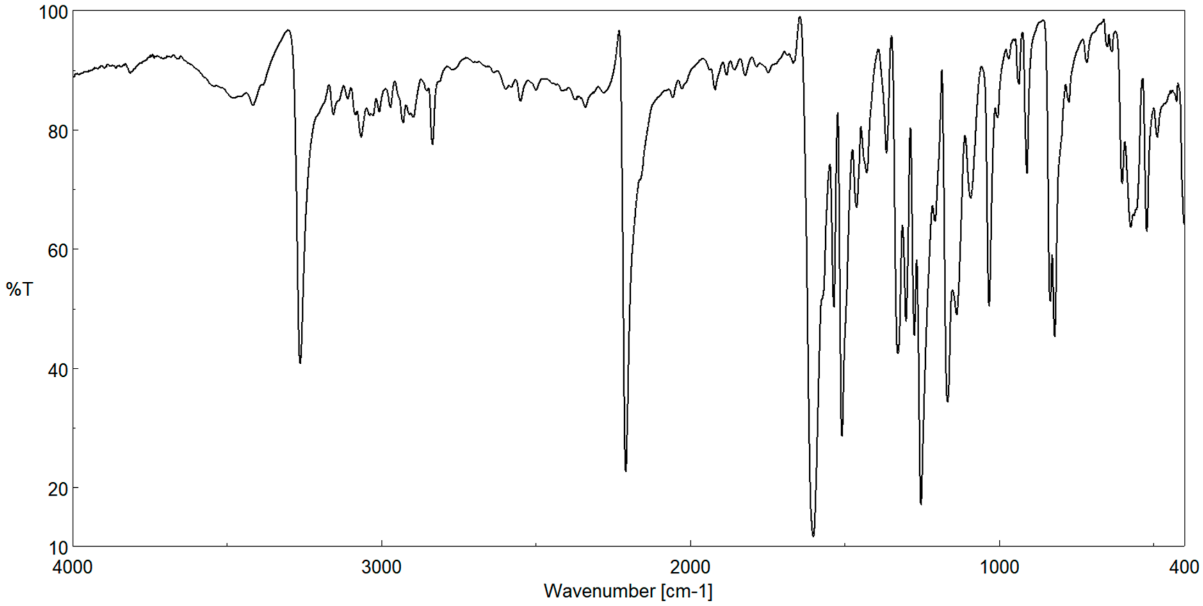

Figure S81. IR spectrum of (E)-4-(2-(4-methoxybenzylidene)hydrazineyl)benzonitrile (H1).

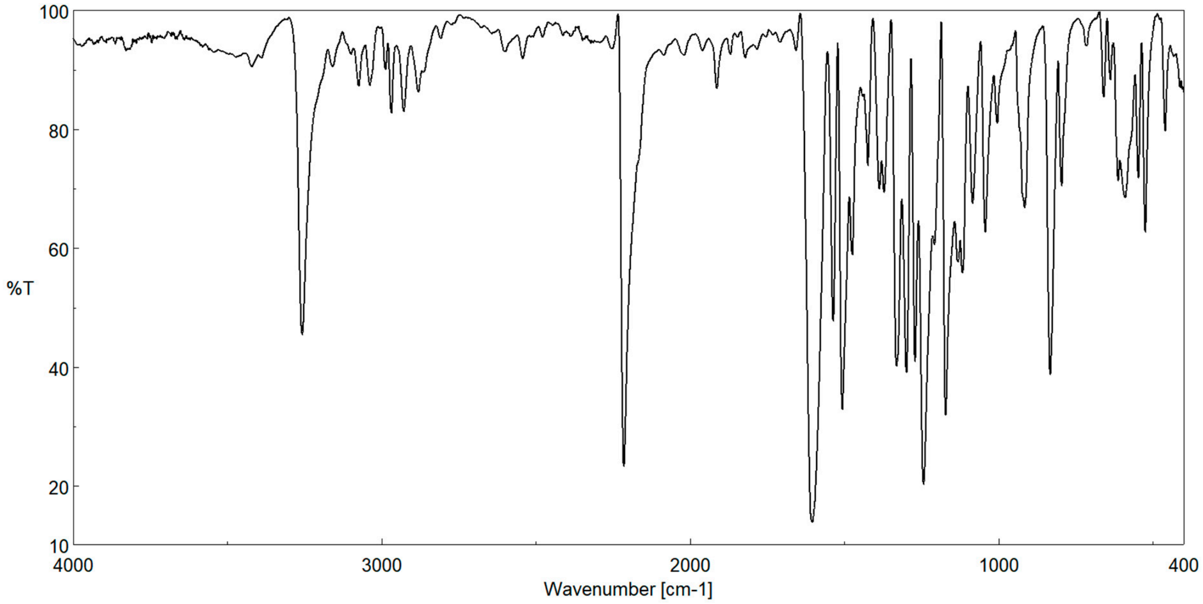

Figure S82. IR spectrum of (E)-4-(2-(4-ethoxybenzylidene)hydrazineyl)benzonitrile (H2).

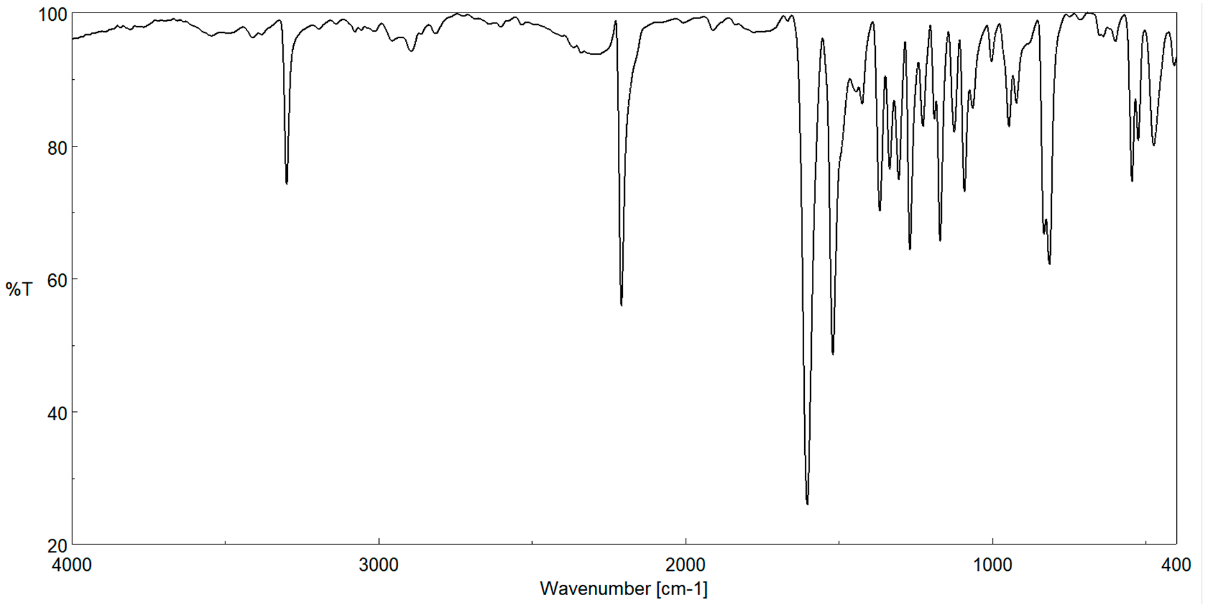

Figure S83. IR spectrum of (E)-4-(2-(4-(dimethylamino)benzylidene)hydrazineyl)benzonitrile (H3).

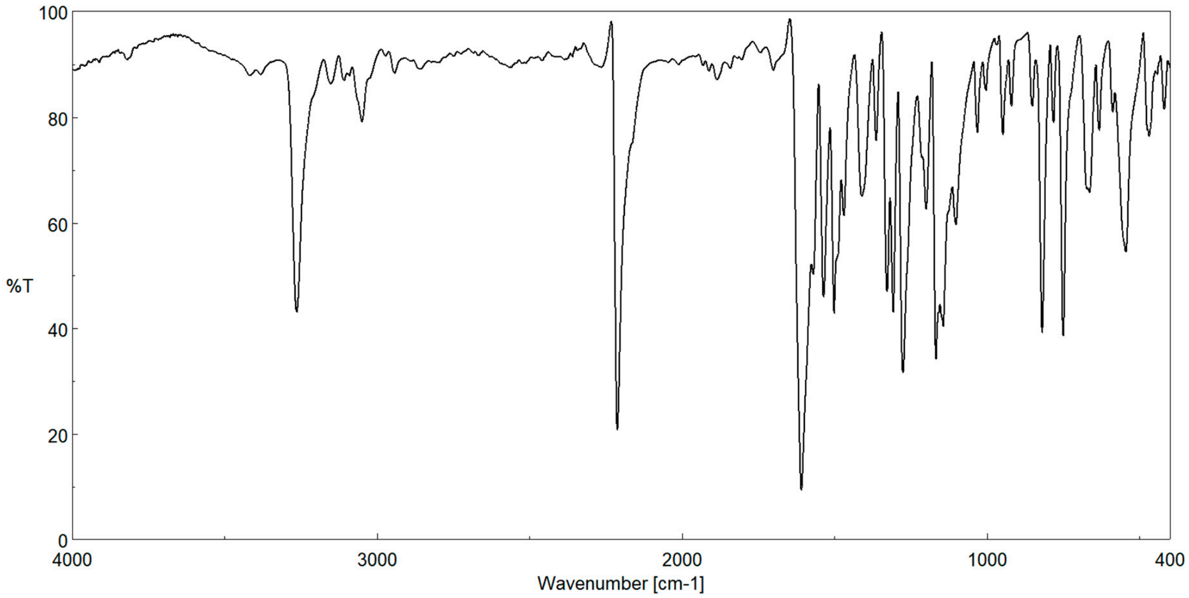

Figure S84. IR spectrum of (E)-4-(2-(2-hydroxybenzylidene)hydrazineyl)benzonitrile (H4).

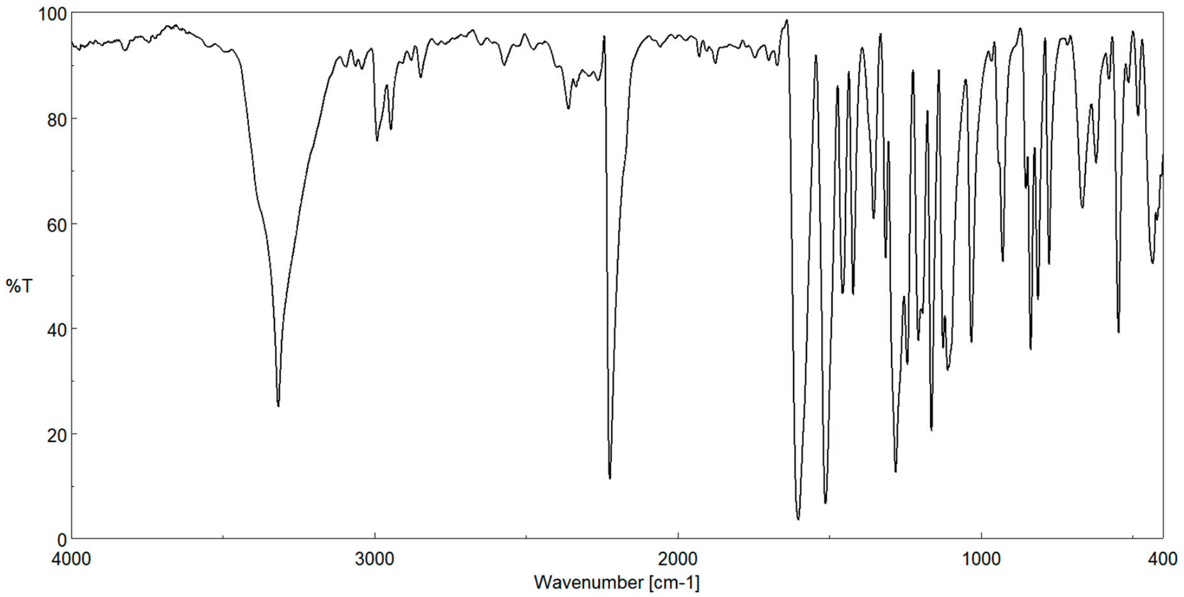

Figure S85. IR spectrum of (E)-4-(2-(4-hydroxy-3-methoxybenzylidene)hydrazineyl)benzonitrile (H5).

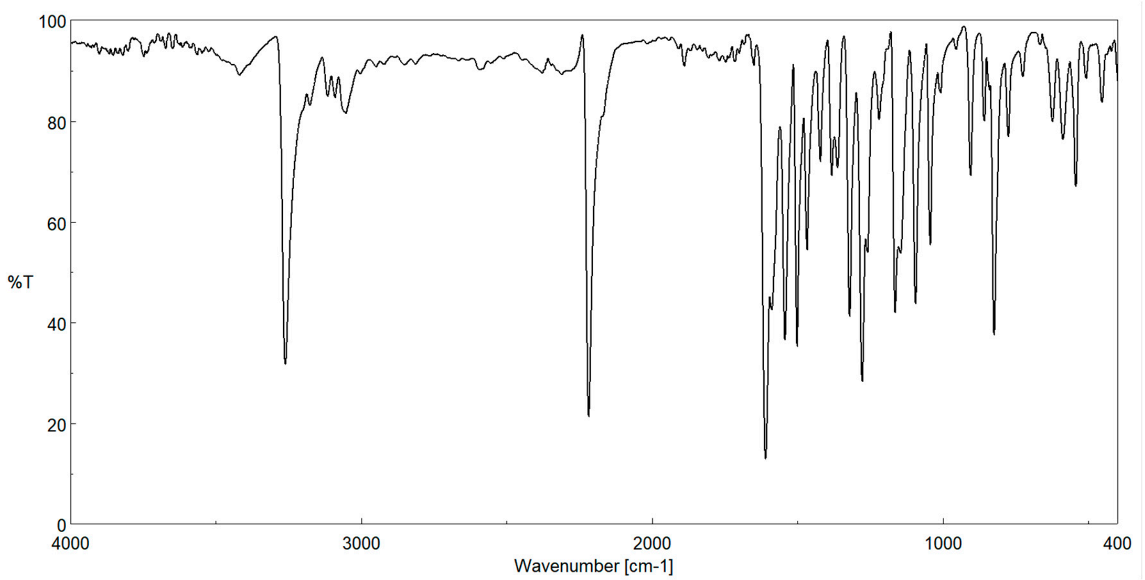

Figure S86. IR spectrum of (E)-4-(2-(2,4-dichlorobenzylidene)hydrazineyl)benzonitrile (H6).

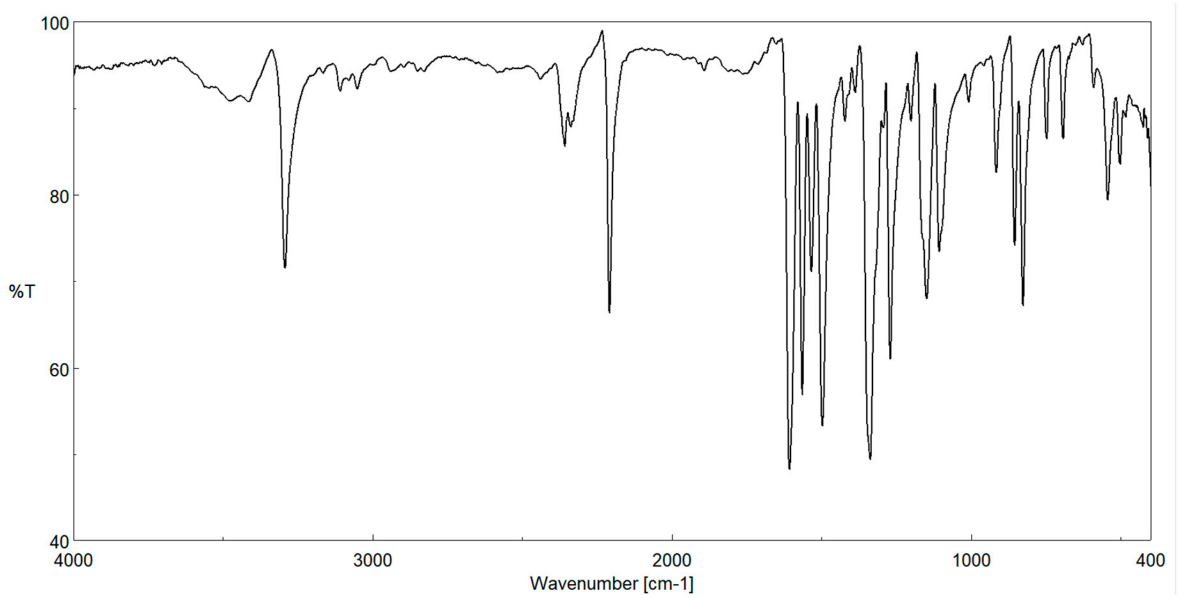

Figure S87. IR spectrum of (E)-4-(2-((4-nitrobenzylidene)hydrazineyl)benzonitrile (H7).

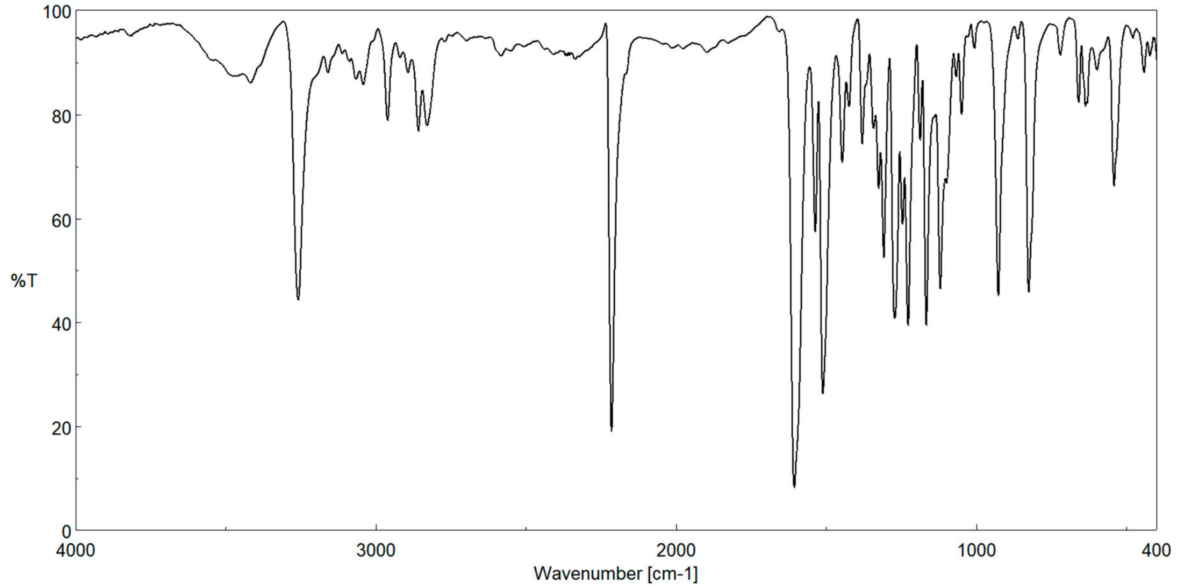

Figure S88. IR spectrum of (E)-4-(2-(4-morpholinobenzylidene)hydrazineyl)benzonitrile (H8).

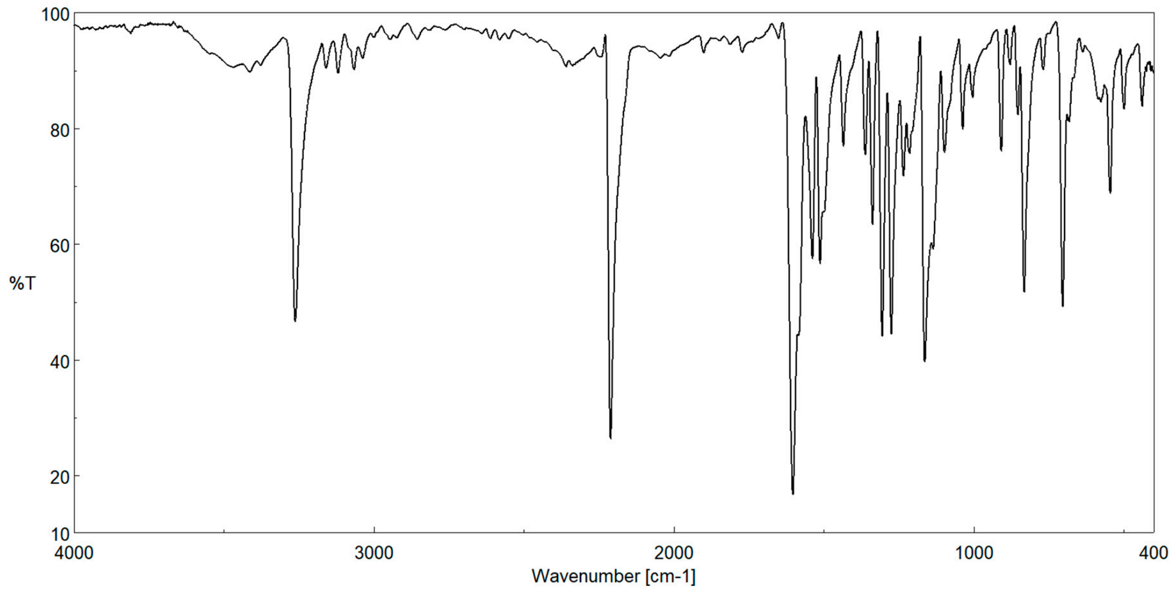

Figure S89. IR spectrum of (E)-4-(2-(thiophen-2-ylmethylene)hydrazineyl)benzonitrile (H9).

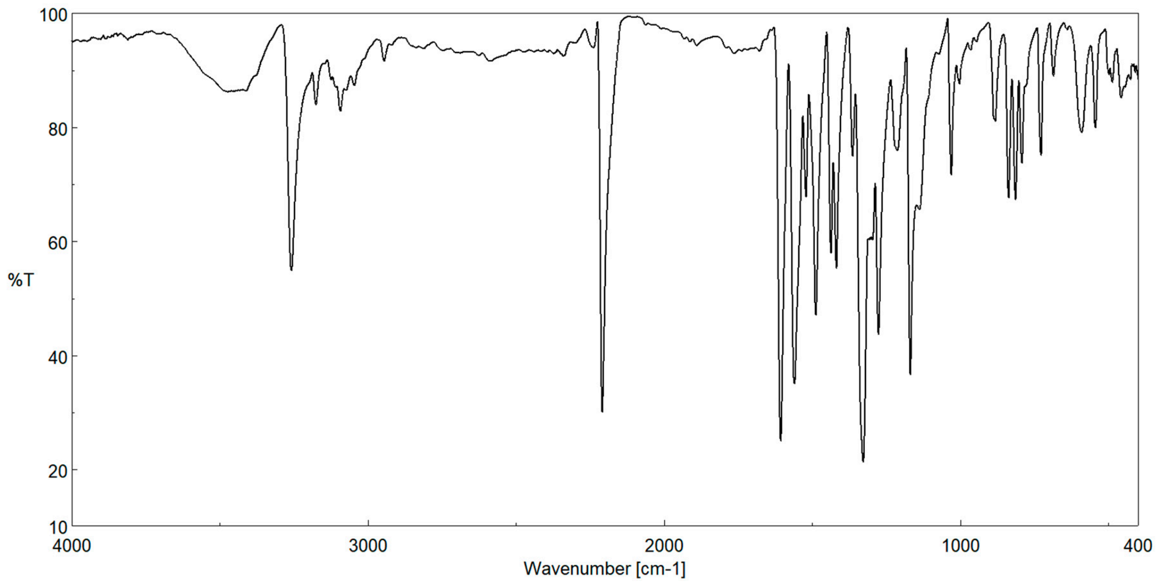

Figure S90. IR spectrum of (E)-4-(2-((5-nitrothiophen-2yl)methylene)hydrazineyl)benzonitrile (H10).

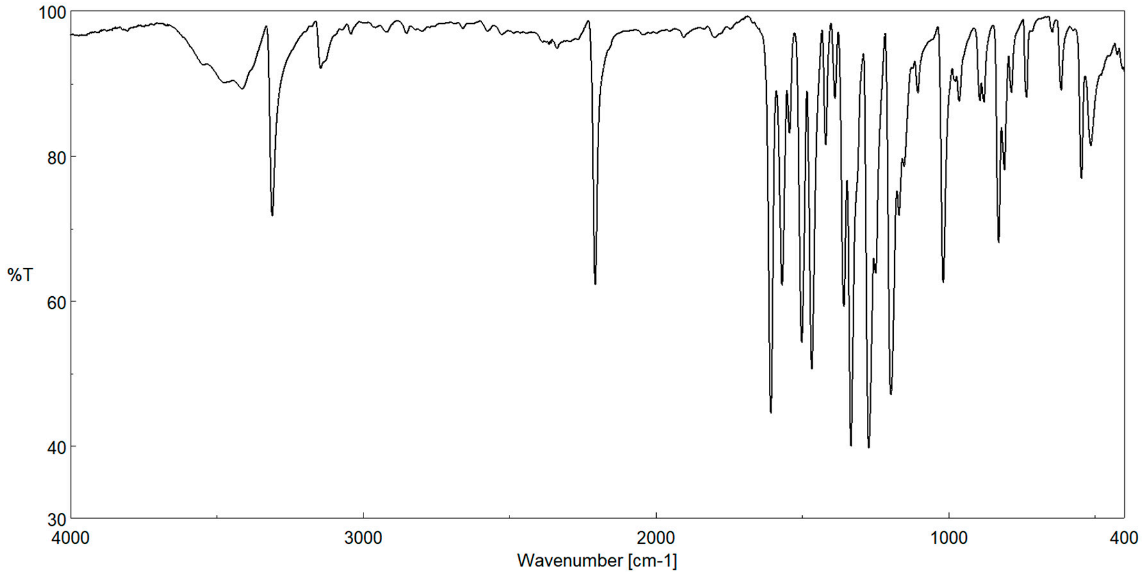

Figure S91. IR spectrum of (E)-4-(2-((5-nitrofuran-2-yl)methylene)hydrazineyl)benzonitrile (H11).

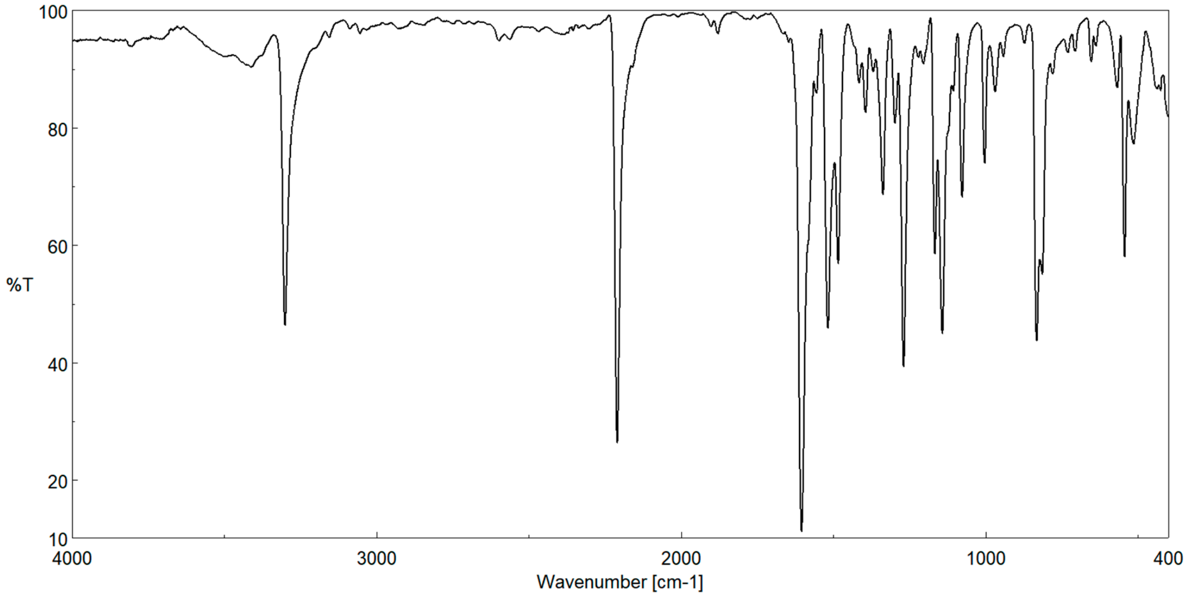

Figure S92. IR spectrum of (E)-4-(2-(1-(4-bromophenyl)ethylidene)hydrazineyl)benzonitrile (H12).

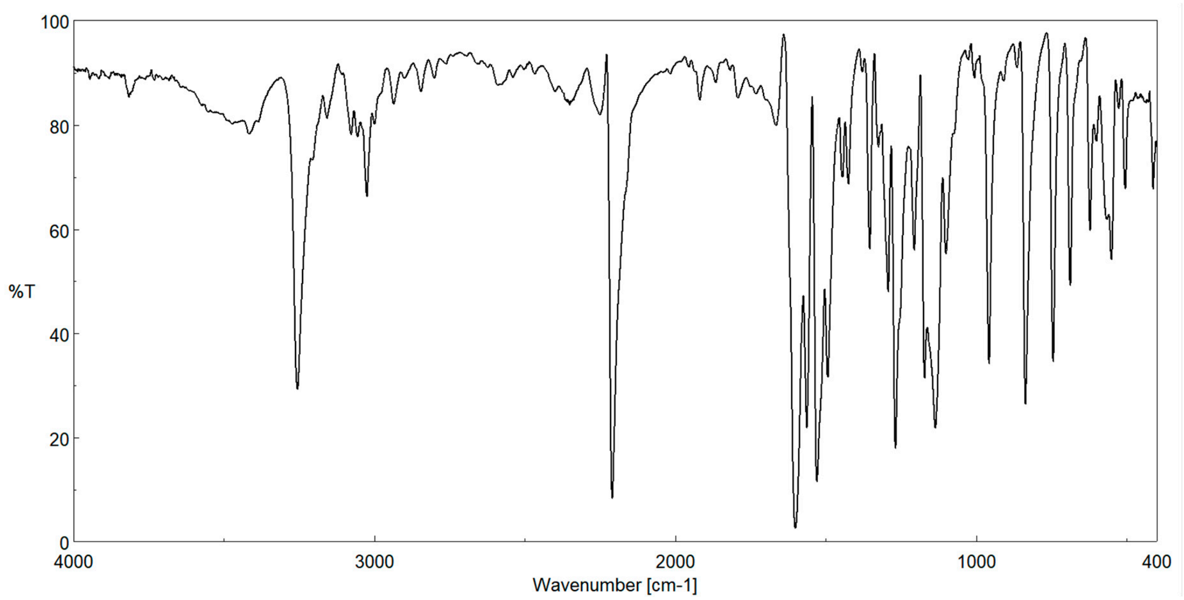

Figure S93. IR spectrum of 4-(2-((1E,2E)-3-phenylallylidene)hydrazineyl)benzonitrile (H13).

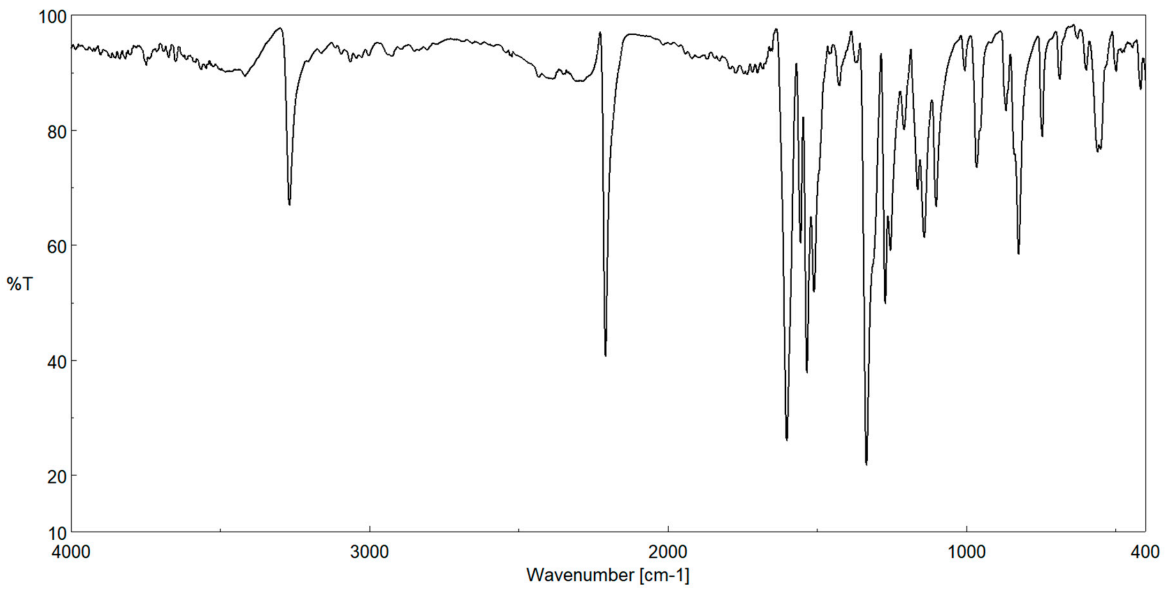

Figure S94. IR spectrum of 4-(2-((1E,2E)-3-(4-nitrophenyl)allylidene)hydrazineyl)benzonitrile (H14).

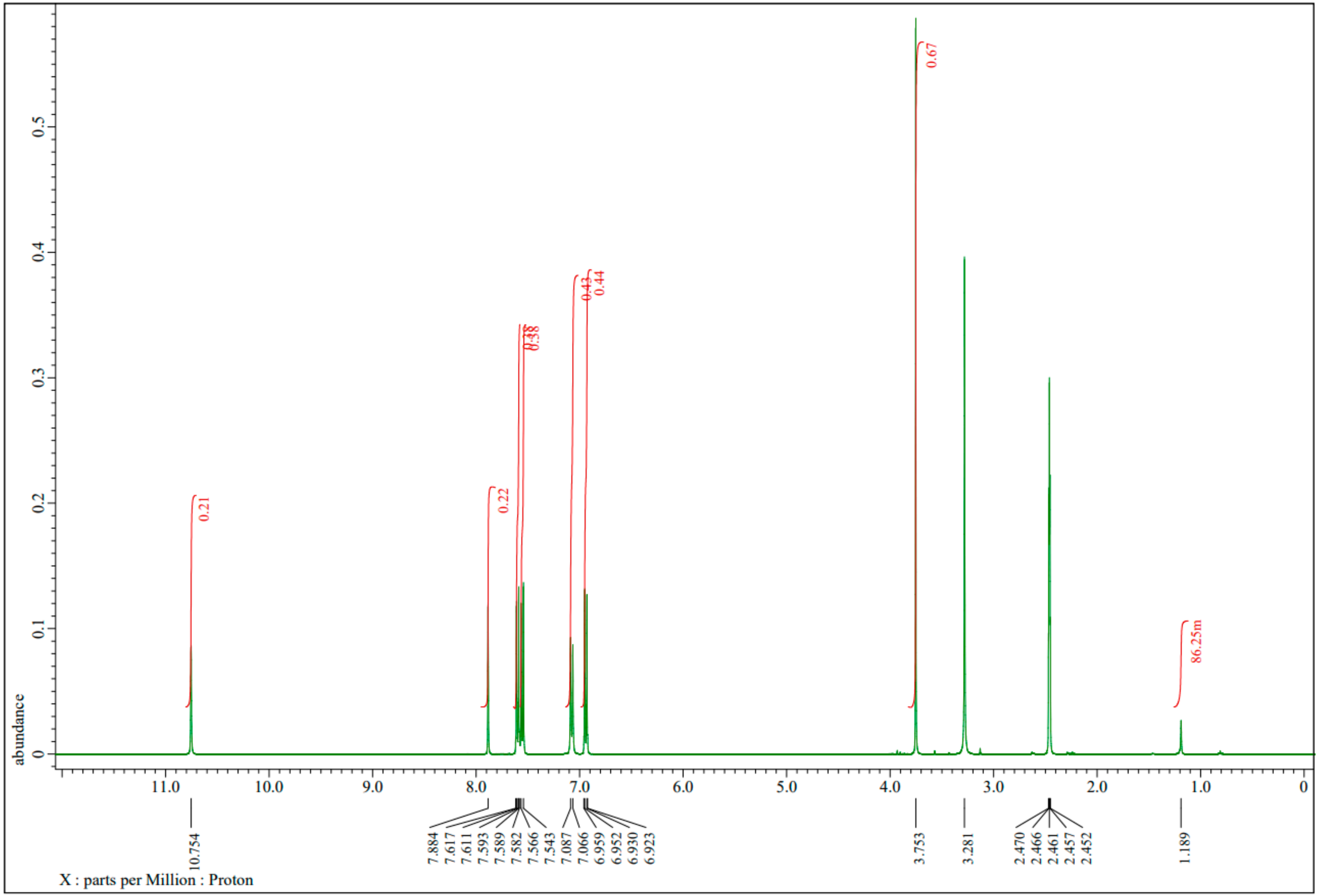

Figure S95. NMR spectrum of (E)-4-(2-(4-methoxybenzylidene)hydrazineyl)benzonitrile (H1).

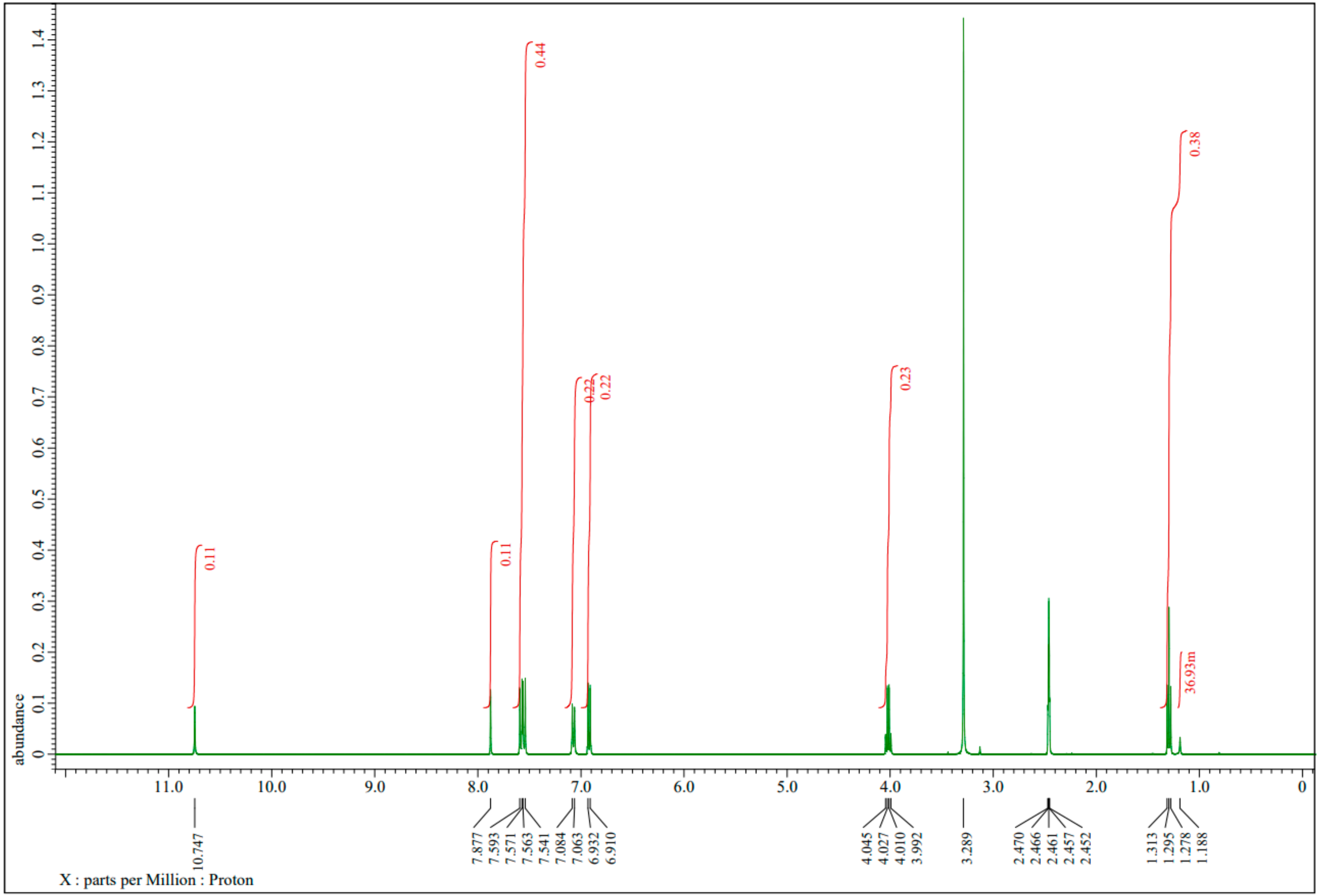

Figure S96. NMR spectrum of (E)-4-(2-(4-ethoxybenzylidene)hydrazineyl)benzonitrile (H2).

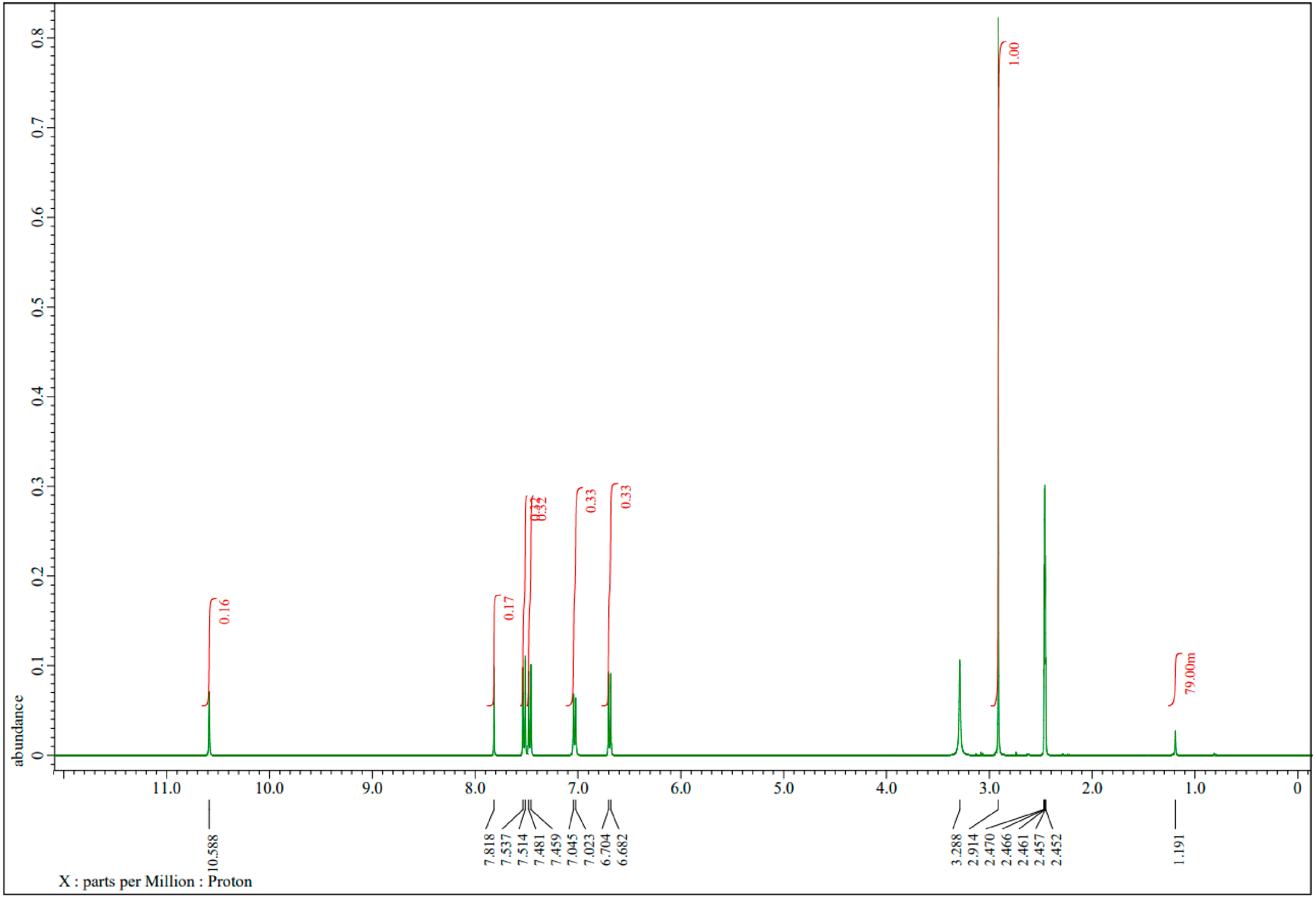

Figure S97. NMR spectrum of (E)-4-(2-(4-(dimethylamino)benzylidene)hydrazineyl)benzonitrile (H3).

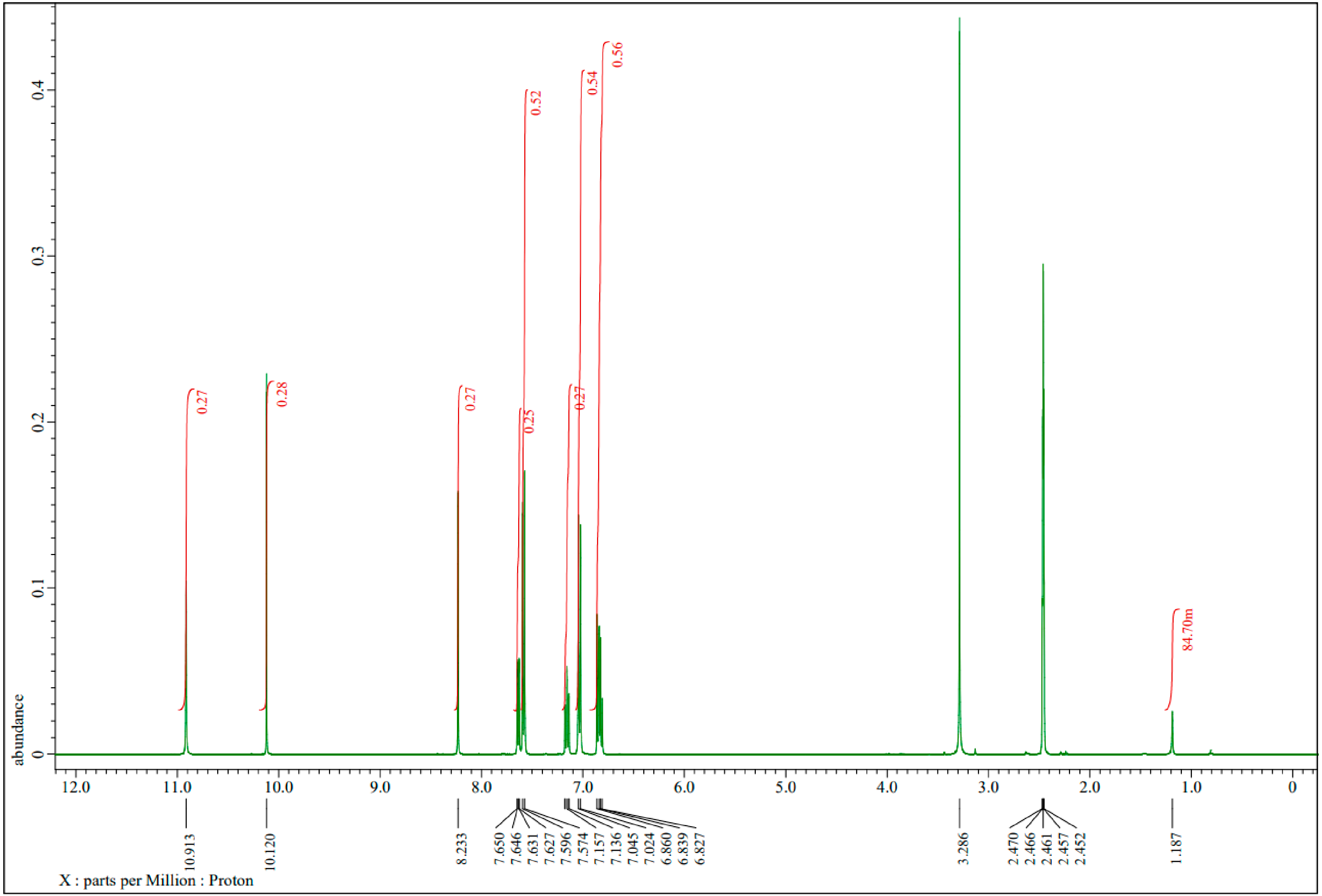

Figure S98. NMR spectrum of (E)-4-(2-(2-hydroxybenzylidene)hydrazineyl)benzonitrile (H4).

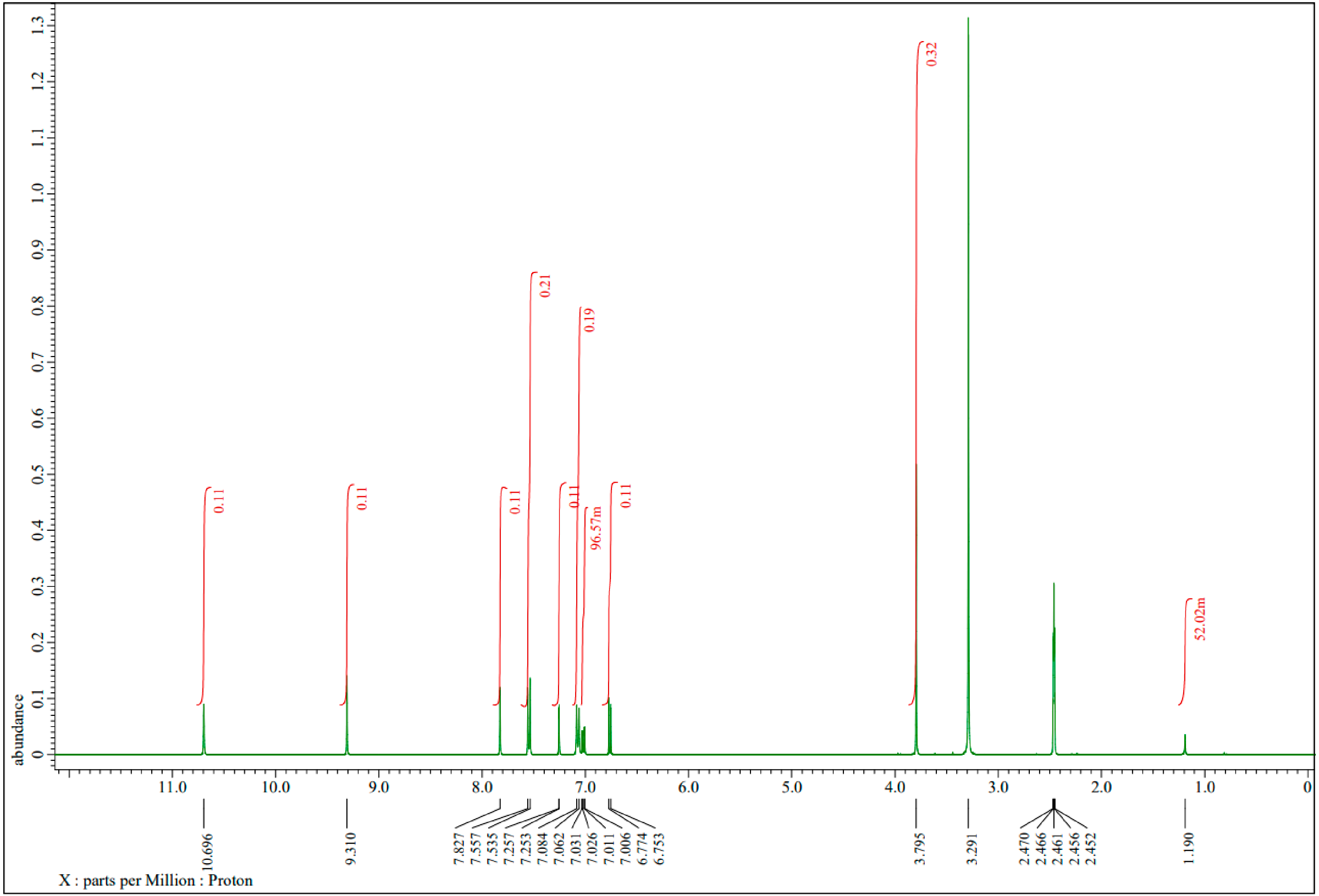

Figure S99. NMR spectrum of (E)-4-(2-(4-hydroxy-3-methoxybenzylidene)hydrazineyl)benzonitrile (H5).

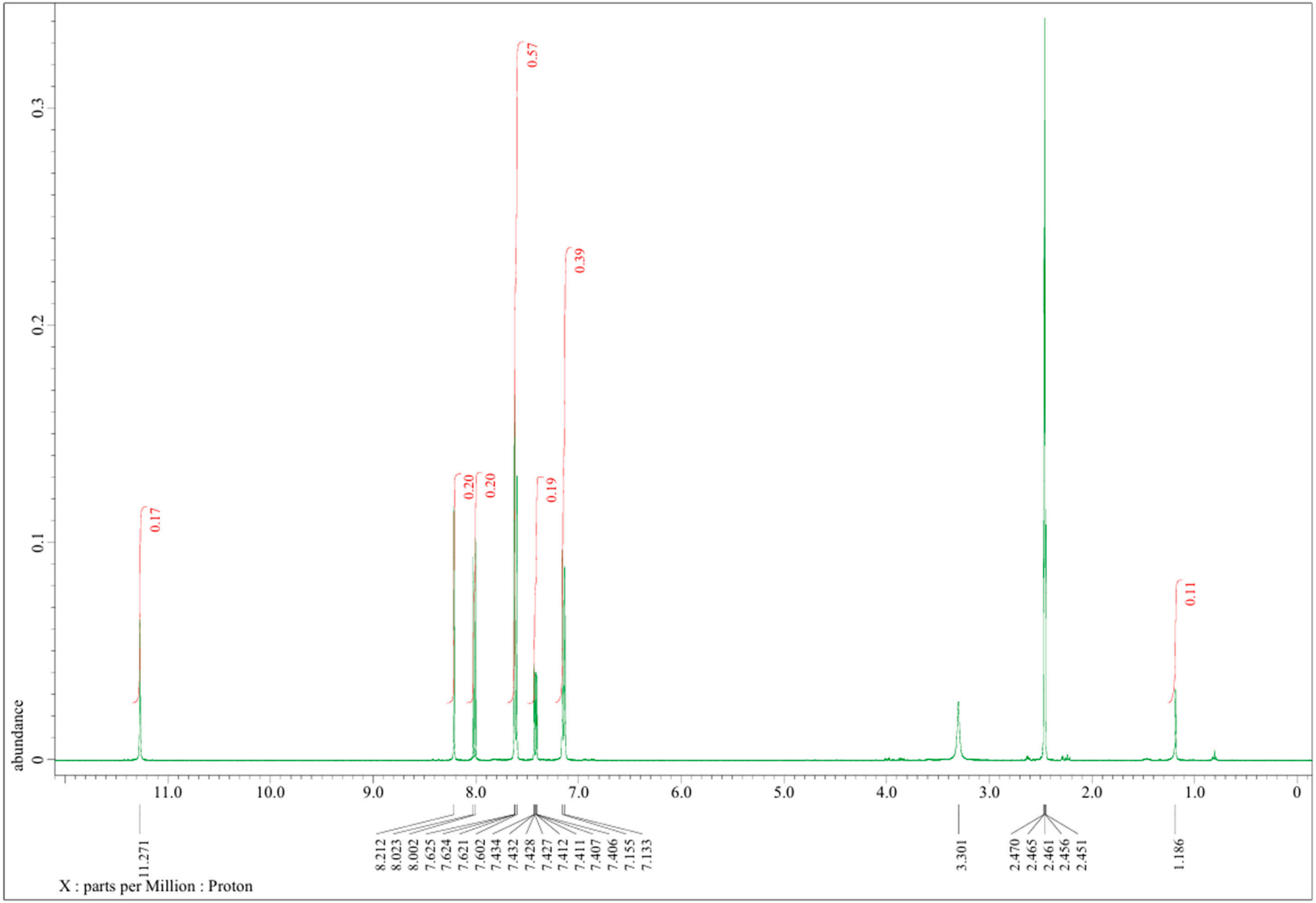

Figure S100. NMR spectrum of (E)-4-(2-(2,4-dichlorobenzylidene)hydrazineyl)benzonitrile (H6).

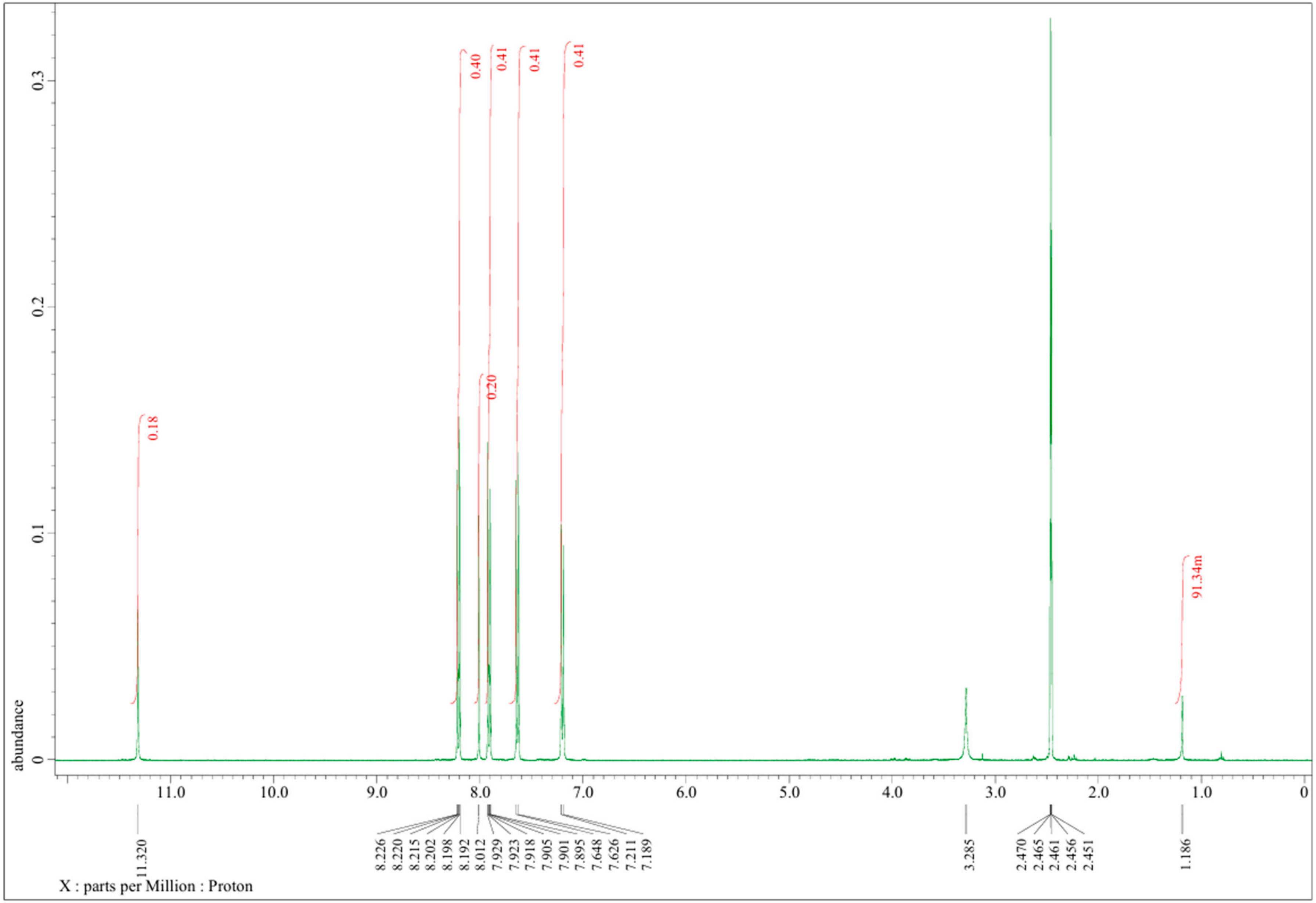

Figure S101. NMR spectrum of (E)-4-(2-((4-nitrobenzylidene)hydrazineyl)benzonitrile (H7).

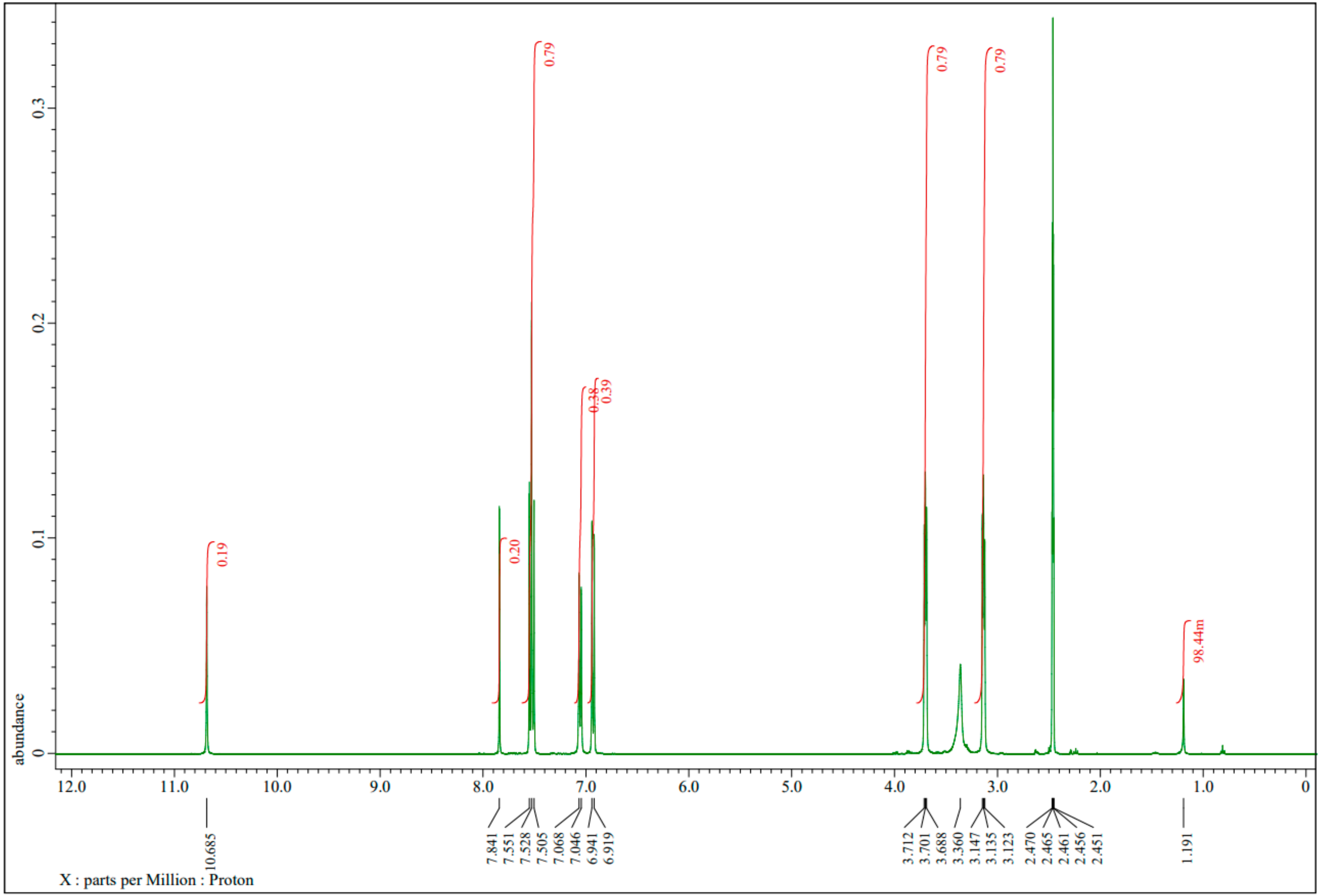

Figure S102. NMR spectrum of (E)-4-(2-(4-morpholinobenzylidene)hydrazineyl)benzonitrile (H8).

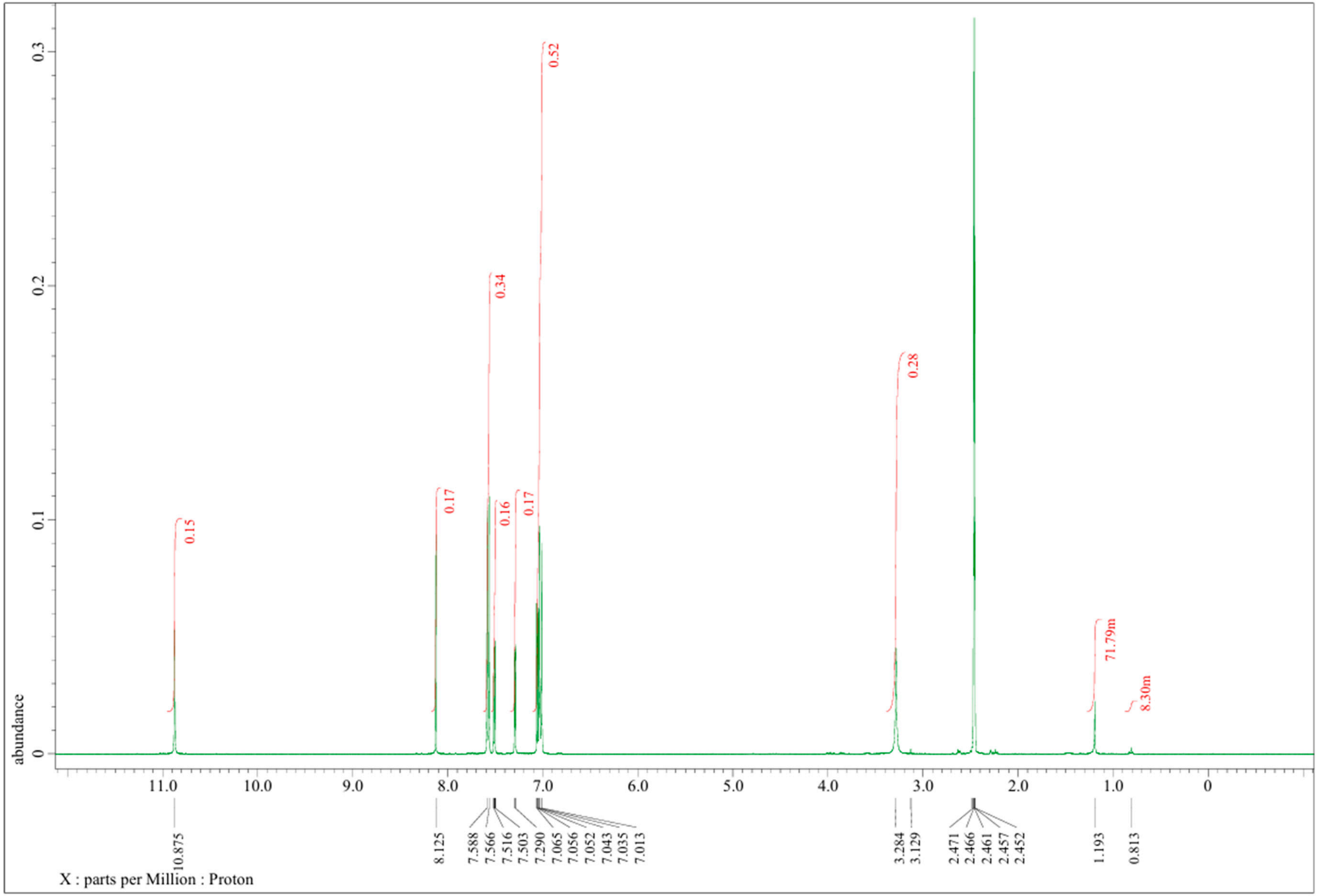

Figure S103. NMR spectrum of (E)-4-(2-(thiophen-2-ylmethylene)hydrazineyl)benzonitrile (H9).

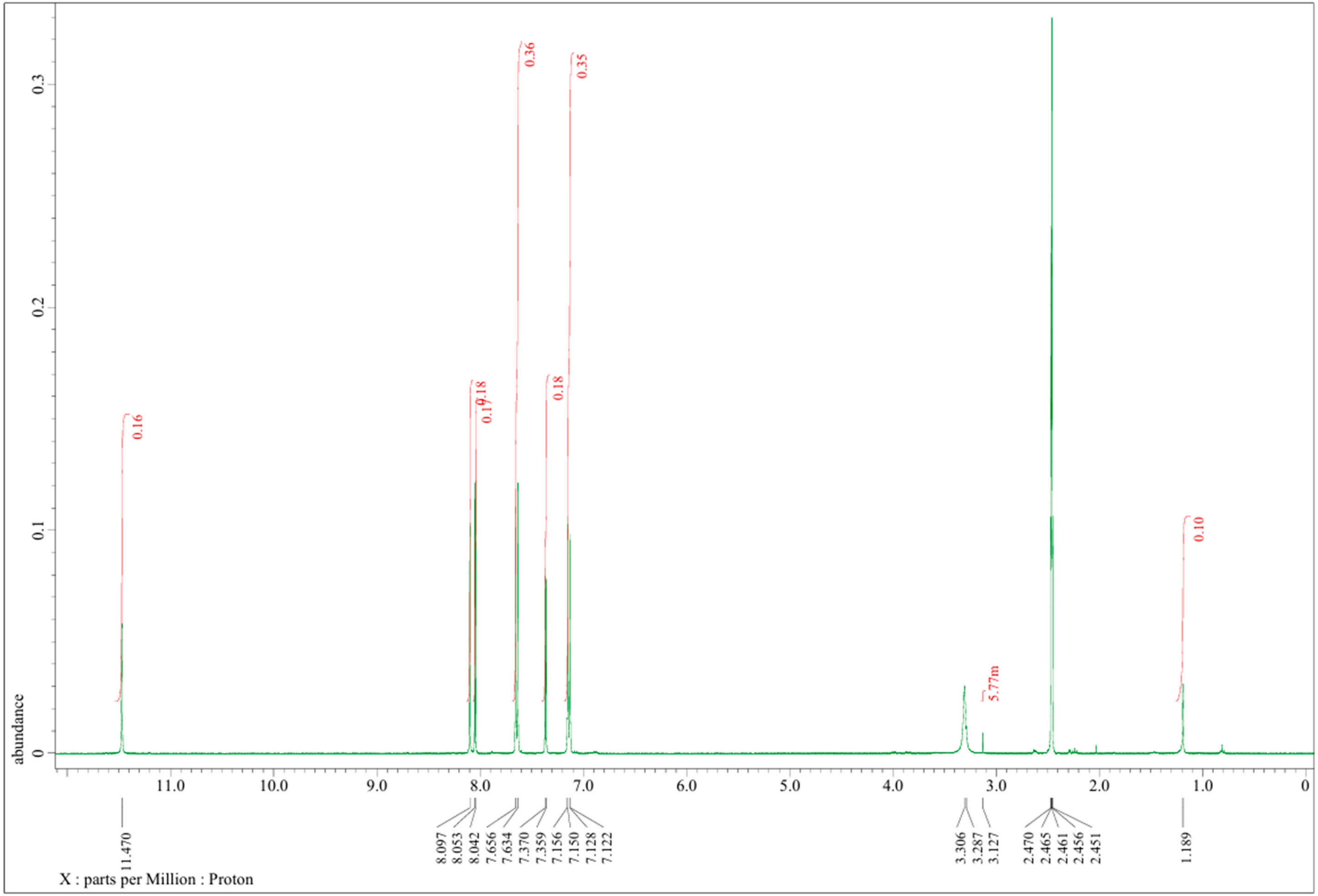

Figure S104. NMR spectrum of (E)-4-(2-((5-nitrothiophen-2yl)methylene)hydrazineyl)benzonitrile (H10).

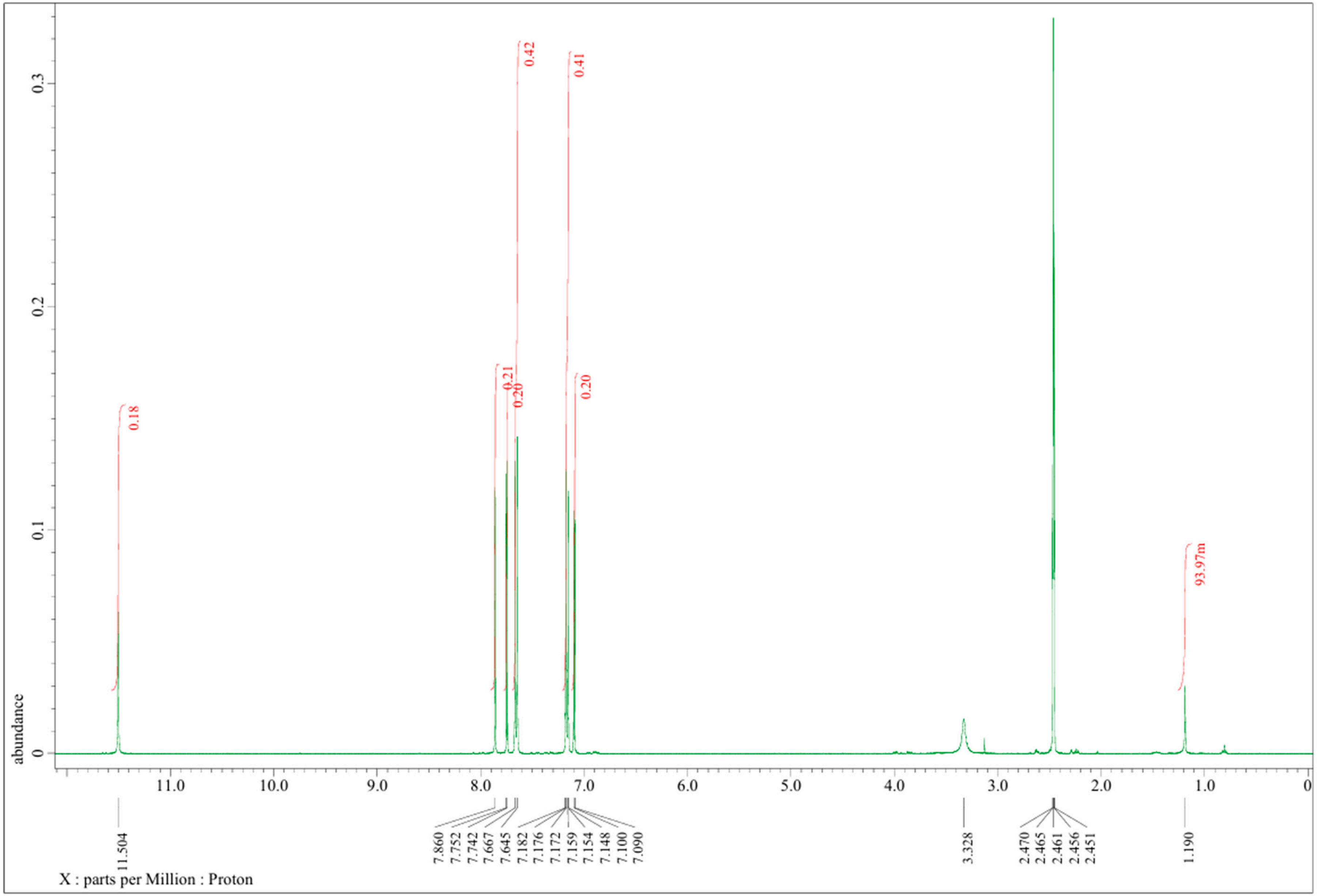

Figure S105. NMR spectrum of (E)-4-(2-((5-nitrofuran-2-yl)methylene)hydrazineyl)benzonitrile (H11).

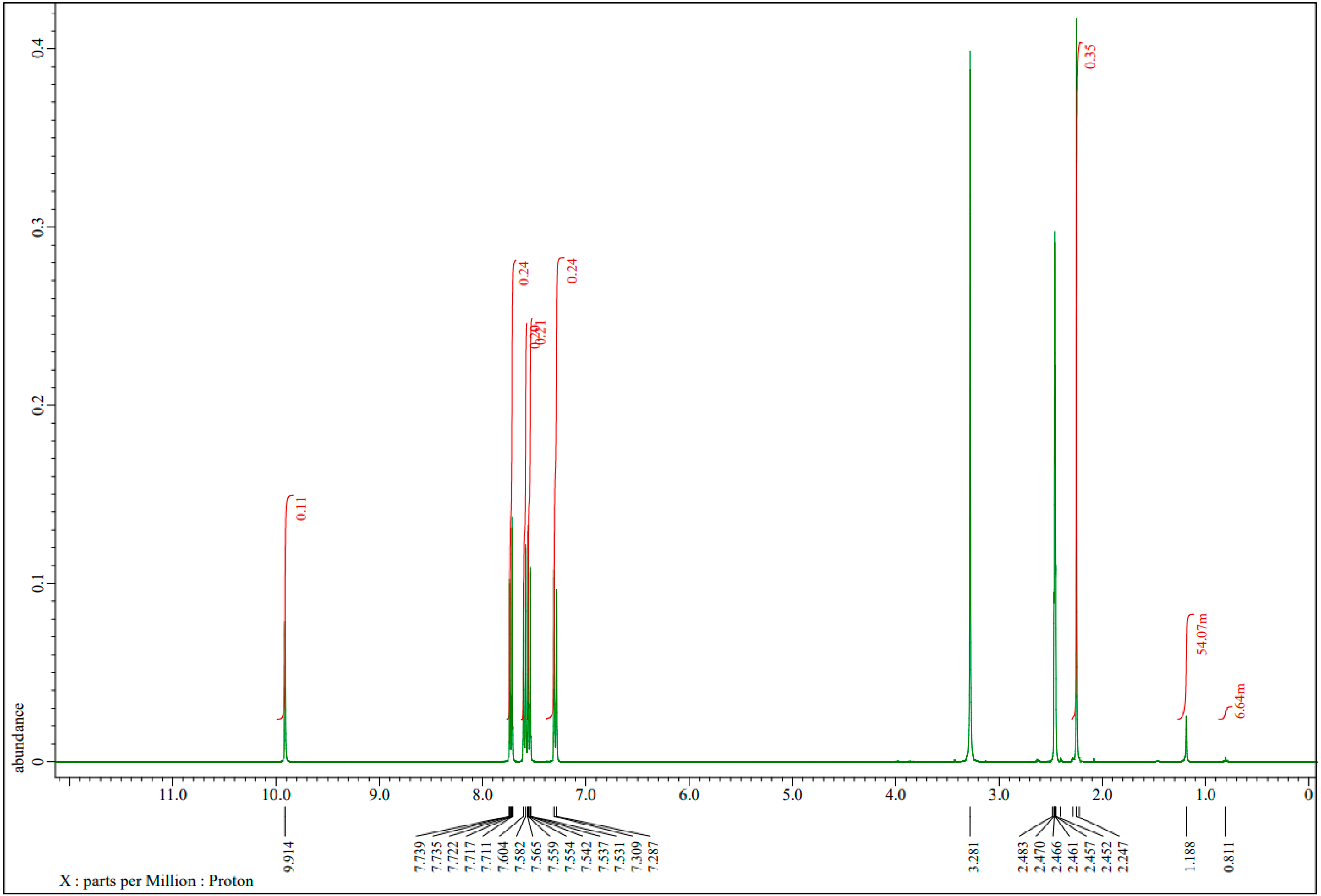

Figure S106. NMR spectrum of (E)-4-(2-(1-(4-bromophenyl)ethylidene)hydrazineyl)benzonitrile (H12).

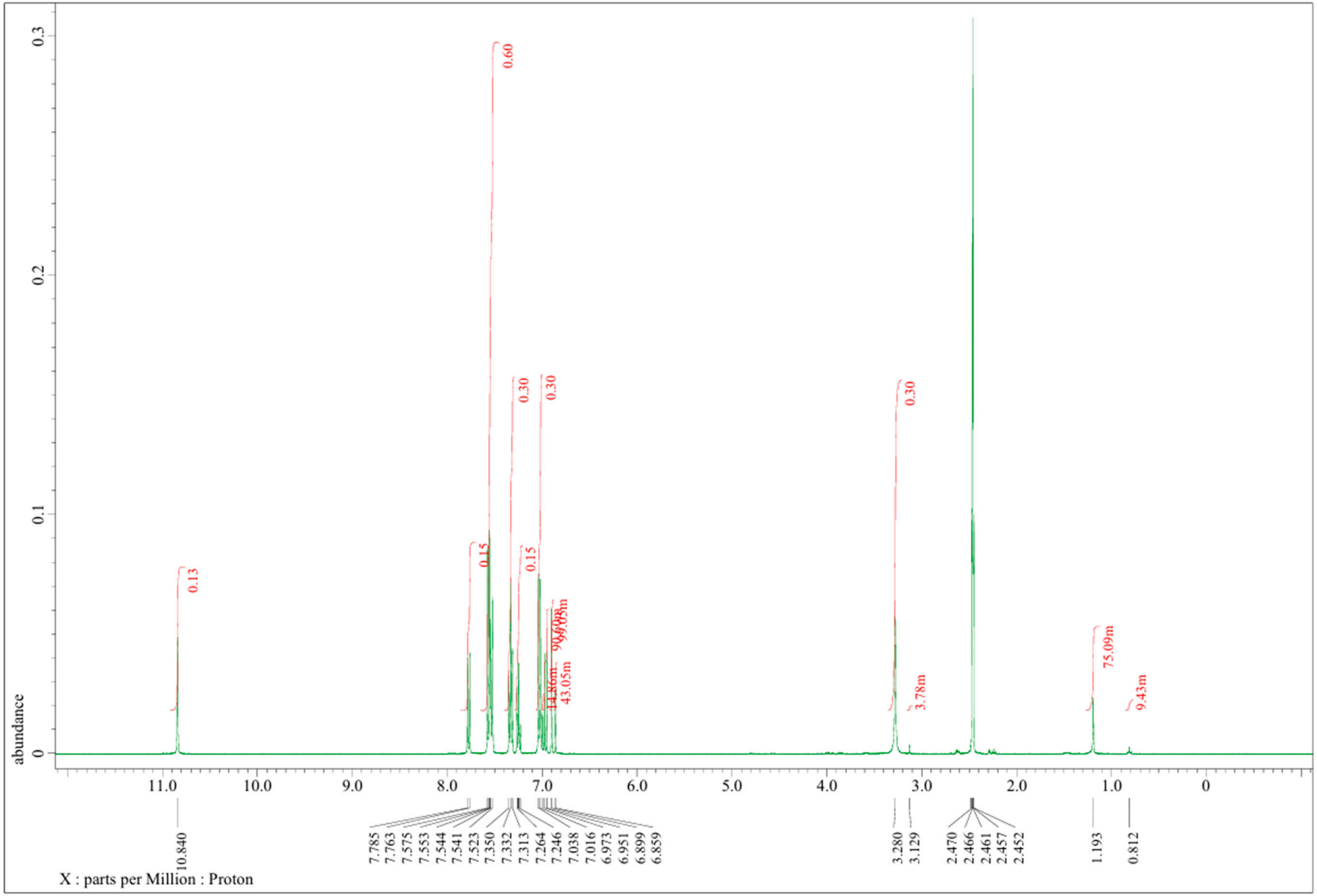

Figure S107. NMR spectrum of 4-(2-((1E,2E)-3-phenylallylidene)hydrazineyl)benzonitrile (H13).

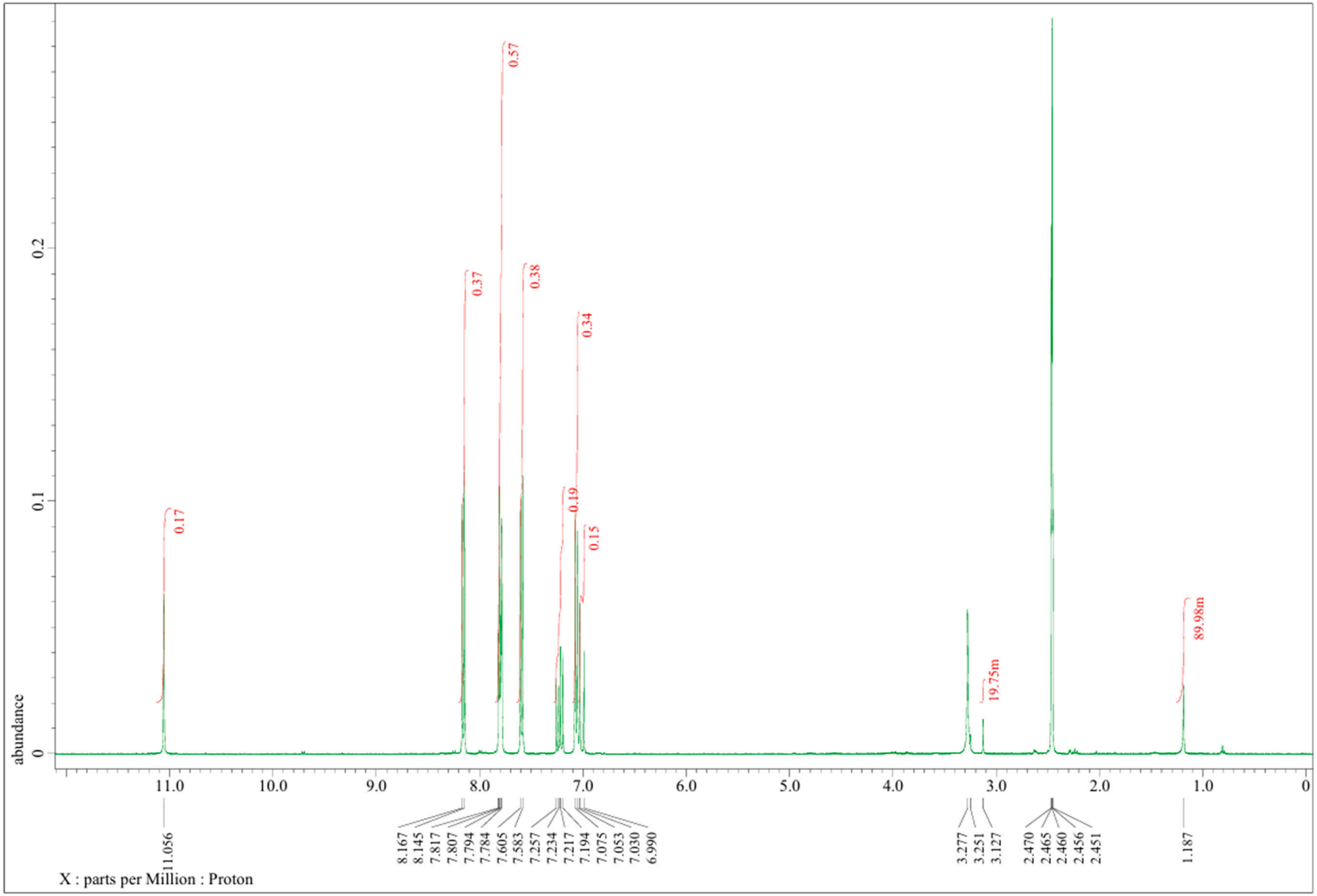

Figure S108. NMR spectrum of 4-(2-((1E,2E)-3-(4-nitrophenyl)allylidene)hydrazineyl)benzonitrile (H14).

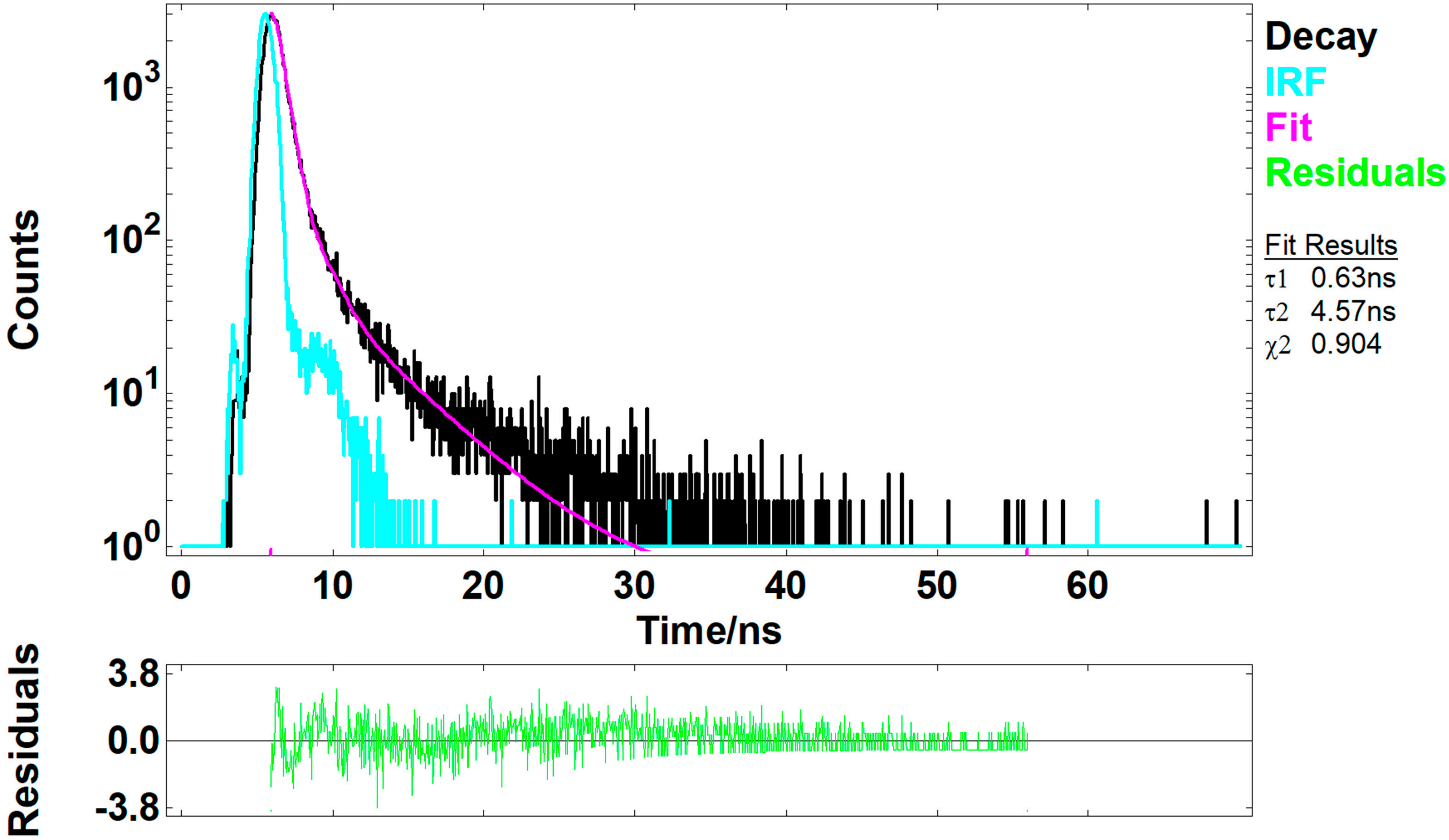

Figure S109. Fluorescence lifetime of **H1**.

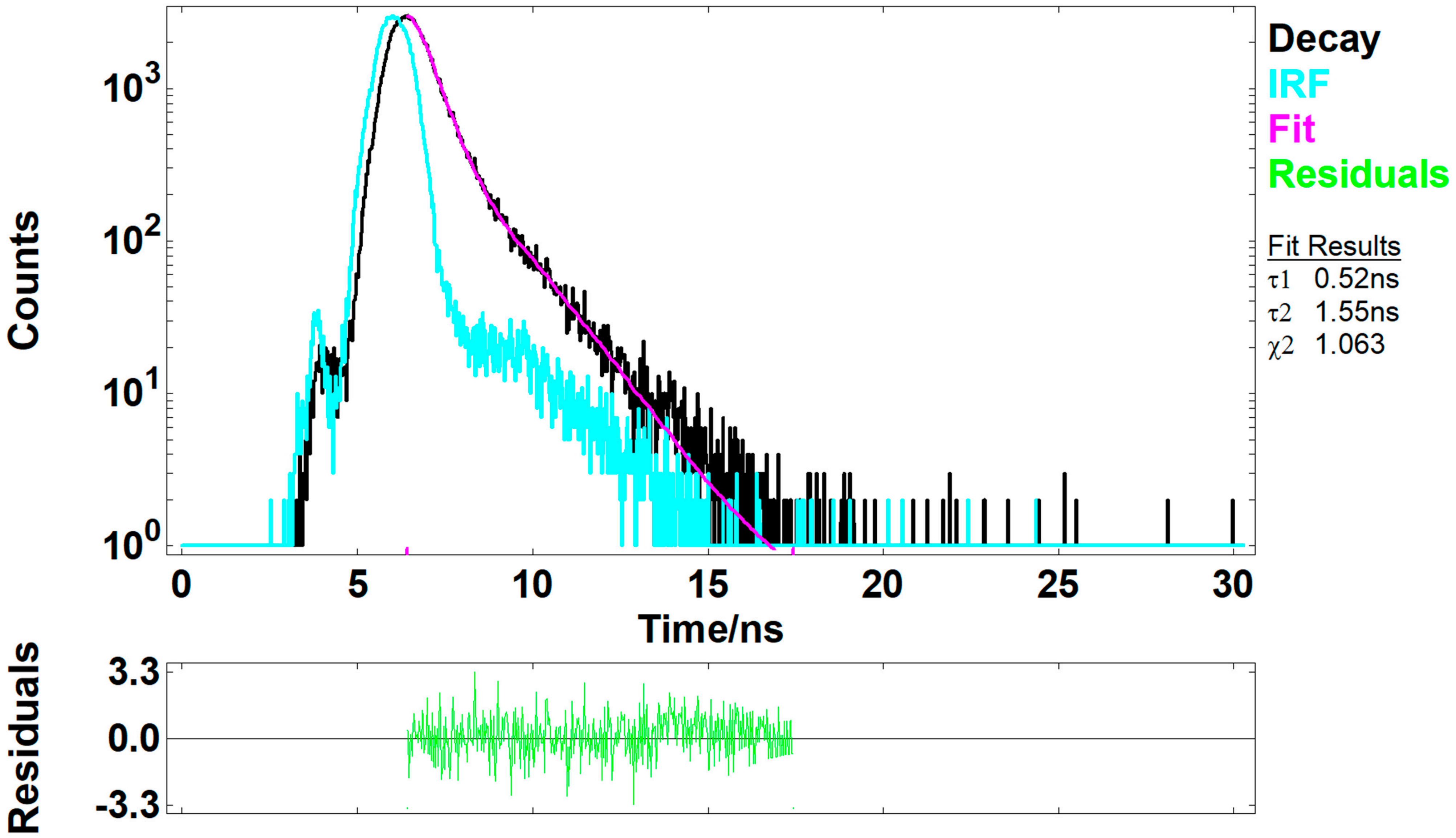

Figure S110. Fluorescence lifetime of **H2**.

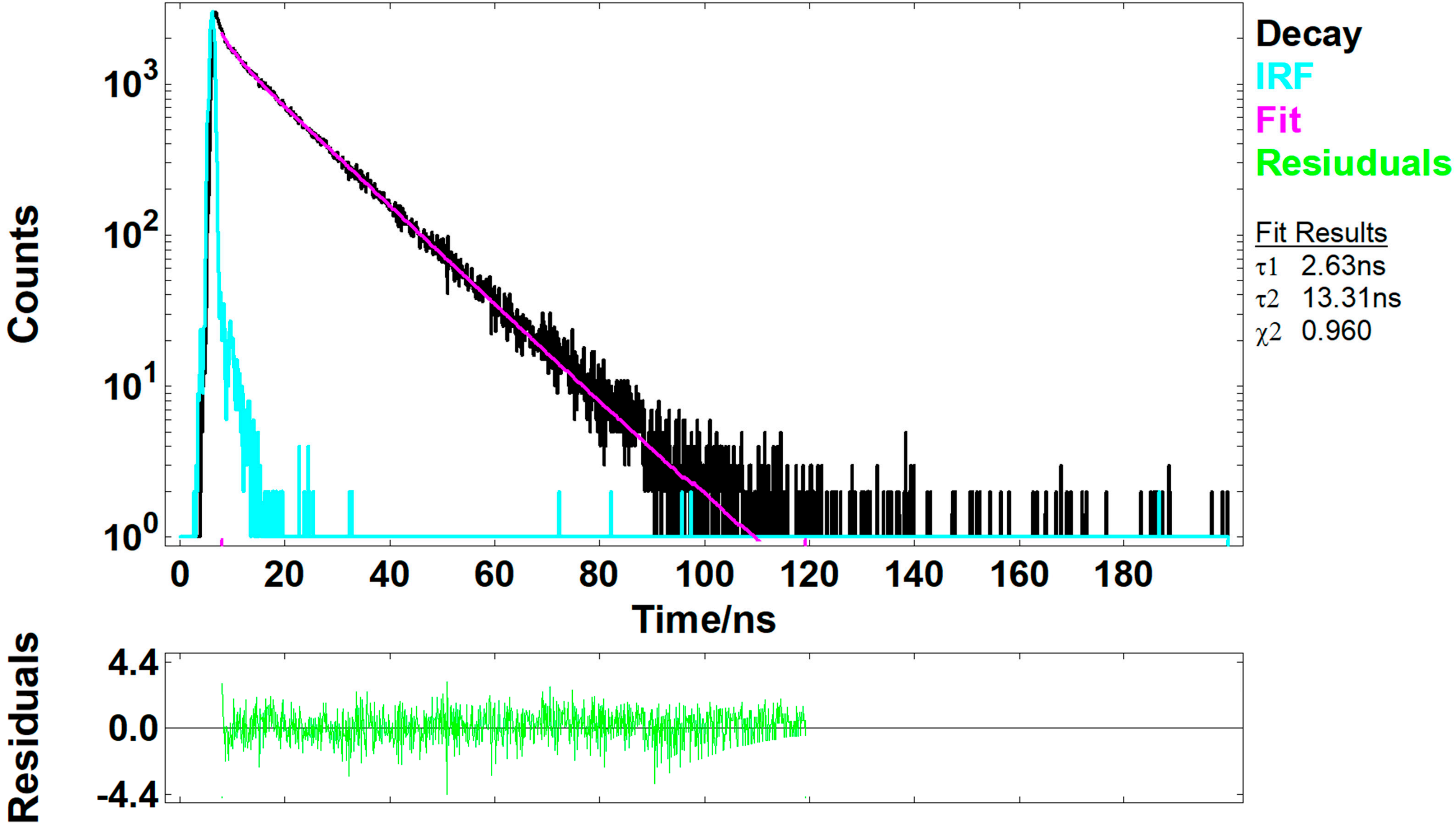

Figure S111. Fluorescence lifetime of **H3**.

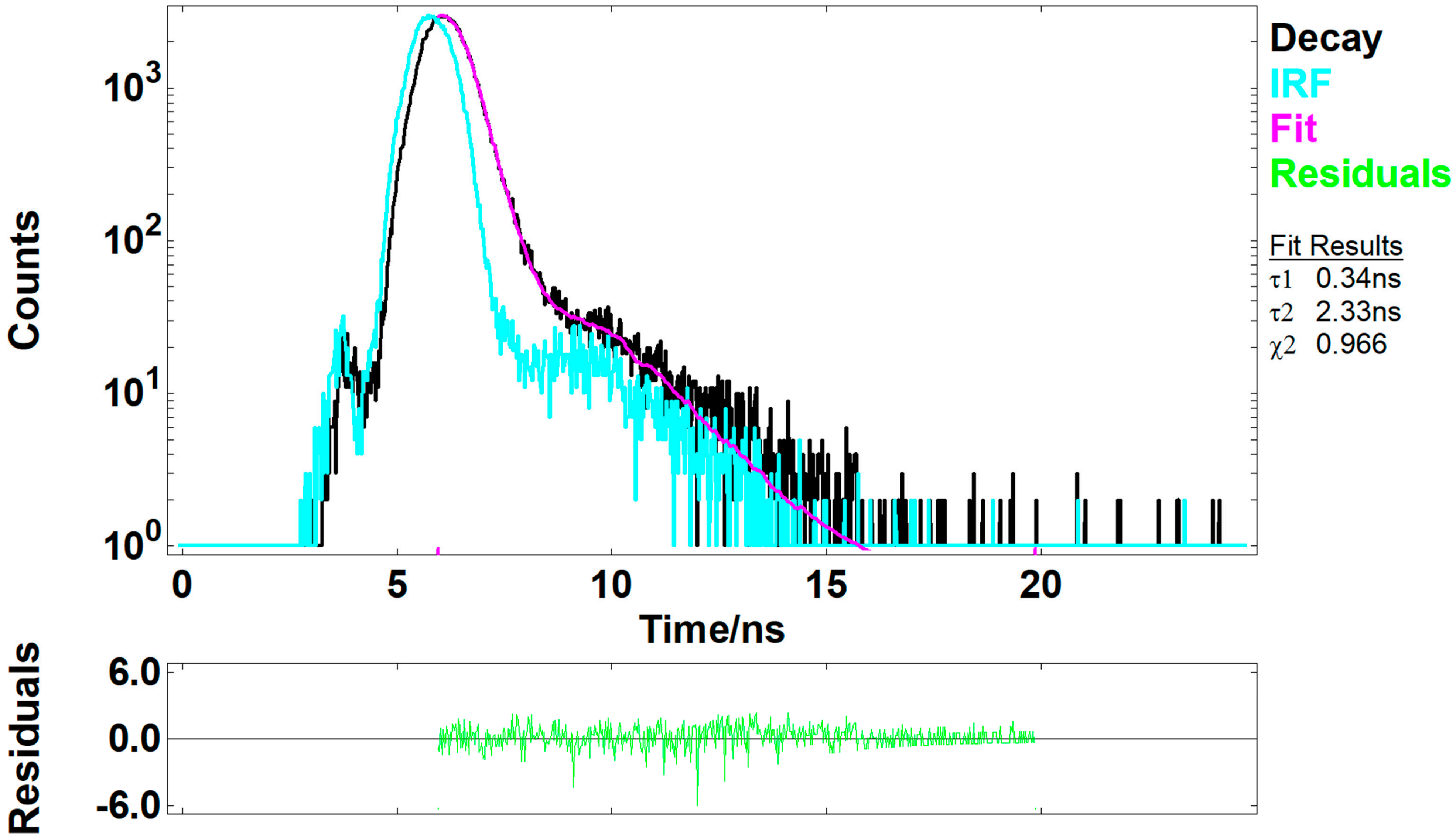

Figure S112. Fluorescence lifetime of **H4**.

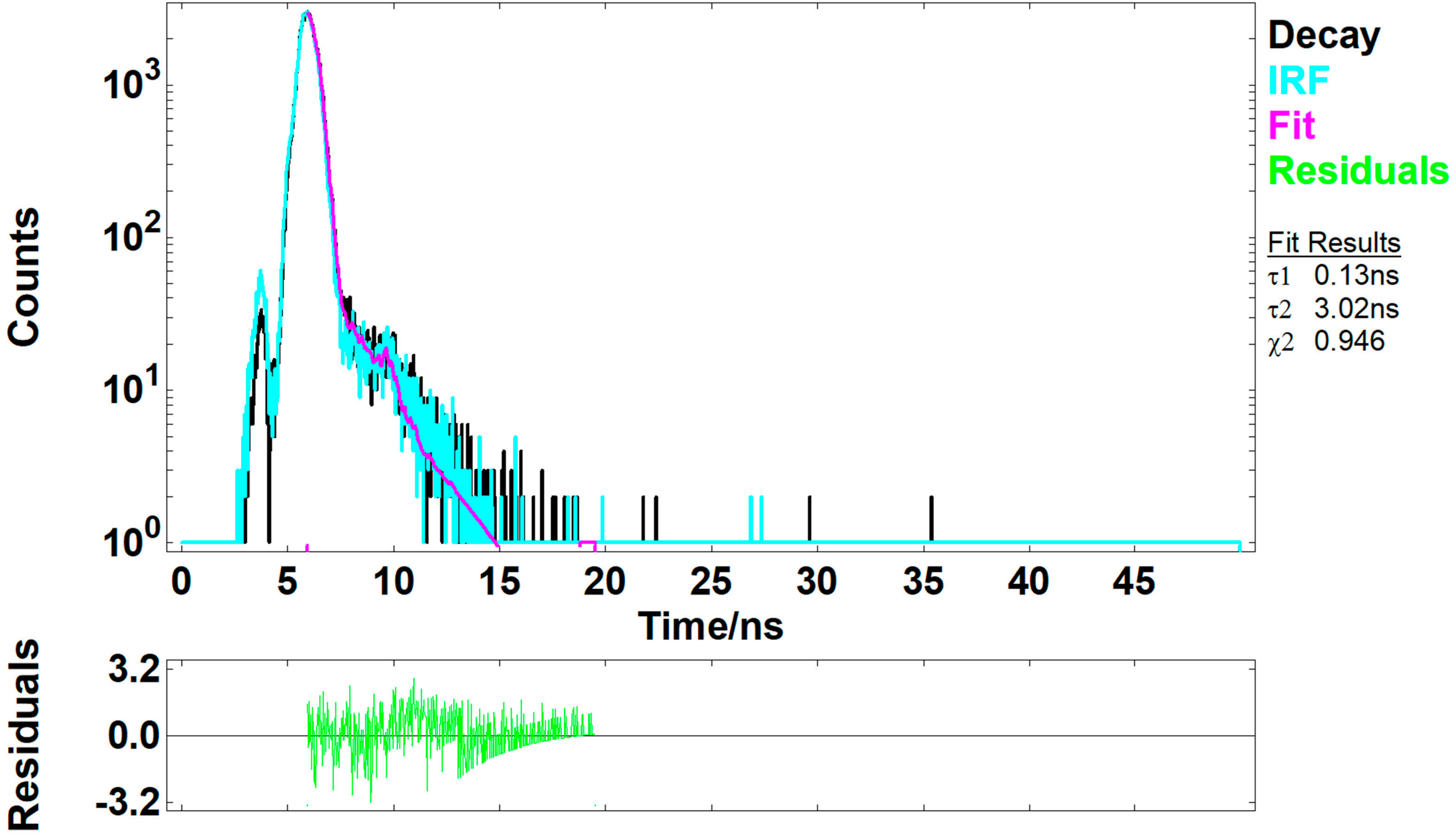

Figure S113. Fluorescence lifetime of **H5**.

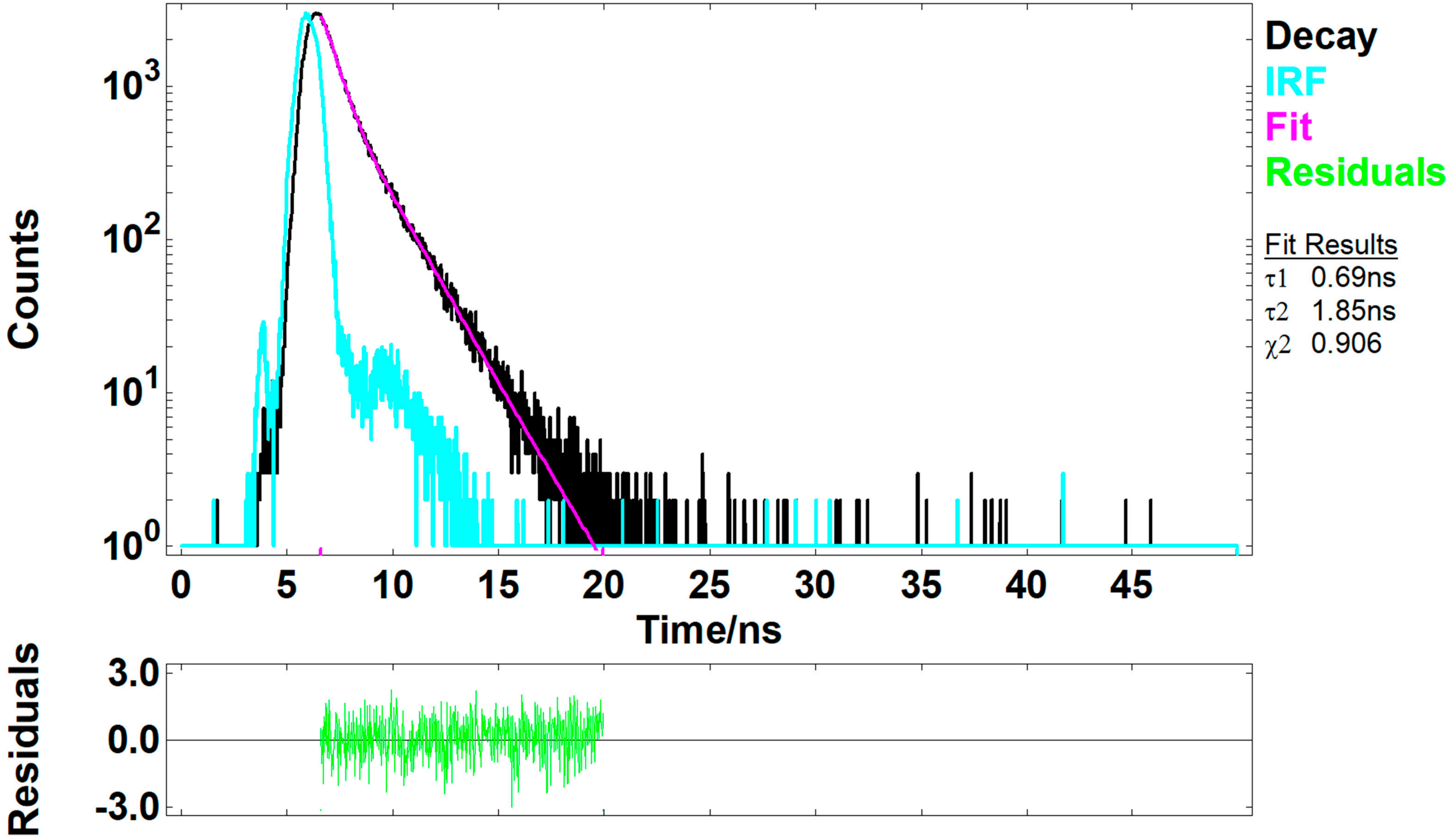

Figure S114. Fluorescence lifetime of **H6**.

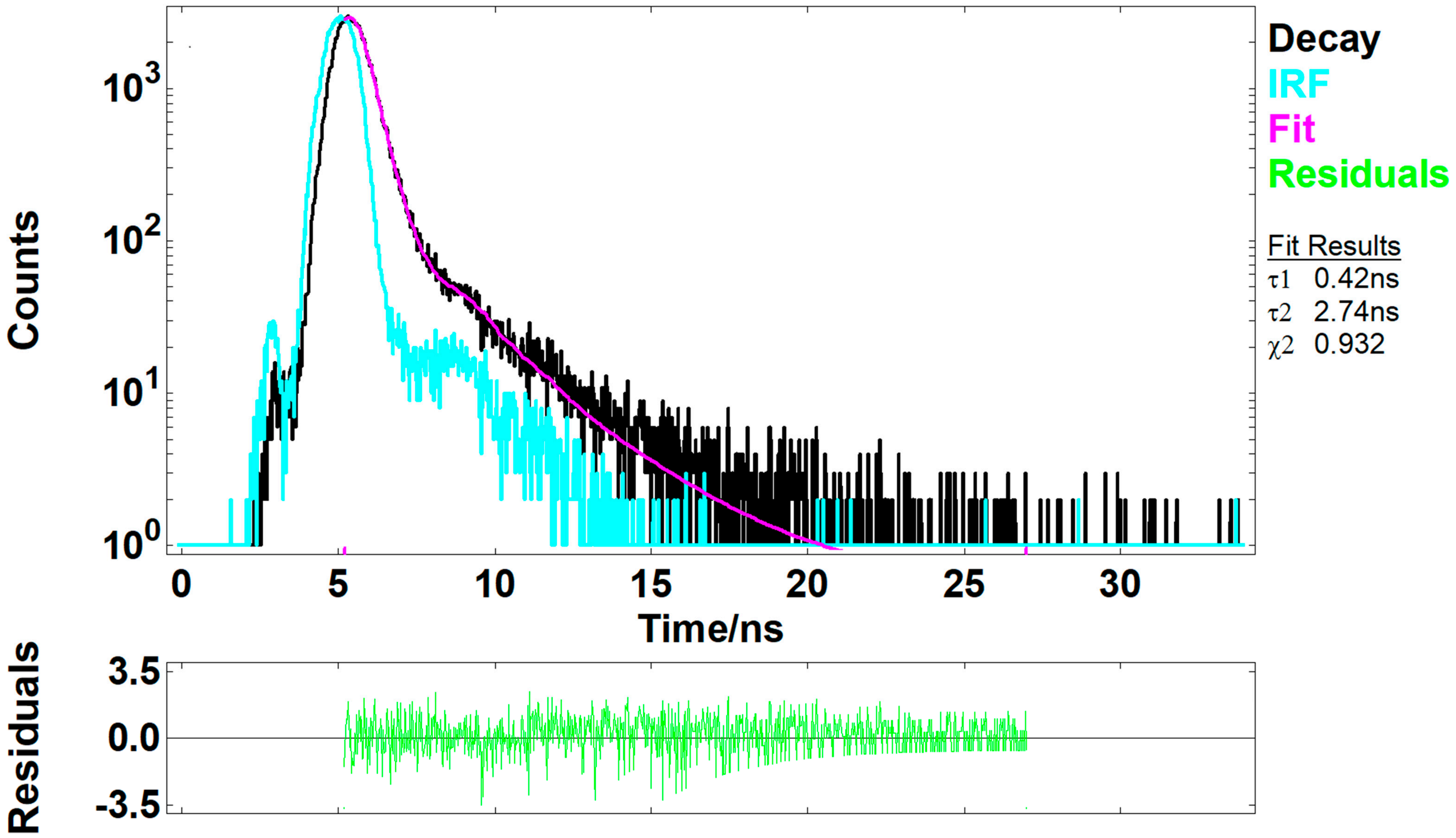

Figure S115. Fluorescence lifetime of **H8**.

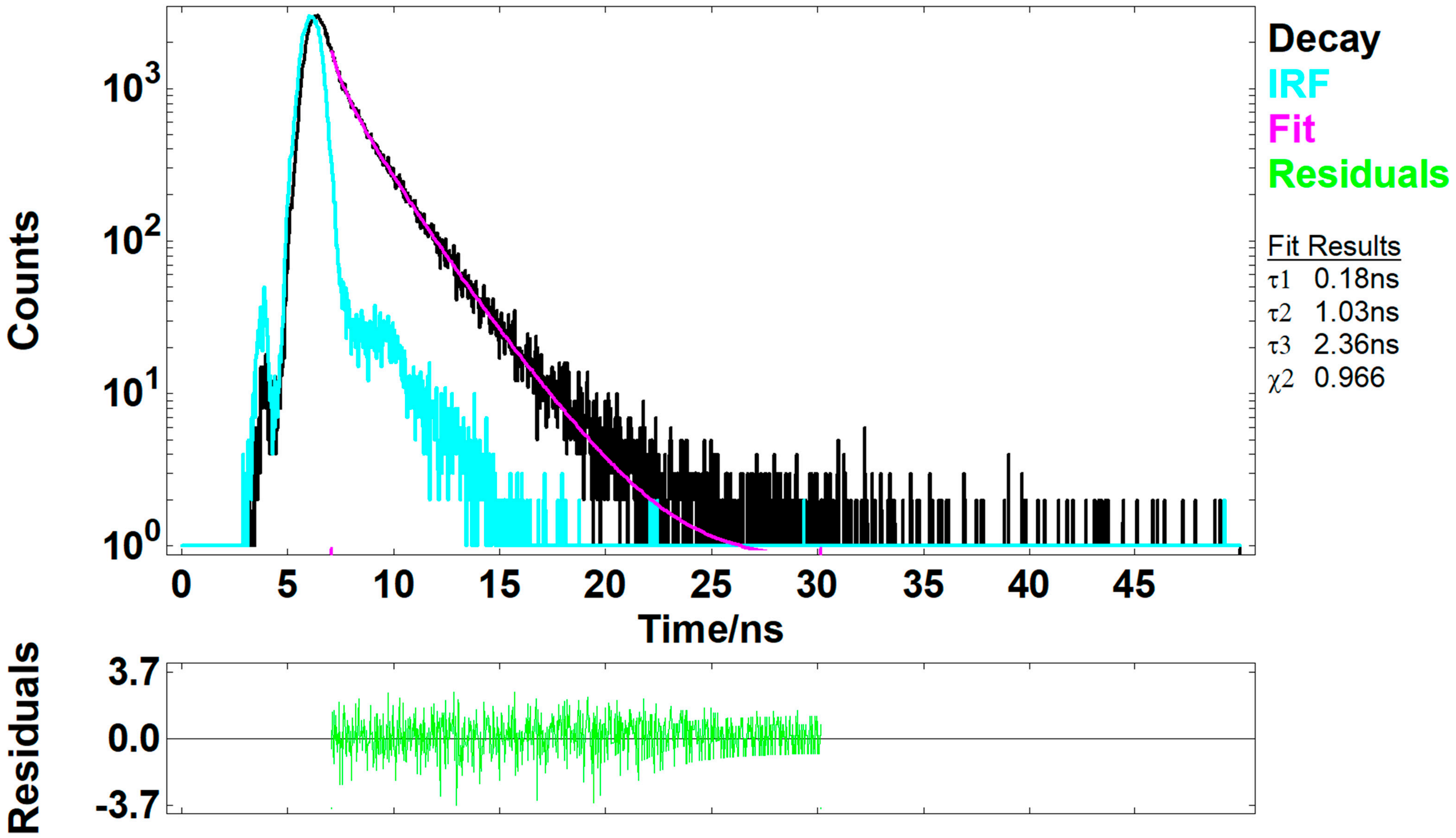

Figure S116. Fluorescence lifetime of **H12**.

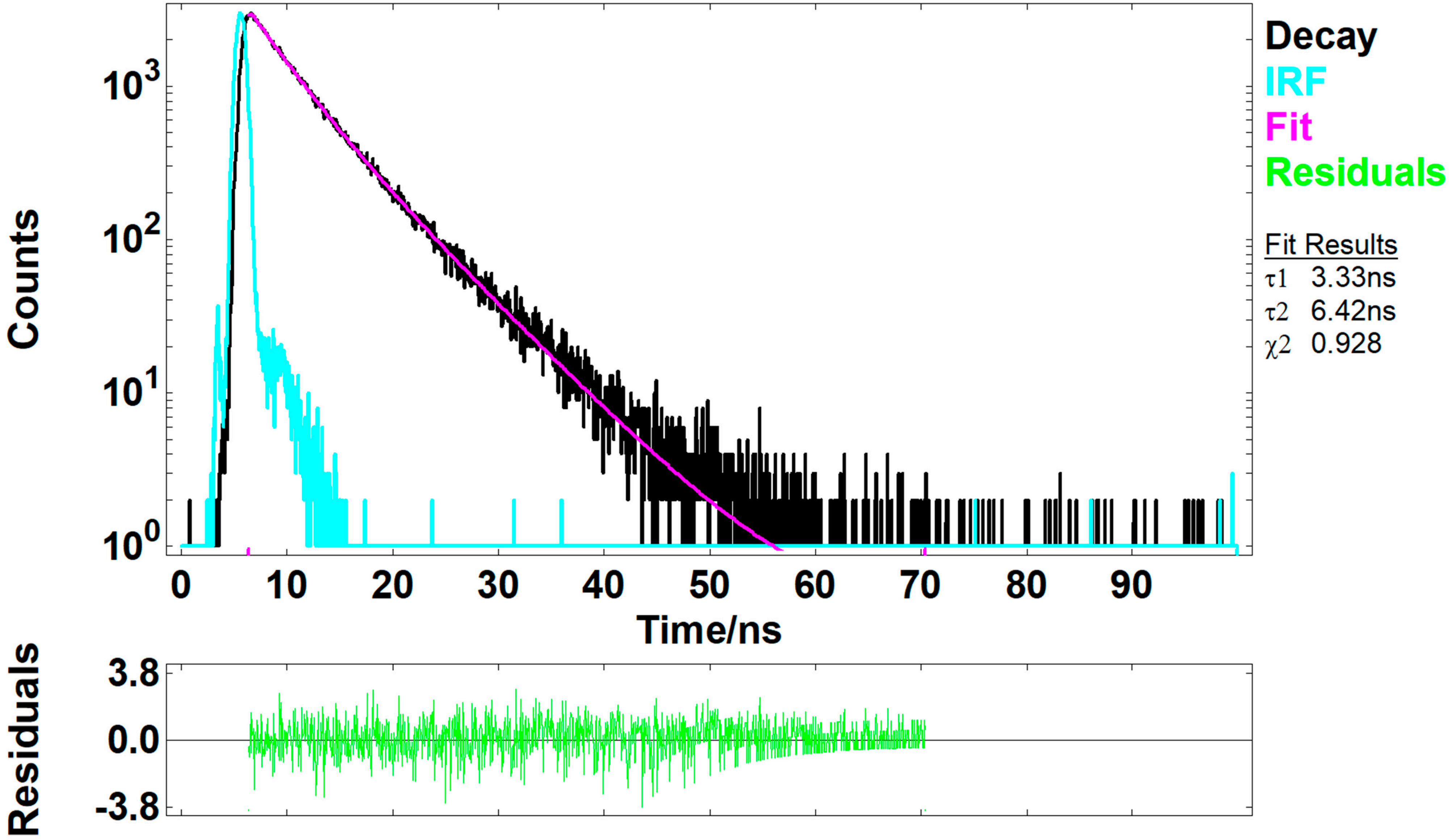

Figure S117. Fluorescence lifetime of **H13**.

Table S1. Crystal data and structure refinement details for studied compounds.

Compound	**H1**	**H2**	**H3**	**H4**	**H5**	**H6**	**H7**
CCDC number	2476861	2476860	2476858	2476863	2476859	2476862	2476850
Empirical formula	$C_{15}H_{13}N_3O$	$C_{16}H_{15}N_3O$	$C_{16}H_{16}N_4$	$C_{14}H_{11}N_3O$	$C_{15}H_{13}N_3O_2$	$C_{14}H_9Cl_2N_3$	$C_{14}H_{10}N_4O_2$
Formula weight	251.28	265.31	264.33	237.26	267.28	290.14	266.26
Temperature (K)	100.00(10)	100.00(11)	100.02(10)	201(140)	100.00(13)	100.00(10)	100.02(10)
Crystal system	monoclinic	monoclinic	monoclinic	triclinic	monoclinic	monoclinic	monoclinic
Space group	$P2_1/c$	$P2_1/n$	$P2_1/n$	P-1	$P2_1/c$	Cc	$P2_1/c$
a (Å)	7.49320(10)	8.23680(10)	5.96970(10)	8.02368(17)	13.9592(7)	3.81310(10)	4.96110(10)
b (Å)	10.32410(10)	10.80930(10)	7.38260(10)	11.5183(2)	13.6737(6)	24.7305(4)	17.0811(3)
c (Å)	16.8037(3)	15.7011(2)	30.8308(5)	13.37755(20)	6.8028(3)	14.2280(2)	14.8238(3)
α (°)	90	90	90	104.1109(14)	90	90	90
β (°)	94.502(2)	100.8310(10)	90.6840(10)	103.7380(16)	93.571(4)	96.171(2)	96.203(2)
γ (°)	90	90	90	95.6390(16)	90	90	90
Volume ($Å^3$)	1295.93(3)	1373.03(3)	1358.68(4)	1148.62(4)	1295.96(10)	1333.92(5)	1248.83(4)
Z	4	4	4	4	4	4	4
ρ_{calc} (g/cm^3)	1.288	1.283	1.292	1.372	1.370	1.445	1.416
μ (mm^{-1})	0.673	0.662	0.630	0.727	0.767	4.277	0.822
F(000)	528.0	560.0	560.0	496.0	560.0	592.0	552.0
Crystal size (*mm^3*)	0.2 × 0.09 × 0.06	0.17 × 0.08 × 0.05	0.275 × 0.2 × 0.165	0.212 × 0.082 × 0.074	0.068 × 0.050 × 0.038	0.161 × 0.037 × 0.025	0.297 × 0.048 × 0.04
Radiation	Cu Kα (λ = 1.54184)	Cu Kα (λ = 1.54184)	Cu Kα (λ = 1.54184)	Cu Kα (λ = 1.54184)	Cu Kα (λ = 1.54184)	Cu Kα (λ = 1.54184)	Cu Kα (λ = 1.54184)
2q range for data collect. (°)	10.064 to 159.246	9.994 to 159.856	5.734 to 158.822	7.078 to 158.922	6.344 to 158.824	7.148 to 158.736	7.924 to 158.978
Index ranges	$-9 \le h \le 9, -13 \le k \le 9, -19 \le l \le 21$	$-10 \le h \le 9, -13 \le k \le 13, -19 \le l \le 20$	$-6 \le h \le 7, -8 \le k \le 9, -38 \le l \le 35$	$-10 \le h \le 10, -14 \le k \le 14, -16 \le l \le 14$	$-17 \le h \le 14, -17 \le k \le 17, -8 \le l \le 8$	$-3 \le h \le 4, -30 \le k \le 29, -17 \le l \le 18$	$-5 \le h \le 6, -21 \le k \le 21, -18 \le l \le 14$
Reflections collected/ independent	13519/2710	25761/2912	14338/2866	20766/4703	3062/3062	12935/2380	12655/2616
R_{int}	0.0312	0.0352	0.0251	0.0246	0.0371	0.0328	0.0781
Data / restraints / parameters	2710/0/176	2912/0/185	2866/0/186	4703/0/333	3062/0/189	2380/2/175	2616/0/184
Goodness-of-fit on F^2	1.104	1.035	1.041	1.054	1.044	1.119	1.068
Final *R* indexes [I>2s(I)]	R_1 = 0.0340, wR_2 = 0.0874	R_1 = 0.0334, wR_2 = 0.0834	R_1 = 0.0354, wR_2 = 0.0884	R_1 = 0.0323, wR_2 = 0.0877	R_1 = 0.0315, wR_2 = 0.0864	R_1 = 0.0279, wR_2 = 0.0731	R_1 = 0.0541, wR_2 = 0.1527
R indexes (all data)	R_1 = 0.0392, wR_2 = 0.0987	R_1 = 0.0366, wR_2 = 0.0855	R_1 = 0.0377, wR_2 = 0.0898	R_1 = 0.0369, wR_2 = 0.0906	R_1 = 0.0359, wR_2 = 0.0889	R_1 = 0.0291, wR_2 = 0.0824	R_1 = 0.0624, wR_2 = 0.1603
Largest diff. peak and hole (e•$Å^{-3}$)	0.21/-0.27	0.21/-0.23	0.21/-0.23	0.27/-0.22	0.22/-0.21	0.27/-0.20	0.34/-0.33
Flack parameter	-	-	-	-	-	-0.012(11)	-

Table S1. *continued*

Compound	**H8**	**H9**	**H10**	**H11**	**H12**	**H13**	**H14**
CCDC number	2476857	2476852	2476853	2476854	2476851	2476856	2476855
Empirical formula	$C_{18}H_{18}N_4O$	$C_{12}H_9N_3S$	$C_{12}H_8N_4O_2S$	$C_{12}H_8N_4O_3$	$C_{15}H_{12}BrN_3$	$C_{16}H_{13}N_3$	$C_{16}H_{12}N_4O_2$
Formula weight	306.36	227.28	272.28	256.22	314.19	247.29	292.30
Temperature (K)	100.02(11)	100.02(11)	100.02(10)	100.00(10)	100.01(10)	100.00(10)	100.00(10)
Crystal system	monoclinic	monoclinic	monoclinic	triclinic	orthorhombic	monoclinic	monoclinic
Space group	*C*2	$P2_1/c$	$P2_1/c$	*P*-1	$P2_12_12_1$	$P2_1/c$	$P2_1/n$
a (Å)	33.3048(3)	6.9491(2)	3.79080(10)	7.19729(16)	4.19910(10)	7.84040(10)	8.29250(10)
b (Å)	5.58557(5)	15.8541(4)	27.4360(4)	8.6892(2)	13.5852(3)	10.35100(10)	11.46310(10)
c (Å)	34.7837(3)	10.0853(3)	11.2267(2)	20.3207(5)	23.3902(16)	16.3625(2)	14.38940(10)
α (°)	90	90	90	88.6777(19)	90	90	90
β (°)	103.0785(9)	91.238(3)	91.1170(10)	86.8647(19)	90	97.1770(10)	90.0580(10)
γ (°)	90	90	90	65.609(2)	90	90	90
Volume ($Å^3$)	6302.85(11)	1110.86(5)	1167.40(4)	1155.67(5)	1334.31(10)	1317.51(3)	1367.82(2)
Z	16	4	4	4	4	4	4
ρ_{calc} (g/cm^3)	1.291	1.359	1.549	1.473	1.564	1.247	1.419
μ (mm^{-1})	0.667	0.264	2.520	0.930	4.100	0.597	0.803
F(000)	2592.0	472.0	560.0	528.0	632.0	520.0	608.0
Crystal size (*mm*^{3})	0.62 × 0.034 × 0.028	0.4 × 0.11 × 0.07	0.119 × 0.027 × 0.023	0.26 × 0.035 × 0.021	0.300 × 0.052 × 0.029	0.148 × 0.079 × 0.071	0.17 × 0.05 × 0.03
Radiation	Cu Kα (λ = 1.54184)	Mo Kα (λ = 0.71073)	Cu Kα (λ = 1.54184)	Cu Kα (λ = 1.54184)	Cu Kα (λ = 1.54184)	Cu Kα (λ = 1.54184)	Cu Kα (λ = 1.54184)
2q range for data collect. (°)	5.448 to 159.656	5.138 to 62.696	6.444 to 158.492	8.716 to 157.698	7.526 to 163.232	10.134 to 158.328	9.866 to 159.034
Index ranges	-41 ≤ h ≤ 35, -7 ≤ k ≤ 6, -44 ≤ l ≤ 44	-9 ≤ h ≤ 9, -21 ≤ k ≤ 21, -13 ≤ l ≤ 14	-4 ≤ h ≤ 4, -32 ≤ k ≤ 33, -13 ≤ l ≤ 13	-9 ≤ h ≤ 9, -11 ≤ k ≤ 10, -25 ≤ l ≤ 24	-4 ≤ h ≤ 5, -17 ≤ k ≤ 15, -26 ≤ l ≤ 29	-9 ≤ h ≤ 9, -13 ≤ k ≤ 13, -20 ≤ l ≤ 16	-10 ≤ h ≤ 9, -14 ≤ k ≤ 12, -18 ≤ l ≤ 17
Reflections collected/ independent	61472/12308	26382/3260	16411/2412	31143	4143/4143	25983/2812	26557/2902
R_{int}	0.0590	0.0537	0.0277	0.0479	-	0.0291	0.0300
Data / restraints / parameters	12308/1/841	3260/0/148	2412/0/175	4734/0/349	4143/0/177	2812/0/175	2902/0/202
Goodness-of-fit on F^2	1.124	1.043	1.054	1.141	1.073	1.026	1.104
Final *R* indexes [*I*>2s(*I*)]	R_1 = 0.0466, wR_2 = 0.1106	R_1 = 0.0383, wR_2 = 0.0947	R_1 = 0.0299, wR_2 = 0.0722	R_1 = 0.0987, wR_2 = 0.2330	R_1 = 0.0603, wR_2 = 0.1679	R_1 = 0.0324, wR_2 = 0.0854	R_1 = 0.0342, wR_2 = 0.0870
R indexes (all data)	R_1 = 0.0517, wR_2 = 0.1140	R_1 = 0.0520, wR_2 = 0.1036	R_1 = 0.0345, wR_2 = 0.0767	R_1 = 0.1009, wR_2 = 0.2389	R_1 = 0.0639, wR_2 = 0.1730	R_1 = 0.0350, wR_2 = 0.0873	R_1 = 0.0379, wR_2 = 0.0969
Largest diff. peak and hole (e•$Å^{-3}$)	0.24/-0.22	0.36/-0.55	0.27/-0.28	1.49/-0.45	0.94/-1.30	0.18/-0.17	0.20/-0.23
Flack parameter	0.5*	-	-	-	0.01(3)	-	-

* Refined as a perfect inversion twin

Table S2. Selected bond lengths (Å) for the studied compounds.

Compound	N1—C1	C1—C2	C5—N2	N2—N3	N3—C8	C8—C9
**H1**	1.1515(16)	1.4321(16)	1.3666(15)	1.3681(13)	1.2815(15)	1.4605(16)
**H2**	1.1504(13)	1.4322(13)	1.3748(12)	1.3709(12)	1.2882(13)	1.4581(14)
**H3**	1.1507(15)	1.4369(15)	1.3684(14)	1.3718(12)	1.2858(14)	1.4547(14)
**H4**(i) 1st mol.	1.1503(14)	1.4348(14)	1.3711(13)	1.3639(12)	1.2894(13)	1.4527(14)
**H4**(i) 2nd mol.	1.1530(15)	1.4318(15)	1.3763(14)	1.3682(12)	1.2867(14)	1.4569(14)
**H5**	1.1482(14)	1.4329(15)	1.3781(13)	1.3664(13)	1.2874(14)	1.4585(15)
**H6**	1.146(5)	1.439(5)	1.370(4)	1.358(4)	1.284(4)	1.466(4)
**H7**	1.149(2)	1.437(2)	1.372(2)	1.3513(19)	1.285(2)	1.462(2)
**H8**(ii) 1st mol.	1.153(5)	1.427(5)	1.374(4)	1.359(4)	1.290(4)	1.457(5)
**H8**(ii) 2nd mol.	1.154(4)	1.433(5)	1.382(4)	1.367(4)	1.281(4)	1.463(4)
**H8**(ii) 3th mol.	1.150(5)	1.439(5)	1.370(4	1.361(4)	1.290(4)	1.454(4)
**H8**(ii) 4th mol.	1.148(5)	1.436(5)	1.374(4)	1.362(4)	1.288(5)	1.461(4)
**H9**	1.1532(18)	1.4336(18)	1.3725(16)	1.3625(15)	1.2924(17)	1.4412(18)
**H10**	1.153(2)	1.433(2)	1.378(2)	1.3467(18)	1.286(2)	1.445(2)
**H11**(i) 1st mol.	1.147(4)	1.442(4)	1.379(4)	1.345(4)	1.304(4)	1.433(4)
**H11**(i) 2nd mol.	1.180(4)	1.417(5)	1.377(4)	1.350(3)	1.277(4)	1.427(4)
**H12**	1.145(11)	1.435(11)	1.382(10)	1.361(10)	1.278(10)	1.480(11)
**H13**	1.1513(12)	1.4315(12)	1.3757(11)	1.3606(11)	1.2901(12)	1.4409(13)
**H14**	1.1515(16)	1.4294(16)	1.3736(15)	1.3647(14)	1.2923(15)	1.4388(17)

(i) Z'=2, two symmetry-independent molecules exist in the asymmetric unit; (ii) Z'=4, four symmetry-independent molecules exist in the asymmetric unit. The atom numbering in the second, third and fourth molecules corresponds to that of the first molecule as follows: N1→N21→N41→N61, C1→C21→C41→C61, etc.

Table S3. Selected bond angles (°) for the studied compounds.

Compound	N1—C1—C2	C5—N2—H2	N3—N2—H2	C5—N2—N3	N2—N3—C8	N3—C8—C9
**H1**	178.50(12)	120.6(9)	118.1(9)	121.15(9)	115.66(9)	122.14(10)
**H2**	177.68(11)	118.6(9)	119.2(8)	121.69(8)	114.50(8)	123.39(9)
**H3**	176.87(12)	118.5(9)	118.5(9)	122.63(9)	113.97(9)	124.07(10)
**H4**(i) 1st mol.	179.35(12)	119.5(9)	118.5(9)	121.90(9)	115.73(9)	122.01(9)
**H4**(i) 2nd mol.	179.28(12)	119.3(8)	119.9(9)	120.35(9)	117.23(9)	121.49(9)
**H5**	178.48(12)	120.8(9)	119.0(9)	120.25(9)	116.49(9)	120.85(10)
**H6**	178.8(4)	121(3)	116(3)	120.1(3)	116.1(3)	119.1(3)
**H7**	176.35(18)	118.4(14)	120.5(14)	120.83(14)	117.04(14)	119.80(14)
**H8**(ii) 1st mol.	179.0(4)	119(3)	120(3)	120.2(3)	116.7(3)	120.8(3)
**H8**(ii) 2nd mol.	178.6(4)	119(2)	119(2)	120.6(3)	115.5(3)	122.2(3)
**H8**(ii) 3th mol.	179.3(4)	119(3)	118(3)	122.5(3)	114.9(3)	122.6(3)
**H8**(ii) 4th mol.	178.7(4)	122(3)	115(3)	121.8(3)	116.5(3	122.6(3)
**H9**	178.52(15)	118.2(12)	120.3(12)	120.96(11)	115.37(11)	121.02(12)
**H10**	179.56(18)	119.9(13)	119.3(13)	120.47(8)	116.85(13)	119.31(14)
**H11**(i) 1st mol.	178.4(3)	117(2)	122(2)	120.6(3)	115.0(3)	118.2(3)
**H11**(i) 2nd mol.	177.1(3)	107(2)	130(2)	121.5(3)	116.2(3)	120.2(3)
**H12**	178.0(8)	113(5)	129(5)	117.7(6)	118.9(7)	115.7(7)
**H13**	178.51(10)	118.0(8)	121.3(8)	120.36(8)	116.77(8)	119.51(9)
**H14**	176.85(13)	119.3(10)	119.0(9)	120.94(10)	115.01(10)	121.37(11)

(i) Z'=2, two symmetry-independent molecules exist in the asymmetric unit; (ii) Z'=4, four symmetry-independent molecules exist in the asymmetric unit). The atom numbering in the second, third and fourth molecules corresponds to that of the first molecule as follows: N1→N21→N41→N61, C1→C21→C41→C61, etc.

Table S4. Interplanar angles (φ) between cyano-benzene and benzene/furan/thiophene ring mean planes, and selected torsion angles (χ) in the studied compounds.

Compound	φ (°)	χ [C5—N2—N3—C8] (°)	χ [N2—N3—C8—C9] (°)	χ [N3—C8—C9—C10] (°)	χ [C8—C9—C10—C11] (°)
**H1**	2.7	175.95 (10)	179.95 (9)	-	-
**H2**	7.7	-172.55 (9)	-179.43 (9)	-	-
**H3**	9.1	-172.73(10)	178.99(9)	-	-
**H4**(i) 1st mol.	4.8	-178.93(9)	-179.91(9)	-	-
**H4**(i) 2nd mol.	15.5	-175.54(9)	-177.12(8)	-	-
**H5**	5.7	-175.43(9)	179.76(9)	-	-
**H6**	10.6	-174.5(3)	179.2(3)	-	-
**H7**	15.0	174.40(14)	176.45(13)	-	-
**H8**(ii) 1st mol.	4.6	179.8(3)	-178.4(3)	-	-
**H8**(ii) 2nd mol.	8.1	-175.8(3)	178.7(3)	-	-
**H8**(ii) 3th mol.	5.2	-179.8(3)	-178.5(3)	-	-
**H8**(ii) 4th mol.	4.7	178.3(3)	179.2(3)	-	-
**H9**	5.1	175.93(11)	179.32(11)	-	-
**H10**	6.2	-179.97(14)	178.60(13)	-	-
**H11**(i) 1st mol.	1.6	179.6(2)	-178.8(2)	-	-
**H11**(i) 2nd mol.	2.0	-178.7(3)	-179.6(2)	-	-
**H12**	21.3	-170.9(8)	178.2(7)	-	-
**H13**	10.3	174.73(8)	178.32(8)	179.83(9)	-179.78(8)
**H14**	13.8	-175.05(10)	-176.25(10)	179.06(11)	-176.49(11)

(i) Z'=2, two symmetry-independent molecules exist in the asymmetric unit; (ii) Z'=4, four symmetry-independent molecules exist in the asymmetric unit. The atom numbering in the second, third and fourth molecules corresponds to that of the first molecule as follows: N1→N21→N41→N61, C1→C21→C41→C61, etc.

Table S5. Hydrogen bonds in studied compounds [$G_d^a(n)$ – hydrogen-bond pattern of primary graph level].

D-H•••A	d(D—H) (Å)	d(H•••A) (Å)	d(D•••A) (Å)	<(DHA) (°)	$G_d^a(n)$
			**H1**		
N2—H2•••N1(i)	0.89(2)	2.15(2)	3.038(1)	172(1)	C(8)
			**H2**		
N2—H2•••N1(ii)	0.89(1)	2.14(1)	3.018(1)	169(1)	C(8)
			**H4**		
O1—H1•••N3	0.88(1)	1.86(1)	2.658(1)	150(2)	S(6)
N2—H2•••N21(iii)	0.90(1)	2.16(1)	3.041(1)	165(1)	D(2)
O21—H21•••N23	0.89(2)	1.83(2)	2.654(1)	151(2)	S(6)
N22—H22•••N1(iv)	0.91(2)	2.22(2)	3.084(1)	158(1)	D(2)
			**H5**		
O1—H1•••N1(v)	0.88(2)	2.02(2)	2.842(1)	156(1)	C(15)
N2—H2•••N3(i)	0.86(2)	2.81(2)	3.517(1)	141(1)	C(3)
			**H6**		
N2—H2•••N1(vi)	0.79(4)	2.26(4)	3.046(4)	175(4)	C(8)
			**H7**		
N2—H2•••O1(vii)	0.85(2)	2.20(2)	3.008(2)	160(2)	C(10)
N2—H2•••N1(viii)	0.85(2)	2.91(2)	3.452(2)	124(2)	C(8)
			**H8**		
N2—H2•••N61(ix)	0.85(4)	2.21(4)	3.039(4)	166(4)	D(2)
N22—H22•••N1(x)	0.95(4)	2.08(4)	3.005(4)	165(4)	D(2)
N42—H42•••N21(xi)	0.86(4)	2.19(4)	3.022(4)	163(4)	D(2)
N62—H62•••N41(xii)	0.88(4)	2.16(4)	3.029(4)	171(4)	D(2)
			**H9**		

N2—H2•••N1(viii)	0.84(2)	2.23(2)	3.048(2)	163(2)	C(8)
		**H10**			
N2—H2•••N1(xiii)	0.86(2)	2.19(2)	3.042(2)	173(2)	C(8)
		**H11**			
N2—H2•••N1(xiv)	1.02(4)	2.48(4)	3.199(4)	127(3)	C(8)
N2—H2•••O2(xv)	1.02(4)	2.12(4)	2.970(4)	140(4)	$R_2^2(18)$
N22—H22•••N21(xvi)	1.01(4)	2.39(4)	3.181(4)	134(4)	C(8)
N22—H22•••O22(xvii)	1.01(4)	2.29(4)	2.946(4)	122(3)	$R_2^2(18)$
		**H12**			
N2—H2•••N1(xix)	1.1(1)	2.1(1)	3.164(2)	174(9)	C(8)
		**H13**			
N2—H2•••N1(xx)	0.89(1)	2.20(1)	3.076(1)	170(1)	C(8)
		**H14**			
N2—H2•••N1(xxi)	0.93(2)	2.14(2)	3.049(1)	166(1)	C(8)

Symmetry transformations used to generate equivalent atoms: (i) x,1.5-y,-1/2+z; (ii) 1/2+x,1/2-y,1/2+z, (iii) 2-x,1-y,2-z; (iv) 1-x,-y,2-z; (v) -1+x,y,1+z; (vi) 1+x,1-y,-1/2+z; (vii) -x,1/2+y,1/2-z; (viii) -1+x,1.5-y,-1/2+z; (ix) 1-x,-2+y,1-z; (x) x,-1+y,z; (xi) 1/2+x,-1/2+y,z; (xii) 1-x,-1+y,1-z; (xiii) -1+x,1/2-y,1/2+z; (xiv) 1+x,-1+y,z; (xv) 2-x,1-y,1-z; (xvi) x,1+y,z; (xvii) 1-x,2-y,-z; (xix) -x,-1/2+y,1.5-z; (xx) x,1/2-y,-1/2+z; (xxi) -1/2+x,1.5-y,-1/2+z;

Table S6. Stacking interactions in the studied compounds. Each ring, R, is indicated by one membered atom. The d is a distance between centroid of I, Cg(I), and centroid of J, Cg(J), α is a dihedral angle between planes of rings I and J, β is an angle between Cg(I)-Cg(J) vector and normal to plane I, d_p is a perpendicular distance of Cg(I) on the ring J plane.

I•••J	d (Å)	α (°)	β (°)	d_p (Å)
		**H4**		
R(C2)•••R(C9)(i)	4.0859(7)	4.78(5)	29.8	3.3690(5)
R(C2)•••R(C9)(ii)	4.1185(7)	4.78(5)	33.4	3.3325(5)
R(C22)•••R(C22)(iii)	3.8181(6)	0.02(5)	27.1	3.3991(5)
R(C22)•••R(C29)(iv)	4.2996(6)	15.46(5)	30.5	3.1832(4)
		**H5**		
R(C2)•••R(C9)(v)	3.5803(2)	5.74(5)	7.1	3.4962(4)
		**H9**		
R(S1)•••R(C2)(vi)	3.7894(8)	5.13(6)	29.2	3.4063(6)
		**H6**		
R(C2)•••R(C2)(vi)	3.813(2)	0.00(16)	26.7	3.4078(14)
R(C9)•••R(C9)(vi)	3.813(2)	0.03(16)	25.1	3.4517(14)
		**H10**		
R(S1)•••R(S1)(vi)	3.7908(9)	0.02(8)	26.9	3.3798(6)
R(C2)•••R(C2)(vi)	3.7908(9)	0.03(7)	27.8	3.3547(6)
		**H11**		
R(O1)•••R(C2)(vii)	3.664(2)	1.60(18)	24.7	3.3593(15)
R(O1)•••R(C2)(viii)	3.537(2)	1.60(18)	21.0	3.3269(15)
R(O21)•••R(C22)(ix)	3.622(2)	1.99(18)	21.9	3.3979(14)
R(2O1)•••R(C22)(x)	3.576(2)	1.99(18)	22.5	3.3390(14)
		**H12**		
R(C2)•••R(C2)(vi)	4.199(5)	0.0(4)	35.3	3.428(3)
R(C9)•••R(C9)(vi)	4.200(5)	0.0(4)	32.0	3.560(4)
		**H14**		
R(C2)•••R(C2)(v)	4.1563(7)	13.78(6)	27.9	3.1060(5)

Symmetry transformations used to generate equivalent atoms: (i) 1-x,1-y,2-z, (ii) 2-x,1-y,2-z; (iii) 1-x,-y,1-z; (iv) -x,-y,1-z; (v) 1-x,1-y,1-z; (vi) -1+x,y,z; (vii) 1-x,2-y,1-z; (viii) 2-x,2-y,1-z; (ix) 1-x,1-y,-z; (x) 2-x,1-y,-z.

Table S7. Absorption data from UV-Vis diffuse reflectance spectra (experimental) and quantum-mechanical calculations (theoretical) for the studied compounds.

Compound	Wavelength of absorption maxima (nm)		Oscillator strength
	Experimental	Theoretical	
**H1**	383	331	0.671
		337	1.158
		347	0.642
**H2**	324	225	0.303
		255	0.141
		302	0.139
	375	338	3.591
**H3**	249	231	0.118
	292	292	0.249
	407	342	1.463
**H4**	285	274	0.132
	318	292	0.207
	377	333	1.502
**H5**	268	214	0.125
	285	262	0.336
	375	352	1.803
**H6**	252	218	0.072
	322	271	0.476
	365	344	1.436
	395	364	0.899
**H7**	252	203	0.048
		247	0.091
	294	311	0.286
	352	377	0.844
	483	431	0.359
		475	0.366
**H8**	246	230	0.148
	330	353	2.415
	390	365	0.672
	478	-	-
	580	-	-
**H9**	293	258	0.299
	386	353	1.351
	408	359	1.107
	470	513	0.001
**H10**	276	312	0.421
	380	388	0.560
		424	0.831
	502	512	0.006
**H11**	265	226	0.107
		267	0.135
		312	0.807
	382	395	1.734
	513	477	0.510
**H12**	243	213	0.056
	296	255	0.273
	369	336	1.379
**H13**	250	231	0.259
	287	303	0.319
	398	352	4.360
**H14**	277	212	0.089
		269	0.100
	330	320	1.248

376	415	3.141
511	500	0.072

Table S8. Radiative (k_r) and non-radiative (k_{nr}) rate constants for solid-state fluorescence of 4-cyanophenylhydrazones.

Notes: Values were computed from the measured quantum yields (Φ_f) and mono-exponential lifetimes (τ) reported in the manuscript's Table 2, using $k_r = \Phi_f/\tau$ and $k_{nr} = (1-\Phi_f)/\tau$ with τ expressed in seconds. For entries with multiple excitation wavelengths (two Φ_f values) and a single τ per compound, the same τ was applied to both maxima, consistent with the excitation-invariant decay observed experimentally.

Compound	λ_{ex} (nm)	λ_{em} (nm)	Φ_f (—)	τ (ns)	k_r (s^{-1})	k_{nr} (s^{-1})
H1	343	440	0.23	0.94	2.45e+08	8.19e+08
H1	419	440	0.11	0.94	1.17e+08	9.47e+08
H2	338	425	0.25	0.72	3.47e+08	1.04e+09
H2	407	425	0.20	0.72	2.78e+08	1.11e+09
H3	362	462	0.22	12.50	1.76e+07	6.24e+07
H3	438	462	0.10	12.50	8.00e+06	7.20e+07
H4	372	434	0.02	0.45	4.44e+07	2.18e+09
H5	367	433	0.03	0.19	1.58e+08	5.11e+09
H6	357	463	0.07	1.18	5.93e+07	7.88e+08
H6	434	463	0.04	1.18	3.39e+07	8.14e+08
H8	344	446	0.05	0.58	8.62e+07	1.64e+09
H8	425	446	0.03	0.58	5.17e+07	1.67e+09
H12	366	421	0.02	1.33	1.50e+07	7.37e+08
H13	351	494	0.40	5.14	7.78e+07	1.17e+08
H13	439	494	0.22	5.14	4.28e+07	1.52e+08

Table S9. Fluorescence excitation and emission maxima for **H3** and **H13** phenylhydrazones in powder form and in PVP and PS polymer matrices.

Sample	λ_{ex} (nm)	λ_{em} (nm)	Fluorescence Intensity
**H3** powder	438	462	359
1% **H3**/PVP	413	454	158
1% **H3**/PS	398	442	57
**H13** powder	439	494	296
1% **H13**/PVP	420	477	44
1% **H13**/PS	420	475	25

Table S10. Photoluminescence quantum yields for the **H3** and **H13** penolhydrazones in powder form and dispersed in a PVP matrix.

Sampel	Excitation wavelength (nm)	Quantum yield (%)
**H3** powder	415	21,23
5% **H3**/PVP	415	20,20
1% **H3**/PVP	415	14,28
PVP_R	415	4,46
**H13** powder	405	22,35
5% **H13**/PVP	405	19,72
1% **H13**/PVP	405	14,52
PVP_R	405	0,52
